# Vaxjo 2.0: An ontology- and large language model-powered knowledge base of vaccine adjuvants and mechanisms

**DOI:** 10.3389/fcimb.2026.1763384

**Published:** 2026-06-05

**Authors:** Joshua Monickaraj, Hasin Rehana, Ani Bernardi, Yuping Zheng, Taiyu Lin, Le Liu, Amogh Madireddi, Leo Yeh, Jie Zheng, Junguk Hur, Yongqun He

**Affiliations:** 1 College of Literature, Science, and the Arts, University of Michigan, Ann Arbor, MI, United States; 2 Department of Biomedical Sciences, University of North Dakota, School of Medicine and Health Sciences, Grand Forks, ND, United States; 3 School of Electrical Engineering & Computer Science, University of North Dakota, Grand Forks, ND, United States; 4 School of Data Science, Chinese University of Hong Kong, Shenzhen, Guangdong, China; 5 Division of Life Science, The Hong Kong University of Science and Technology, Hong Kong, China; 6 Marine College, Shandong University, Weihai, Shandong, China; 7 College of Engineering, University of Michigan, Ann Arbor, MI, United States; 8 Unit for Laboratory Animal Medicine, University of Michigan, Ann Arbor, MI, United States; 9 Department of Learning Health Sciences, University of Michigan Medical School, Ann Arbor, MI, United States; 10 Center of Computational Medicine and Bioinformatics, University of Michigan Medical School, Ann Arbor, MI, United States

**Keywords:** immune profiles, knowledgebase, pattern recognition receptors (PRRs), vaccine adjuvant, vaccine ontology, Vaxjo

## Abstract

**Introduction:**

Vaccine adjuvants enhance immune responses by boosting vaccine efficacy, reducing required doses, and improving long-term immunity. The Vaxjo database is a web-based resource that stores information on vaccine adjuvants, including their names, storage conditions, structures, preparation methods, components, functions, safety, and references. The original version of Vaxjo, released in 2012 with 103 vaccine adjuvants, has been expanded and modernized as Vaxjo 2.0, a significantly enhanced version.

**Methods:**

In Vaxjo 2.0, newly identified adjuvants from biomedical literature retrieved through PubMed searches, as well as from the Vaccine Adjuvant Compendium (VAC) and AdjuvareDB, were added. To accelerate data collection and curation, we developed a vaccine adjuvant large language model (Vaxjo-LLM) system that automatically identifies and annotates new vaccine adjuvants and characterizes their mechanisms. The LLM-mined results were manually evaluated, annotated, and selectively included in the database to ensure quality.

**Results:**

Overall, Vaxjo 2.0 includes 448 vaccine adjuvants, organized into 16 distinct categories (e.g., mineral salt, emulsion, cytokine, peptide, and toll-like receptor (TLR) agonist vaccine adjuvants), all of which are represented in the Vaccine Ontology (VO) to streamline information storage and exchange. From 817 PubMed abstracts, Vaxjo-LLM identified mechanisms for 323 unique vaccine adjuvants across 16 mechanism families, including T cell activation/polarization, dendritic cell activation, TLR signaling, inflammasome activation, cytokine signaling, B cell/antibody production, and pattern recognition receptor (PRR) sensing. The mined information was subsequently manually reviewed to ensure consistency and accuracy. Based on this analysis, the mechanism profiles and clustering of the top 20 adjuvants were generated, revealing shared and distinct mechanistic signatures. For deeper mechanistic understanding, Vaxjo 2.0 further classified adjuvants based on PRR families, including TLRs, C-type lectin receptors, NOD-like receptors, and RIG-I-like receptors. Vaccine adjuvants were also categorized based on host immune response profiles, such as Th1/Th2/Th17-biased, Th1/Th2-mixed, and Treg-biased immune profiles.

**Discussion:**

The newly designed Vaxjo 2.0 web interface (https://violinet.org/vaxjo) provides an openly available and user-friendly platform for querying, visualizing, and analyzing vaccine adjuvant data.

## 1 Introduction

Vaccine adjuvants are compounds that enhance the host immune response to co-administered antigens in vaccines. In conjunction with vaccine antigens, adjuvants improve vaccine effectiveness through multiple biological processes. Vaccine adjuvants act by shaping the immune response profile, influencing both the magnitude and quality of the immune response, and prolonging the body’s response to antigens presented in vaccines (Zhao et al., 2023). They can enhance the production of antibodies, stimulate different types of T helper cells (Th1, Th2, Th17), and promote the formation of immunological memory, leading to stronger and more durable protection against the targeted pathogen. However, different adjuvants play distinct roles, and their specific roles and mechanisms often remain incompletely characterized.

Multiple vaccine adjuvant resources are currently available. Vaxjo, developed by our group, was the first comprehensive vaccine adjuvant database and analysis system that curated, stored, and analyzed vaccine adjuvants and their uses in vaccine development (Sayers et al., 2012). It is part of the Vaccine Investigation and OnLine Information Network (VIOLIN) (Xiang et al., 2008; He et al., 2014), which includes information, literature, and statistics regarding current vaccines, adjuvants, and their efficacy (Sayers et al., 2012). The vaccine adjuvants in Vaxjo are standardized by the Vaccine Ontology (VO) (Lin and He, 2012; Zheng et al., 2025). In addition to Vaxjo, AdjuvareDB provides a compendium of 331 potential adjuvants with composition, functions, and other features extracted from PubMed and clinical trials (Ren et al., 2024). The United States National Institutes of Health (NIH) has also actively funded vaccine adjuvant research, including the development of the Vaccine Adjuvant Compendium (VAC), a web-based database of vaccine adjuvants developed within NIH intramural programs (NIH-NIAID, 2025). VAC vaccine adjuvants are also standardized using VO (He et al., 2025; Zheng et al., 2025).

While a substantial amount of information about various vaccine adjuvants is stored in different databases, a significant portion of mechanistic knowledge remains buried in unstructured literature and is not represented in structured repositories. To enhance vaccine adjuvant data collection and analysis, large language models (LLMs) offer a promising approach by enabling automated extraction and synthesis of complex biological information at scale. Recent studies have surveyed LLM applications for named entity recognition and scientific knowledge extraction in biomedicine, highlighting both achievements and challenges in domain adaptation and mechanistic interpretation (Garcia et al., 2024; Lu et al., 2024). Our team’s work has also demonstrated the utility of LLMs in vaccine adjuvant research through VaxLLM, a fine-tuned large language model for systematically annotating vaccine components (Li et al., 2024). Additionally, we developed methods for recognizing cancer vaccine adjuvant names using LLMs, facilitating accurate identification and characterization of adjuvants in biomedical texts (Rehana et al., 2025). These developments support the incorporation of LLM-driven meta-analysis in Vaxjo 2.0, expanding its capacity to systematically annotate a broader landscape of vaccine adjuvants and their immune response mechanisms.

An important consideration in vaccine adjuvant research is species specificity. Adjuvant mechanisms characterized in animal models, predominantly murine, may not directly translate to human immunology due to differences in receptor expression, signaling pathways, and immune cell composition across species. For example, TLR expression patterns differ between mice and humans, and some adjuvants that are effective in mice may show reduced or altered activity in humans. Additionally, most adjuvant studies rely on mouse models, with relatively fewer studies employing non-human primates or other species that may be more predictive of human responses. In terms of the regulatory landscape, only a limited number of adjuvants have been approved by the U.S. Food and Drug Administration (FDA) for use in licensed human vaccines, including aluminum salts, AS01B, AS04, MF59, and CpG 1018. This gap between the large number of adjuvants studied in preclinical models and those approved for human use underscores the importance of systematic databases like Vaxjo 2.0 that can facilitate cross-species comparison and support translational adjuvant research.

This paper presents Vaxjo 2.0, a significantly updated version of the Vaxjo database that expands the coverage through secondary annotation, incorporation of VAC entries, VO-based modeling, and LLM-assisted meta-analysis of adjuvant mechanisms. In addition to the collection, storage, and display of vaccine adjuvants, we systematically explored the immune mechanisms of various vaccine adjuvants, linking mechanistic summaries to ontology-based representations to enable transparent querying and reuse.

## 2 Methods

### 2.1 General pipeline of vaccine adjuvant collection and annotation

**Figure 1:**
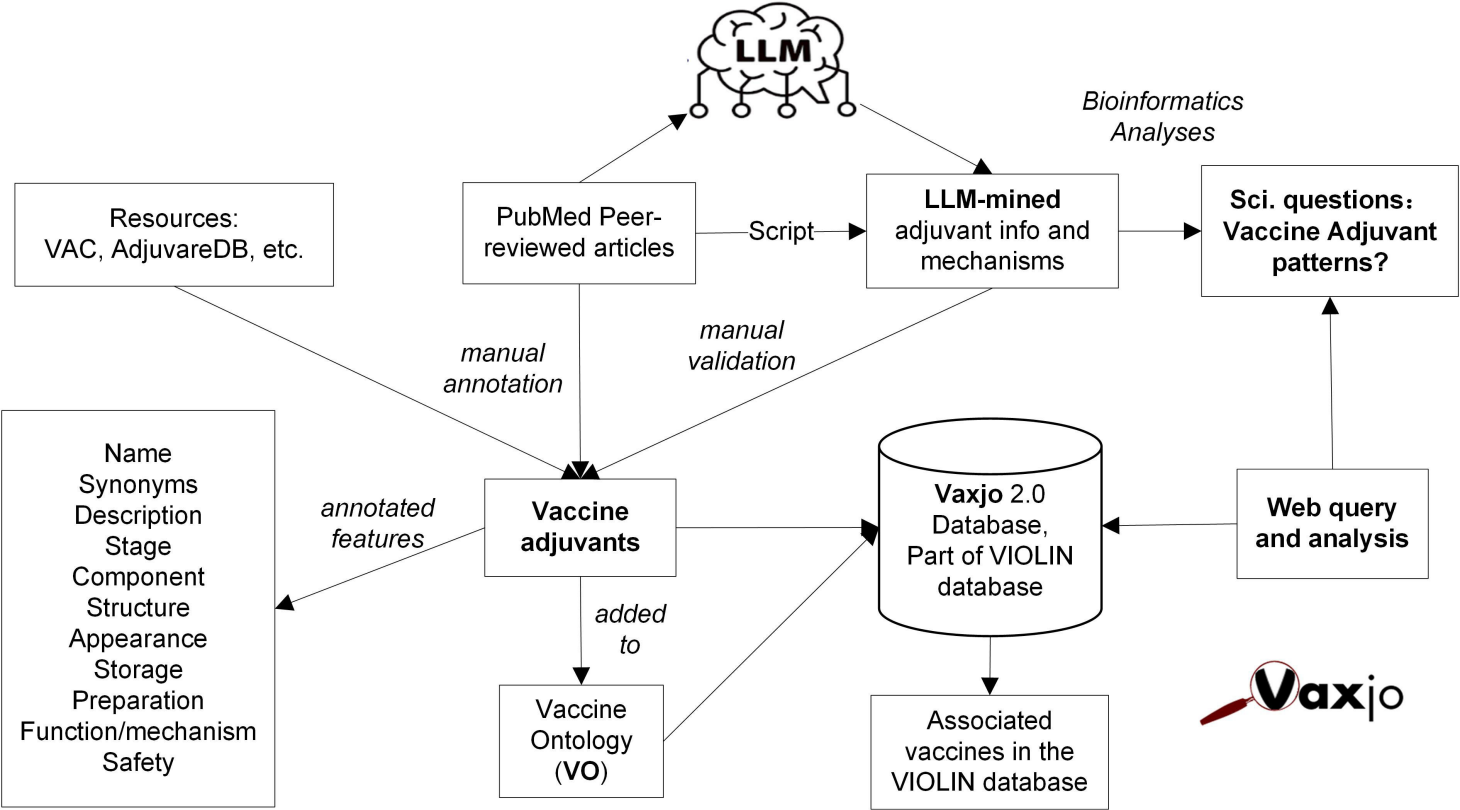
Overall Vaxjo 2.0 project workflow. Overview of the Vaxjo 2.0 data-processing pipeline. Vaccine adjuvants were collected from PubMed, VAC, and AdjuvareDB through manual curation and LLM-assisted extraction of vaccine adjuvants and their immune mechanisms. All entries were standardized in the Vaccine Ontology (VO) and integrated into the VIOLIN database.

The overall workflow of the Vaxjo 2.0 project is illustrated in **Figure 1**. Specifically, vaccine adjuvant information from public resources, including PubMed, VAC (NIH-NIAID, 2025), and AdjuvareDB (Ren et al., 2024), was collected, extracted, annotated, and stored in the Vaxjo vaccine adjuvant module of the VIOLIN database. Two primary methods were employed. The first utilized the VIOLIN bioinformatics pipeline with manual annotation and verification (Xiang et al., 2008; He et al., 2014), while the second incorporated LLM-assisted processing focused on extracting vaccine adjuvant mechanisms from the biomedical literature (see Section 2.2). All newly identified information not included in Vaxjo 1.0 was independently reviewed by at least one domain expert prior to being released to the public. It should be noted that the adjuvant studies curated in Vaxjo 2.0 span diverse animal models, including murine, non-human primate, porcine, avian, and bovine systems, as well as human clinical data where available. Because adjuvant mechanisms can vary across species, the host organism information is recorded alongside each adjuvant entry to support species-aware interpretation of mechanistic annotations.

Following their identification, information regarding each adjuvant was obtained from published articles. Each adjuvant has its corresponding published article(s) listed in its VO entry (**Supplementary File 1**). The manually curated data were then submitted to the VIOLIN platform, specifically the Vaxjo module, which compiles scientific evidence on vaccines and adjuvants into a centralized and structured knowledgebase. Each newly added adjuvant was assigned a corresponding VO identifier to streamline information storage, standardization, and interoperability. This approach enabled consistent curation, ensuring data integrity and comparability across multiple annotation sources. Overall, the updated system enables researchers with an enriched database for comparing vaccine adjuvants across efficacy, safety, and immune response parameters, supporting data-driven vaccine design and optimization. This combined manual and semi-automated workflow ensured consistency across entries and enabled the incorporation of both newly reported adjuvants and those detected through LLM-assisted extraction of immune mechanisms from the literature.

### 2.2 LLM analysis of vaccine adjuvant mechanisms

**Figure 2:**
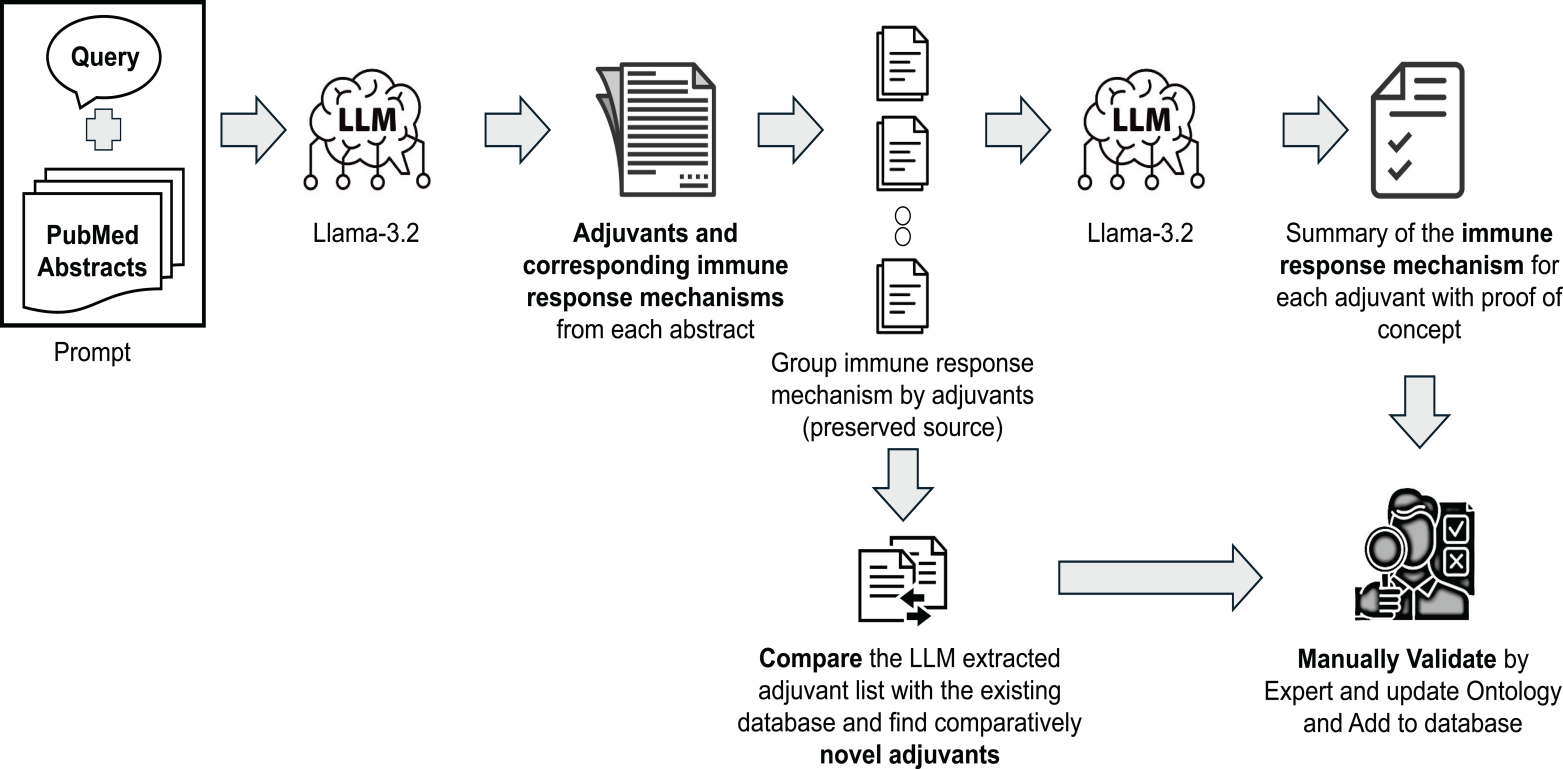
LLM pipeline for extracting and summarizing vaccine adjuvant mechanisms. Detailed view of the automated extraction module shown in **Figure 1**. In Phase I, vaccine adjuvants and associated immune-response mechanisms were extracted from PubMed abstracts and normalized to canonical adjuvant labels. In Phase II, aggregated mechanism evidence was summarized and structured into mechanism subtypes with supporting PMIDs. Structured outputs were reviewed prior to integration into Vaxjo 2.0.

To complement manual curation, we implemented a two-phase LLM-assisted workflow (**Supplementary File 2**) to extract and organize information on vaccine adjuvant mechanisms from PubMed abstracts (**Figure 2**). This workflow represents the automated extraction module within the broader Vaxjo 2.0 pipeline shown in **Figure 1**.

In Phase I, the Llama-3.2 3B model was used to identify vaccine adjuvants and extract their associated immune-response mechanisms from individual abstracts using a standardized prompt (**Supplementary File 3**). Each abstract was submitted to the Llama-3.2-3B-Instruct model using a chat-style structure consisting of a system instruction and a user-provided abstract. The model output was constrained to a predefined JSON schema containing structured fields for adjuvant name and immune-response mechanism description. These outputs were stored as plain-text records indexed by PMID, then programmatically parsed to extract structured fields (PMID, adjuvant, mechanism text) into tabular form for downstream analysis. This structured intermediate representation enabled systematic normalization, aggregation, and traceability to source abstracts.

An intermediate normalization step was applied to harmonize adjuvant names across abstracts using a deterministic, rule-based procedure. Specifically, extracted adjuvant names were mapped to canonical identifiers using a curated mapping dictionary that collapsed spelling variants, capitalization differences, Unicode representations (including Greek characters), formatting artifacts, trademarks, and known aliases. The normalization procedure employed exact string substitutions, case-insensitive matching, and Unicode normalization, while preserving biologically distinct formulations (e.g., MPLA vs. GLA-SE vs. MF59). The mapping dictionary was developed iteratively by reviewing the complete set of extracted adjuvant names and aligning them with established immunological nomenclature. After normalization, mechanism text from all abstracts referring to the same canonical adjuvant was concatenated to create a single aggregated mechanism evidence block per adjuvant, while preserving PMID traceability to the source abstracts.

In Phase II, each adjuvant and its aggregated mechanism text were passed to a second LLM prompt (**Supplementary File 4**). This prompt instructed the model to generate (i) a concise multi-sentence summary of the adjuvant’s immune mechanisms and (ii) a structured list of mechanism subtypes, each linked to supporting PMIDs. This phase transformed abstract-level evidence into adjuvant-level mechanistic representations.

Finally, LLM-generated mechanism subtypes were standardized using a rule-based regular-expression classifier that mapped free-text mechanism phrases to a predefined set of mechanistic families. These families were derived by reviewing the full set of LLM-generated mechanism phrases, identifying recurring immunological themes, and grouping them into biologically coherent categories (e.g., T cell activation/polarization, dendritic cell activation, TLR signaling, cytokine signaling, and inflammasome activation). Each family was associated with a set of keyword-based pattern-matching rules, for example, phrases containing “TLR2/4/7/9,” “MyD88,” or “TRIF” were assigned to the TLR signaling family; mentions of “caspase-1,” “pyroptosis,” or “inflammasome” were mapped to NLRP3 inflammasome activation; and cytokine names such as “IL-6,” “IFN-γ,” or “TNF” were mapped to cytokine signaling/production.

For both phases, standardized prompts (**Supplementary Files 3**, **4**) were used to ensure reproducibility. All LLM-generated annotations were subsequently reviewed and refined by domain experts to verify accuracy, resolve ambiguities, and align with established terminology and ontology standards. Only expert-validated annotations were incorporated into the Vaxjo 2.0 database, ensuring that the final mechanistic dataset was both comprehensive and accurate. All code, LLM prompts, and pattern-matching rules used in this two-phase extraction workflow are provided in **Supplementary File 2** and are also available in a maintained Vaxjo-LLM GitHub repository and in a Zenodo repository (see details below).

### 2.3 Ontological representation and analysis of vaccine adjuvants

The VO (Lin and He, 2012; Zheng et al., 2025) was used to represent all the vaccine adjuvants. For each newly added vaccine adjuvant, a unique VO identifier was assigned using the format “VO_#######”. Each VO term also includes a term label, textual definition, references, immune profiles, target receptors, and other related metadata. Parent-child relationships were also established to position each adjuvant under its appropriate higher-level category. The new vaccine adjuvant terms with associated information were added in a CSV format and converted to OWL ontology format using the ROBOT template feature (Jackson et al., 2019), and all added vaccine adjuvants were validated using Protégé 5.6.4 OWL editor (Musen and Protege, 2015). To enable semantic exploration, Description Logic (DL) queries were developed for retrieving adjuvant-related data directly from VO. This approach ensured machine-readable, semantically interoperable representation of all adjuvants curated in Vaxjo 2.0.

### 2.4 Website query and analysis

The Vaxjo 2.0 website (https://violinet.org/vaxjo) provides an updated, user-friendly interface for querying vaccine adjuvants. Users can perform keyword searches across multiple fields, including adjuvant name, structure, components, function, storage, and safety, and can also filter results by vaccine type, pathogen, and host species. Each adjuvant entry is linked to detailed metadata, including its VO identifier, structure, description, and references to associated vaccines.

### 2.5 Data access, sharing, and license

The Vaxjo 2.0 website is freely accessible and serves as the primary portal for curated vaccine adjuvant data. Associated datasets, including VO-based models and annotated entries, are freely available for download. VO, which provides the underlying ontological structure for Vaxjo, is publicly available on GitHub (https://github.com/vaccineontology/VO). The Vaxjo-LLM pipeline is available on GitHub (https://github.com/hurlab/Vaxjo-LLM), and supporting materials for the current study are archived on Zenodo (https://zenodo.org/records/17808635).

## 3 Results

### 3.1 Data quantification of collected vaccine adjuvants in Vaxjo 2.0

Using the annotation pipeline defined in **Figure 1**, the Vaxjo 2.0 development team collected and annotated a total of 448 vaccine adjuvants, derived from various resources. In addition to the 103 adjuvants from the original Vaxjo, Vaxjo 2.0 added 345 new vaccine adjuvants, including 88 from VAC (NIH-NIAID, 2025), 233 from AdjuvareDB (Ren et al., 2024), and 25 newly identified by the LLM from VO-recorded vaccine adjuvant-associated literature in PubMed (**Supplementary File 1**).

For each vaccine adjuvant collected, we annotated various types of information, including the vaccine adjuvant name, synonyms, components, structure, development stage, storage, preparation, function, mechanism, and safety. A complete listing of all curated adjuvants and their associated metadata is provided in **Supplementary File 1**. Different from AdjuvareDB, Vaxjo provides many additional adjuvant data fields, such as targeted receptors and immune profiles. We have also extracted specific adjuvant mechanisms based on our LLM and manual annotation.

VAC is an NIH intramural resource that stores all newly identified vaccine adjuvants resulting from NIH funding. For example, ‘Alhydroxiquim-II’ is recorded in VAC as ID 12 (https://vac.niaid.nih.gov/view?id=12). As a Toll-Like Receptor (TLR) 7/8 small-molecule agonist chemisorbed on aluminum hydroxide (Alhydrogel), Alhydroxiquim-II activates TLR7/TLR8 signaling in lymph nodes and induces a Th1 immune response (Counoupas et al., 2022). Alhydroxiquim-II has served as the adjuvant for BBV152 (COVAXIN), the inactivated COVID-19 vaccine made by Bharat Biotech International, Hyderabad, India (Vadrevu et al., 2022). This adjuvant is now annotated and included in Vaxjo as Vaxjo ID 113 (https://violinet.org/vaxjo/vaxjo_detail.php?c_vaxjo_id=113).

### 3.2 Ontological modeling and classification of vaccine adjuvants using the vaccine ontology

**Figure 3:**
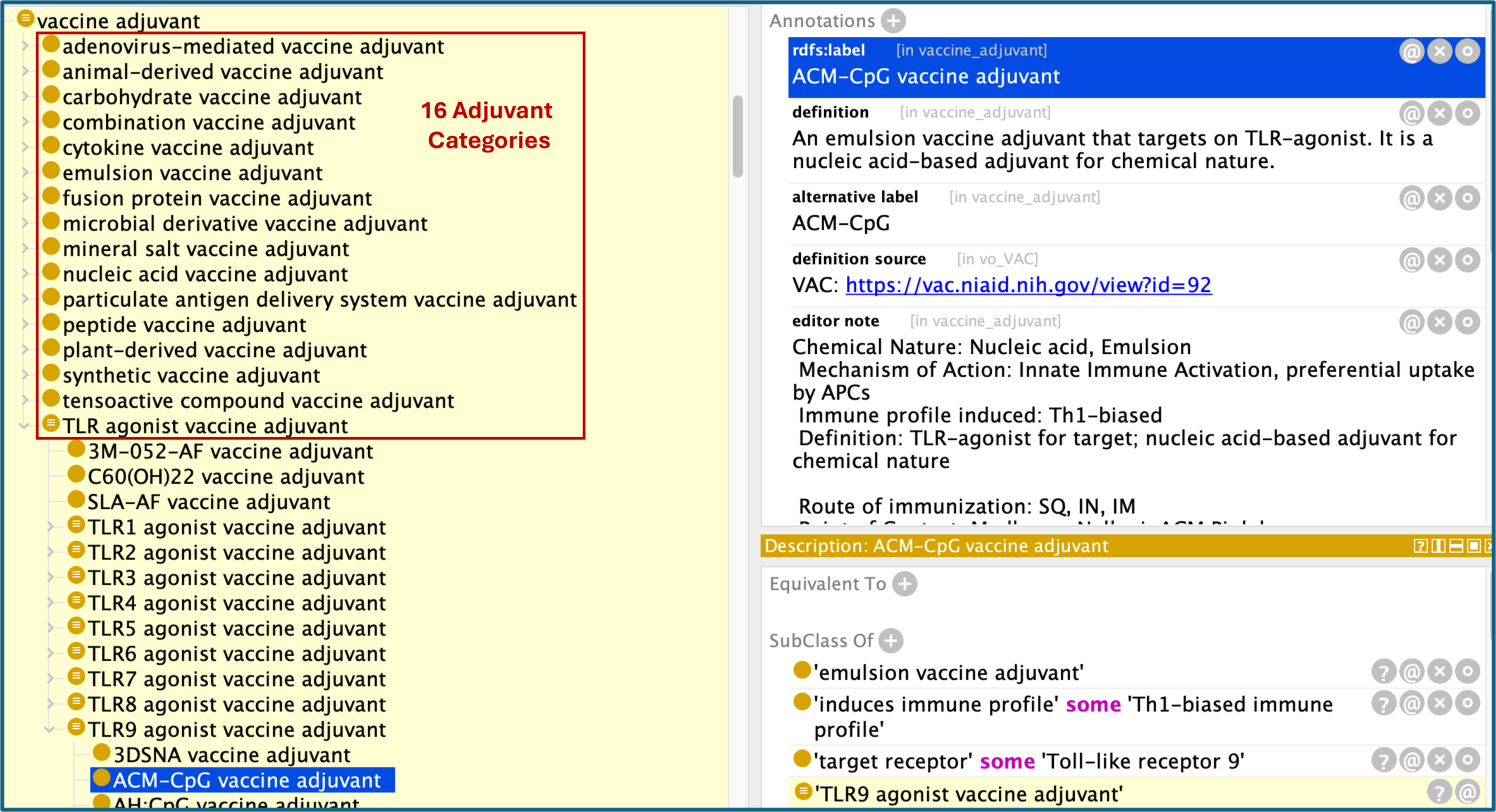
Ontological classification of representative vaccine adjuvants in VO. The image illustrates hierarchical VO-based categorization of vaccine adjuvants according to their sources, compositions, and immunological functions. The red box at the top left highlights 16 distinct vaccine adjuvant categories defined in the VO. On the right, the ontological representation of an example adjuvant,‘ACM-CpG vaccine adjuvant,‘ is shown. Annotations for this adjuvant, including its label, textual definition, and associated published articles, are displayed at the top right. At the bottom right, logical axioms associated with the adjuvant are presented, such as its parent category, immune mechanisms, and the inferred parent, ‘TLR9 agonist vaccine adjuvant,‘ which is highlighted with a yellow background.

All 448 vaccine adjuvants in Vaxjo are represented in the VO. The VO classifies these adjuvants into 16 distinct categories (**Figure 3**), according to their sources, compositions, and functions. Based on sources, adjuvants can be animal-derived, plant-derived, or microbial derivatives. According to biological compositions, there are carbohydrate, nucleic acid, peptide, cytokine, fusion protein, and adenovirus-mediated vaccine adjuvants. Based on chemical compositions, vaccine adjuvant categories include mineral salt, tensioactive compound, synthetic, emulsion, and combination vaccine adjuvants. By immunological functions, adjuvants are classified as particulate antigen delivery systems or TLR agonist vaccine adjuvants.

In addition to specifying the parent category of each vaccine adjuvant, the VO standardizes each adjuvant’s label and provides a textual definition, along with other available annotations, such as synonyms. Moreover, the immune mechanisms of adjuvants are represented as logical axioms in the VO, enabling computational reasoning. For example, **Figure 3** shows the representation of the ‘ACM-CpG vaccine adjuvant’ in the VO. ‘ACM-CpG vaccine adjuvant’ is classified as an ‘emulsion vaccine adjuvant’ and is associated with two logical axioms:

‘induces immune profile’ some ‘Th1-based immune profile’

‘target receptor’ some ‘Toll-like receptor 9’

Through reasoning, this adjuvant is also inferred to be a ‘TLR9 agonist vaccine adjuvant,’ since the category ‘TLR9 agonist vaccine adjuvant’ is defined as:

‘vaccine adjuvant’ and (‘target receptor’ some ‘Toll-like receptor 9’)

This ontological framework supports semantic consistency and enables computational reasoning over adjuvant relationships across datasets.

### 3.3 Identification and classification of vaccine adjuvant mechanisms using LLM

In this study, a two-phase LLM-based natural language processing (NLP) workflow was applied to systematically identify and classify mechanisms of vaccine adjuvants from PubMed abstracts. The LLM component served three primary roles: (1) discovering new adjuvants not previously captured in manual curation, (2) identifying mechanistic descriptions that were subsequently merged with expert-reviewed annotations, and (3) statistically analyzing mechanistic patterns across different adjuvants.

**Figure 4:**
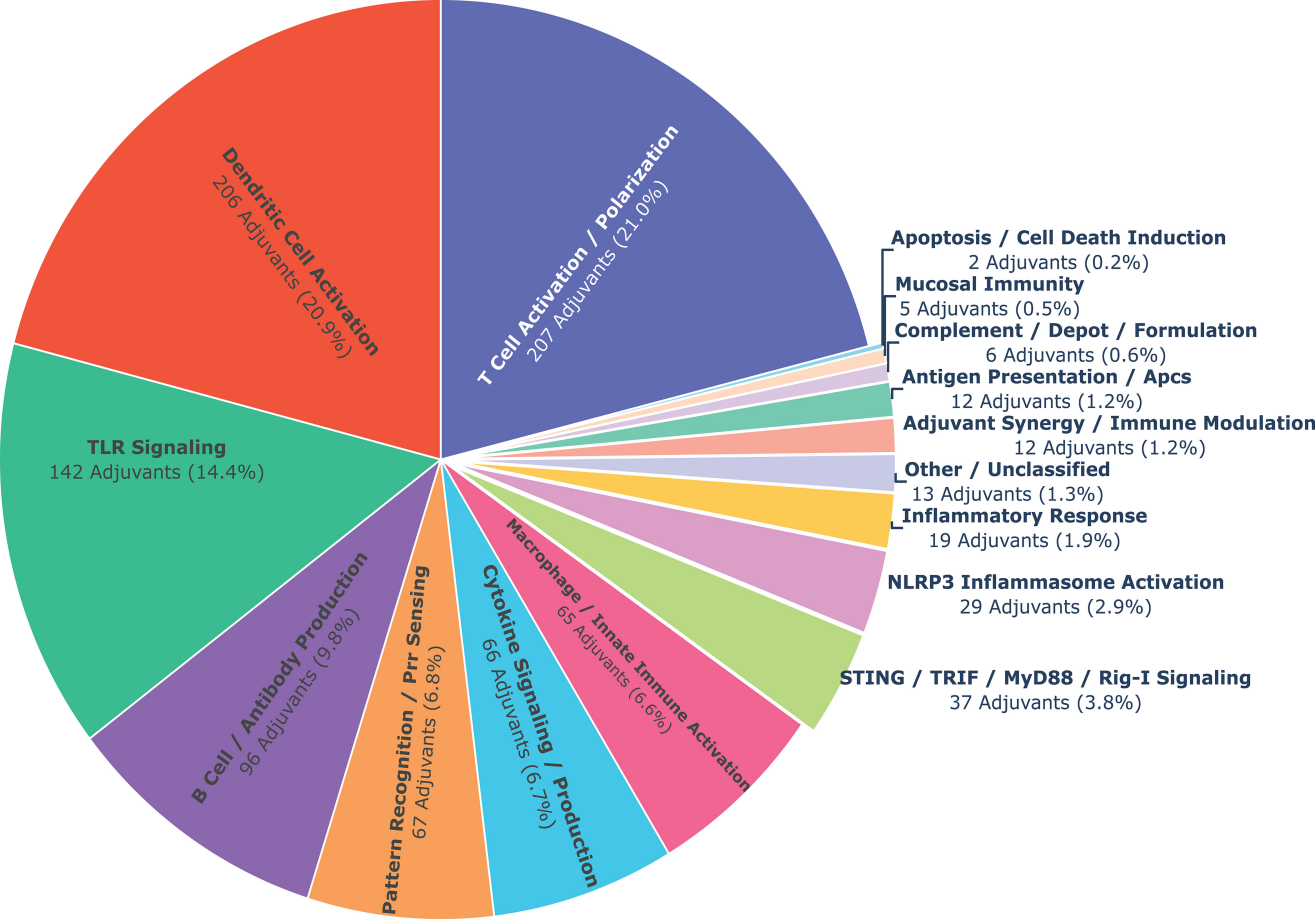
Distribution of adjuvants across LLM-predicted mechanism families. This chart illustrates the distribution of 323 unique adjuvants across mechanism families. The numbers in each slice indicate the total count and percentage of adjuvants associated with each mechanism family.

Across 817 PubMed abstracts, the pipeline identified mechanistic annotations for 323 unique vaccine adjuvants, which were organized into 16 predefined mechanism families. These families represent major immunological processes, including T cell activation/polarization, dendritic cell activation, TLR signaling, inflammasome activation, cytokine signaling, B cell/antibody production, PRR sensing, and others. A hierarchical analysis revealed the specific subtypes contributing to each mechanism family. T cell activation/polarization (associated with 207 unique adjuvants) and dendritic cell activation (206 adjuvants) were the two most common mechanisms, followed by TLR signaling (142 adjuvants). Additional families included B cell/antibody production (96 adjuvants), pattern recognition/PRR sensing (67 adjuvants), cytokine signaling/production (66 adjuvants), and macrophage/innate immune activation (65 adjuvants). Less frequent families involved NLRP3 inflammasome activation or mucosal immunity. The ‘Other/Unclassified’ category contained only 13 adjuvants, confirming broad coverage across mechanistic types. The overall distribution of adjuvants across the 16 mechanism families is summarized in **Figure 4** and discussed below. Representative LLM extraction and summarization outputs used to generate these mechanism-family assignments are provided in **Supplementary Files 3**, **4**.

To evaluate the performance and reliability of the LLM-based extraction, we further analyzed the identified adjuvants and their mechanistic annotations. The LLM identified 348 adjuvants, 301 of which were partial or exact matches to existing adjuvants in Vaxjo. Of the 44 identified as having no match to existing adjuvants, 13 were duplicates that incorrectly returned no match due to differences in names such as abbreviations. Six were incorrectly identified as adjuvants entirely. The remaining 25 were correctly identified as novel adjuvants. These were added to Vaxjo after subsequent review by a domain expert. Out of the 301 adjuvants, we randomly selected 50 and assessed them for mechanistic accuracy. A total of 178 mechanism subtypes were identified, of which 146 were correct, for an overall accuracy of 82% (**Supplementary File 5**).

One common error type involved articles that mentioned or compared multiple adjuvants with differing mechanisms. Sometimes, the LLM would mix up which mechanism corresponded to each adjuvant. Another common error type was authors hypothesizing about a mechanism that was not experimentally supported, which could then be incorrectly identified as definitively true by the LLM. Lastly, some primary literature itself contains outdated or incorrect information that may be propagated by LLM.

**Figure 5:**
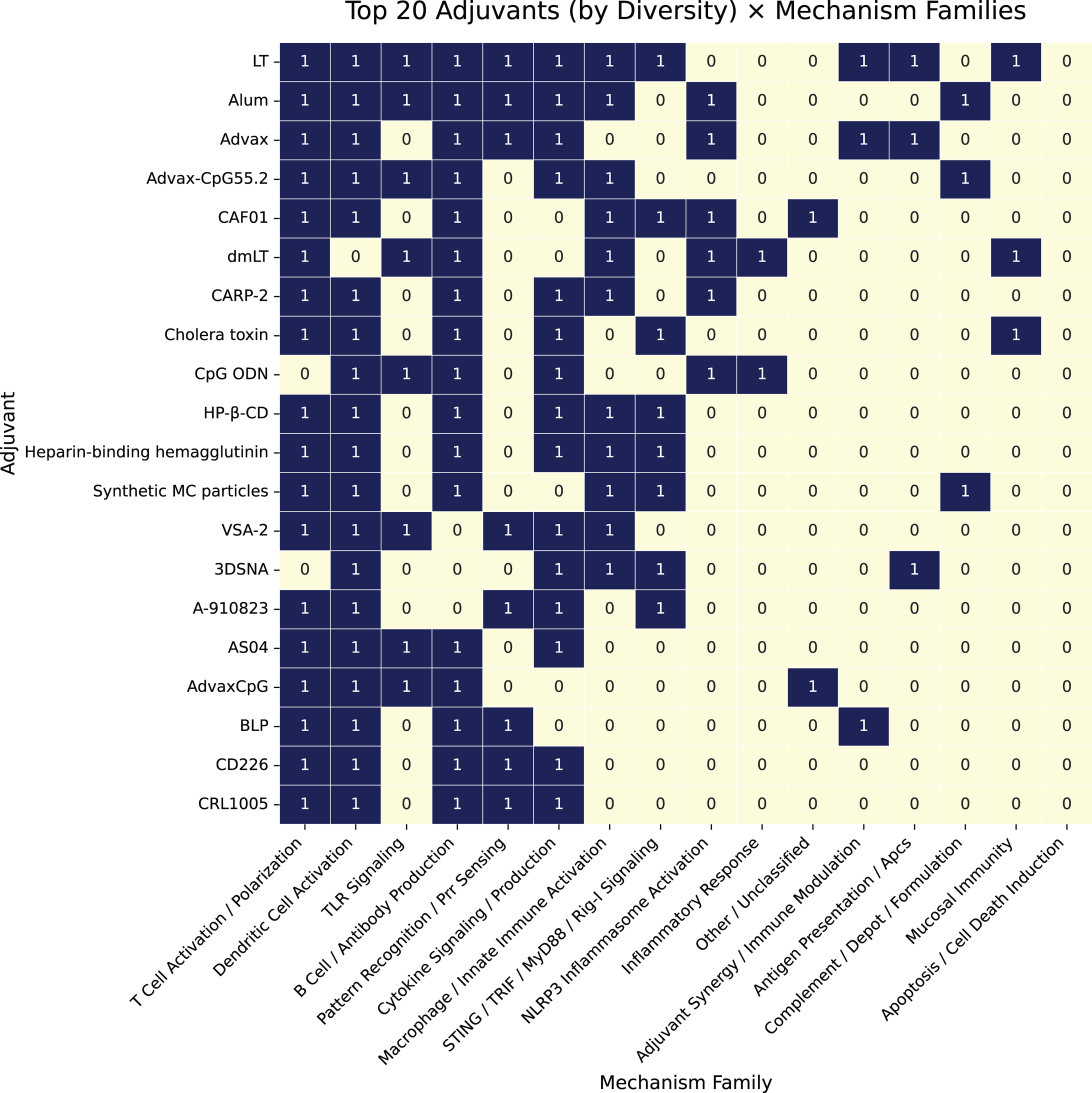
Mechanism profiles and clustering of the top 20 vaccine adjuvants based on LLM analysis. “1” means association, and “0” means no association. This heatmap displays the mechanism profiles of the top 20 adjuvants (ranked by mechanistic diversity) across mechanism families (ranked by prevalence) derived from normalized LLM-predicted mechanism subtypes.

To visualize adjuvant-specific patterns, a heatmap was generated profiling the top 20 adjuvants (ranked by mechanistic diversity) against the mechanism families (**Figure 5**). The heatmap reveals distinct mechanistic signatures. While many adjuvants engage in common pathways, such as T cell activation, dendritic cell activation, and B cell/antibody production, the heatmap shows distinct grouping patterns that highlight unique mechanistic profiles. Known TLR agonists (e.g., MPLA, CpG ODN, Poly(I:C)) cluster with “TLR Signaling”. Alum displays a distinct pattern, linked primarily to “NLRP3 Inflammasome Activation” and “Complement/Depot/Formulation”. Saponins (QS-21, Matrix-M) are associated with T/B cell activation and “Antigen presentation/APCs”, while emulsions (AS03, MF59) and STING agonists (cGAMP, c-di-GMP) form their own groups (**Figure 5**). A complete binary adjuvant-by-mechanism association matrix for all 323 adjuvants is provided in **Supplementary File 6**. A global UpSet visualization derived from this matrix, illustrating higher-order overlaps among mechanism families across all adjuvants, is provided in **Supplementary File 7**. These **Supplementary Materials** enable direct inspection of all combinatorial overlaps underlying **Figures 4** and **5** and support independent downstream analysis. These results highlight diverse activation strategies and reveal functional convergence among structurally unrelated adjuvants.

Together, the LLM-derived analyses provide a systems-level perspective on how various vaccine adjuvants interact with immune pathways. These analyses show that many adjuvants act through overlapping or complementary mechanisms that converge on shared innate immune processes, including dendritic cell activation, cytokine signaling, and inflammasome activation. To further delineate the molecular basis of these responses, we next examined adjuvants according to the specific pattern recognition receptors (PRRs) they target. Because many of the mechanisms identified through LLM analysis originate from PRR-mediated signaling, this receptor-based classification provides a complementary framework linking molecular recognition to the broader functional categories described above.

### 3.4 Classification of vaccine adjuvants based on pattern recognition receptors

The innate and adaptive immune systems are the two primary branches of host defense against pathogens, with innate pattern recognition mechanisms providing one of the initial signals that guide downstream adaptive responses. Based on the LLM-derived functional classifications, vaccine adjuvants were next categorized according to their experimentally validated or literature-supported PRR interactions (**Table 1**). PRRs recognize two main patterns, microbe- or pathogen-associated molecular patterns (MAMPs/PAMPs) and damage-associated molecular patterns (DAMPs), to initiate signaling pathways that connect innate and adaptive immunity (Ong et al., 2021; Chen et al., 2025).

**Table 1:** Classification of vaccine adjuvants based on PRRs. These are sorted based on PRR receptor types and the number of vaccine adjuvants.

Host receptors	Number of adjuvants in Vaxjo	Representative examples
Targeting TLRs: 158		
TLR4	80	MPLA, GLA-SE, GLA-AF, GLA-Alum, LPS, AS01, AS02, AS04
TLR2	32	Pam3Cys, CL429, OMPC, NanoVax/NE01, P. acnes, CoVaccine HT™, Fuc-Sc, CS3
TLR7	30	Imiquimod, Alhydroxiquim-II, 3M-052-SE, INI-4001, 1V270, UM-3003/4/5, Resiquimod, RNAdjuvant
TLR9	26	ISS 1018 CpG ODN, CpG 7909, Advax-CpG55.2, K3-SPG, S-540956, CpG M362, 3DSNA, ACM-CpG
TLR3	11	Poly(I:C), Poly IC: LC, PIKA, CAF09b, CAF05, Nexavant, DVG-Derived Oligonucleotides, CAF24a
TLR8	13	Resiquimod, 3M-052-SE, INI-4001, UM-3003/4/5, Alhydroxyquim-II, TLR7/8 RNA ligands, Algel-IMDQ
TLR5	5	Flagellin, MSP-102, SAG1+TLR5 Ligand Fusion, FLAB-PSPA
TLR1	1	Pam3Cys
Targeting CLR: 26		
Dectin-1	8	CVC1302, Candida Skin Test Reagent, Algal Glucan, Chitin, Chitosan, Glycated Chitosan, Zymosan, CS3
Dectin-2	3	Bl-Eng2
Mincle	17	CAF01, CAF09b, Bl-Eng2, CAF06, TDM, MPL+TDM, MMG-1, CAF19
CLEC9A	1	CBP-12
Targeting NLR: 19		
NOD1	2	PI-RI, PI-sRI
NOD2	17	MDP, AdDP, GMDP, Murametide, Murapalmitine, D-Murapalmitine, Theramide, TMDP
Targeting RLR: 8		
RIG-I	5	DVG-Derived Oligonucleotides, 3pRNA, NP-3pRNA-CpG, PIKA, VISA
MDA5	5	MDA5, Poly(I:C), Poly IC: LC, DVG-Derived Oligonucleotides, PIKA
Targeting cGAS: 8		
cGAS	8	c-di-AMP, cGAMP, c-di-GMP, glycated chitosan, chitosan, chitin, STING V155M, LP-GMP

Vaxjo 2.0 classifies adjuvants into four principal PRR families: (i) toll-like receptors (TLRs), (ii) C-type lectin receptors (CLRs), (iii) nucleotide-binding oligomerization domain (NOD)-like receptors (NLRs), and (iv) retinoic acid-inducible gene receptors or RIG-I-like receptors (RLRs). Two additional PRR groups include absent in melanoma (AIM2)-like receptors (ALRs) (Caneparo et al., 2018) and cyclic guanosine monophosphate-adenosine monophosphate synthase (cGAS) (Van Herck et al., 2021). Among these, TLRs and CLRs are primarily membrane-bound receptors, whereas the others are cytosolic receptors (Chen et al., 2025).

Many TLR agonists in this family function as potent vaccine adjuvants (Kayesh et al., 2023). These receptors detect pathogens and cellular damage, stimulate immune cells, and trigger proinflammatory responses and cytokine production, thereby linking innate and adaptive immune systems and generating strong and durable immune protection against diseases. Found on cell surfaces and in endosomes, examples of TLR agonists include imiquimod (a TLR7 agonist), lipopolysaccharide (LPS), monophosphoryl lipid A (MPL, a TLR4 agonist), and CpG oligodeoxynucleotides (CpG ODN, TLR9 agonists). TLR1 and TLR6 cannot trigger signaling independently and require dimerization with TLR2 for ligand recognition. Similarly, TLR2 cannot activate signaling as a monomer and requires heterodimerization with TLR1 or TLR6 for activation (Farhat et al., 2008). For adjuvants known to activate TLR2 but lacking confirmation of their heterodimer component, these were classified under TLR2.

CLRs are located on the cell surface and recognize major carbohydrate structures such as β-glucans and mannan found in fungal cell walls (Geijtenbeek and Gringhuis, 2009; Hardison and Brown, 2012). As one of the best characterized CLRs, transmembrane Dectin-1 is N-glycosylated and predominantly expressed by myeloid cells, including monocytes, macrophages, DCs, and neutrophils (Brown and Crocker, 2016). Dectin-1 can recognize β-1, 3-glucan-containing carbohydrates found predominantly in fungal cell walls. Dectin-2 also has an important role in antifungal immunity and serves as a direct receptor for mannose-capped lipoarabinomannan of mycobacteria (Yonekawa et al., 2014), suggesting its role in host immunity against mycobacterial infection. Mincle (macrophage inducible C-type lectin receptor) recognizes trehalose dimycolate (TDM) and works with Dectin receptors to activate the NLRP3 (NLR family, pyrin domain-containing 3) inflammasome, demonstrating cross-talk between receptor pathways (Brown and Crocker, 2016). Collectively, Dectin-1, Dectin-2, and Mincle are all targeted by vaccine adjuvants represented in Vaxjo (**Table 1**).

NOD-like receptors (NLRs), including key members NOD1 and NOD2, are intracellular receptors that recognize bacterial components like muramyl dipeptide (MDP) and meso-DAP from peptidoglycan and initiate proinflammatory signaling and cytokine production upon bacterial recognition (Kim et al., 2016). Vaccine adjuvants such as PI-RI (i.e., RIBI Natural) (Lukehart et al., 2022) and muramyl dipeptide (MDP) (Girardin et al., 2003) (**Table 1**) are recognized by NOD1 and NOD2, respectively. Note that many other types of NLRs are also available and targeted by vaccine adjuvants. For example, NLRP3 (i.e., NLR ‘pyrin’ domain containing 3) protein is a core sensor of the NLRP3 inflammasome, a multiprotein complex that can be activated by danger signals such as pathogens or adjuvants like Alum and QS-21 (Marty-Roix et al., 2016) to trigger caspase-1 activation and IL-1β and IL-18 secretion (Paik et al., 2021). Notably, some oxidized lipids, such as oxidized low-density lipoprotein (oxLDL), a valuable vaccine adjuvant (Habets et al., 2010), can activate NLRP3, while some other lipids inhibit its inflammasome activity, highlighting the context-dependent regulation of innate immune signaling (Liang et al., 2021).

RLRs are intracellular receptors that detect viral RNA, including mRNA (Rehwinkel and Gack, 2020). Eight vaccine adjuvants targeting RLRs, including RIG-I (retinoic acid-inducible gene I) and MDA5 (melanoma differentiation-associated protein 5), were identified in Vaxjo. A representative example is 3pRNA (5’-triphosphate RNA), an effective immune-stimulating adjuvant that activates RIG-I and triggers a strong type I interferon (IFN-I) response, leading to robust cytotoxic T lymphocyte (CTL) responses (Hochheiser et al., 2016). Polyinosinic-polycytidylic acid (Poly(I:C)) and its stabilized derivatives Poly IC: LC (stabilized with poly-L-lysine and carboxymethylcellulose) and PIKA (stabilized with kanamycin and calcium), have been used as an adjuvant for the development of rabies vaccines (Nicholson, 2016; Zhang et al., 2016) and COVID-19 vaccines (Lim et al., 2024).

### 3.5 Classification of vaccine adjuvants based on host immune response (outcomes) profiles

We have classified Vaxjo adjuvants by the adaptive immune response they induce, summarized as T helper cell polarization (**Table 2**). Adjuvants that promote a skewed response to a single T helper subset were labeled as inducing a biased response, while adjuvants that promote responses toward multiple T helper subsets were labeled as inducing a mixed response. Th1-biased responses were the most frequent category, followed by Th1/Th2 balanced responses.

**Table 2:** Classification of vaccine adjuvants based on adaptive immune response profiles.

Immune profile	No. of adjuvants in Vaxjo	Representative examples
Th1-biased	254	MPLA, CpG ODNs, Poly(I:C), Resiquimod, GLA-SE, Imiquimod, cGAMP, 3pRNA
Th2-biased	29	Alum, Incomplete Freund’s Adjuvant, Calcium Phosphate, Etx B, Mastoparan 7, LTR72, AF03, Bupivacaine
Th17-biased	7	Dibutyl phthalate, IL-1R1, CTA1-M2e-DD, UM-1098, Pam3Cys, Vaxfectin
Treg-biased	3	IL-27, Kynurenine, AAV-IL-27
Th1/Th2 balanced	100	MF59, Matrix-M, ISCOMs, AS15, 2B182C, Gerbu, (PVP)-057, SMNP
Th1/Th17 balanced	21	CAF01, Adjuplex + GLA, IL-1β, CAF05, CAF06, dmLT, NanoVax, NE01, Retinoic Acid + IL-15
Th2/Th17 balanced	4	Bordetella Pertussis Component, CTA1- DD Gene Fusion Protein, CTB, LTK63
Th1/Th2/Th17 balanced	6	STING V155M, Cholera Toxin, CPDI-02, LTA-1, T-Vant, c-di-GMP
Tfh/Th17 balanced	1	IL-21
Total	425	

Adjuvants that induce Th1-biased responses were connected to a variety of PRRs, including TLRs (MPLA, CpG ODNs, Resiquimod), cGAS, and RIG-I (cGAMP and 3pRNA). Th2-biased responses and Th1/Th17 mixed responses were the next largest categories, with moderate representation. Adjuvants with Th2-biased responses were typically less dependent on PRR-mediated signaling but involve diverse mechanisms. Two mechanistic categories are particulate depot formation (Alum and CaP) and emulsion-based depot formation (IFA, AF03). The remaining immune profile types made modest contributions, with each totaling single-digit counts (**Table 2**).

One special category of adjuvant-associated adaptive immune response profile is the Regulatory T (Treg) cell response (**Table 2**). The discovery and functional characterization of Treg cells have recently received major scientific recognition (Hori et al., 2003; Vignali et al., 2008) and were awarded the 2025 Nobel Prize in Physiology or Medicine (Bluestone, 2025). While vaccine adjuvants boost immune responses to vaccine antigens, Treg cells suppress or regulate immune responses. As a result, adjuvants can either inhibit Treg cell function to enhance immunogenicity or promote Treg induction to create a regulatory or tolerogenic immune environment (Keijzer et al., 2013; Zheng et al., 2023). Some adjuvants are designed to suppress Treg cells to enhance the desired immune response, while a growing class of tolerogenic adjuvants is specifically designed to promote Treg cells and induce a regulatory response (Ndure and Flanagan, 2014; Zheng et al., 2023).

### 3.6 Vaccine adjuvant query using the Vaxjo web interface or semantic VO-based search

**Figure 6:**
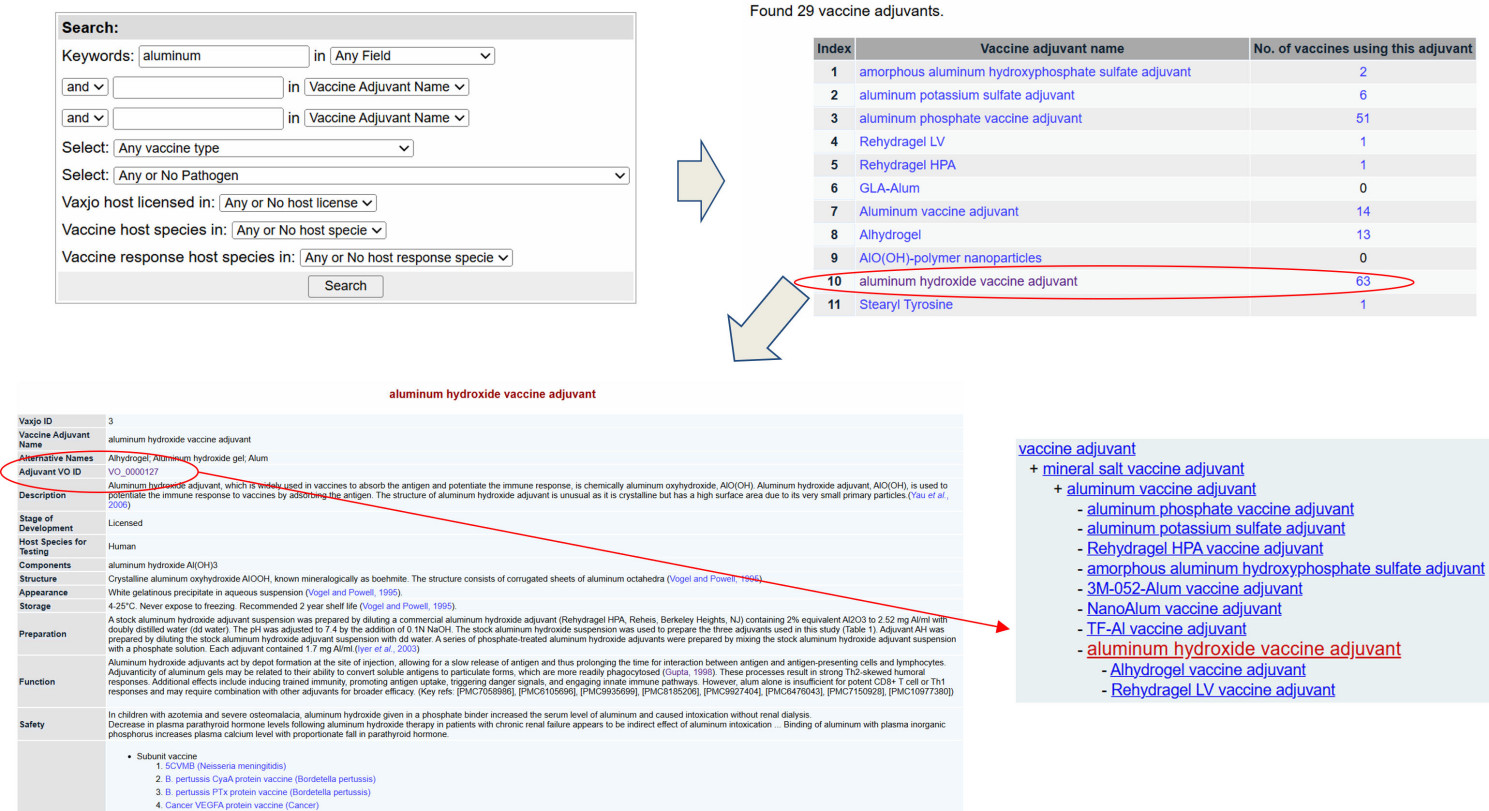
Vaxjo web query and exploration demonstration.

As demonstrated in **Figure 6**, the Vaxjo web program provides a user-friendly interface for querying vaccine adjuvants. In this example, the keyword “aluminum” returned 29 vaccine adjuvants. Among them, aluminum hydroxide vaccine adjuvant has been used in 63 vaccines recorded in the VIOLIN database. Selecting this adjuvant opens a dedicated webpage containing detailed metadata, including VO identifier, description, structure, storage information, and the list of vaccines in which it is used. Clicking the VO identifier (VO_0000127) directs users to its Ontobee (Ong et al., 2017) page, which presents the ontological classification and annotation of this specific vaccine adjuvant.

The Vaxjo website has incorporated LLM-mined mechanistic information to enhance its adjuvant annotations. For example, the page for ‘aluminum hydroxide vaccine adjuvant’ (https://violinet.org/vaxjo/vaxjo_detail.php?c_vaxjo_id=3) includes a section on adjuvant function, which has incorporated LLM-mined information. In this process, we first prompted an LLM to generate a mechanism-oriented summary with references. After manual annotation, only verified content was merged with the existing manually annotated information to produce an updated, quality-controlled version.

**Figure 7:**
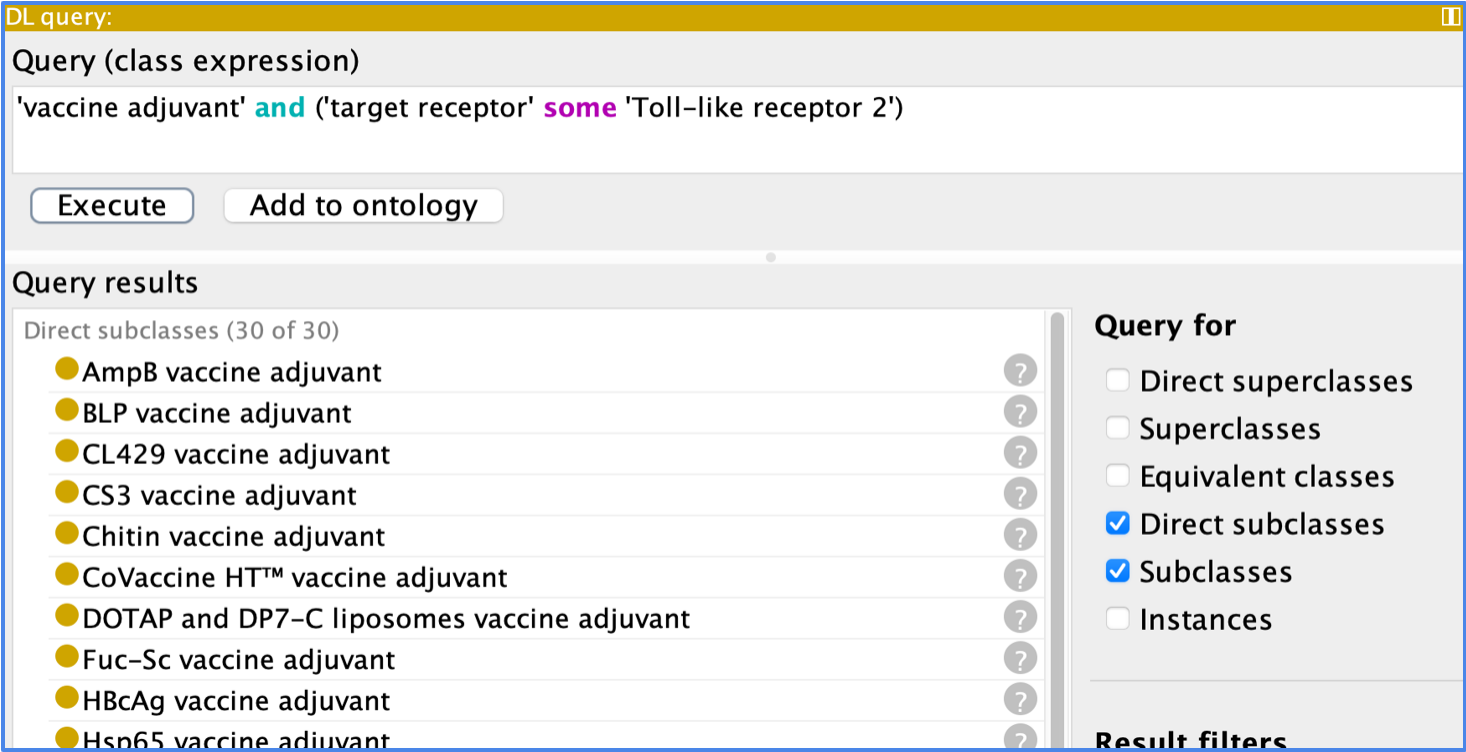
DL query example for finding vaccine adjuvants targeting the TLR-2 receptor. The DL query was executed using the Protégé-OWL editor. The query identified 30 vaccine adjuvants that target the TLR-2 receptor.

Another way to access the Vaxjo vaccine adjuvant information is through the Vaccine Ontology (VO), which stores information on all Vaxjo vaccine adjuvants in a computer- and human-understandable manner (He et al., 2009; Lin and He, 2012). The ontology enables semantic searches on vaccine adjuvant knowledge using DL queries (Baader, 2003). For example, the vaccine adjuvants can be queried based on their target receptors within the VO, as shown in **Figure 7**. In this example, the following DL query was used, resulting in 30 specific vaccine adjuvants:

‘vaccine adjuvant’ and (‘target receptor’ some ‘Toll-like receptor 2’)

## 4 Discussion

This article makes several contributions. First, we introduced the updated vaccine adjuvant database Vaxjo 2.0, which includes newly added adjuvants and updated web features. Second, we presented our LLM methods for vaccine adjuvant extraction from the literature. Third, we described our systematic analysis of vaccine adjuvant mechanisms, with two focus areas: pattern recognition receptors and adjuvant-induced immune response profiles. Furthermore, we provide ontological classification and data download services to the community.

The Vaxjo database compiles scientific evidence about various vaccine adjuvants from different resources, including VAC, AdjuvareDB, and LLM extraction from PubMed articles, into a centralized location. During this process, we standardized adjuvant names and classified them into 16 categories. We also added information on adjuvants’ target receptors and induced immune profiles, which facilitate the analysis of adjuvants’ immunological mechanisms. In addition, LLM-assisted extraction enabled the identification of additional adjuvants from the literature that had not yet been curated in existing databases. All data entries were manually annotated before being incorporated into both the Vaxjo database and the VO. This study updated the Vaxjo website, enabling researchers to compare the efficacy, safety, and immune response profiles of various adjuvants.

Vaxjo focuses on the ontological representation and analysis of a wide variety of vaccine adjuvants and their integration into the wider VIOLIN database. In comparison, VAC focuses on adjuvants developed through NIAID-funded research programs. AdjuvareDB focuses on the potential of adjuvants to be used in cancer immunotherapy through pan-cancer analysis and an emphasis on clinical trials.

To further illustrate the complementary nature of these resources, we compare the three databases across key dimensions. Vaxjo 2.0 curates 448 adjuvants from PubMed, VAC, and AdjuvareDB with VO-based standardization, LLM-assisted mechanism extraction, PRR-based and immune-profile classifications, and integration into the VIOLIN platform. VAC catalogs adjuvants developed within NIH-NIAID intramural programs, with entries also standardized in the VO. AdjuvareDB focuses on 331 candidate adjuvants with emphasis on clinical trials and pan-cancer immunotherapy applications. While all three databases aim to organize vaccine adjuvant knowledge, Vaxjo 2.0 uniquely provides ontology-grounded mechanistic annotations, multi-label mechanism family assignments, and systematic LLM-assisted extraction. A detailed comparison is provided in **Table 3**.

**Table 3:** Comparison of Vaxjo 2.0 with existing vaccine adjuvant databases.

Feature	Vaxjo 2.0	VAC	AdjuvareDB
Number of adjuvants	448	88 (included in Vaxjo)	331
Ontology standardization	VO-based (all entries)	VO-based	No
LLM-assisted extraction	Yes (Llama-3.2-3B)	No	No
Mechanism classification	16 mechanism families (multi-label)	Limited	Functional descriptions
PRR/immune profile data	Yes (**Tables 1**, **2**)	No	Limited
Clinical trial focus	No (literature-based)	NIH intramural focus	Yes (pan-cancer)
Open access/download	Yes (VIOLIN, GitHub, Zenodo)	Yes (NIH website)	Yes (website)

To the best of our knowledge, this paper represents the first application of large language models to systematically characterize vaccine adjuvant mechanisms. In our study, the LLM served as an effective tool for transforming unstructured literature into structured mechanistic knowledge. Rather than replacing expert curation, the LLM acted as a curation assistant, rapidly identifying relevant adjuvant–mechanism relationships and helping to organize mechanistic descriptions into coherent themes. This significantly reduced the burden on manual reviewers, who traditionally must read hundreds of abstracts to extract comparable information. Importantly, all LLM-derived insights were reviewed, corrected when necessary, and integrated only after expert validation, ensuring both accuracy and consistency. By combining automated text extraction with expert review, this work enabled the identification of mechanistic patterns that would be difficult to capture through manual reading alone.

However, because the current approach relies mainly on abstracts and one model configuration, and because mechanistic knowledge continues to evolve, periodic updates will be necessary to maintain coverage and accuracy. In addition, abstract-level information often lacks important experimental details such as adjuvant formulation, dosage, delivery systems, and the strength of supporting evidence. The absence of these details may limit the depth of mechanistic interpretation that can be derived directly from abstracts. When additional clarification was required, we manually examined the corresponding full-text articles to verify and supplement the extracted information. Future improvements of the LLM pipeline could incorporate full-text literature to provide richer mechanistic context.

It should be noted that although the LLM was used to assist in extracting information from literature, the classifications reported in the published articles are often not definitive. For example, caspase 1 was frequently studied and emphasized in many studies; however, the mouse models used by most groups were often deficient in both caspase 1 and caspase 11 (Kayagaki et al., 2011; Man et al., 2017; Downs et al., 2020). Consequently, some results attributed to caspase 1 may originate from inaccuracies in the original papers, which raises concerns about the reliability of the information added into the database. Similarly, there is significant debate and controversy regarding vaccine adjuvants that target NLRP3, as it is often unclear whether these adjuvants truly interact with the complex in many cases (Franchi and Nunez, 2008; Li et al., 2008; Spreafico et al., 2010).

Because Vaxjo derives its mechanistic annotations from peer-reviewed literature, these ambiguities or controversies may propagate into secondary databases. To mitigate this limitation, the LLM was used strictly as an assistive tool, and all extracted information was subsequently reviewed and validated by human domain experts prior to inclusion in the database. Nevertheless, uncertainties inherent to the underlying literature remain an important limitation of literature-derived knowledge bases and are explicitly acknowledged here.

While vaccine adjuvants are able to enhance vaccine efficacy, their powerful immunostimulatory effects may also be accompanied by severe safety issues (Zhao et al., 2023). Some adjuvants can induce excessive inflammation or side effects, especially with local reactions being the most frequent manifestations or when administered systemically (Petrovsky, 2015). It is important to further investigate and understand the underlying safety issues associated with vaccine adjuvants, optimize delivery systems for individual adjuvants, and maximize their efficacy while maintaining an acceptable safety profile. By linking mechanisms, receptors, and immune outcomes, Vaxjo 2.0 provides a useful reference for weighing the desired immunogenicity of an adjuvant against possible safety concerns.

In future studies, several important directions warrant further exploration. We plan to continue expanding our LLM-based literature mining pipeline to enable broader identification of vaccine adjuvants and their immunological mechanisms. The Vaxjo dataset can also be used for different applications. For example, the Vaxjo data provide a foundation for machine learning and AI-based approaches for vaccine adjuvant prediction and rational design. Recently, we have developed a prototype of VaxjoGNN, a graph neural network approach that uses Vaxjo data and their ontological and graph-based representation for vaccine adjuvant recommendation (He and Zheng, 2025). The Vaxjo database provides a foundation for computational approaches to guide future discovery and rational prediction of vaccine adjuvants for vaccines against infectious diseases, cancer vaccines, or immune therapeutic applications (Elsheikh et al., 2023; Asfaw et al., 2024; Zheng et al., 2024).

## Data Availability

The original contributions presented in the study are included in the article/**Supplementary Material**. Further inquiries can be directed to the corresponding author.

## Supplementary material

Supplementary File 1 The master Excel file of all vaccine adjuvants in Vaxjo. Comprehensive table listing all 448 curated adjuvants, including the adjuvant source, Vaxjo record link, Vaxjo name, VO ID, VO label, adjuvant category, target receptor(s), and induced immune profile(s) (if available).

Supplementary File 2 LLM code. Complete code used to implement the two-phase LLM workflow for extracting and summarizing vaccine adjuvant mechanisms from PubMed abstracts. The file includes the full extraction module and the secondary summarization module used in Vaxjo 2.0.

Supplementary File 3 LLM prompt 1 and output 1 for getting adjuvant mechanisms. Contains the exact prompt used in Phase I to extract immune-response mechanism text for each adjuvant, together with representative raw model outputs before expert curation.

Supplementary File 4 LLM prompt 2 and output 2 file for summarizing the adjuvant mechanisms. Contains the summarization prompt used in Phase II to generate concise mechanism summaries and mechanism-family assignments, together with representative model outputs prior to expert review.

Supplementary File 5 Excel file containing subset of 50 adjuvants and their mechanism subtypes extracted via LLM. Adjuvants selected were the first 50 alphabetically. Each mechanism subtype was manually checked for accuracy and then tallied to get overall accuracy as a percentage.

Supplementary File 6 Adjuvant–mechanism binary association matrix. A comprehensive spreadsheet providing the full binary matrix of associations between all curated adjuvants and mechanism families. This file underlies **Figures 4** and **5** and enables direct inspection of all combinatorial overlaps across mechanisms for the complete adjuvant set.

Supplementary File 7 UpSet plot of higher-order mechanism overlaps across adjuvants. An UpSet plot visualizing higher-order intersections among mechanism families across all adjuvants. This figure complements **Figure 5** by systematically displaying complex multi-mechanism overlaps that are not easily captured in heatmap form.
