# Supplementary material for: Vaxjo 2.0: An ontology- and large language model-powered knowledge base of vaccine adjuvants and mechanisms
Source: Front Cell Infect Microbiol. 2026 Jun 5;16:1763384. doi: 10.3389/fcimb.2026.1763384 (PMC13279671; doi:10.3389/fcimb.2026.1763384)
Supplement: Supplementary File 2 — LLM code. Complete code used to implement the two-phase LLM workflow for extracting and summarizing vaccine adjuvant mechanisms from PubMed abstracts. The file includes the full extraction module and the secondary summarization module used in Vaxjo 2.0. [file Supplementaryfile2.pdf]

Supplementary File 2. LLM code. Complete code used to implement the two-phase LLM workf extracting and summarizing vaccine adjuvant mechanisms from PubMed abstracts. The file inclu full extraction module and the secondary summarization module used in Vaxjo 2.0

Phase I: Llama Inferencing

In [ ]:
```
import torch
from transformers import pipeline, AutoModelForCausalLM, AutoTokenizer
import os
os.environ["PYTORCH_CUDA_ALLOC_CONF"] = "expandable_segments:True"
```

In [ ]:
```
from huggingface_hub import login

# Replace 'your_token_here' with your actual Hugging Face token
from huggingface_hub import login
import os

token = os.getenv("HF_TOKEN")

if token is None:
    raise ValueError("HF_TOKEN environment variable not set.")

login(token=token)

print("Successfully logged in!")
```

In [ ]:
```
n_gpus = torch.cuda.device_count()
print("N GPUS: ", n_gpus)

# Set memory limits per GPU
model_vram_limit_mib = 8192  #12000
max_memory = f'{model_vram_limit_mib}MiB'
max_memory_dict = {i: max_memory for i in range(n_gpus)}

model_id = "meta-llama/Llama-3.2-3B-Instruct"

# Load model and tokenizer with memory limits
model = AutoModelForCausalLM.from_pretrained(
    model_id,
    device_map="auto",
    torch_dtype=torch.bfloat16,
    max_memory=max_memory_dict
)

tokenizer = AutoTokenizer.from_pretrained(model_id)

# Now create the pipeline using pre-loaded model/tokenizer
pipe = pipeline(
    "text-generation",
    model=model,
    tokenizer=tokenizer
)
```

In [ ]:
```
# Custom system prompt for extracting adjuvants and immune mechanisms
system_prompt = (
        """You are a biomedical assistant with expertise in immunology. E
```

```
For each adjuvant, extract the following:
- "adjuvant": The name of the adjuvant (e.g., Alum, MPLA, QS-21)
- "immune_response_mechanism": A brief description of how the adjuvant wo

Guidelines:
- Include only substances that are explicitly described as adjuvants in t
- Do not include delivery systems (e.g., liposomes, virosomes, VLPs) unle
- Do not infer or guess mechanisms that are not mentioned; leave them emp
- If no adjuvants are found, say 'No adjuvants mentioned.
- Return valid JSON in the following format:


[
  {
    "adjuvant": "Alum",
    "immune_response_mechanism": "Activates NLRP3 inflammasome and forms
  },
  {
    "adjuvant": "MPLA",
    "immune_response_mechanism": "Engages TLR4 pathway to promote Th1 res
  }
]"""
    )
```

In [ ]:
```
import json
import os
import csv
import torch
import gc
from datetime import datetime

def extract_and_save_adjuvant_response_text_from_string(
    pmc_id: str,
    text: str,
    system_prompt: str,
    pipe,
    output_file: str,
    max_new_tokens: int = 512
):
    """
    Processes a single abstract string and saves the response with PMID t
    """
    messages = [
        {"role": "system", "content": system_prompt},
        {"role": "user", "content": text},
    ]
    outputs = pipe(messages, max_new_tokens=max_new_tokens)
    response_text = outputs[0]["generated_text"][-1]["content"]
    with open(output_file, "a", encoding="utf-8") as out_f:
        out_f.write(f"=== {pmc_id} ===\n")
        out_f.write(response_text.strip() + "\n\n")
    print(f"Saved response for {pmc_id} to {output_file}")

def process_all_papers(
    input_json_file: str,
    system_prompt: str,
    pipe,
    output_txt_file: str,
    log_csv_file: str,
```

```python
    max_new_tokens: int = 512
):
    """
    Processes all abstracts from a JSON file and logs each result (succes
    Args:
        input_json_file (str): Path to input JSON file with {PMID: {title
        system_prompt (str): System prompt for the LLM.
        pipe: Hugging Face pipeline object.
        output_txt_file (str): Path to save model responses (plain text).
        log_csv_file (str): Path to save logs (CSV).
        max_new_tokens (int): Generation token limit.
    """
    # Read all abstracts into a dict
    with open(input_json_file, encoding="utf-8") as f:
        papers_dict = json.load(f)

    print(f"Found {len(papers_dict)} papers. Starting extraction...\n")

    # Prepare log CSV (write headers if file doesn't exist)
    log_exists = os.path.exists(log_csv_file)
    with open(log_csv_file, "a", newline='', encoding="utf-8") as log_f:
        log_writer = csv.writer(log_f)
        if not log_exists:
            log_writer.writerow(["PMID", "Status", "Message", "Timestamp"

        for i, (pmcid, paper_data) in enumerate(papers_dict.items(), 1):
            abstract_text = paper_data.get("abstract", "") or ""
            status = "success"
            message = "Processed successfully"
            try:
                extract_and_save_adjuvant_response_text_from_string(
                    pmcid, abstract_text, system_prompt, pipe, output_txt
                )
            except Exception as e:
                status = "error"
                message = str(e)[:500]
                print(f"� Error processing PMID {pmcid}: {message}")
            else:
                print(f"� [{i}/{len(papers_dict)}] Processed PMID {pmcid}
            finally:
                log_writer.writerow([pmcid, status, message, datetime.now
                log_f.flush()
                torch.cuda.empty_cache()
                gc.collect()
```

In [ ]:

```python
import os
import gc
import csv
import torch
from datetime import datetime

input_json_file = "Dataset/Vaxjo/All PMID abstracts.txt"  # should contai
output_txt_file = "Outputs/Vaxjo-LLM-Phase I-Response.txt"
log_csv_file = "Outputs/Vaxjo_PMIDs_adjuvant_log.csv"

process_all_papers(
    input_json_file,
    system_prompt,
    pipe,
    output_txt_file,
```

```
    log_csv_file,
    max_new_tokens=512
)
print("Phase I LLM inferencing complete")
```

Phase I - Processing

In [ ]:
```
import re
import json
import pandas as pd

# Load the content from the text file
with open("Outputs/Vaxjo-LLM-Phase I-Response.txt", "r", encoding="utf-8"
    content = file.read()

# Extract each PMID block
entries = re.split(r'===\s+(\d+)\s+===', content)[1:]
pmid_ids = entries[::2]   # PMIDs
texts = entries[1::2]    # Corresponding text blocks

# Extract data
data = []
for pmid_id, text in zip(pmid_ids, texts):
    if "No adjuvants mentioned." in text:
        continue
    # Find JSON-like blocks
    json_blocks = re.findall(r"\[\s*{.*?}\s*]", text, re.DOTALL)
    for block in json_blocks:
        try:
            adjuvant_list = json.loads(block)
            for item in adjuvant_list:
                data.append({
                    "PMID": pmid_id,
                    "adjuvant": item.get("adjuvant"),
                    "immune_response_mechanism": item.get("immune_respons
                })
        except json.JSONDecodeError:
            continue

# Save to CSV
df = pd.DataFrame(data)
df.to_csv("Outputs/Vaxjo_PMIDs_adjuvant_extracted.csv", index=False)

print("Extraction complete. Saved to 'Vaxjo_PMIDs_adjuvant_extracted.csv'
df
```

In [ ]:
```
# Count unique adjuvants
unique_adjuvants = df["adjuvant"].nunique()
print(f"Number of unique adjuvants: {unique_adjuvants}")
```

In [ ]:
```
# Find unique immune response mechanisms containing the word "not" (case-
mechanisms = df["immune_response_mechanism"].dropna().unique()

mechanisms_with_not_none = [
    m for m in mechanisms if ("not " in m.lower() or "none " in m.lower()
]
```

```python
for mechanism in mechanisms_with_not_none:
    print(mechanism)
```

In [ ]:
```python
not_valid_keywords = [
    "None mentioned",
"Not explicitly described",
"None mentioned.",
"Not specified",
"None described",
"Not explicitly described in the text",
"Mechanism of action not yet completely known, but it is comprised of Qui
"None specified",
"Not mentioned",
"Mechanism not explicitly described",
"Not explicitly stated, but based on the context, it appears to stimulate
"Not specified in the text.",
"Not explicitly described, but it is known that aluminum salts activate N
"Not explicitly described, but it is known that saponins like QS21 can st
"Not described",
"Mechanism not described",
"Not explicitly described, left blank."
]


# Drop rows with NaN or empty strings (after stripping whitespace)
df_cleaned = df[df["immune_response_mechanism"].notna()].copy()
df_cleaned = df_cleaned[df_cleaned["immune_response_mechanism"].str.strip

# Remove rows containing any invalid keyword (case-insensitive)
df_cleaned = df_cleaned[
    ~df_cleaned["immune_response_mechanism"].str.lower().apply(
        lambda x: any(keyword in x for keyword in not_valid_keywords)
    )
].reset_index(drop=True)

# Save to CSV
df_cleaned.to_csv("Outputs/Vaxjo_PMIDs_adjuvant_extracted_clean.csv", ind

# Preview the result
df_cleaned
```

In [ ]:
```python
# Get and print the sorted list of unique adjuvant names
unique_adjuvant_names = sorted(df_cleaned["adjuvant"].unique())
print("Unique adjuvants:")
for name in unique_adjuvant_names:
    print(name)
```

In [ ]:
```python
mapping = {
    # ---- Adjuplex / ADJ ----
    "ADJ": "Adjuplex (ADJ)",

    # ---- Alum / Aluminum hydroxide family (exact naming variants only)
    "Alum": "Alum (aluminum hydroxide)",
    "Al(OH)3": "Alum (aluminum hydroxide)",
    "Alhydrogel": "Alum (aluminum hydroxide)", #commercial brand name for
    "Aluminium Hydroxide (AH)": "Alum (aluminum hydroxide)",
    "Aluminium hydroxide": "Alum (aluminum hydroxide)",
    "Aluminum Hydroxide": "Alum (aluminum hydroxide)",
    "Aluminum Hydroxide (AH)": "Alum (aluminum hydroxide)",
```

```
    "Aluminum hydroxide": "Alum (aluminum hydroxide)",
    "Aluminum hydroxide (AH)": "Alum (aluminum hydroxide)",
    "Aluminum hydroxide (Alum)": "Alum (aluminum hydroxide)",
    "Aluminum oxyhydroxide": "Alum (aluminum hydroxide)", #Aluminum oxyhy
    # ---- Advax / delta inulin (brand vs generic, diacritics, ™/®) ----
    "Advax": "Advax (delta inulin)",
    "Advax delta inulin": "Advax (delta inulin)",
    "Advax® delta inulin": "Advax (delta inulin)",
    "Advax™": "Advax (delta inulin)",
    "Delta Inulin (DI)": "Advax (delta inulin)",
    "Delta inulin": "Advax (delta inulin)",
    "delta inulin": "Advax (delta inulin)",
    "delta inulin (DI)": "Advax (delta inulin)",
    "delta inulin adjuvant (Advax™)": "Advax (delta inulin)",
    "δ-inulin": "Advax (delta inulin)",
    "delta-inulin": "Advax (delta inulin)",

    # Keep specific Advax *formulations* distinct:
    # Advax-2 / -M / -P / -SM / CpG / CpG55.2 etc. are NOT collapsed.

    # ---- BECC spelling/spacing ----
    "BECC 438": "BECC438",
    "BECC 470": "BECC470",

    # ---- Calcium phosphate shorthand ----
    "CAP": "Calcium Phosphate (CAP)",

    # ---- CAF01 encoding variant ----
    "CAF\u00b01": "CAF01",

    # ---- Cholera toxin naming ----
    "Cholera toxin": "Cholera toxin (CT)",

    # ---- Compound 48/80 naming ----
    "C48/80": "Compound 48/80 (C48/80)",

    # ---- CoVaccine HT trademark ----
    "CoVaccine HT™": "CoVaccine HT",

    # ---- CpG ODN (shorthands & plurals used as the same thing here) ---
    "CpG": "CpG ODN",
    "CpG ODNs": "CpG ODN",
    #"CpG motifs": "CpG ODN",
    "CpG oligodeoxynucleotide (CpG-ODN)": "CpG ODN",
    "CpG-ODN": "CpG ODN",

    # Keep specific sequences distinct (not collapsed):
    # "CpG ODN 1826", "CpG M362", "ODN2006" remain separate.


    "Complete Freund's adjuvant": "Complete Freund's Adjuvant (CFA)",

    # ---- EM-005 aka GLA-SE ----
    "EM-005 (GLA-SE)": "GLA-SE",

    # ---- Flagellin (case only) ----
    "flagellin": "Flagellin",

    # (FliC, FlaB kept distinct—specific flagellins.)
```

```
    # ---- GLA naming variants ----
    "Glucopyranosyl Lipid Adjuvant": "GLA",
    "Glucopyranosyl Lipid Adjuvant (GLA)": "GLA",
    "Glucopyranosyl lipid A": "GLA",
    #"Glucopyranosyl lipid A (G100)": "GLA",
    "Glucopyranosyl lipid adjuvant (GLA)": "GLA",
    "ID93/glucopyranosyl lipid adjuvant (GLA)": "GLA",
    # GLA-AF / GLA-SE / GLA-LSQ are formulations—kept distinct.

    "GLA-SE (components included: glucopyranosyl lipid A, squalene emulsi
    "GLA-squalene emulsion": "GLA-SE",
    "Glucopyranosyl lipid A (G100)": "GLA",

    # ---- IFN naming (alpha/beta symbol) ----
    "IFN-alpha": "IFN-α",
    "IFNβ": "IFN-β",

    # ---- Imiquimod alias ----
    "imiquimod (R837)": "Imiquimod (R837)",
    "Imiquimod": "Imiquimod (R837)",

    # ---- Matrix-M trademark ----
    "Matrix-M™": "Matrix-M",

    # ---- MCT trademark ----
    "MCT®": "MCT",

    # ---- MPL naming variants ----
    '''"MPL": "MPL (monophosphoryl lipid A)",
    "MPL®": "MPL (monophosphoryl lipid A)",
    "Monophosphoryl lipid": "MPL (monophosphoryl lipid A)",
    "Monophosphoryl lipid A (MPLA)": "MPLA",'''

    # Keep "MPLA" as is (canonical already).
    "MPL": "MPL (monophosphoryl lipid A)",
    "MPL®": "MPL (monophosphoryl lipid A)",
    "Monophosphoryl lipid": "MPL (monophosphoryl lipid A)",
    "Monophosphoryl lipid A (MPLA)": "MPL (monophosphoryl lipid A)",

    "MPL (monophosphoryl lipid A)": "MPLA",
    "MPL": "MPLA",   # redundant safety check, in case not yet mapped

    # "MPLA" stays as is (already canonical).



    "M7": "Mastoparan-7 (M7)",


    # Keep MPL-SE / MPL+TDM / MPL/DDA distinct (formulations/combinations

    # ---- Montanide ISA 51 spacing ----
    "Montanide ISA-51": "Montanide ISA 51",
    "Montanide ISA51": "Montanide ISA 51",

    # --- Mastoparan naming ---
    "Mastoparan 7": "Mastoparan-7 (M7)",

    # ---- PCL abbreviation expansion ----
    "PCL/chitosan NPs": "poly-ϵ-caprolactone/chitosan NPs",
```

```
    # ---- Poly(I:C) case ----
    "poly(I:C)": "Poly(I:C)",

    # ---- Polyclonal Antibody Stimulator naming ----
    "polyclonal antibody stimulator-PAS": "Polyclonal Antibody Stimulator

    # ---- QS-21 dash/spacing ----
    "QS21": "QS-21",

    # ---- R848 (resiquimod) alias ----
    "R848": "Resiquimod (R848)",
    "resiquimod": "Resiquimod (R848)",

    # ---- SE (squalene emulsion) naming (keep brands separate like MF59/
    "Squalene-based oil-in-water emulsion system (SE)": "SE",
    "squalene oil-in-water emulsion (SE)": "SE",

    # ---- SWE (Sepivac SWE) naming ----
    "Sepivac SWE": "SWE",
    "SEPIVAC SWETM": "SWE",

    # ---- Squalene case only ----
    "squalene": "Squalene",

    # ---- TDM (trehalose-6,6'-dimycolate) spelling variants ----
    "6,6'-trehalose dimycolate (TDM)": "Trehalose-6,6'-dimycolate (TDM)",

    # ---- α-GalCer naming variants ----
    "α-Galactosylceramide (α-GC)": "α-GalCer",
    "α-Galactosylceramide (αGalCer)": "α-GalCer",
}
df_cleaned["adjuvant_canonical"] = df_cleaned["adjuvant"].replace(mapping
df_cleaned
```

In [ ]:
```
print("Before:", df_cleaned["adjuvant"].nunique())
print("After :", df_cleaned["adjuvant_canonical"].nunique())
```

In [ ]:
```
# Get and print the sorted list of unique adjuvant names
unique_adjuvant_names_canonical = sorted(df_cleaned["adjuvant_canonical"]
print("Unique adjuvants_canonical:")
for name in unique_adjuvant_names_canonical:
    print(name)
```

In [ ]:
```
df_grouped = (
    df_cleaned
    .assign(immune_response_mechanism=lambda x: x["immune_response_mechan
    .groupby("adjuvant_canonical")["immune_response_mechanism"]
    .agg(lambda x: " | ".join(sorted(set(x))))
    .reset_index()
    .sort_values(by="adjuvant_canonical", key=lambda s: s.str.lower())
)

df_grouped.to_csv("Outputs/Vaxjo_PMIDs_adjuvant_mechanism_collapsed.csv",
df_grouped
```

Phase II: Llama Inferencing

In [ ]:
```python
import pandas as pd

system_prompt = """You are an expert immunologist and biomedical research
TASK: Analyze the provided text on a vaccine adjuvant's immune response.

## Instructions for the "summary" field:
- **Synthesize the information into a cohesive, mechanistic narrative of
- This summary should not be a simple list of facts. Instead, it should d
- For example, describe how the adjuvant is initially sensed (e.g., by PR
- Integrate the corresponding PMIDs directly into the text immediately fo

## Instructions for the "mechanism_subtypes" field:
- Identify **every distinct** immunological mechanism.
- For each identified subtype, list all unique PMIDs cited as evidence fo
- Do not merge related subtypes; for example, if both "dendritic cell" an

## General Rules:
- **Strict JSON Output:** The entire response MUST be a single, valid JSO
- **Source Adherence:** Use ONLY the information and PMIDs present in the

## JSON Schema:
{
  "adjuvant": "<string>",
  "summary": "<A cohesive, mechanistic narrative of 3-5 sentences describ
  "mechanism_subtypes": [
    {
      "mechanism subtype": "<mechanism subtype_1>",
      "evidence_refs": ["########", "..."]
    },
    {
      "mechanism subtype": "<mechanism subtype_2>",
      "evidence_refs": ["########", "..."]
    },...
  ]
}

"""

# Load one row from your collapsed CSV
df = pd.read_csv("Outputs/Vaxjo_PMIDs_adjuvant_mechanism_collapsed.csv")
# Rename the column
df = df.rename(columns={"adjuvant_canonical": "adjuvant"})
df
```

In [ ]:
```python
# Iterate over the whole DataFrame, run generation, and save raw outputs
# Assumes you already have: df, system_prompt, and pipe(...) defined.

import json
import os

OUT_TXT = "Outputs/Vaxjo-LLM-Phase II-Response.txt"   # plain text (human
# (optional) also keep a machine-friendly JSONL:
OUT_JSONL = "Outputs/Vaxjo-LLM-Phase II-Response.jsonl"

os.makedirs(os.path.dirname(OUT_TXT) or ".", exist_ok=True)

# If you want to change token budget, tweak here:
GEN_MAX_NEW_TOKENS = 4028
```

```
# Open files once and append per row (flush to avoid losing progress mid-
with open(OUT_TXT, "w", encoding="utf-8") as f_txt, open(OUT_JSONL, "w",
    for idx, row in df.iterrows():
        adjuvant = str(row.get("adjuvant", ""))
        mechanism = str(row.get("immune_response_mechanism", ""))

        messages = [
            {"role": "system", "content": system_prompt},
            {"role": "user", "content": f"Adjuvant: {adjuvant}\nImmune re
        ]

        status = "ok"
        try:
            outputs = pipe(messages, max_new_tokens=GEN_MAX_NEW_TOKENS)
            raw = outputs[0]["generated_text"][-1]["content"]
        except Exception as e:
            status = "error"
            raw = f"__ERROR__: {e}"

        # ---- Write human-readable TXT ----
        header = f"===== ROW {idx} | {adjuvant} | {status} =====\n"
        f_txt.write(header)
        f_txt.write((raw or "").strip() + "\n\n")
        f_txt.flush()

        # ---- (Optional) also write JSONL per row ----
        f_jsonl.write(json.dumps({
            "row_index": int(idx),
            "adjuvant": adjuvant,
            "status": status,
            "raw": raw
        }, ensure_ascii=False) + "\n")
        f_jsonl.flush()

print(f"Saved outputs to:\n- {OUT_TXT}\n- {OUT_JSONL} (optional JSONL)")
```

Phase II - Processing

In [ ]:

```
import os
import re
import json
import pandas as pd
import matplotlib.pyplot as plt
import seaborn as sns
from collections import defaultdict, Counter

INPUT_FILE = "Outputs/Vaxjo-LLM-Phase II-Response.txt"
OUTDIR = "Outputs/final_adjuvant_mechanism_analysis/"
os.makedirs(OUTDIR, exist_ok=True)

print(f"Libraries imported and output directory set to: {OUTDIR}")
```

In [ ]:

```
def normalize_text(s: str) -> str:
    s = str(s).strip()
    s = re.sub(r"\bresponses\b", "response", s, flags=re.I)
    s = re.sub(r"\bactivations\b", "activation", s, flags=re.I)
    s = re.sub(r"\bcells\b", "cell", s, flags=re.I)
    s = re.sub(r"\bcytokines\b", "cytokine", s, flags=re.I)
    s = re.sub(r"\bantibodies\b", "antibody", s, flags=re.I)
```

```python
    s = re.sub(r"\bpathways\b", "pathway", s, flags=re.I)
    s = re.sub(r"\bmechanisms\b", "mechanism", s, flags=re.I)
    s = re.sub(r"\s+", " ", s)
    s = s.replace("–", "-").replace("—", "-")
    # Remove quotes
    s = s.replace('"', "").replace("'", "")
    return s.strip()

CANONICAL_REPLACEMENTS = [
    (r"\bT[- ]?cell\b", "T cell"),
    (r"\bTh[- ]?1\b", "Th1"),
    (r"\bTh[- ]?2\b", "Th2"),
    (r"\bTh[- ]?17\b", "Th17"),
    (r"\bIFN ?- ?γ\b", "IFN-γ"),
    (r"\bNF.?κB\b", "NF-κB"),
    (r"\bB[- ]?cell\b", "B cell"),
]

def canonicalize(s: str) -> str:
    s = normalize_text(s)
    for pat, repl in CANONICAL_REPLACEMENTS:
        s = re.sub(pat, repl, s, flags=re.I)
    # Force to lowercase for consistent grouping
    return s.lower()

def restore_acronyms(s: str) -> str:
    s = re.sub(r"\btlr\b", "TLR", s, flags=re.I)
    s = re.sub(r"\bdc\b", "DC", s, flags=re.I)
    s = re.sub(r"\bnlrp3\b", "NLRP3", s, flags=re.I)
    s = re.sub(r"\bifn\b", "IFN", s, flags=re.I)
    s = re.sub(r"\bmhc\b", "MHC", s, flags=re.I)
    s = re.sub(r"\bmyd88\b", "MyD88", s, flags=re.I)
    s = re.sub(r"\btrif\b", "TRIF", s, flags=re.I)
    s = re.sub(r"\bsting\b", "STING", s, flags=re.I)
    return s

# [MODIFIED] Map replacement values are now clean and simple
ADJUVANT_NORM_MAP = [
    # Pattern (regex, case-insensitive)  ->  Canonical Name
    # Alum Group
    (r"alum",                                "Alum"),
    (r"aluminium",                           "Alum"),
    (r"aluminum",                            "Alum"),
    # Heat-Labile Toxin Group
    (r"\blt\b",                              "LT"),
    (r"heat-labile toxin",                   "LT"),
    # Freund's Adjuvant
    (r"freund",                              "Freund's Adjuvant"),
    (r"cationic.*liposome",                  "Cationic Liposome"),

]

def normalize_adjuvant(s: str) -> str:
    s = str(s).strip()

    # [MODIFIED] Remove anything in parentheses (e.g., descriptions)
    s = re.sub(r"\(.*?\)", "", s).strip()

    s_lower = s.lower()
```

```
    for pat, repl in ADJUVANT_NORM_MAP:
        if re.search(pat, s_lower, flags=re.I):
            return repl

    # If no rule matches, return the new parenthetical-stripped string
    return s

print("All helper functions defined (v4 with corrected map).")
```

In [ ]:
```
FAMILY_TO_SUBBRANCH = {
    # --- 1. SPECIFIC SIGNALING PATHWAYS ---
    "NLRP3 inflammasome activation": {
        "NLRP3 core branch": [r"\bNLRP3\b"],
        "MAPK/JNK pathway": [r"\bMAPK\b", r"\bJNK\b"],
        "Caspase / pyroptosis": [r"caspase", r"pyroptosis"],
        "Other inflammasome activity": [r"inflammasome"],
    },
    "STING / TRIF / MyD88 / RIG-I signaling": {
        "STING": [r"\bSTING\b"],
        "TRIF": [r"\bTRIF\b"],
        "MyD88": [r"\bMyD88\b"],
        "RIG-I-like": [r"\bRIG"],
        "NOD-like": [r"\bNOD"],
        "Other signaling adaptors": [r"adaptor", r"signaling"],
    },
    "TLR signaling": {
        "TLR2 branch": [r"\bTLR2\b"],
        "TLR3 branch": [r"\bTLR3\b"],
        "TLR4 branch": [r"\bTLR4\b"],
        "TLR5 branch": [r"\bTLR5\b"],
        "TLR7/8 branch": [r"\bTLR7\b", r"\bTLR8\b"],
        "TLR9 branch": [r"\bTLR9\b"],
        "MyD88/TRIF-related": [r"MyD88", r"TRIF"],
        "Other TLR-related": [r"toll-?like receptor", r"\bTLR\b", r"lipid
    },
    "Pattern recognition / PRR sensing": {
        "PRR family": [r"\bprr(s)?\b"],
        "Pattern recognition": [r"pattern recognition"],
        "C-type lectin receptors": [r"Dectin", r"Mincle", r"\bMCL\b", r"d
        "Other pattern sensors": [r"recognition", r"sensing", r"sensors"]
    },

    # --- 2. SPECIFIC CELLULAR RESPONSES ---
    "T cell activation / polarization": {
        "T cell branch": [r"T cell", r"T-cell", r"T lymphocyte", r"\bctl\
        "Th1 branch": [r"Th1"],
        "Th2 branch": [r"Th2"],
        "Th17 branch": [r"Th17"],
        "CD4/CD8 branch": [r"CD4", r"CD8"],
        "Tfh branch": [r"Tfh"],
        "Regulatory T cell branch": [r"Treg", r"regulatory T"],
    },
    "Dendritic cell activation": {
        "DC maturation": [r"maturation"],
        "DC polarization": [r"polarization"],
        "Plasmacytoid DC": [r"plasmacytoid"],
        "Antigen presentation-related DC": [r"antigen", r"\bAPC\b", r"pre
        "Other DC activation": [r"dendritic"],
    },
    "B cell / antibody production": {
```

```
        "B cell activation": [r"\bB cell\b", r"\bB-cell\b"],
        "Antibody production": [r"antibody", r"\bIgG\b", r"\bIgA\b", r"\b
        "Humoral immunity": [r"humoral"],
        "Plasma cell / differentiation": [r"\bplasma\b", r"plasmablast",
        "Germinal center / memory": [r"germinal", r"memory"],
        "Other B cell mechanisms": [r"\bB\b"],
    },
    "Macrophage / innate immune activation": {
        "Macrophage": [r"macrophage"],
        # [MODIFIED] Added 'cd56' (NK cell marker)
        "NK / Monocyte": [r"\bNK\b", r"monocyte", r"cd56"],
        "Innate immune cells": [r"innate"],
        "Neutrophils / Granulocytes": [r"neutrophil", r"granulocyte"],
        "Other innate activation": [r"activation"],
    },

    # --- 3. FUNCTIONAL / PROCESS-BASED ---
    "Antigen presentation / APCs": {
        "APC activation": [r"activation", r"\bAPC\b"],
        "Cross-presentation": [r"cross-?presentation", r"\bcross\b"],
        "MHC / Co-stimulation": [r"\bMHC\b", r"\bCD40\b", r"\bCD80\b", r"
        "Migration / trafficking": [r"migration", r"traffick", r"recruitm
        "Antigen processing / uptake": [r"antigen", r"uptake", r"processi
        "Other APC function": [r"presentation"],
    },
    "Cytokine signaling / production": {
        "Interleukins": [r"\bIL[- ]?\d", r"interleukin"],
        "Interferons": [r"\bIFN", r"interferon"],
        "TNF": [r"\bTNF"],
        "Chemokines": [r"chemokine", r"\bCCL", r"\bCXCL"],
        "Other cytokines": [r"cytokine"],
    },
    "Inflammatory response": {
        "Pro-inflammatory genes": [r"inflamm", r"NF[- ]?κB", r"NF[- ]?kB"
        "Cytokine-mediated inflammation": [r"cytokine"],
        "Chemokine signaling": [r"chemokine", r"\bCCL", r"\bCXCL"],
        "Immune suppression / regulation": [r"regulation", r"inhibition"]
        "Other": [r"response", r"activation", r"nitric oxide", r"oxidativ
    },

    # --- 4. OTHER / HIGH-LEVEL ---
    "Complement / depot / formulation": {
        "Complement activation": [r"complement"],
        "Depot / release mechanisms": [r"depot", r"release"],
        "Adjuvant formulation / emulsions": [r"\balum\b", r"emulsion", r"
        "Other": [r"activation"],
    },
    "Adjuvant synergy / immune modulation": {
        "Immune enhancement": [r"enhanc", r"promotion"],
        "Costimulation": [r"co-?stimul", r"\bCD40\b", r"\bCD86\b"],
        "Immune modulation": [r"modulat"],
        "Synergy": [r"synerg", r"combination", r"co-?activation"],
        "Other": [r"activation", r"retinoic acid", r"immunogenicity"],
    },
    "Mucosal immunity": {
        "Mucosal keywords": [r"mucosal", r"homing"],
    },
    "Apoptosis / Cell Death Induction": {
        "Apoptosis keywords": [r"apoptosis", r"cell death"],
    },
```

```
}

print(f"Classification map 'FAMILY_TO_SUBBRANCH' defined (v5) with {len(F
```

In [ ]:

```
records = []
raw_adjuvants = [] # Store raw adjuvant names
raw_subtypes = []  # Store raw subtype names
content = ""

try:
    with open(INPUT_FILE, "r", encoding="utf-8") as f:
        content = f.read()
except FileNotFoundError:
    print(f"□ ERROR: Input file not found: {INPUT_FILE}")
    print("Please make sure the file is in the same directory, or provide
    # Stop execution if file not found
    raise
except Exception as e:
    print(f"□ ERROR: An unexpected error occurred while reading {INPUT_FI
    # Stop execution if other read error
    raise

if not content:
    print("□ ERROR: File was read but is empty. Cannot proceed.")
else:
    # Split the file by the '===== ROW ...' headers
    chunks = re.split(r"===== ROW \d+ \| .*? \| ok =====", content)

    for i, chunk in enumerate(chunks):
        if not chunk.strip():
            continue

        try:
            # Clean up the chunk to get raw JSON
            json_text = chunk.strip().strip("`").replace("json", "").stri

            if not json_text.startswith("{"):
                print(f"⚠ Skipping chunk {i}: No valid JSON object found
                continue

            inner = json.loads(json_text)

            # --- Adjuvant Normalization ---
            adjuvant_raw = inner.get("adjuvant", "Unknown").strip()
            raw_adjuvants.append(adjuvant_raw) # Store raw
            adjuvant = normalize_adjuvant(adjuvant_raw) # Get normalized
            #adjuvant = adjuvant_raw # Do not normalize


            for item in inner.get("mechanism_subtypes", []):
                # --- Subtype Normalization ---
                subtype_raw = item.get("mechanism_subtype", "")
                raw_subtypes.append(subtype_raw) # Store raw
                subtype = canonicalize(subtype_raw) # Get normalized

                if subtype:
                    records.append({"Adjuvant": adjuvant, "Subtype": subt

        except Exception as e:
            print(f"⚠ Skipping chunk {i}: Failed to parse JSON. Error: {e
```

```python
    # Create the master DataFrame
    df = pd.DataFrame(records)

    if df.empty:
        print("□ ERROR: No records were successfully parsed. DataFrame is
        print("Please check the INPUT_FILE format.")
    else:
        print(f"□ Loaded {len(df)} total adjuvant–subtype pairs.")

        print("\n--- Adjuvant Normalization Stats ---")
        print(f"   Unique adjuvants BEFORE normalization: {len(set(raw_ad
        print(f"   Unique adjuvants AFTER normalization:  {df['Adjuvant']

        print("\n--- Subtype Normalization Stats ---")
        print(f"   Unique subtypes BEFORE normalization: {len(set(raw_sub
        print(f"   Unique subtypes AFTER normalization:  {df['Subtype'].n

        # Display the first 5 rows
        print("\n--- DataFrame Head ---")
        print(df.head())
```

In [ ]:

```python
def map_to_family(sub):
    """
    Iterates through the FAMILY_TO_SUBBRANCH map (from Cell 3)
    and returns the first family that matches the subtype.
    """
    for fam, submap in FAMILY_TO_SUBBRANCH.items():
        for kws in submap.values():
            if any(re.search(kw, sub, flags=re.I) for kw in kws):
                return fam
    # If no match is found after checking all families
    return "Other / Unclassified"

# Apply the function to the 'Subtype' column to create the new 'Family' c
df["Family"] = df["Subtype"].apply(map_to_family)

print("□ 'Family' column created successfully.")

# Display the head to show the new column
print("\n--- DataFrame Head with 'Family' Column ---")
print(df.head())

# Show the distribution of classified families
print("\n--- Family Distribution (Top 15) ---")
print(df["Family"].value_counts().head(15))
```

In [ ]:

```python
try:
    # 1. Get unique raw adjuvants (from Cell 4)
    unique_raw_adjuvants = sorted(list(set(raw_adjuvants)))
    df_raw_adj = pd.DataFrame(unique_raw_adjuvants, columns=["Raw_Adjuvan
    raw_adj_path = os.path.join(OUTDIR, "review_unique_adjuvants_raw.csv"
    df_raw_adj.to_csv(raw_adj_path, index=False)
    print(f"Saved {len(df_raw_adj)} unique raw adjuvant names to: {raw_ad

    # 2. Get unique normalized adjuvants (from Cell 4's df)
    unique_norm_adjuvants = sorted(list(df["Adjuvant"].unique()))
    df_norm_adj = pd.DataFrame(unique_norm_adjuvants, columns=["Normalize
    norm_adj_path = os.path.join(OUTDIR, "review_unique_adjuvants_normali
```

```
    df_norm_adj.to_csv(norm_adj_path, index=False)
    print(f"Saved {len(df_norm_adj)} unique normalized adjuvant names to:

    # 3. Get unique raw subtypes (from Cell 4)
    unique_raw_subtypes = sorted(list(set(raw_subtypes)))
    df_raw_sub = pd.DataFrame(unique_raw_subtypes, columns=["Raw_Subtype_
    raw_sub_path = os.path.join(OUTDIR, "review_unique_subtypes_raw.csv")
    df_raw_sub.to_csv(raw_sub_path, index=False)
    print(f"Saved {len(df_raw_sub)} unique raw subtype names to: {raw_sub

    # 4. Get unique normalized subtypes (from Cell 4's df)
    unique_norm_subtypes = sorted(list(df["Subtype"].unique()))
    df_norm_sub = pd.DataFrame(unique_norm_subtypes, columns=["Normalized
    norm_sub_path = os.path.join(OUTDIR, "review_unique_subtypes_normaliz
    df_norm_sub.to_csv(norm_sub_path, index=False)
    print(f"Saved {len(df_norm_sub)} unique normalized subtype names to:

    print("\nAll review files saved.")

except NameError:
    print("□ ERROR: Could not find variables 'raw_adjuvants', 'raw_subtyp
    print("Please make sure you have successfully run Cell 4 and Cell 5."
```

In [ ]:

```
try:
    # 1. Create a binary DataFrame
    # This drops all duplicate Adjuvant/Family pairs.
    df_binary = df.drop_duplicates(subset=["Adjuvant", "Family"])
    print(f"□ 'df_binary' created. Dropped {len(df) - len(df_binary)} dup

    # 2. Create the main pivot table (Adjuvant x Family -> Count)
    # This will now only have 0s and 1s
    pivot = (
        df_binary.groupby(["Adjuvant", "Family"])
        .size()
        .reset_index(name="Count")
    )

    print("□ Binary 'pivot' table created successfully.")

    # 3. Create the family counts for the bar/pie chart
    # This now counts the number of ADJUVANTS, not mentions
    family_counts = (
        pivot.groupby("Family")["Count"]
        .sum()
        .sort_values(ascending=False)
        .reset_index()
    )
    family_counts.columns = ["Family", "Total_Adjuvants"] # Renamed colum

    print("□ Binary 'family_counts' created successfully.")
    print("\n--- Binary Family Counts Head (Counts Adjuvants, not Mention
    print(family_counts.head())

    # 4. Save the main pivot table to a CSV
    csv_path = os.path.join(OUTDIR, "adjuvant_family_summary_BINARY.csv")
    pivot.to_csv(csv_path, index=False)
    print(f"\n□ Binary Summary CSV saved → {csv_path}")

except NameError:
    print("□ ERROR: Could not find DataFrame 'df'.")
```

```
    print("Please make sure you have successfully run Cell 4 and Cell 5."
except Exception as e:
    print(f"□ ERROR: An unexpected error occurred: {e}")
```

In [ ]:

```
try:
    # 1. Filter the DataFrame for "Other / Unclassified"
    df_other = df[df['Family'] == 'Other / Unclassified']

    # 2. Get the unique subtypes
    unique_other_count = df_other['Subtype'].nunique()
    unique_other_list = sorted(list(df_other['Subtype'].unique()))

    print(f"□ Found {unique_other_count} unique subtypes classified as 'O
    print(f"   (These {unique_other_count} subtypes appeared a total of {

    # 3. Print the list
    print("--- List of 'Other / Unclassified' Subtypes ---")
    for subtype in unique_other_list:
        print(subtype)

    # 4. Save the list to a new CSV for review
    df_review_other = pd.DataFrame(unique_other_list, columns=["Unclassif
    other_csv_path = os.path.join(OUTDIR, "review_unclassified_subtypes.c
    df_review_other.to_csv(other_csv_path, index=False)

    print("\n-------------------------------------------------")
    print(f"□ Saved this list for review → {other_csv_path}")

except NameError:
    print("□ ERROR: Could not find DataFrame 'df'.")
    print("Please make sure you have successfully run Cell 4 and Cell 5."
except Exception as e:
    print(f"□ ERROR: An unexpected error occurred: {e}")
```

In [ ]:

```
try:
    import plotly.graph_objects as go
    import plotly.io as pio
except ImportError:
    print("□ ERROR: Plotly is not installed. Please run 'pip install plot
    # You can skip this cell if you don't want the Plotly chart

try:
    # 1. --- Prepare the DataFrame for Plotly ---
    # We use 'family_counts' from Cell 7B
    df_plotly = family_counts.copy()

    total = df_plotly["Total_Adjuvants"].sum()
    df_plotly["percent"] = (df_plotly["Total_Adjuvants"] / total) * 100

    df_plotly["label_text"] = df_plotly.apply(
        lambda row: f"<b>{restore_acronyms(row['Family'].title())}</b><br
        axis=1
    )
    df_plotly["textposition"] = df_plotly["percent"].apply(lambda p: 'out

    print("--- Pie Chart Data (Counts Adjuvants) ---")
    print(df_plotly.to_string())
    print("\n")
```

```
    # 2. --- Build Plotly Pie Chart ---
    fig = go.Figure(
        data=[
            go.Pie(
                #labels=df_plotly["Family"].apply(lambda f: restore_acron
                labels=df_plotly["Family"].apply(restore_acronyms),
                values=df_plotly["Total_Adjuvants"],
                text=df_plotly["label_text"],
                textinfo="text",
                textposition=df_plotly["textposition"],
                textfont_size=14,
                outsidetextfont_size=14,
                insidetextorientation="radial",
                hovertemplate="<b>%{label}</b><br>%{value} Adjuvants<br>%
                marker=dict(line=dict(color="white", width=1.5)),
                pull=[0.03 if p < 5 else 0 for p in df_plotly["percent"]]
            )
        ]
    )

    # 3. --- Layout ---
    fig.update_layout(
        title=dict(
            text="Distribution of Adjuvants across Mechanism Families",
            x=0.5,
            font=dict(size=24)
        ),
        showlegend=False,
        width=1200,
        height=900,
        margin=dict(t=100, b=100, l=100, r=150),
    )

    # 4. --- Save as HTML file ---
    plotly_path = os.path.join(OUTDIR, "adjuvant_family_pie_interactive.h
    fig.write_html(plotly_path)

    # 5. --- Save as static image (requires 'kaleido') ---
    try:
        static_path = os.path.join(OUTDIR, "adjuvant_family_pie_static.pn
        pio.write_image(fig, static_path, scale=2)
        print(f"□ Static Plotly pie chart saved → {static_path}")
    except Exception as e:
        print(f"⚠ Could not save static image. (Requires 'kaleido'). Erro

    print(f"□ Interactive Plotly pie chart saved → {plotly_path}")

except NameError:
    print("□ ERROR: Could not find 'family_counts' DataFrame.")
    print("Please make sure you have successfully run Cell 7B.")
except Exception as e:
    print(f"□ ERROR: An unexpected error occurred while plotting: {e}")
```

In [ ]:

```
try    # 1. Find top adjuvants (by mechanistic diversity)
    # The .sum() counts the # of families, since 'Count' is always 1
    top_adjuvants = pivot.groupby("Adjuvant")["Count"].sum().nlargest(20)

    # 2. And the top families (by total # of adjuvants)
    top_families = family_counts["Family"].head(20)
```

```
    # 3. Ensure we only select columns that exist after pivoting
    valid_families = [f for f in top_families if f in pivot["Family"].uni
    valid_adjuvants = [a for a in top_adjuvants if a in pivot["Adjuvant"]

    if not valid_families or not valid_adjuvants:
        print("⚠ Could not generate heatmap: Not enough valid adjuvants o
    else:
        # 4. Build the final heatmap DataFrame from the main 'pivot' tabl
        heatmap_df = (
            pivot.pivot_table(index="Adjuvant", columns="Family", values=
            .loc[valid_adjuvants, valid_families]
        )

        # 5. Restore acronyms for heatmap labels
        heatmap_df.columns = [restore_acronyms(f.title()) for f in heatma

        # [MODIFIED] Use a different colormap for binary (0/1) data
        plt.figure(figsize=(10, 10))
        sns.heatmap(
            heatmap_df,
            cmap="YlGnBu", # A good map for 0/1 data
            linewidths=0.5,
            annot=True, # Will just show 0s and 1s
            fmt=".0f",
            cbar=False # No color bar needed for binary
        )
        plt.title("Top 20 Adjuvants (by Diversity) × Mechanism Families",
        plt.xlabel("Mechanism Family", fontsize=12)
        plt.ylabel("Adjuvant", fontsize=12)
        plt.xticks(rotation=45, ha='right')
        plt.yticks(rotation=0)
        plt.tight_layout()
        heatmap_path = os.path.join(OUTDIR, "adjuvant_family_heatmap.png"
        plt.savefig(heatmap_path, dpi=300)
        plt.close()

        print(f"□ Heatmap saved → {heatmap_path}")
        print("\nAnalysis complete.")

except NameError:
    print("□ ERROR: Could not find 'pivot' or 'family_counts' DataFrames.
    print("Please make sure you have successfully run Cell 7B.")
except Exception as e:
    print(f"□ ERROR: An unexpected error occurred while plotting: {e}")
```

For latest update of the project please fork https://github.com/hurlab/Vaxjo-LLM
