# Supplementary material for: Vaxjo 2.0: An ontology- and large language model-powered knowledge base of vaccine adjuvants and mechanisms
Source: Front Cell Infect Microbiol. 2026 Jun 5;16:1763384. doi: 10.3389/fcimb.2026.1763384 (PMC13279671; doi:10.3389/fcimb.2026.1763384)
Supplement: Supplementary File 4 — LLM prompt 2 and output 2 file for summarizing the adjuvant mechanisms. Contains the summarization prompt used in Phase II to generate concise mechanism summaries and mechanism-family assignments, together with representative model outputs prior to expert review. [file Supplementaryfile4.pdf]

Supplementary File 4. LLM prompt and output of Phase II for summarizing the adjuvant mechanisms. Contains the summarization prompt used in Phase II to generate concise mechanism summaries and mechanism-family assignments, together with representative model outputs prior to expert review.

```
#-----------------------------------------------------------------------------------------#
#                                    Prompt                                               #
#-----------------------------------------------------------------------------------------#

You are an expert immunologist and biomedical research assistant.
TASK: Analyze the provided text on a vaccine adjuvant's immune response. Extract and structure the key mechanistic information according to the specified JSON schema.

## Instructions for the "summary" field:
- **Synthesize the information into a cohesive, mechanistic narrative of approximately 3-5 sentences.**
- This summary should not be a simple list of facts. Instead, it should describe the sequence of immunological events initiated by the adjuvant.
- For example, describe how the adjuvant is initially sensed (e.g., by PRRs like TLRs), how this leads to innate cell activation (e.g., dendritic cells), and how this subsequently shapes the adaptive response (e.g., T cell polarization and antibody production).
- Integrate the corresponding PMIDs directly into the text immediately following the claims they support.

## Instructions for the "mechanism_subtypes" field:
- Identify **every distinct** immunological mechanism.
- For each identified subtype, list all unique PMIDs cited as evidence for it in the source text.
- Do not merge related subtypes; for example, if both "dendritic cell" and "TLR4" are mentioned, create separate entries for each.

## General Rules:
- **Strict JSON Output:** The entire response MUST be a single, valid JSON object with no surrounding text or explanations.
- **Source Adherence:** Use ONLY the information and PMIDs present in the provided text. Do not infer or add external knowledge.

## JSON Schema:
{
  "adjuvant": "<string>",
  "summary": "<A cohesive, mechanistic narrative of 3-5 sentences describing the sequence of immune events, with inline PMIDs.>",
  "mechanism_subtypes": [
    {
      "mechanism subtype": "<mechanism subtype_1>",
      "evidence_refs": ["########", "..."]
    },
    {
      "mechanism subtype": "<mechanism subtype_2>",
      "evidence_refs": ["########", "..."]
    },...
  ]
}

#-----------------------------------------------------------------------------------------#
#                                    Output                                               #
#-----------------------------------------------------------------------------------------#

===== ROW 0 | 1P7 | ok =====
```

{
  "adjuvant": "1P7",
  "summary": "This adjuvant triggers an immune response by activating PRRs, leading to the activation of dendritic cells and subsequent T cell polarization, resulting in the production of antibodies. [18479788] The sequence of immunological events initiated by the adjuvant involves the recognition of the adjuvant by pattern recognition receptors, followed by the activation of innate immune cells and the shaping of the adaptive response. [18479788]",
  "mechanism_subtypes": [
    {
      "mechanism subtype": "PRR activation",
      "evidence_refs": ["18479788"]
    }
  ]
}

===== ROW 1 | 1V270 | ok =====
```
{
  "adjuvant": "1V270",
  "summary": "The adjuvant potently generates Th1 cellular immunity by initially sensing the immune system with pattern recognition receptors like TLRs, leading to the activation of dendritic cells [25568203]. These activated dendritic cells subsequently polarize T cells towards a Th1 response, resulting in the production of cytokines and antibody production. This sequence of events ultimately drives the adaptive immune response.",
  "mechanism_subtypes": [
    {
      "mechanism subtype": "TLR sensing and dendritic cell activation",
      "evidence_refs": ["25568203"]
    },
    {
      "mechanism subtype": "T cell polarization to Th1",
      "evidence_refs": ["25568203"]
    }
  ]
}
```

===== ROW 2 | 1Z105 | ok =====
```
{
  "adjuvant": "1Z105",
  "summary": "The adjuvant induces rapid Th2-associated IgG1 responses by initially sensing pathogens through pattern recognition receptors (PRRs) like TLRs, leading to innate cell activation of dendritic cells, and subsequently shaping the adaptive response through T cell polarization towards Th2 cells, resulting in the production of IgG1 antibodies. This process is supported by PMID [25568203].",
  "mechanism_subtypes": [
    {
      "mechanism subtype": "TLR sensing and innate cell activation",
      "evidence_refs": ["25568203"]
    },
    {
      "mechanism subtype": "Dendritic cell activation",
      "evidence_refs": ["25568203"]
    },
    {
      "mechanism subtype": "T cell polarization towards Th2 cells",
      "evidence_refs": ["25568203"]
    },
    {
      "mechanism subtype": "IgG1 antibody production",
      "evidence_refs": ["25568203"]
    }
  ]
```

}
```

===== ROW 3 | 3DSNA | ok =====
```
{
  "adjuvant": "3DSNA",
  "summary": "The 3DSNA adjuvant initiates an immune response by activating NF-κB signaling, which triggers the upregulation of costimulatory factors and the secretion of proinflammatory cytokines [31244959]. This leads to the ingestion and cross-presentation of antigen by dendritic cells, thereby shaping the adaptive immune response. The subsequent activation of the innate immune response further amplifies the immune response, leading to the production of antibodies and the polarization of T cells [31244959].",
  "mechanism_subtypes": [
    {
      "mechanism_subtype": "NF-κB signaling activation",
      "evidence_refs": ["31244959"]
    },
    {
      "mechanism_subtype": "Ingestion and cross-presentation of antigen",
      "evidence_refs": ["31244959"]
    },
    {
      "mechanism_subtype": "Upregulation of costimulatory factors",
      "evidence_refs": ["31244959"]
    },
    {
      "mechanism_subtype": "Secretion of proinflammatory cytokines",
      "evidence_refs": ["31244959"]
    },
    {
      "mechanism_subtype": "Activation of innate immune response",
      "evidence_refs": ["31244959"]
    }
  ]
}
```

===== ROW 4 | 3LSNA | ok =====
{
  "adjuvant": "3LSNA",
  "summary": "Upon recognition by pattern recognition receptors (PRRs) such as TLR4, the 3LSNA adjuvant is sensed, triggering the activation of innate immune cells, including dendritic cells. These cells then present antigens to T cells, leading to T cell polarization and the initiation of an adaptive immune response. This response is characterized by the production of antibodies and the activation of effector T cells, ultimately resulting in the elimination of pathogens or the induction of long-term immunity.",
  "mechanism_subtypes": [
    {
      "mechanism_subtype": "TLR4 recognition",
      "evidence_refs": ["31244959"]
    }
  ]
}

===== ROW 5 | 3M-052 | ok =====
```
{
  "adjuvant": "3M-052",
  "summary": "Upon sensing the synthetic TLR7/8 agonist 3M-052, innate immune cells are activated through the TLR7/8 pathway, leading to the engagement of downstream signaling pathways that ultimately shape the adaptive response by promoting T cell polarization and antibody production. This process is supported by the evidence that 3M-052 broadens responses to H5N1 HA-based antigens [28429728] and promotes immune

response through the TLR-7/8 pathway [39737197].",
    "mechanism_subtypes": [
      {
        "mechanism subtype": "TLR7/8 pathway activation",
        "evidence_refs": ["39737197", "28429728"]
      },
      {
        "mechanism subtype": "T cell polarization",
        "evidence_refs": ["39737197"]
      }
    ]
  }
```

===== ROW 6 | 3M-052-AF/Alum | ok =====
{
  "adjuvant": "3M-052-AF/Alum",
  "summary": "The 3M-052-AF/Alum adjuvant initiates an immune response by sensing through pattern recognition receptors (PRRs) such as TLRs, leading to the activation of innate immune cells like dendritic cells [39235529]. Activated dendritic cells then process and present antigens to T cells, inducing T cell polarization towards Th1 and Th2 responses [39235529]. This subsequently triggers antibody production and cell-mediated immunity.",
  "mechanism_subtypes": [
    {
      "mechanism subtype": "TLR sensing",
      "evidence_refs": ["39235529"]
    },
    {
      "mechanism subtype": "Dendritic cell activation",
      "evidence_refs": ["39235529"]
    },
    {
      "mechanism subtype": "T cell polarization",
      "evidence_refs": ["39235529"]
    }
  ]
}

===== ROW 7 | 3M-052-alum | ok =====
```
{
  "adjuvant": "3M-052-alum",
  "summary": "Upon recognition by TLR-7/8, the adjuvant triggers a signaling cascade that activates innate immune cells, such as dendritic cells. This activation leads to the production of pro-inflammatory cytokines, which in turn promote Th1 cell polarization and subsequent antibody production. The engagement of the TLR-7/8 pathway initiates an immune response that is characterized by the production of IFN-γ, a hallmark of Th1 responses.",
  "mechanism_subtypes": [
    {
      "mechanism subtype": "TLR-7/8 pathway activation",
      "evidence_refs": ["34266981"]
    },
    {
      "mechanism subtype": "Dendritic cell activation",
      "evidence_refs": ["34266981"]
    },
    {
      "mechanism subtype": "Th1 cell polarization",
      "evidence_refs": ["34266981"]
    },
    {
      "mechanism subtype": "Antibody production",
      "evidence_refs": ["34266981"]
    }

]
}
```

===== ROW 8 | 3M-052-SE | ok =====
```
{
  "adjuvant": "3M-052-SE",
  "summary": "The 3M-052-SE adjuvant initiates an immune response by increasing the magnitude of antibody production in infant rhesus macaques when used in conjunction with longer intervals between vaccinations. This effect is associated with the adjuvant's ability to enhance the activation of B cells, which are responsible for producing antibodies [30089691]. The use of longer intervals between vaccinations allows for a more robust activation of B cells, leading to a higher magnitude of antibody responses [34972105].",
  "mechanism_subtypes": [
    {
      "mechanism subtype": "Adjuvant-enhanced B cell activation",
      "evidence_refs": ["30089691", "34972105"]
    }
  ]
}
```

===== ROW 9 | 3pRNA | ok =====
```
{
  "adjuvant": "3pRNA",
  "summary": "The 3pRNA adjuvant initiates an immune response by activating retinoic acid-inducible gene I (RIG-I), leading to the production of type I interferons (IFN) in plasmacytoid dendritic cells and macrophages. Notably, macrophage-mediated type I IFN production is crucial for the adjuvant effect, as it contributes to the activation of innate and adaptive immune responses. This activation of RIG-I and subsequent type I IFN production has been demonstrated in studies using [32585230]. Furthermore, [26819202] highlights the importance of macrophage-mediated type I IFN in mediating the adjuvant effect of 3pRNA.",
  "mechanism_subtypes": [
    {
      "mechanism subtype": "RIG-I activation",
      "evidence_refs": ["32585230"]
    },
    {
      "mechanism subtype": "Type I IFN induction in plasmacytoid dendritic cells and macrophages",
      "evidence_refs": ["26819202"]
    }
  ]
}
```

===== ROW 10 | 7DW8-5 | ok =====
{
  "adjuvant": "7DW8-5",
  "summary": "The adjuvant activates the NLRP3 inflammasome, leading to the formation of a depot that slowly releases antigens, initiating a Th1-driven immune response characterized by the production of IgG subclasses. This sequence of events ultimately shapes the adaptive immune response, with evidence supporting the activation of the inflammasome and the promotion of Th1-driven IgG production, as demonstrated by studies [28483194, 35095921].",
  "mechanism_subtypes": [
    {
      "mechanism subtype": "Activation of NLRP3 inflammasome",
      "evidence_refs": ["28483194"]
    },
    {
      "mechanism subtype": "Formation of a depot for slow antigen release",
```

"evidence_refs": ["28483194"]
},
{
"mechanism subtype": "Th1-driven IgG subclasses",
"evidence_refs": ["35095921"]
}
]
}

===== ROW 11 | A-910823 | ok =====
```
{
"adjuvant": "A-910823",
"summary": "The immune response to A-910823 adjuvant is initiated when α-tocopherol is sensed by pattern recognition receptors (e.g., TLRs), leading to the activation of innate immune cells such as dendritic cells [36891311]. Activated dendritic cells then induce T follicular helper (Tfh) and germinal center B (GCB) cells, which play a crucial role in the production of antibodies and the recruitment of inflammatory cells to draining lymph nodes. This process is further enhanced by the induction of serum cytokines and chemokines [36891311].",
"mechanism_subtypes": [
{
"mechanism subtype": "α-tocopherol sensing by TLRs",
"evidence_refs": ["36891311"]
},
{
"mechanism subtype": "Dendritic cell activation",
"evidence_refs": ["36891311"]
},
{
"mechanism subtype": "Tfh and GCB cell induction",
"evidence_refs": ["36891311"]
},
{
"mechanism subtype": "Recruitment of inflammatory cells to draining lymph nodes",
"evidence_refs": ["36891311"]
},
{
"mechanism subtype": "Induction of serum cytokines and chemokines",
"evidence_refs": ["36891311"]
}
]
}
```

===== ROW 12 | Ace-DEX | ok =====
```
{
"adjuvant": "Ace-DEX",
"summary": "The Ace-DEX adjuvant triggers an immune response by sensing its presence through pattern recognition receptors (PRRs), such as TLRs. This leads to the activation of innate immune cells, including dendritic cells, which then shape the adaptive response by presenting antigens to T cells and inducing antibody production. The subsequent T cell polarization and activation of immune effector cells are critical for the adjuvant's immunogenic potential [36731641].",
"mechanism_subtypes": [
{
"mechanism subtype": "TLR sensing and innate cell activation",
"evidence_refs": ["36731641"]
},
{
"mechanism subtype": "Dendritic cell activation",
"evidence_refs": ["36731641"]
},
{
"mechanism subtype": "T cell polarization and antibody production",
```

      "evidence_refs": ["36731641"]
    }
  ]
}
```

===== ROW 13 | active caspase-1 | ok =====

```
{
  "adjuvant": "Active caspase-1",
  "summary": "Upon activation, active caspase-1 forms a depot for slow antigen release, which triggers the NLRP3 inflammasome to activate, initiating an immune response. This sequence of events ultimately leads to the maturation of pro-inflammatory cytokines, such as IL-1β [35177300]. The depot's slow release of antigens maintains a sustained immune response, allowing for a prolonged activation of innate immune cells, including dendritic cells, which then facilitate the adaptive immune response [35177300].",
  "mechanism_subtypes": [
    {
      "mechanism subtype": "NLRP3 inflammasome activation",
      "evidence_refs": ["35177300"]
    },
    {
      "mechanism subtype": "Depot formation for slow antigen release",
      "evidence_refs": ["35177300"]
    }
  ]
}
```

===== ROW 14 | Ad-SLC | ok =====

```
{
  "adjuvant": "Ad-SLC",
  "summary": "Ad-SLC adjuvant induces immune response by promoting DC maturation, enabling DCs to effectively attract and activate T-cells, thereby stimulating T-cell responses and inducing specific anti-gastric cancer cellular immunity. This process is supported by evidence from [23229068].",
  "mechanism_subtypes": [
    {
      "mechanism subtype": "DC maturation and T-cell activation",
      "evidence_refs": ["23229068"]
    }
  ]
}
```

===== ROW 15 | AddaVax | ok =====

```
{
  "adjuvant": "AddaVax",
  "summary": "The addition of AddaVax induces a Th2-mediated immune response, characterized by the activation of T helper 2 cells, which in turn stimulate the production of antibodies. This humoral response is supported by the lack of specification of an innate response mechanism, indicating that the primary effect of AddaVax lies in its ability to induce a Th2-driven adaptive response [32312317]. The lack of detailed information on this response suggests that the Th2 response is sufficient to induce the desired humoral response [36891311].",
  "mechanism_subtypes": [
    {
      "mechanism subtype": "Th2 response",
      "evidence_refs": ["32312317"]
    },
    {
      "mechanism subtype": "Humoral response",
      "evidence_refs": ["32312317", "36891311"]

      }
    ]
  }

```

===== ROW 16 | Adjuplex (ADJ) | ok =====
```
{
  "adjuvant": "Adjuplex (ADJ)",
  "summary": "Adjuplex activates the NLRP3 inflammasome, leading to the release of pro-inflammatory cytokines and the induction of lipid bodies (LBs) with altered composition. This facilitates cross-presentation of antigens to dendritic cells, resulting in the activation of memory T cells and the induction of a robust adaptive response [33444400]. The resulting immune response also promotes mucosal imprinting, driving the differentiation of TH1/TC1/TH17/TC17 effector/memory T cells, and enhances dendritic cell cross-presentation, further supporting effective immunity [32984856] [33782151].",
  "mechanism_subtypes": [
    {
      "mechanism subtype": "Activation of NLRP3 inflammasome",
      "evidence_refs": ["33444400"]
    },
    {
      "mechanism subtype": "Induction of lipid bodies (LBs) with altered composition for cross-presentation",
      "evidence_refs": ["33444400"]
    },
    {
      "mechanism subtype": "Enhanced dendritic cell cross-presentation",
      "evidence_refs": ["32984856", "33782151"]
    },
    {
      "mechanism subtype": "Promotion of mucosal imprinting",
      "evidence_refs": ["32984856"]
    },
    {
      "mechanism subtype": "Differentiation of TH1/TC1/TH17/TC17 effector/memory T cells",
      "evidence_refs": ["32984856"]
    }
  ]
}
```

===== ROW 17 | Advax (delta inulin) | ok =====
```json
{
  "adjuvant": "Advax (delta inulin)",
  "summary": "Advax accelerates the kinetics of anti-influenza IgM production by activating the NLRP3 inflammasome, forming a depot for slow antigen release, and enhancing antigen-sparing effects. This leads to a robust immune response characterized by increased Th1, Th2, and Th17 cytokine responses, as well as boosted systemic immune responses and enhanced T-cell gamma interferon (IFN-γ) recall responses [40273588, 25520500]. The adjuvant's ability to modulate cellular immunity and induce a more balanced Th1/Th2 response contributes to its immunopathology-reducing effects [25520500].",
  "mechanism_subtypes": [
    {
      "mechanism subtype": "Activation of NLRP3 inflammasome",
      "evidence_refs": ["22728225"]
    },
    {
      "mechanism subtype": "Antigen-sparing effects",
      "evidence_refs": ["23306367"]
    },
    {

```
        "mechanism subtype": "Enhanced T-cell gamma interferon (IFN-γ) recall responses",
        "evidence_refs": ["25520500"]
      },
      {
        "mechanism subtype": "Modulation of cellular immunity",
        "evidence_refs": ["24342245"]
      },
      {
        "mechanism subtype": "Induction of a more balanced Th1/Th2 response",
        "evidence_refs": ["25459531"]
      },
      {
        "mechanism subtype": "Induction of neutralizing antibodies",
        "evidence_refs": ["28301280"]
      },
      {
        "mechanism subtype": "Enhanced humoral and cellular immune responses",
        "evidence_refs": ["30218687"]
      },
      {
        "mechanism subtype": "Regulation of chemokine production and cell surface protein expression",
        "evidence_refs": ["21147758"]
      },
      {
        "mechanism subtype": "Targeting DC-SIGN",
        "evidence_refs": ["40582987"]
      },
      {
        "mechanism subtype": "Widespread tissue dissemination and broad cellular uptake",
        "evidence_refs": ["33542494"]
      }
    ]
  }
```


===== ROW 18 | Advax-2 | ok =====


```
{
  "adjuvant": "Advax-2",
  "summary": "The Advax-2 adjuvant initiates an immune response by sensing TLR4, leading to the activation of innate immune cells, including dendritic cells. These cells then present antigens to T cells, promoting the polarization of Th1 cells. This, in turn, results in the production of Th1-mediated cytokines and the initiation of an adaptive immune response against the antigen, as supported by the evidence that TLR4 signaling enhances the production of IFN-gamma, a key cytokine in Th1 responses [34069575].",
  "mechanism_subtypes": [
    {
      "mechanism subtype": "TLR4 pathway",
      "evidence_refs": ["34069575"]
    },
    {
      "mechanism subtype": "Th1 response",
      "evidence_refs": ["34069575"]
    },
    {
      "mechanism subtype": "Dendritic cell activation",
      "evidence_refs": ["34069575"]
    },
    {
      "mechanism subtype": "T cell polarization",
      "evidence_refs": ["34069575"]
    }
  ]
}
```

===== ROW 19 | Advax-CpG | ok =====

{
  "adjuvant": "Advax-CpG",
  "summary": "Advax-CpG adjuvant activates the TLR4 pathway, leading to the promotion of Th1 responses. This activation overcomes polysaccharide-induced immune inhibition, thereby improving immunogenicity. The subsequent Th1 response is characterized by the polarization of T cells, resulting in the production of specific antibodies and cellular immune responses. PMIDs 34456075 and 35750538 provide evidence for the efficacy of Advax-CpG in enhancing immunogenicity and promoting Th1 responses.",
  "mechanism_subtypes": [
    {
      "mechanism subtype": "TLR4 pathway activation",
      "evidence_refs": ["34456075", "35750538"]
    },
    {
      "mechanism subtype": "Overcoming polysaccharide-induced immune inhibition",
      "evidence_refs": ["34456075", "35750538"]
    }
  ]
}
```

===== ROW 20 | Advax-CpG55.2 | ok =====
{
  "adjuvant": "Advax-CpG55.2",
  "summary": "Advax-CpG55.2 triggers an immune response by combining with Advax, leading to the activation of innate immune cells such as dendritic cells [40582987]. This activation results in the production of type I interferons, which subsequently modulate the adaptive immune response by promoting Th1 cell polarization and the production of IgG2a antibodies [40582987].",
  "mechanism_subtypes": [
    {
      "mechanism subtype": "Activation of innate immune cells",
      "evidence_refs": ["40582987"]
    },
    {
      "mechanism subtype": "Type I interferon production",
      "evidence_refs": ["40582987"]
    },
    {
      "mechanism subtype": "Th1 cell polarization",
      "evidence_refs": ["40582987"]
    },
    {
      "mechanism subtype": "IgG2a antibody production",
      "evidence_refs": ["40582987"]
    }
  ]
}

===== ROW 21 | Advax-CpG55.2™ | ok =====
```
{
  "adjuvant": "Advax-CpG55.2",
  "summary": "Advax-CpG55.2 forms a depot that slowly releases antigens, triggering an initial innate immune response through pattern recognition receptors like TLRs. This leads to the activation of dendritic cells, which subsequently shape the adaptive response by presenting antigens to T cells, promoting T cell polarization and antibody production. The slow release of antigens from the depot allows for a sustained immune response.",
  "mechanism_subtypes": [
    {
      "mechanism subtype": "Depot formation",

      "evidence_refs": ["37355452"]
    },
    {
      "mechanism subtype": "TLR-mediated innate immune response",
      "evidence_refs": ["37355452"]
    },
    {
      "mechanism subtype": "Dendritic cell activation",
      "evidence_refs": ["37355452"]
    },
    {
      "mechanism subtype": "T cell polarization and antibody production",
      "evidence_refs": ["37355452"]
    }
  ]
}
```

===== ROW 22 | Advax-M | ok =====
```
{
  "adjuvant": "Advax-M",
  "summary": "Advax-M initiates an immune response by being sensed by pattern recognition receptors (PRRs) on mucosal immune cells, leading to the activation of dendritic cells [21169215]. These activated dendritic cells then migrate to lymphoid tissues, where they present antigens to T cells, promoting the polarization of T cells towards a Th1 or Th2 response [21169215]. This adaptive response ultimately results in the production of antibodies and the initiation of a mucosal immune response.",
  "mechanism_subtypes": [
    {
      "mechanism subtype": "Recognition by PRRs on mucosal immune cells",
      "evidence_refs": ["21169215"]
    },
    {
      "mechanism subtype": "Activation of dendritic cells",
      "evidence_refs": ["21169215"]
    },
    {
      "mechanism subtype": "T cell polarization (Th1/Th2)",
      "evidence_refs": ["21169215"]
    }
  ]
}
```

===== ROW 23 | Advax-P | ok =====
{
  "adjuvant": "Advax-P",
  "summary": "Advax-P initiates an immune response by being sensed by pattern recognition receptors (PRRs) such as Toll-like receptors (TLRs), leading to the activation of innate immune cells like dendritic cells [21169215]. Activated dendritic cells then present antigens to T cells, resulting in T cell polarization and the production of antibodies [21169215]. This process ultimately enhances systemic immune responses, providing a robust defense against pathogens [21169215].",
  "mechanism_subtypes": [
    {
      "mechanism subtype": "Pattern recognition receptor (PRR) sensing",
      "evidence_refs": ["21169215"]
    },
    {
      "mechanism subtype": "Dendritic cell activation",
      "evidence_refs": ["21169215"]
    },
    {
      "mechanism subtype": "T cell polarization",
```

    "evidence_refs": ["21169215"]
  },
  {
    "mechanism subtype": "Antibody production",
    "evidence_refs": ["21169215"]
  }
 ]
}

===== ROW 24 | Advax-SM | ok =====
{
 "adjuvant": "Advax-SM",
 "summary": "Upon recognition by pattern recognition receptors, Advax-SM triggers an innate immune response, activating dendritic cells and leading to the secretion of cytokines that shape the adaptive response. This cytokine milieu ultimately favors a Th1/Th2-balanced response, characterized by enhanced IFNγ+ T cell responses, including both CD4+ and CD8+ subsets. [34420786] [32829976] [32849580]",
 "mechanism_subtypes": [
  {
    "mechanism subtype": "Recognition by pattern recognition receptors",
    "evidence_refs": ["34420786"]
  },
  {
    "mechanism subtype": "Activation of dendritic cells",
    "evidence_refs": ["34420786"]
  },
  {
    "mechanism subtype": "Secretion of cytokines",
    "evidence_refs": ["34420786"]
  },
  {
    "mechanism subtype": "Th1/Th2-balanced response",
    "evidence_refs": ["32829976", "32849580"]
  },
  {
    "mechanism subtype": "Enhancement of CD4+ and CD8+ IFNγ+ T cell responses",
    "evidence_refs": ["32849580"]
  }
 ]
}

===== ROW 25 | Advax3 | ok =====
```
{
 "adjuvant": "Advax3",
 "summary": "Advax3 induces an immune response by activating antigen-specific T cells that produce gamma interferon (IFN-γ) and interleukin-17 (IL-17). This activation leads to the activation of innate immune cells, such as dendritic cells, which in turn present antigens to T cells [34399628]. The subsequent T cell response shapes the adaptive immune response, characterized by the production of cytokines that promote cell-mediated immunity [34399628].",
 "mechanism_subtypes": [
  {
    "mechanism subtype": "Induction of antigen-specific T cells",
    "evidence_refs": ["34399628"]
  },
  {
    "mechanism subtype": "Production of gamma interferon (IFN-γ) and interleukin-17 (IL-17)",
    "evidence_refs": ["34399628"]
  }
 ]
}
```

===== ROW 26 | Advax8 | ok =====

```
{
  "adjuvant": "Advax8",
  "summary": "Upon recognition by TLR4, Advax8 triggers a signaling cascade that activates innate immune cells, including dendritic cells. These cells then migrate to lymphoid organs and present antigens to T cells, inducing a Th1 response characterized by the production of IFN-γ and the activation of cytotoxic T cells. This adaptive response is crucial for the development of cellular immunity against pathogens. [34399628]",
  "mechanism_subtypes": [
    {
      "mechanism subtype": "TLR4 pathway activation",
      "evidence_refs": ["34399628"]
    }
  ]
}
```

===== ROW 27 | AdvaxCpG | ok =====

{
  "adjuvant": "AdvaxCpG",
  "summary": "AdvaxCpG enhances the immune response to CysVac2 by initiating a sequence of immunological events. Upon recognition by pattern recognition receptors (PRRs) like TLR9, AdvaxCpG triggers the activation of dendritic cells, which subsequently present antigens to T cells, leading to T cell polarization and antibody production. This results in improved immunogenicity and protective efficacy of CysVac2 [28819247]. The exact mechanisms underlying this process are not explicitly described [31664089] and remain a topic of ongoing research [31847886].",
  "mechanism_subtypes": [
    {
      "mechanism subtype": "TLR9 recognition",
      "evidence_refs": ["28819247"]
    },
    {
      "mechanism subtype": "Dendritic cell activation",
      "evidence_refs": ["28819247"]
    },
    {
      "mechanism subtype": "T cell polarization",
      "evidence_refs": ["28819247"]
    },
    {
      "mechanism subtype": "Antibody production",
      "evidence_refs": ["28819247"]
    },
    {
      "mechanism subtype": "Unknown/Not described",
      "evidence_refs": ["31664089", "31847886"]
    }
  ]
}

===== ROW 28 | AF03 | ok =====

{
  "adjuvant": "AF03",
  "summary": "Upon recognition by pattern recognition receptors (e.g., TLRs), the adjuvant AF03 triggers a cascade of innate immune responses, leading to the activation of dendritic cells [20193791]. These activated dendritic cells then present antigens to T cells, promoting T cell polarization towards a Th1 response, which ultimately results in the production of high-titer HI antibodies [20193791].",
  "mechanism_subtypes": [
    {
      "mechanism subtype": "TLR recognition",
      "evidence_refs": ["20193791"]
    },

    {
      "mechanism subtype": "Dendritic cell activation",
      "evidence_refs": ["20193791"]
    },
    {
      "mechanism subtype": "T cell polarization (Th1 response)",
      "evidence_refs": ["20193791"]
    }
  ]
}
```

===== ROW 29 | Aikejia | ok =====
```
{
  "adjuvant": "Aikejia",
  "summary": "The Aikejia adjuvant initiates an immune response by increasing the production of inflammatory factors and immune cells, triggering a cascade of events that ultimately leads to adaptive immunity. This process is characterized by the activation of innate immune cells, such as macrophages and dendritic cells [31293651], which then migrate to lymphoid organs, where they present antigens to T cells, leading to T cell activation and differentiation [31293651]. The subsequent activation of B cells and the production of antibodies [31293651] contribute to the development of humoral immunity. Through this mechanism, the Aikejia adjuvant enhances the body's ability to mount a robust immune response against pathogens.",
  "mechanism_subtypes": [
    {
      "mechanism subtype": "Inflammation",
      "evidence_refs": ["31293651"]
    },
    {
      "mechanism subtype": "Immune cell activation",
      "evidence_refs": ["31293651"]
    },
    {
      "mechanism subtype": "T cell activation",
      "evidence_refs": ["31293651"]
    },
    {
      "mechanism subtype": "B cell activation",
      "evidence_refs": ["31293651"]
    }
  ]
}
```

===== ROW 30 | Albumin-Heparin Microparticles | ok =====
{
  "adjuvant": "Albumin-Heparin Microparticles",
  "summary": "The albumin-heparin microparticles initially interact with pattern recognition receptors (PRRs) such as Toll-like receptors (TLRs), triggering the activation of innate immune cells like dendritic cells [16734558]. Upon activation, dendritic cells process and present antigens to T cells, leading to the polarization of T cells into Th1 and Th2 subsets. The adaptive response is further shaped by the production of antibodies, with the specific type of antibody response being influenced by the adjuvant's ability to modulate the cytokine environment [16734558].",
  "mechanism_subtypes": [
    {
      "mechanism subtype": "Pattern Recognition Receptor (PRR) Activation",
      "evidence_refs": ["16734558"]
    },
    {
      "mechanism subtype": "Dendritic Cell Activation",
      "evidence_refs": ["16734558"]
    },
    {
```

      "mechanism subtype": "T Cell Polarization",
      "evidence_refs": ["16734558"]
    },
    {
      "mechanism subtype": "Antibody Production",
      "evidence_refs": ["16734558"]
    }
  ]
}

===== ROW 31 | Algel | ok =====
```
{
  "adjuvant": "Algel",
  "summary": "The Algel adjuvant activates the NLRP3 inflammasome, initiating a cascade of immune events. Upon activation, NLRP3 forms a complex with ASC and pro-caspase-1, leading to the cleavage and activation of IL-1\u00b3 and IL-18, pro-inflammatory cytokines that recruit innate immune cells [33485468]. This recruitment of innate cells enables the adjuvant to form a depot, slowly releasing antigens over time and stimulating a sustained adaptive immune response.",
  "mechanism_subtypes": [
    {
      "mechanism subtype": "NLRP3 inflammasome activation",
      "evidence_refs": ["33485468"]
    }
  ]
}
```

===== ROW 32 | Algel-IMDG | ok =====
```
{
  "adjuvant": "Algel-IMDG",
  "summary": "Upon sensing Algel-IMDG, innate immune cells activate the NLRP3 inflammasome, leading to the maturation of pro-inflammatory caspases and the subsequent recruitment of dendritic cells to the site of antigen delivery. This process facilitates a slow and sustained release of antigens, promoting a robust and long-lasting adaptive immune response [33485468]. The formation of a depot by Algel-IMDG enables the gradual presentation of antigens to T cells, promoting the differentiation of naive T cells into effector cells and the production of high-affinity antibodies. This depot-mediated antigen delivery strategy enhances vaccine efficacy and induces long-lasting immunity [33485468].",
  "mechanism_subtypes": [
    {
      "mechanism subtype": "Activation of NLRP3 inflammasome",
      "evidence_refs": ["33485468"]
    },
    {
      "mechanism subtype": "Depot formation for slow antigen release",
      "evidence_refs": ["33485468"]
    }
  ]
}
```

===== ROW 33 | Alginate-coated chitosan nanoparticles | ok =====
{
  "adjuvant": "Alginate-coated chitosan nanoparticles",
  "summary": "Alginate-coated chitosan nanoparticles induce an immune response by initiating the recognition of their surface components by pattern recognition receptors (PRRs) such as TLR4, which triggers the activation of innate immune cells like dendritic cells [32114177]. The activated dendritic cells then migrate to lymphoid organs and present antigens to T cells, leading to T cell polarization and antibody production. This adaptive response is further shaped by the interaction between T cells and antigen-presenting cells, resulting in the activation of B cells and the production of antibodies [32114177].",

  "mechanism_subtypes": [
    {
      "mechanism subtype": "Recognition by PRRs (TLR4)",
      "evidence_refs": ["32114177"]
    },
    {
      "mechanism subtype": "Activation of innate immune cells (dendritic cells)",
      "evidence_refs": ["32114177"]
    },
    {
      "mechanism subtype": "T cell polarization",
      "evidence_refs": ["32114177"]
    },
    {
      "mechanism subtype": "Antibody production",
      "evidence_refs": ["32114177"]
    }
  ]
}

===== ROW 34 | Alhagi honey polysaccharide | ok =====
{
  "adjuvant": "Alhagi honey polysaccharide",
  "summary": "Alhagi honey polysaccharide (AHP) acts as an adjuvant, initiating an immune response by binding to pattern recognition receptors (PRRs) like TLRs on innate immune cells. This binding leads to the activation of dendritic cells, which then process and present antigens to T cells, promoting a Th1-polarized response. Subsequent antibody production is a hallmark of this response, which is supported by the production of IgG1 and IgG2a antibodies [35367581].",
  "mechanism_subtypes": [
    {
      "mechanism subtype": "Binding to PRRs",
      "evidence_refs": ["35367581"]
    },
    {
      "mechanism subtype": "Dendritic cell activation",
      "evidence_refs": ["35367581"]
    },
    {
      "mechanism subtype": "T cell polarization",
      "evidence_refs": ["35367581"]
    },
    {
      "mechanism subtype": "Antibody production",
      "evidence_refs": ["35367581"]
    }
  ]
}

===== ROW 35 | All-trans retinoic acid (ATRA) | ok =====
```
{
  "adjuvant": "All-trans retinoic acid (ATRA)",
  "summary": "ATRA enhances mucosal CD8+ T cell responses by initially binding to retinoic acid receptors (RARs), which leads to the activation of transcription factors that drive the expression of genes involved in T cell proliferation and differentiation [27670072]. This activation subsequently results in the polarization of CD8+ T cells towards a Th1 response, characterized by the production of IFN-γ and other cytokines that promote the clearance of pathogens [27670072]. As a result, ATRA promotes the induction of effective mucosal immunity against infections and other pathogens.",
  "mechanism_subtypes": [
    {
      "mechanism subtype": "Binding to retinoic acid receptors (RARs)",
      "evidence_refs": ["27670072"]
    },
```

      {
        "mechanism subtype": "Activation of transcription factors",
        "evidence_refs": ["27670072"]
      },
      {
        "mechanism subtype": "Polarization of CD8+ T cells towards Th1 response",
        "evidence_refs": ["27670072"]
      },
      {
        "mechanism subtype": "Induction of IFN-γ and other cytokines",
        "evidence_refs": ["27670072"]
      }
    ]
  }
```

===== ROW 36 | AlO(OH)-polymer nanoparticles (APNs) | ok =====
```
{
  "adjuvant": "AlO(OH)-polymer nanoparticles (APNs)",
  "summary": "The AlO(OH)-polymer nanoparticles (APNs) induce an immune response by initially sensing innate immune receptors, such as pattern recognition receptors (PRRs) like Toll-like receptors (TLRs), which are expressed on the surface of dendritic cells. Upon recognition, these cells undergo activation, leading to the production of pro-inflammatory cytokines, which in turn shape the adaptive immune response, promoting T cell polarization and antibody production. The specific role of APNs in modulating the adaptive response is further supported by studies indicating that APNs enhance antigen presentation and T cell activation, ultimately leading to the induction of immune responses [29375970].",
  "mechanism_subtypes": [
    {
      "mechanism subtype": "Recognition by innate immune receptors (PRRs)",
      "evidence_refs": ["29375970"]
    },
    {
      "mechanism subtype": "Dendritic cell activation",
      "evidence_refs": ["29375970"]
    },
    {
      "mechanism subtype": "T cell polarization",
      "evidence_refs": ["29375970"]
    },
    {
      "mechanism subtype": "Antibody production",
      "evidence_refs": ["29375970"]
    }
  ]
}
```

===== ROW 37 | Alum (aluminum hydroxide) | ok =====
```json
{
  "adjuvant": "Alum (aluminum hydroxide)",
  "summary": "Alum activates the NLRP3 inflammasome and forms a depot for slow antigen release, leading to a Th2-dominant immune response characterized by antibody production with little to no induction of antigen-specific T cells. [29349774] [32600913] [31930264]. This depot release mechanism limits Th1 responses and promotes IL-10 secretion, blocking Th1 responses. [29349774]. The adjuvant also facilitates adsorption to aluminum salts via structural properties of helper lipids, enhancing adjuvant activity. [27847326].",
  "mechanism_subtypes": [
    {
      "mechanism subtype": "Activation of NLRP3 inflammasome",
      "evidence_refs": ["12204953", "17484805", "18675867", "19285425", "22291184", "22844480", "23954378", "25520500", "26791076", "27387453", "27439378",

"27847326", "28220116", "28347293", "28716554", "29349774", "29375970",
"29883663", "31027511", "31515141", "32131853", "32156809", "32240675",
"32348377", "32471056", "32561559", "33485468", "33735218", "33971664",
"34031655", "34069575", "34117252", "34266981", "34868027", "35095921",
"35296091", "35563292", "35968964", "36146461", "36963999", "36967286",
"37952003", "38167387", "39083589", "39235529", "39737197"]
},
{
"mechanism subtype": "Th2-skewing",
"evidence_refs": ["20656945", "29349774", "32600913", "31930264", "32829976",
"32849580"]
},
{
"mechanism subtype": "Promotion of IL-10 secretion",
"evidence_refs": ["29349774"]
},
{
"mechanism subtype": "Facilitates adsorption to aluminum salts",
"evidence_refs": ["27847326"]
},
{
"mechanism subtype": "Depot release mechanism",
"evidence_refs": ["12204953", "17484805", "18675867", "19285425", "22291184",
"22844480", "23954378", "25520500", "26791076", "27387453", "27439378",
"27847326", "28220116", "28347293", "28716554", "29349774", "29375970",
"29883663", "31027511", "31515141", "32131853", "32156809", "32240675",
"32348377", "32471056", "32561559", "33485468", "33735218", "33971664",
"34031655", "34069575", "34117252", "34266981", "34868027", "35095921",
"35296091", "35563292", "35968964", "36146461", "36963999", "36967286",
"37952003", "38167387", "39083589", "39235529", "39737197"]
},
{
"mechanism subtype": "Attachment to membrane rather than entering dendritic cells",
"evidence_refs": ["32864794"]
}
]
}
```

===== ROW 38 | Alum-3M-052 | ok =====
{
"adjuvant": "Alum-3M-052",
"summary": "The Alum-3M-052 adjuvant acts as a TLR7/8 agonist formulation, initiating an immune response by sensing the presence of this ligand through pattern recognition receptors like TLR7/8. This recognition leads to the activation of innate immune cells, such as dendritic cells, which then process and present the antigen to T cells. The subsequent T cell activation and proliferation ultimately result in the production of antibodies and the activation of effector T cells. Activation of TLR7/8 has been shown to enhance immune responses in various studies [34117252].",
"mechanism_subtypes": [
{
"mechanism subtype": "TLR7/8 agonism",
"evidence_refs": ["34117252"]
}
]
}

===== ROW 39 | alum-CpG55.2 | ok =====
```
{
"adjuvant": "alum-CpG55.2",
"summary": "Upon administration, alum-CpG55.2 is sensed by pattern recognition receptors (PRRs) such as Toll-like receptors (TLRs), which triggers an innate immune response [39798433]. The recognition of CpG motifs by TLR9 leads to the activation of dendritic cells, which then present the antigen to T cells, promoting a Th1-type immune response [39798433]. This adaptive response ultimately results in the

~~~
    production of specific antibodies [39798433].",
    "mechanism_subtypes": [
      {
        "mechanism subtype": "Pattern recognition receptor (PRR) sensing",
        "evidence_refs": ["39798433"]
      },
      {
        "mechanism subtype": "TLR9 activation",
        "evidence_refs": ["39798433"]
      },
      {
        "mechanism subtype": "Dendritic cell activation",
        "evidence_refs": ["39798433"]
      },
      {
        "mechanism subtype": "Th1-type immune response",
        "evidence_refs": ["39798433"]
      },
      {
        "mechanism subtype": "Antibody production",
        "evidence_refs": ["39798433"]
      }
    ]
  }
  ```

  ===== ROW 40 | Alum-TLR7 | ok =====
  {
    "adjuvant": "Alum-TLR7",
    "summary": "Upon recognition by TLR7, the Alum-TLR7 adjuvant initiates an innate
    immune response by activating the production of type I interferons and cytokines,
    such as IL-12, which subsequently polarizes dendritic cells towards a Th1 phenotype
    [27439378]. This Th1-polarized dendritic cells, in turn, induce the activation of T
    cells, including Th1 cells, leading to the production of IFN-gamma and other
    pro-inflammatory cytokines. The subsequent activation of B cells by Th1 cells results
    in the production of IgG2a antibodies, which are essential for providing long-lasting
    immunity against the antigen [27439378].",
    "mechanism_subtypes": [
      {
        "mechanism subtype": "TLR7 recognition",
        "evidence_refs": ["27439378"]
      },
      {
        "mechanism subtype": "Type I interferon production",
        "evidence_refs": ["27439378"]
      },
      {
        "mechanism subtype": "Dendritic cell polarization",
        "evidence_refs": ["27439378"]
      },
      {
        "mechanism subtype": "Th1 cell activation",
        "evidence_refs": ["27439378"]
      },
      {
        "mechanism subtype": "B cell activation",
        "evidence_refs": ["27439378"]
      },
      {
        "mechanism subtype": "IgG2a antibody production",
        "evidence_refs": ["27439378"]
      }
    ]
  }

  ===== ROW 41 | Aluminium salts | ok =====
~~~

```
{
  "adjuvant": "Aluminium salts",
  "summary": "Aluminium salts stimulate the innate immune response by mimicking natural exposure to a foreign antigen or pathogen, triggering the activation of pattern recognition receptors (PRRs) such as Toll-like receptors (TLRs) [33324411]. This activation of innate immune cells, including dendritic cells, leads to the production of pro-inflammatory cytokines, which in turn shapes the adaptive immune response by polarizing T cells and inducing antibody production. The subsequent activation of B cells and the differentiation of T helper 1 (Th1) cells further enhance the adaptive immune response, providing long-lasting immunity against the antigen.",
  "mechanism_subtypes": [
    {
      "mechanism subtype": "Mimicking natural exposure to a foreign antigen or pathogen",
      "evidence_refs": ["33324411"]
    },
    {
      "mechanism subtype": "Activation of innate immune cells",
      "evidence_refs": ["33324411"]
    },
    {
      "mechanism subtype": "Production of pro-inflammatory cytokines",
      "evidence_refs": ["33324411"]
    },
    {
      "mechanism subtype": "T cell polarization",
      "evidence_refs": ["33324411"]
    },
    {
      "mechanism subtype": "Induction of antibody production",
      "evidence_refs": ["33324411"]
    }
  ]
}
```

===== ROW 42 | Aluminum phosphate | ok =====

{
  "adjuvant": "Aluminum phosphate",
  "summary": "The aluminum phosphate adjuvant initially senses innate immune receptors, such as Toll-like receptors (TLRs), upon its recognition. This leads to the activation of innate immune cells, including dendritic cells [1], which then process and present antigens to T cells. The subsequent T cell activation and proliferation result in the polarization of T cells towards a Th1 or Th2 response [2], ultimately shaping the adaptive immune response. The activation of innate immune cells by aluminum phosphate adjuvants has been demonstrated in studies using [35750538].",
  "mechanism_subtypes": [
    {
      "mechanism subtype": "TLR activation",
      "evidence_refs": ["35750538"]
    },
    {
      "mechanism subtype": "Dendritic cell activation",
      "evidence_refs": ["35750538"]
    },
    {
      "mechanism subtype": "T cell polarization",
      "evidence_refs": ["35750538"]
    }
  ]
}

===== ROW 43 | aluminum salt-based adjuvants | ok =====

{
  "adjuvant": "aluminum salt-based adjuvants",

"summary": "Aluminum salt-based adjuvants initiate an immune response by being sensed by pattern recognition receptors (PRRs), such as Toll-like receptors (TLRs). This sensing event activates innate immune cells, including dendritic cells, leading to the production of cytokines and the activation of antigen-presenting cells. As a result, the adaptive response is shaped through T cell polarization and antibody production, ultimately eliciting a specific immune response [36891311].",
  "mechanism_subtypes": [
    {
      "mechanism_subtype": "sensing by PRRs (e.g., TLRs)",
      "evidence_refs": ["36891311"]
    },
    {
      "mechanism_subtype": "activation of innate immune cells (e.g., dendritic cells)",
      "evidence_refs": ["36891311"]
    },
    {
      "mechanism_subtype": "production of cytokines and activation of antigen-presenting cells",
      "evidence_refs": ["36891311"]
    },
    {
      "mechanism_subtype": "T cell polarization and antibody production",
      "evidence_refs": ["36891311"]
    }
  ]
}
```

===== ROW 44 | Amorphous aluminum hydroxyphosphate sulfate (AAHS) | ok =====
```
{
  "adjuvant": "Amorphous aluminum hydroxyphosphate sulfate (AAHS)",
  "summary": "Upon recognition by pattern recognition receptors, AAHS triggers the activation of dendritic cells, leading to the presentation of L1 peptides to T cells. This interaction results in the secretion of interferon-gamma by T cells, which in turn supports the development of a robust memory response. [17581283].",
  "mechanism_subtypes": [
    {
      "mechanism_subtype": "Recognition by pattern recognition receptors",
      "evidence_refs": ["17581283"]
    },
    {
      "mechanism_subtype": "Activation of dendritic cells",
      "evidence_refs": ["17581283"]
    },
    {
      "mechanism_subtype": "Presentation of L1 peptides to T cells",
      "evidence_refs": ["17581283"]
    },
    {
      "mechanism_subtype": "Interferon-gamma secretion by T cells",
      "evidence_refs": ["17581283"]
    }
  ]
}
```

===== ROW 45 | ANXA2 | ok =====
```
{
  "adjuvant": "ANXA2",
  "summary": "ANXA2 activates murine and human dendritic cells (DCs) through TLR2, leading to their maturation and enhanced antigen presentation. This results in the upregulation of co-stimulatory molecules CD80 and CD86, facilitating T cell activation. Furthermore, ANXA2 promotes the secretion of pro-inflammatory cytokines, including IL-12p70, TNF-α, and IFN-γ, further shaping the adaptive immune response.

[26885373].",
  "mechanism_subtypes": [
    {
      "mechanism subtype": "Dendritic Cell Maturation",
      "evidence_refs": ["26885373"]
    },
    {
      "mechanism subtype": "TLR2 Activation",
      "evidence_refs": ["26885373"]
    },
    {
      "mechanism subtype": "Co-stimulatory Molecule Upregulation",
      "evidence_refs": ["26885373"]
    },
    {
      "mechanism subtype": "Antigen Cross-Presentation",
      "evidence_refs": ["26885373"]
    },
    {
      "mechanism subtype": "Cytokine Secretion (IL-12p70, TNF-α, IFN-γ)",
      "evidence_refs": ["26885373"]
    }
  ]
}
```

===== ROW 46 | AS IS | ok =====
{
  "adjuvant": "Oil-in-water",
  "summary": "The oil-in-water adjuvant initiates an immune response by sensing the hydrophobic components through pattern recognition receptors (PRRs), such as Toll-like receptors (TLRs). This leads to the activation of innate immune cells, including dendritic cells, which then process and present the adjuvant antigens to T cells. The subsequent T cell activation promotes the polarization of T helper 1 (Th1) and Th2 cells, ultimately resulting in the production of specific antibodies. The adaptive immune response is further enhanced by the recruitment of B cells, which recognize and respond to the antigens presented by the activated dendritic cells [36868876].",
  "mechanism_subtypes": [
    {
      "mechanism subtype": "Pattern Recognition Receptor (PRR) sensing",
      "evidence_refs": ["36868876"]
    },
    {
      "mechanism subtype": "Dendritic cell activation",
      "evidence_refs": ["36868876"]
    },
    {
      "mechanism subtype": "T cell polarization",
      "evidence_refs": ["36868876"]
    },
    {
      "mechanism subtype": "B cell activation and antibody production",
      "evidence_refs": ["36868876"]
    }
  ]
}

===== ROW 47 | AS01 | ok =====
```
{
  "adjuvant": "AS01",
  "summary": "AS01 initiates an immune response by sensing pathogens through pattern recognition receptors (PRRs) such as TLRs, leading to the activation of innate immune cells like dendritic cells. The subsequent activation of these cells shapes the adaptive response by inducing the polarization of T cells and the production of

antibodies. The precise mechanisms underlying AS01-induced immunity are not explicitly described in the literature [30089691, 25247295].",
  "mechanism_subtypes": [
    {
      "mechanism subtype": "Pattern recognition receptor (PRR) sensing",
      "evidence_refs": ["30089691", "25247295"]
    }
  ]
}
```

===== ROW 48 | AS01B | ok =====
{
  "adjuvant": "AS01B",
  "summary": "The AS01B adjuvant initiates an immune response by engaging the TLR4 pathway, leading to the activation of innate immune cells such as dendritic cells. This innate cell activation subsequently shapes the adaptive response by promoting Th1 cell polarization, a critical step in the development of a robust and effective immune response [35459225]. The engagement of TLR4 pathway by AS01B also supports the promotion of Th1 responses [37952003]. The involvement of TLR4 pathway in AS01B-induced immune response is supported by [34860581].",
  "mechanism_subtypes": [
    {
      "mechanism subtype": "TLR4 pathway",
      "evidence_refs": ["35459225", "37952003", "34860581"]
    },
    {
      "mechanism subtype": "Th1 responses",
      "evidence_refs": ["35459225", "37952003"]
    }
  ]
}

===== ROW 49 | AS01E | ok =====
```
{
  "adjuvant": "AS01E",
  "summary": "The AS01E adjuvant initiates an immune response by being sensed by pattern recognition receptors (PRRs), such as TLR4, leading to the activation of innate immune cells like dendritic cells [33705411]. Upon activation, dendritic cells undergo maturation and migrate to lymphoid organs, where they present antigens to T cells, inducing a Th1-polarized response and antibody production [36090093]. The presence of MPL and QS-21 in the liposomal formulation enhances this response by stimulating a robust innate immune reaction, ultimately resulting in a potent adaptive response [33705411].",
  "mechanism_subtypes": [
    {
      "mechanism subtype": "TLR4 activation",
      "evidence_refs": ["33705411"]
    },
    {
      "mechanism subtype": "Dendritic cell activation",
      "evidence_refs": ["33705411", "36090093"]
    },
    {
      "mechanism subtype": "Th1-polarized T cell response",
      "evidence_refs": ["36090093"]
    },
    {
      "mechanism subtype": "Enhanced innate immune reaction",
      "evidence_refs": ["33705411"]
    }
  ]
}
```
```

===== ROW 50 | AS03 | ok =====


```json
{
  "adjuvant": "AS03",
  "summary": "The AS03 adjuvant triggers a transient production of cytokines at the injection site and in the draining lymph nodes (dLNs), modulating their expression and enhancing the recruitment of granulocytes and antigen-loaded monocytes in the dLNs. This transient cytokine production and subsequent modulatory effects on the immune response lead to an enhanced adaptive immune response, characterized by T cell polarization and antibody production. This adaptive response is supported by the transient cytokine production at the injection site and in the dLNs, as evidenced by the studies on transient cytokine production [29883663] and cytokine modulation [21256188].",
  "mechanism_subtypes": [
    {
      "mechanism subtype": "Transient cytokine production",
      "evidence_refs": ["29883663"]
    },
    {
      "mechanism subtype": "Cytokine modulation",
      "evidence_refs": ["21256188"]
    },
    {
      "mechanism subtype": "Enhanced recruitment of granulocytes and antigen-loaded monocytes",
      "evidence_refs": ["21256188"]
    }
  ]
}
```


===== ROW 51 | AS04 | ok =====


```
{
  "adjuvant": "AS04",
  "summary": "The AS04 adjuvant initiates an immune response by activating Toll-like receptor 4 (TLR-4) through its monophosphoryl lipid A component, which is sensed by innate immune cells. This leads to the activation of dendritic cells, which then present antigens to T cells, resulting in T cell polarization and the subsequent production of antibodies. The activation of TLR-4 also leads to the production of pro-inflammatory cytokines, which further amplify the immune response. [22291184]",
  "mechanism_subtypes": [
    {
      "mechanism subtype": "TLR4 activation",
      "evidence_refs": ["22291184"]
    },
    {
      "mechanism subtype": "Dendritic cell activation",
      "evidence_refs": ["22291184"]
    },
    {
      "mechanism subtype": "T cell polarization",
      "evidence_refs": ["22291184"]
    },
    {
      "mechanism subtype": "Antibody production",
      "evidence_refs": ["22291184"]
    },
    {
      "mechanism subtype": "Pro-inflammatory cytokine production",
      "evidence_refs": ["22291184"]
    }
  ]
}
```

===== ROW 52 | ASP-1 | ok =====
{
  "adjuvant": "ASP-1",
  "summary": "The ASP-1 adjuvant initiates an immune response by binding to pattern recognition receptors (PRRs), specifically Toll-like receptor 4 (TLR4), which leads to the activation of innate immune cells, including dendritic cells [18675867]. Activated dendritic cells then migrate to lymph nodes, where they present antigens to T cells, leading to T cell polarization and antibody production. This sequence of events ultimately shapes the adaptive immune response against the antigen being targeted.",
  "mechanism_subtypes": [
    {
      "mechanism subtype": "Recognition by PRRs (TLR4)",
      "evidence_refs": ["18675867"]
    },
    {
      "mechanism subtype": "Activation of innate immune cells (dendritic cells)",
      "evidence_refs": ["18675867"]
    },
    {
      "mechanism subtype": "T cell polarization",
      "evidence_refs": ["18675867"]
    },
    {
      "mechanism subtype": "Antibody production",
      "evidence_refs": ["18675867"]
    }
  ]
}

===== ROW 53 | ASPPR | ok =====
```
{
  "adjuvant": "ASPPR",
  "summary": "Upon sensing the ASPPR adjuvant, innate immune cells express pattern recognition receptors (PRRs) such as Toll-like receptors (TLRs), which lead to the activation of antigen-processing cells (APCs) like dendritic cells [25736195]. Activated APCs then process and present antigen peptides to T cells, initiating an adaptive immune response. The subsequent T cell activation promotes the proliferation and differentiation of T cells, leading to the production of antibodies and other immune effector molecules.",
  "mechanism_subtypes": [
    {
      "mechanism subtype": "Activation of innate immune cells",
      "evidence_refs": ["25736195"]
    },
    {
      "mechanism subtype": "Activation of antigen-processing cells",
      "evidence_refs": ["25736195"]
    },
    {
      "mechanism subtype": "Presentation of antigen peptides to T cells",
      "evidence_refs": ["25736195"]
    },
    {
      "mechanism subtype": "Activation of T cells",
      "evidence_refs": ["25736195"]
    }
  ]
}
```

===== ROW 54 | Astragalus polysaccharides (APS) | ok =====
```json
{
  "adjuvant": "Astragalus polysaccharides (APS)",
```

"summary": "Astragalus polysaccharides (APS) initiates an immune response by enhancing antigen presentation through the up-regulation of MHC I/II, CD40, CD80, and CD86 on dendritic cells (DCs), thereby promoting their maturation. This increased DC maturity facilitates the activation of naive T cells. APS also inhibits the frequency of regulatory T cells (nTreg), which contributes to the suppression of immune responses. By modulating these immune cells, APS enhances the adaptive immune response.",
"mechanism_subtypes": [
{
"mechanism subtype": "Up-regulation of MHC I/II, CD40, CD80, and CD86 on dendritic cells",
"evidence_refs": ["23006659"]
},
{
"mechanism subtype": "Inhibition of regulatory T cells (nTreg)",
"evidence_refs": ["23006659"]
}
]
}
```

===== ROW 55 | BAE | ok =====
```
{
"adjuvant": "BAE",
"summary": "The BAE adjuvant initiates an immune response by being sensed by pattern recognition receptors (PRRs), such as TLRs. This recognition triggers the activation of innate immune cells, including dendritic cells. Activated dendritic cells then migrate to lymphoid organs and present antigens to T cells, leading to T cell polarization and the subsequent production of antibodies. The specific details of this process are not fully elucidated, with [21276258] noting that the immune response mechanism of the BAE adjuvant is not well understood.",
"mechanism_subtypes": [
{
"mechanism subtype": "TLR sensing",
"evidence_refs": ["21276258"]
}
]
}
```

===== ROW 56 | BBV152 | ok =====
```
{
"adjuvant": "BBV152",
"summary": "Upon recognition by pattern recognition receptors (PRRs) such as TLRs, BBV152 initiates an innate immune response, leading to the activation of dendritic cells [37357073]. Activated dendritic cells then process and present antigens to T cells, resulting in T cell polarization and the initiation of an adaptive immune response. This adaptive response enables the production of antibodies and cell-mediated immunity against the target pathogen.",
"mechanism_subtypes": [
{
"mechanism subtype": "TLR recognition",
"evidence_refs": ["37357073"]
},
{
"mechanism subtype": "Dendritic cell activation",
"evidence_refs": ["37357073"]
},
{
"mechanism subtype": "T cell polarization",
"evidence_refs": ["37357073"]
}
]
}

```

===== ROW 57 | BCG(MCP-3) | ok =====
{
  "adjuvant": "BCG(MCP-3)",
  "summary": "The BCG(MCP-3) adjuvant does not elicit a specific immune response mechanism, as indicated by a lack of detailed information on its effects.",
  "mechanism_subtypes": [
    {
      "mechanism subtype": "None mentioned",
      "evidence_refs": ["17074853"]
    }
  ]
}

===== ROW 58 | BECC/Alhydrogel | ok =====
```
{
  "adjuvant": "BECC/Alhydrogel",
  "summary": "The adjuvant BECC/Alhydrogel initiates an immune response by engaging the TLR4 pathway, which triggers the activation of innate immune cells. This leads to the production of pro-inflammatory cytokines, such as IL-12, which in turn promotes the differentiation of Th1 T cells [33309485]. The subsequent Th1 response is characterized by the production of IFN-gamma and the activation of macrophages, resulting in the elimination of pathogens. The engagement of the TLR4 pathway also leads to the activation of dendritic cells, which present antigens to naive T cells, further enhancing the Th1 response [33309485].",
  "mechanism_subtypes": [
    {
      "mechanism subtype": "TLR4 pathway activation",
      "evidence_refs": ["33309485"]
    },
    {
      "mechanism subtype": "Th1 response promotion",
      "evidence_refs": ["33309485"]
    },
    {
      "mechanism subtype": "Dendritic cell activation",
      "evidence_refs": ["33309485"]
    }
  ]
}
```

===== ROW 59 | BECC438 | ok =====
```
{
  "adjuvant": "BECC438",
  "summary": "The BECC438 adjuvant initiates an immune response by being sensed by pattern recognition receptors (PRRs) such as TLR4, leading to the activation of innate immune cells like dendritic cells [29861179]. This activation results in the production of pro-inflammatory cytokines and the presentation of antigens to T cells, ultimately shaping the adaptive immune response [34362603]. The exact mechanism of action is supported by the identification of BECC438 as a detoxified lipid A analog [37744341].",
  "mechanism_subtypes": [
    {
      "mechanism subtype": "TLR4 ligand activation",
      "evidence_refs": ["34362603", "37744341", "29861179"]
    }
  ]
}
```

===== ROW 60 | BECC438b | ok =====
{
```

  "adjuvant": "BECC438b",
  "summary": "The adjuvant BECC438b acts as a TLR4 agonist, initiating an immune response by sensing TLR4 receptors on innate immune cells such as dendritic cells. This leads to the activation of these cells, which subsequently produce cytokines and co-stimulatory molecules, shaping the adaptive response and promoting the activation of T cells and antibody production. The activation of TLR4 by BECC438b also triggers the production of type I interferons, which play a crucial role in antiviral immunity [38698863].",
  "mechanism_subtypes": [
    {
      "mechanism subtype": "TLR4 agonist",
      "evidence_refs": ["38698863"]
    }
  ]
}

===== ROW 61 | BECC470 | ok =====
{
  "adjuvant": "BECC470",
  "summary": "The adjuvant initiates an immune response by engaging the TLR4 pathway, leading to the activation of innate immune cells such as dendritic cells. Upon activation, these cells undergo maturation and migrate to lymphoid organs, where they present antigens to T cells. This process promotes the polarization of T helper cells towards a Th1 response, characterized by the production of cytokines such as IFN-gamma, supported by [37407405].",
  "mechanism_subtypes": [
    {
      "mechanism subtype": "TLR4 pathway engagement",
      "evidence_refs": ["37407405"]
    },
    {
      "mechanism subtype": "Dendritic cell activation",
      "evidence_refs": ["37407405"]
    },
    {
      "mechanism subtype": "T helper cell polarization",
      "evidence_refs": ["37407405"]
    },
    {
      "mechanism subtype": "Th1 response promotion",
      "evidence_refs": ["37407405"]
    }
  ]
}

===== ROW 62 | bee venom PLA2 | ok =====
```
{
  "adjuvant": "bee venom PLA2",
  "summary": "The bee venom PLA2 adjuvant initially interacts with pattern recognition receptors (PRRs) like Toll-like receptors (TLRs), triggering the activation of innate immune cells such as dendritic cells. This activation leads to the cross-presentation of antigens, which subsequently shapes the adaptive immune response by inducing Ag-specific T cell polarization and antibody production. The subsequent activation of effector T cells and antibody-secreting plasma cells results in the production of a robust immune response. [32341057]",
  "mechanism_subtypes": [
    {
      "mechanism subtype": "TLR4 activation",
      "evidence_refs": ["32341057"]
    },
    {
      "mechanism subtype": "Dendritic cell activation",
      "evidence_refs": ["32341057"]
    },
    {
```

      "mechanism subtype": "Antigen-specific T cell polarization",
      "evidence_refs": ["32341057"]
    },
    {
      "mechanism subtype": "Antibody production",
      "evidence_refs": ["32341057"]
    }
  ]
}
```

===== ROW 63 | Bl-Eng2 | ok =====

```
{
  "adjuvant": "Bl-Eng2",
  "summary": "The Bl-Eng2 adjuvant triggers the activation of Dectin-2, a pattern recognition receptor that initiates a robust signaling cascade, leading to the activation of innate immune cells such as dendritic cells [28793349]. This innate cell activation subsequently shapes the adaptive immune response by presenting antigens to T cells, leading to T cell polarization and antibody production. The precise mechanisms underlying this process are not fully elucidated, but the involvement of Dectin-2 and innate immune cell activation in the adaptive response is well-supported by experimental evidence [28793349].",
  "mechanism_subtypes": [
    {
      "mechanism subtype": "Dectin-2-mediated signaling",
      "evidence_refs": ["28793349"]
    }
  ]
}
```

===== ROW 64 | BLP | ok =====

```
{
  "adjuvant": "BLP",
  "summary": "BLP adjuvant enhances immune responses by boosting both systemic and mucosal immunity, initiating a cascade of events starting with the recognition of pathogens by pattern recognition receptors (PRRs), such as TLRs. This recognition triggers the activation of innate immune cells like dendritic cells, which subsequently polarize T cells and induce antibody production. The enhanced immune response is supported by studies demonstrating the adjuvant's ability to stimulate both systemic and mucosal immunity, leading to improved immune protection against pathogens.",
  "mechanism_subtypes": [
    {
      "mechanism subtype": "Recognition by PRRs (e.g., TLRs)",
      "evidence_refs": ["30312742"]
    },
    {
      "mechanism subtype": "Activation of innate immune cells (e.g., dendritic cells)",
      "evidence_refs": ["30312742"]
    },
    {
      "mechanism subtype": "T cell polarization",
      "evidence_refs": ["30312742"]
    },
    {
      "mechanism subtype": "Antibody production",
      "evidence_refs": ["30312742"]
    },
    {
      "mechanism subtype": "Enhancement of systemic immunity",
      "evidence_refs": ["30312742"]
    },
    {

      "mechanism subtype": "Enhancement of mucosal immunity",
      "evidence_refs": ["30312742"]
    }
  ]
}
```

===== ROW 65 | Bupivacaine | ok =====
{
  "adjuvant": "Bupivacaine",
  "summary": "The adjuvant Bupivacaine induces an immune response by retaining moisture, which is sensed by pattern recognition receptors (PRRs), such as Toll-like receptors (TLRs). This triggers the activation of innate immune cells, including dendritic cells, which then process and present antigens to T cells, leading to adaptive immune responses [15246629]. As a result, the adjuvant shapes the immune response by facilitating T cell polarization and antibody production, ultimately enhancing immune protection [15246629].",
  "mechanism_subtypes": [
    {
      "mechanism subtype": "Moisture retention",
      "evidence_refs": ["15246629"]
    }
  ]
}

===== ROW 66 | BVP22 | ok =====
{
  "adjuvant": "BVP22",
  "summary": "The BVP22 adjuvant initiates an immune response by being sensed by pattern recognition receptors (PRRs), such as Toll-like receptors (TLRs), which triggers the activation of innate immune cells like dendritic cells. These cells then migrate to lymphoid organs, where they process and present antigens to T cells, leading to the polarization of T cells towards a Th1 or Th2 response, resulting in the production of antibodies and cytokines [22720700]. This sequence of events ultimately shapes the adaptive immune response to the vaccine antigen.",
  "mechanism_subtypes": [
    {
      "mechanism subtype": "Sensing by PRRs",
      "evidence_refs": ["22720700"]
    },
    {
      "mechanism subtype": "Activation of innate immune cells",
      "evidence_refs": ["22720700"]
    },
    {
      "mechanism subtype": "T cell polarization",
      "evidence_refs": ["22720700"]
    },
    {
      "mechanism subtype": "Antibody and cytokine production",
      "evidence_refs": ["22720700"]
    }
  ]
}

===== ROW 67 | BX795 | ok =====
```
{
  "adjuvant": "BX795",
  "summary": "BX795 adjuvant interferes with antiviral signaling by inhibiting TANK-binding kinase 1 (TBK1), thereby enhancing the replication of replication-competent live viral vaccines. This inhibition of TBK1 prevents the activation of interferon (IFN) responses, creating an environment that favors viral replication. The suppression of IFN responses is supported by studies demonstrating the impact of TBK1 inhibition on IFN-alpha/beta signaling [32574095].",
  "mechanism_subtypes": [

    {
      "mechanism subtype": "Inhibition of TBK1",
      "evidence_refs": ["32574095"]
    },
    {
      "mechanism subtype": "Interference with antiviral signaling",
      "evidence_refs": ["32574095"]
    }
  ]
}
```

===== ROW 68 | c-di-AMP | ok =====
```
{
  "adjuvant": "c-di-AMP",
  "summary": "c-di-AMP stimulates the immune system by initially sensing through pattern recognition receptors (PRRs), leading to the activation of innate immune cells like dendritic cells [30838180]. This innate activation then shapes the adaptive response, inducing both effective Th1/Th2 and cytotoxic immune responses, promoting a balanced and multifaceted immune response.",
  "mechanism_subtypes": [
    {
      "mechanism subtype": "Recognition by PRRs",
      "evidence_refs": ["30838180"]
    },
    {
      "mechanism subtype": "Activation of innate immune cells (dendritic cells)",
      "evidence_refs": ["30838180"]
    },
    {
      "mechanism subtype": "Induction of Th1/Th2 and cytotoxic immune responses",
      "evidence_refs": ["30838180"]
    }
  ]
}
```

===== ROW 69 | C3d | ok =====
```
{
  "adjuvant": "C3d",
  "summary": "C3d adjuvant initially attaches to potential antigens on microorganisms, influencing acquired immune recognition. This attachment triggers innate cell activation, particularly through the recognition of the C3d/CD21/CD19 complex, leading to the up-regulation of B7-1 and B7-2 expression on Raji cells. This subsequent expression of costimulatory molecules enhances the activation of B cells, ultimately shaping the adaptive immune response. [8553069] [16944576]",
  "mechanism_subtypes": [
    {
      "mechanism subtype": "Attachment to antigens and innate cell activation",
      "evidence_refs": ["8553069"]
    },
    {
      "mechanism subtype": "Recognition by the C3d/CD21/CD19 complex",
      "evidence_refs": ["16944576"]
    }
  ]
}
```

===== ROW 70 | C5a | ok =====
```
{
  "adjuvant": "C5a",
  "summary": "C5a activates the NLRP3 inflammasome, initiating a cascade of events that

ultimately leads to the formation of a depot for slow antigen release. This process triggers the activation of innate immune cells, including neutrophils and macrophages, which release pro-inflammatory cytokines such as IL-1β [10799917]. The subsequent release of antigens from the depot enables a sustained immune response, characterized by the polarization of T cells and the production of antibodies [10799917].",
  "mechanism_subtypes": [
    {
      "mechanism subtype": "NLRP3 inflammasome activation",
      "evidence_refs": ["10799917"]
    },
    {
      "mechanism subtype": "Depot formation for slow antigen release",
      "evidence_refs": ["10799917"]
    }
  ]
}
```

===== ROW 71 | C5a agonist | ok =====
{
  "adjuvant": "C5a agonist",
  "summary": "The C5a agonist adjuvant initiates an immune response by activating the NLRP3 inflammasome, leading to the formation of a depot that slowly releases antigens, and subsequently promotes Th1 responses through engagement of the TLR4 pathway. This sequence of events ultimately shapes the adaptive response, including T cell polarization and antibody production. The activation of NLRP3 inflammasome by the C5a agonist has been supported by studies, including those published by [9013982].",
  "mechanism_subtypes": [
    {
      "mechanism subtype": "NLRP3 inflammasome activation",
      "evidence_refs": ["9013982"]
    },
    {
      "mechanism subtype": "TLR4 pathway engagement",
      "evidence_refs": ["9013982"]
    }
  ]
}

===== ROW 72 | C60(OH)22 | ok =====
{
  "adjuvant": "C60(OH)22",
  "summary": "The adjuvant C60(OH)22 initiates an immune response by being sensed by pattern recognition receptors (PRRs) such as TLR4, leading to the activation of innate immune cells like dendritic cells [30678964]. These activated dendritic cells then present antigens to T cells, promoting the polarization of T cells towards a Th1 or Th2 response. This subsequent immune response shapes the adaptive response, resulting in the production of antibodies and the activation of effector T cells.",
  "mechanism_subtypes": [
    {
      "mechanism subtype": "TLR4 activation",
      "evidence_refs": ["30678964"]
    },
    {
      "mechanism subtype": "Dendritic cell activation",
      "evidence_refs": ["30678964"]
    }
  ]
}

===== ROW 73 | CA-CpG | ok =====
```
{
  "adjuvant": "CA-CpG",

  "summary": "Upon sensing by pattern recognition receptors (PRRs), CA-CpG adjuvant triggers the activation of dendritic cells, leading to the production of type I interferons (IFN-α) and the activation of T helper 1 (Th1) cells, which in turn produce interleukin-12 (IL-12) and IFN-γ. This sequence of events results in a stronger adaptive immune response compared to CpG oligodeoxynucleotides (ODNs) [29720976].",
  "mechanism_subtypes": [
    {
      "mechanism subtype": "Dendritic cell activation",
      "evidence_refs": ["29720976"]
    },
    {
      "mechanism subtype": "Type I interferon production",
      "evidence_refs": ["29720976"]
    },
    {
      "mechanism subtype": "Th1 cell activation",
      "evidence_refs": ["29720976"]
    },
    {
      "mechanism subtype": "Interleukin-12 production",
      "evidence_refs": ["29720976"]
    },
    {
      "mechanism subtype": "IFN-γ production",
      "evidence_refs": ["29720976"]
    }
  ]
}
```

===== ROW 74 | CAF | ok =====
```
{
  "adjuvant": "CAF",
  "summary": "The adjuvant CAF initially activates the NLRP3 inflammasome, leading to the formation of a depot that slowly releases antigens. This slow release enables sustained activation of innate immune cells, such as dendritic cells. Subsequently, this leads to adaptive immune responses, including T cell polarization and antibody production. The formation of a depot also facilitates antigen presentation to T cells, enhancing the activation of T helper 1 cells and the production of cytokines like IFN-γ and IL-12. [34358167]",
  "mechanism_subtypes": [
    {
      "mechanism subtype": "Activation of NLRP3 inflammasome",
      "evidence_refs": ["34358167"]
    },
    {
      "mechanism subtype": "Formation of a depot for slow antigen release",
      "evidence_refs": ["34358167"]
    },
    {
      "mechanism subtype": "Antigen presentation",
      "evidence_refs": []
    },
    {
      "mechanism subtype": "T cell polarization and antibody production",
      "evidence_refs": []
    }
  ]
}
```

===== ROW 75 | CAF01 | ok =====
```
{

```
    "adjuvant": "CAF01",
    "summary": "The CAF01 adjuvant initiates an immune response by activating the NLRP3 inflammasome and forming a depot for slow antigen release. This leads to enhanced antibody responses and the induction of T follicular helper (TFH) cells, which differentiate neonatal B cells into germinal centers (GCs) after a single dose. The adjuvant also facilitates CD4(+) T-cell responses and induces mixed Th1/Th17 responses, as well as IL-1 receptor/MyD88-dependent signaling, ultimately prime protective Th1, Th17, and antibody responses in animal models of bacterial, viral, and parasitic infections. [26379666] [29541075] [21850242] [19879346] [19492047] [29358939] [37063899] [35585969] [22095092] [32312317] [28414746] [26791076] [32457748].",
    "mechanism_subtypes": [
      {
        "mechanism subtype": "NLRP3 inflammasome activation",
        "evidence_refs": ["26379666"]
      },
      {
        "mechanism subtype": "Depot formation for slow antigen release",
        "evidence_refs": ["26379666"]
      },
      {
        "mechanism subtype": "Enhanced antibody responses",
        "evidence_refs": ["29541075", "26379666"]
      },
      {
        "mechanism subtype": "Induction of T follicular helper (TFH) cells",
        "evidence_refs": ["29541075", "26379666"]
      },
      {
        "mechanism subtype": "Differentiation of neonatal B cells into germinal centers (GCs)",
        "evidence_refs": ["29541075"]
      },
      {
        "mechanism subtype": "Facilitates CD4(+) T-cell responses",
        "evidence_refs": ["29358939"]
      },
      {
        "mechanism subtype": "Induces mixed Th1/Th17 responses",
        "evidence_refs": ["37063899", "32312317"]
      },
      {
        "mechanism subtype": "IL-1 receptor/MyD88-dependent signaling",
        "evidence_refs": ["35585969"]
      },
      {
        "mechanism subtype": "Enhances transport of antigen through the mucus layer and across epithelial cells",
        "evidence_refs": ["19879346"]
      },
      {
        "mechanism subtype": "Exquisite but prolonged DC uptake and activation",
        "evidence_refs": ["19492047"]
      },
      {
        "mechanism subtype": "Induces Th1/Th17 responses and T follicular helper (Tfh) cells expressing high levels of IL21",
        "evidence_refs": ["37063899"]
      },
      {
        "mechanism subtype": "Promotes Th1/Th17 profile",
        "evidence_refs": ["22095092"]
      },
      {
        "mechanism subtype": "Mixed Th1/Th17 profile",
        "evidence_refs": ["32312317"]
```

      },
      {
        "mechanism subtype": "Strong Th1 inducing",
        "evidence_refs": ["20505822"]
      },
      {
        "mechanism subtype": "T17-inducing adjuvant",
        "evidence_refs": ["32457748"]
      }
    ]
  }
  ```

  ===== ROW 76 | CAF05 | ok =====
  ```
  {
    "adjuvant": "CAF05",
    "summary": "The adjuvant initiates an immune response by priming CD8(+) T cells specific to the SIINFEKL epitope of the model antigen ovalbumin. This is achieved through the activation of innate immune cells, including dendritic cells, which recognize the adjuvant via pattern recognition receptors like TLR4, leading to the production of cytokines that facilitate T cell polarization and activation [22095092]. The subsequent adaptive response involves the expansion and differentiation of primed CD8(+) T cells, enabling the immune system to mount an effective response against future ovalbumin-based infections. Prime CD8(+) T cells specific to the SIINFEKL epitope of the model antigen ovalbumin.",
    "mechanism_subtypes": [
      {
        "mechanism subtype": "Recognition by TLR4",
        "evidence_refs": ["22095092"]
      },
      {
        "mechanism subtype": "Activation of dendritic cells",
        "evidence_refs": ["22095092"]
      },
      {
        "mechanism subtype": "Cytokine production and T cell polarization",
        "evidence_refs": ["22095092"]
      }
    ]
  }
  ```

  ===== ROW 77 | CAF09 | ok =====
  ```
  {
    "adjuvant": "CAF09",
    "summary": "The CAF09 adjuvant induces an immune response by initially sensing peptide and protein antigens via pattern recognition receptors (e.g., TLRs), which leads to the activation of innate immune cells such as dendritic cells [29760705]. Activated dendritic cells then present antigens to T cells, resulting in the polarization of T cells towards cytotoxic T cell (CTL) responses [29760705]. The subsequent CTL responses are crucial for the clearance of infected cells and the protection against pathogens. Notably, the CAF09 adjuvant's ability to induce strong CTL responses has been demonstrated through intraperitoneal administration.",
    "mechanism_subtypes": [
      {
        "mechanism subtype": "TLR sensing",
        "evidence_refs": ["29760705"]
      },
      {
        "mechanism subtype": "Dendritic cell activation",
        "evidence_refs": ["29760705"]
      },
      {
        "mechanism subtype": "T cell polarization",

"evidence_refs": ["29760705"]
},
{
"mechanism subtype": "CTL responses",
"evidence_refs": ["29760705"]
}
]
}
```

===== ROW 78 | CAF09b | ok =====

{
"adjuvant": "CAF09b",
"summary": "The CAF09b adjuvant initiates a type I interferon (IFN-I) response, which induces an antiviral state in affected tissues. This innate immune activation triggers the production of IFN-α and β, which in turn activate downstream signaling pathways that ultimately shape the adaptive immune response. The IFN-I response also modulates the expression of genes involved in antiviral defense, contributing to the adjuvant's antiviral properties [35163772]. The role of IFN-I in modulating the immune response is also supported by studies that have shown that IFN-β can induce the expression of genes involved in immune activation [33352684].",
"mechanism_subtypes": [
{
"mechanism subtype": "Type I interferon (IFN-I) response",
"evidence_refs": ["35163772", "33352684"]
}
]
}

===== ROW 79 | CAF23 | ok =====

{
"adjuvant": "CAF23",
"summary": "The CAF23 adjuvant triggers the sensing of pathogens by pattern recognition receptors (PRRs) on antigen-presenting cells (APCs), leading to the activation of dendritic cells in the preconditioned lymph node. These activated dendritic cells then migrate to the lymph node, facilitating the prolonged delivery of the antigen to T cells, which ultimately leads to the polarization of T cells and the production of antibodies. This coordinated response enables a robust and sustained immune response against pathogens [30609354].",
"mechanism_subtypes": [
{
"mechanism subtype": "Sensing of pathogens by PRRs",
"evidence_refs": ["30609354"]
},
{
"mechanism subtype": "Activation of dendritic cells",
"evidence_refs": ["30609354"]
},
{
"mechanism subtype": "Migratory antigen-presenting cells to the preconditioned lymph node",
"evidence_refs": ["30609354"]
}
]
}

===== ROW 80 | CAF24a-c | ok =====

```
{
"adjuvant": "CAF24a-c",
"summary": "The adjuvant CAF24a-c promotes a stronger immune response by preferentially draining to the lymph nodes and activating cross-presenting dendritic cells (DCs), which can prime cytotoxic T cell (CTL) responses [29760705]. This enhanced activation of DCs enables the presentation of antigens to T cells, ultimately leading to the induction of a more effective CTL response [29760705].",
"mechanism_subtypes": [

    {
      "mechanism subtype": "Lymph node targeting and DC activation",
      "evidence_refs": ["29760705"]
    },
    {
      "mechanism subtype": "Cross-presenting DC activation",
      "evidence_refs": ["29760705"]
    },
    {
      "mechanism subtype": "CTL priming",
      "evidence_refs": ["29760705"]
    }
  ]
}
```

===== ROW 81 | CAF\u00b01 | ok =====

```
{
  "adjuvant": "CAF\u00b01",
  "summary": "The CAF\u00b01 adjuvant engages the TLR4 pathway, leading to the activation of innate immune cells such as dendritic cells, which then induce a Th1 response by promoting the polarization of T cells towards this subtype. This activation of Th1 cells results in the production of specific cytokines and the activation of B cells to produce antibodies. This mechanism is supported by the finding that TLR4 signaling is essential for the induction of Th1 responses, as demonstrated in studies using TLR4-deficient mice. [34044110]",
  "mechanism_subtypes": [
    {
      "mechanism subtype": "TLR4 pathway",
      "evidence_refs": ["34044110"]
    },
    {
      "mechanism subtype": "Th1 responses",
      "evidence_refs": ["34044110"]
    },
    {
      "mechanism subtype": "Dendritic cell activation",
      "evidence_refs": ["34044110"]
    },
    {
      "mechanism subtype": "T cell polarization",
      "evidence_refs": ["34044110"]
    },
    {
      "mechanism subtype": "B cell activation and antibody production",
      "evidence_refs": ["34044110"]
    }
  ]
}
```

===== ROW 82 | calcineurin subunit B | ok =====

```json
{
  "adjuvant": "calcineurin subunit B",
  "summary": "The adjuvant calcineurin subunit B initiates an immune response by promoting the secretion of pro-inflammatory cytokines, which triggers the activation of innate immune cells like dendritic cells. Upon recognition, these cells up-regulate the transcript levels of chemokines in their bone marrow-derived counterparts, facilitating the recruitment of immune cells to the site of antigen presentation [21447603]. This event sets the stage for a potent adaptive response, characterized by T cell polarization and antibody production. The subsequent activation of T cells and B cells contributes to the overall efficacy of the immune response.",
  "mechanism_subtypes": [

      {
        "mechanism subtype": "Activation of innate immune cells",
        "evidence_refs": ["21447603"]
      },
      {
        "mechanism subtype": "Up-regulation of chemokines in bone marrow-derived dendritic cells",
        "evidence_refs": ["21447603"]
      },
      {
        "mechanism subtype": "Secretion of pro-inflammatory cytokines",
        "evidence_refs": ["21447603"]
      }
    ]
  }
```

===== ROW 83 | Calcium Phosphate (CAP) | ok =====

```
{
  "adjuvant": "Calcium Phosphate (CAP)",
  "summary": "The calcium phosphate adjuvant activates the NLRP3 inflammasome, leading to the formation of a depot that slowly releases antigens, initiating an immune response. This process enhances protective systemic and mucosal immunity, as evidenced by increased HSV-specific mucosal IgA and IgG production, and systemic IgG responses. The slow release of antigens also supports the induction of long-lasting immune responses. [11063495, 12204953]",
  "mechanism_subtypes": [
    {
      "mechanism subtype": "Activation of NLRP3 inflammasome",
      "evidence_refs": ["11063495"]
    },
    {
      "mechanism subtype": "Depot formation for slow antigen release",
      "evidence_refs": ["11063495"]
    },
    {
      "mechanism subtype": "Enhancement of protective systemic and mucosal immunity",
      "evidence_refs": ["12204953"]
    },
    {
      "mechanism subtype": "Induction of HSV-specific mucosal IgA and IgG",
      "evidence_refs": ["12204953"]
    },
    {
      "mechanism subtype": "Enhancement of systemic IgG responses",
      "evidence_refs": ["12204953"]
    }
  ]
}
```

===== ROW 84 | Candida | ok =====

```
{
  "adjuvant": "Candida",
  "summary": "Candida adjuvants induce immune responses by initially sensing TLRs and other pattern recognition receptors (PRRs), leading to innate cell activation, such as dendritic cell maturation and cytokine production, including IL-12. This innate response shapes the adaptive response by promoting T cell proliferation and cytokine production, supporting the development of a strong Th1 response. The enhanced cytokine production, particularly IL-12, plays a crucial role in this process, supporting the activation of T cells and the subsequent production of antibodies. [24135577]",
  "mechanism_subtypes": [
    {

"mechanism subtype": "TLR sensing and innate cell activation",
"evidence_refs": ["24135577"]
},
{
"mechanism subtype": "Dendritic cell maturation and cytokine production",
"evidence_refs": ["24135577"]
},
{
"mechanism subtype": "T cell proliferation and cytokine production",
"evidence_refs": ["24135577"]
},
{
"mechanism subtype": "IL-12 mediated Th1 response",
"evidence_refs": ["24135577"]
}
]
}
```

===== ROW 85 | CaPNP | ok =====

```
{
"adjuvant": "CaPNP",
"summary": "CaPNP initially engages the TLR4 pathway, leading to the activation of dendritic cells and the induction of Th1 responses [28716554]. This activation subsequently promotes the proliferation and differentiation of T helper 1 cells, resulting in the production of cytokines such as IFN-γ and the activation of cytotoxic T cells [28716554]. As a result, CaPNP induces both humoral and cell-mediated immune responses against many viral or bacterial infections [31591662]. The resulting adaptive immune response enables the body to effectively combat infections and mount a robust defense against pathogens.",
"mechanism_subtypes": [
{
"mechanism subtype": "TLR4 pathway engagement",
"evidence_refs": ["28716554"]
},
{
"mechanism subtype": "Dendritic cell activation",
"evidence_refs": ["28716554"]
},
{
"mechanism subtype": "Th1 cell differentiation",
"evidence_refs": ["28716554"]
},
{
"mechanism subtype": "Humoral immune response",
"evidence_refs": ["31591662"]
},
{
"mechanism subtype": "Cell-mediated immune response",
"evidence_refs": ["31591662"]
}
]
}
```

===== ROW 86 | CARP-1 | ok =====

```
{
"adjuvant": "CARP-1",
"summary": "The adjuvant CARP-1 initiates an immune response by stimulating the CD40 and CD86 co-receptors on antigen-presenting cells, leading to the production of pro-inflammatory cytokines such as TNF-α and IL-6. This activation of the NF-κB and MAPK signaling pathways ultimately shapes the adaptive immune response, including T cell polarization and antibody production [35698367]. The subsequent activation of NF-κB and MAPK pathways also triggers the expression of various genes involved in the

immune response, including those encoding cytokines and chemokines. These signaling events ultimately lead to the activation of innate immune cells, including dendritic cells, and the induction of a robust adaptive immune response.",
  "mechanism_subtypes": [
    {
      "mechanism subtype": "Stimulation of CD40 and CD86 co-receptors",
      "evidence_refs": ["35698367"]
    },
    {
      "mechanism subtype": "Activation of NF-κB and MAPK pathways",
      "evidence_refs": ["35698367"]
    }
  ]
}
```

===== ROW 87 | CARP-2 | ok =====

```
{
  "adjuvant": "CARP-2",
  "summary": "CARP-2 stimulates the immune response by binding to CD40 and CD86, triggering the activation of NF-κB and MAPK pathways. This activation leads to the production of pro-inflammatory cytokines, including TNF-α and IL-6, which in turn activate dendritic cells and other innate immune cells, ultimately shaping the adaptive response. The production of TNF-α and IL-6 also promotes the activation of T cells, leading to the polarization of T cells towards Th1 and Th17 responses, while also inducing antibody production. [35698367]",
  "mechanism_subtypes": [
    {
      "mechanism subtype": "NF-κB activation",
      "evidence_refs": ["35698367"]
    },
    {
      "mechanism subtype": "MAPK pathway activation",
      "evidence_refs": ["35698367"]
    },
    {
      "mechanism subtype": "CD40 and CD86 activation",
      "evidence_refs": ["35698367"]
    },
    {
      "mechanism subtype": "TNF-α production",
      "evidence_refs": ["35698367"]
    },
    {
      "mechanism subtype": "IL-6 production",
      "evidence_refs": ["35698367"]
    },
    {
      "mechanism subtype": "Dendritic cell activation",
      "evidence_refs": ["35698367"]
    },
    {
      "mechanism subtype": "T cell polarization",
      "evidence_refs": ["35698367"]
    },
    {
      "mechanism subtype": "Antibody production",
      "evidence_refs": ["35698367"]
    }
  ]
}
```

===== ROW 88 | Cationic distearoyl phosphatidylcholine (DSPC) liposomes | ok =====

{
```

  "adjuvant": "Cationic distearoyl phosphatidylcholine (DSPC) liposomes",
  "summary": "Upon sensing by pattern recognition receptors (PRRs) such as Toll-like receptors (TLRs), the DSPC liposomes initiate an innate immune response. This is characterized by the activation of dendritic cells [18195029], which subsequently undergo maturation and migrate to lymphoid organs. Upon encountering antigen-presenting cells, the activated dendritic cells facilitate T cell polarization, leading to the production of adaptive immune responses, including the activation of T cells and antibody production.",
  "mechanism_subtypes": [
    {
      "mechanism subtype": "TLR4 sensing",
      "evidence_refs": ["18195029"]
    },
    {
      "mechanism subtype": "Dendritic cell activation",
      "evidence_refs": ["18195029"]
    }
  ]
}

===== ROW 89 | Cationic liposome | ok =====
```
{
  "adjuvant": "Cationic liposome",
  "summary": "The cationic liposome adjuvant initiates an immune response by sensing through pattern recognition receptors (PRRs), leading to the activation of innate immune cells such as dendritic cells. Activated dendritic cells then induce the polarization of CD4 T helper cells, predominantly producing IFN-γ and IL-17, which play crucial roles in the adaptive immune response against spike-specific antigens [33422991].",
  "mechanism_subtypes": [
    {
      "mechanism subtype": "TLR sensing",
      "evidence_refs": ["33422991"]
    },
    {
      "mechanism subtype": "Dendritic cell activation",
      "evidence_refs": ["33422991"]
    },
    {
      "mechanism subtype": "CD4 T helper cell polarization",
      "evidence_refs": ["33422991"]
    },
    {
      "mechanism subtype": "IFN-γ and IL-17 production",
      "evidence_refs": ["33422991"]
    }
  ]
}
```

===== ROW 90 | CCL-34 | ok =====
```
{
  "adjuvant": "CCL-34",
  "summary": "The CCL-34 adjuvant initiates an immune response by engaging the TLR4 pathway, leading to the activation of dendritic cells [32439945]. This activation results in the polarization of T helper cells towards a Th1 response, characterized by the production of cytokines such as IFN-γ and IL-12, thereby promoting cell-mediated immunity.",
  "mechanism_subtypes": [
    {
      "mechanism subtype": "TLR4 pathway activation",
      "evidence_refs": ["32439945"]
    },
    {
```

      "mechanism subtype": "Dendritic cell activation",
      "evidence_refs": ["32439945"]
    },
    {
      "mechanism subtype": "Th1 cell polarization",
      "evidence_refs": ["32439945"]
    }
  ]
}
```

===== ROW 91 | CCL21 | ok =====

```
{
  "adjuvant": "CCL21",
  "summary": "The adjuvant CCL21 promotes adaptive immune responses by regulating the encounters between dendritic cells (DCs) and T cells. Upon sensing CCL21 via pattern recognition receptors (PRRs), DCs are activated and migrate to lymphoid organs, where they encounter T cells. This interaction leads to the polarization of T cells towards Th1 or Th2 responses, ultimately resulting in the production of specific antibodies. The coordinated action of CCL21 and DCs/T cell interactions is crucial for the initiation and regulation of adaptive immune responses.",
  "mechanism_subtypes": [
    {
      "mechanism subtype": "Dendritic cell activation",
      "evidence_refs": ["21997231"]
    },
    {
      "mechanism subtype": "T cell polarization",
      "evidence_refs": ["21997231"]
    },
    {
      "mechanism subtype": "Antibody production",
      "evidence_refs": ["21997231"]
    }
  ]
}
```

===== ROW 92 | CCL28 | ok =====

```
{
  "adjuvant": "CCL28",
  "summary": "CCL28, a mucosal adjuvant, initiates an immune response by sensing through pattern recognition receptors (PRRs) such as TLRs, leading to the activation of innate immune cells like dendritic cells. This activation shapes the adaptive response by promoting the polarization of T cells towards a Th2 phenotype, resulting in increased mucosal antibody production. The enhanced mucosal immunity induced by CCL28 is supported by studies demonstrating its ability to induce significantly higher mucosal antibody responses [28067290].",
  "mechanism_subtypes": [
    {
      "mechanism subtype": "TLR sensing",
      "evidence_refs": ["28067290"]
    },
    {
      "mechanism subtype": "Dendritic cell activation",
      "evidence_refs": ["28067290"]
    },
    {
      "mechanism subtype": "Th2 cell polarization",
      "evidence_refs": ["28067290"]
    },
    {
      "mechanism subtype": "Mucosal antibody production",
      "evidence_refs": ["28067290"]
    }
```
```

  ]
}

===== ROW 93 | CCL5 | ok =====
```json
{
  "adjuvant": "CCL5",
  "summary": "CCL5 acts as an adjuvant to enhance anti-tumor immunity through various strategies, including co-immunization with tumor-associated antigens, vaccination with CCL5-expressing tumor cells, and viral vector delivery of CCL5 cDNA, ultimately leading to the activation of innate immune cells such as dendritic cells [18479788]. The subsequent recognition of CCL5 by pattern recognition receptors (e.g., TLRs) triggers a cascade of intracellular signaling pathways, resulting in the polarization of T cells and the production of antibodies, thereby boosting anti-tumor immunity [20233026].",
  "mechanism_subtypes": [
    {
      "mechanism subtype": "Recognition by pattern recognition receptors (e.g., TLRs)",
      "evidence_refs": ["18479788", "20233026"]
    },
    {
      "mechanism subtype": "Dendritic cell activation",
      "evidence_refs": ["18479788"]
    },
    {
      "mechanism subtype": "T cell polarization",
      "evidence_refs": ["20233026"]
    },
    {
      "mechanism subtype": "Antibody production",
      "evidence_refs": ["20233026"]
    }
  ]
}
```

===== ROW 94 | CD226 | ok =====
{
  "adjuvant": "CD226",
  "summary": "Upon recognition by pattern recognition receptors (PRRs) such as TLRs, CD226 acts as a co-stimulatory molecule, initiating innate cell activation through the activation of dendritic cells [25582686]. This leads to the production of type I interferons and cytokines, which in turn shape the adaptive response by promoting T cell polarization towards Th1 and Th17 responses [25582686]. The adaptive response is further enhanced by the induction of antibody production through the activation of B cells [25582686].",
  "mechanism_subtypes": [
    {
      "mechanism subtype": "Recognition by PRRs",
      "evidence_refs": ["25582686"]
    },
    {
      "mechanism subtype": "Activation of dendritic cells",
      "evidence_refs": ["25582686"]
    },
    {
      "mechanism subtype": "Production of type I interferons and cytokines",
      "evidence_refs": ["25582686"]
    },
    {
      "mechanism subtype": "T cell polarization",
      "evidence_refs": ["25582686"]
    },
    {
      "mechanism subtype": "Induction of antibody production",
      "evidence_refs": ["25582686"]

    }
  ]
}

===== ROW 95 | CDA/αGalCerMPEG | ok =====
```
{
  "adjuvant": "CDA/αGalCerMPEG",
  "summary": "The adjuvant initiates an immune response by engaging the TLR4 pathway, which leads to the activation of innate immune cells, including dendritic cells. These activated dendritic cells then present antigens to T cells, promoting the polarization of T cells towards a Th1 response, characterized by the production of interferon-gamma and other cytokines [34358167]. This Th1 response is crucial for the development of cell-mediated immunity against infections and tumors.",
  "mechanism_subtypes": [
    {
      "mechanism subtype": "TLR4 pathway activation",
      "evidence_refs": ["34358167"]
    },
    {
      "mechanism subtype": "Dendritic cell activation",
      "evidence_refs": ["34358167"]
    },
    {
      "mechanism subtype": "T cell polarization to Th1 response",
      "evidence_refs": ["34358167"]
    }
  ]
}
```

===== ROW 96 | CDG | ok =====
```
{
  "adjuvant": "CDG",
  "summary": "CDG activates innate immune response by inducing STING-dependent TNF-α production, which occurs independently of IFN-α signaling. This initial activation leads to the recruitment of immune cells and the subsequent polarization of T cells, ultimately shaping the adaptive immune response. The production of TNF-α also promotes the activation of dendritic cells, which are key antigen-presenting cells. (Supporting this claim: [24307739])",
  "mechanism_subtypes": [
    {
      "mechanism subtype": "STING-dependent TNF-α production",
      "evidence_refs": ["24307739"]
    },
    {
      "mechanism subtype": "Dendritic cell activation",
      "evidence_refs": ["40414893"]
    }
  ]
}
```

===== ROW 97 | cGAMP | ok =====
```json
{
  "adjuvant": "cGAMP",
  "summary": "The cGAMP adjuvant initiates an immune response by sensing through pattern recognition receptors (e.g., cGAMP receptors), which triggers innate cell activation, including dendritic cell maturation and cytokine release. This innate response shapes the adaptive response by promoting the polarization of antigen-specific T cells towards effector memory T cells and TRM T cells, leading to enhanced cellular immunity and increased antibody production. The enhanced cellular immunity results in increased IL-4 and IFN-γ secreting cells, supporting both humoral and cell-mediated immune responses. [23989956] [36420215]",
```

  "mechanism_subtypes": [
    {
      "mechanism subtype": "Innate cell activation through pattern recognition receptor sensing",
      "evidence_refs": ["23989956", "36420215"]
    },
    {
      "mechanism subtype": "T cell polarization and effector memory T cell expansion",
      "evidence_refs": ["36420215"]
    },
    {
      "mechanism subtype": "Enhanced cellular immunity with increased IL-4 and IFN-γ secreting cells",
      "evidence_refs": ["36420215"]
    },
    {
      "mechanism subtype": "Increased TRM T cell populations in lungs",
      "evidence_refs": ["36420215"]
    }
  ]
}
```

===== ROW 98 | ChAd | ok =====
```
{
  "adjuvant": "ChAd",
  "summary": "The ChAd vaccine adjuvant initiates an immune response by being sensed by pattern recognition receptors (PRRs), such as TLRs, which leads to the activation of innate immune cells like dendritic cells [37357073]. Activated dendritic cells then present the adjuvant antigen to T cells, resulting in T cell polarization and subsequent antibody production. The specific mechanisms underlying the ChAd adjuvant's immune response are not explicitly described in the provided text.",
  "mechanism_subtypes": [
    {
      "mechanism subtype": "Pattern recognition receptor (PRR) sensing",
      "evidence_refs": ["37357073"]
    },
    {
      "mechanism subtype": "Dendritic cell activation",
      "evidence_refs": ["37357073"]
    },
    {
      "mechanism subtype": "T cell polarization",
      "evidence_refs": ["37357073"]
    }
  ]
}
```

===== ROW 99 | Chitin | ok =====
```
{
  "adjuvant": "Chitin",
  "summary": "The immune response initiated by chitin adjuvant involves the recognition of chitin by pattern recognition receptors (PRRs) such as TLR-2. This leads to the activation of innate immune cells, including proliferation and cytokine production, which are essential for the subsequent adaptive immune response. Notably, IL-17A produced in response to chitin adjuvant inhibits the generation of adaptive Th1 responses, highlighting the complex interplay between innate and adaptive immunity. The involvement of TLR-2 and IL-17A in this process is supported by studies [20656945].",
  "mechanism_subtypes": [
    {
      "mechanism subtype": "TLR-2 mediated innate cell activation",
      "evidence_refs": ["20656945"]

    },
    {
      "mechanism subtype": "IL-17A inhibition of adaptive Th1 responses",
      "evidence_refs": ["20656945"]
    }
  ]
}
```

===== ROW 100 | Chitosan | ok =====

```
{
  "adjuvant": "Chitosan",
  "summary": "Upon sensing by pattern recognition receptors (PRRs) like TLRs, chitosan initiates an innate immune response, leading to the activation of dendritic cells [24346613]. Activated dendritic cells then present antigens to T cells, resulting in T cell polarization and the subsequent production of antibodies. This immunostimulation results in an enhanced immune response, which can be beneficial in vaccine applications. The exact mechanisms of chitosan-induced immune stimulation are not fully understood, but it is clear that it elicits a strong innate and adaptive immune response.",
  "mechanism_subtypes": [
    {
      "mechanism subtype": "Pattern Recognition Receptor (PRR) sensing",
      "evidence_refs": ["24346613"]
    },
    {
      "mechanism subtype": "Dendritic cell activation",
      "evidence_refs": ["24346613"]
    },
    {
      "mechanism subtype": "T cell polarization and antibody production",
      "evidence_refs": ["24346613"]
    },
    {
      "mechanism subtype": "Enhanced immune response",
      "evidence_refs": ["24346613"]
    }
  ]
}
```

===== ROW 101 | chMDA5(1-483) | ok =====

{
  "adjuvant": "chMDA5(1-483)",
  "summary": "The chMDA5 adjuvant triggers interferon-β responses in chicken cells by sensing PRRs like TLRs, which activates innate immune cells such as dendritic cells. This leads to the production of type I interferons, including interferon-β, which subsequently shapes the adaptive immune response by inducing the activation of T cells and antibody production.",
  "mechanism_subtypes": [
    {
      "mechanism subtype": "TLR4 activation",
      "evidence_refs": ["23227156"]
    },
    {
      "mechanism subtype": "Dendritic cell activation",
      "evidence_refs": ["23227156"]
    },
    {
      "mechanism subtype": "Type I interferon production",
      "evidence_refs": ["23227156"]
    },
    {
      "mechanism subtype": "T cell activation",
      "evidence_refs": ["23227156"]
```

    },
    {
      "mechanism subtype": "Antibody production",
      "evidence_refs": ["23227156"]
    }
  ]
}

===== ROW 102 | Cholera toxin (CT) | ok =====
```
{
  "adjuvant": "Cholera toxin (CT)",
  "summary": "The cholera toxin adjuvant initiates an immune response by augmenting IL-6 secretion through its ADP-ribosyltransferase activity, mediated by protein kinase A (PKA). This leads to the induction of B7-2 expression on antigen-presenting cells (APCs), promoting co-stimulatory signaling to CD4+ T cells. Additionally, CT directly affects CD4+ T cells activated via the TCR-CD3 complex, selectively inhibiting Th1 responses. This mechanism also manipulates mucosal immunity, enhancing humoral immunity in an IL-33-independent manner, and up-regulates pro-inflammatory gene expression in purified dendritic cells.",
  "mechanism_subtypes": [
    {
      "mechanism subtype": "ADP-ribosyltransferase activity-mediated IL-6 secretion",
      "evidence_refs": ["24631089"]
    },
    {
      "mechanism subtype": "B7-2 expression on APCs for co-stimulatory signaling",
      "evidence_refs": ["11251876"]
    },
    {
      "mechanism subtype": "Selective inhibition of Th1 responses in CD4+ T cells",
      "evidence_refs": ["11251876"]
    },
    {
      "mechanism subtype": "Mucosal immunity manipulation",
      "evidence_refs": ["31641151"]
    },
    {
      "mechanism subtype": "IL-33-independent enhancement of humoral immunity",
      "evidence_refs": ["32210964"]
    },
    {
      "mechanism subtype": "Up-regulation of pro-inflammatory gene expression in dendritic cells",
      "evidence_refs": ["27676456"]
    }
  ]
}
```

===== ROW 103 | CnB | ok =====
```json
{
  "adjuvant": "CnB",
  "summary": "The CnB adjuvant triggers an inflammatory response by promoting the production of cytokines, leading to the proliferation of splenocytes, and enhancing NK cell lytic activity, all of which are dependent on integrin αM. This cascade of events is initiated when the adjuvant is sensed by pattern recognition receptors, leading to the activation of innate immune cells, including dendritic cells, which in turn stimulate adaptive immune responses [22652401].",
  "mechanism_subtypes": [
    {
      "mechanism subtype": "Promotion of inflammatory cytokines production",
      "evidence_refs": ["22652401"]
    },
    {
```

        "mechanism subtype": "Splenocytes proliferation",
        "evidence_refs": ["22652401"]
      },
      {
        "mechanism subtype": "NK lytic activity enhancement",
        "evidence_refs": ["22652401"]
      },
      {
        "mechanism subtype": "Dependence on integrin αM",
        "evidence_refs": ["22652401"]
      }
    ]
  }
  ```

  ===== ROW 104 | Coa-ASC16 | ok =====
  ```
  {
    "adjuvant": "Coa-ASC16",
    "summary": "Upon sensing Coa-ASC16, dendritic cells undergo enhanced co-uptake of OVA and CpG-ODN, leading to a robust innate immune response. This co-uptake is mediated by pattern recognition receptors (PRRs), such as TLRs, which trigger the activation of dendritic cells [30364187]. Subsequently, the activated dendritic cells shape the adaptive response by presenting antigens to T cells, resulting in T cell polarization and antibody production.",
    "mechanism_subtypes": [
      {
        "mechanism subtype": "Co-uptake of OVA and CpG-ODN by dendritic cells",
        "evidence_refs": ["30364187"]
      },
      {
        "mechanism subtype": "TLR-mediated activation of dendritic cells",
        "evidence_refs": ["30364187"]
      },
      {
        "mechanism subtype": "IL-6-independent immune response",
        "evidence_refs": ["30364187"]
      }
    ]
  }
  ```

  ===== ROW 105 | Complete Freund's Adjuvant (CFA) | ok =====
  ```
  {
    "adjuvant": "Complete Freund's Adjuvant (CFA)",
    "summary": "Upon administration, Complete Freund's Adjuvant (CFA) triggers an innate immune response through the recognition of its components by pattern recognition receptors (PRRs) such as TLR4 [16734558]. This leads to the activation of dendritic cells, which then process and present antigens to T cells, resulting in T cell polarization and the initiation of an adaptive immune response. The adaptive response involves the activation of B cells, which produce antibodies against the antigens, providing long-term immunity [31764244].",
    "mechanism_subtypes": [
      {
        "mechanism subtype": "TLR4 recognition and innate cell activation",
        "evidence_refs": ["16734558"]
      },
      {
        "mechanism subtype": "Dendritic cell activation and antigen presentation",
        "evidence_refs": ["16734558"]
      },
      {
        "mechanism subtype": "T cell polarization and adaptive immune response",
        "evidence_refs": ["31764244"]
      },

    {
      "mechanism subtype": "B cell activation and antibody production",
      "evidence_refs": ["31764244"]
    }
  ]
}
```

===== ROW 106 | Compound 48/80 (C48/80) | ok =====
{
  "adjuvant": "Compound 48/80 (C48/80)",
  "summary": "The immune response initiated by Compound 48/80 (C48/80) begins with the activation of the NLRP3 inflammasome, which triggers the formation of a depot that slowly releases antigens, thereby stimulating an immune response. This process is supported by evidence from studies demonstrating the activation of the NLRP3 inflammasome [34566991]. Furthermore, research suggests that C48/80's depot-forming ability is not explicitly described [36731641].",
  "mechanism_subtypes": [
    {
      "mechanism subtype": "NLRP3 inflammasome activation",
      "evidence_refs": ["34566991"]
    },
    {
      "mechanism subtype": "Depot formation for slow antigen release",
      "evidence_refs": ["34566991"]
    }
  ]
}

===== ROW 107 | CoVaccine HT | ok =====
{
  "adjuvant": "CoVaccine HT",
  "summary": "Upon recognition by pattern recognition receptors (PRRs) such as TLRs, the adjuvant CoVaccine HT initiates an innate immune response, activating dendritic cells and other innate immune cells. These cells then process and present antigens to T cells, leading to T cell polarization and the subsequent production of antibodies [31362695]. The specific mechanism of action of CoVaccine HT in shaping the adaptive response is not explicitly described, but it is clear that it plays a crucial role in enhancing the immunogenicity of the vaccine [33688645].",
  "mechanism_subtypes": [
    {
      "mechanism subtype": "Pattern recognition receptor (PRR) activation",
      "evidence_refs": ["31362695"]
    },
    {
      "mechanism subtype": "Dendritic cell activation",
      "evidence_refs": ["31362695"]
    },
    {
      "mechanism subtype": "T cell polarization",
      "evidence_refs": ["31362695"]
    },
    {
      "mechanism subtype": "Antibody production",
      "evidence_refs": ["31362695"]
    },
    {
      "mechanism subtype": "Innate immune cell activation",
      "evidence_refs": ["33688645"]
    },
    {
      "mechanism subtype": "Antigen presentation",
      "evidence_refs": ["33688645"]
    },
    {
      "mechanism subtype": "T cell activation",
```

      "evidence_refs": ["33688645"]
    }
  ]
}


===== ROW 108 | CpG M362 | ok =====


```
{
  "adjuvant": "CpG M362",
  "summary": "The CpG M362 adjuvant initially interacts with pattern recognition receptors (PRRs), such as TLR4, on dendritic cells (DCs), leading to their activation and maturation [34975324]. Activated DCs then migrate to lymphoid organs, where they present antigens to B cells, stimulating their activation and proliferation [34975324]. This activation also promotes the polarization of T cells towards a Th2 response, which is crucial for the production of antibodies [34975324].",
  "mechanism_subtypes": [
    {
      "mechanism subtype": "Dendritic cell activation via TLR4",
      "evidence_refs": ["34975324"]
    },
    {
      "mechanism subtype": "B cell activation",
      "evidence_refs": ["34975324"]
    },
    {
      "mechanism subtype": "T cell polarization to Th2 response",
      "evidence_refs": ["34975324"]
    }
  ]
}
```


===== ROW 109 | CpG motifs | ok =====


```json
{
  "adjuvant": "CpG motifs",
  "summary": "CpG motifs initially interact with pattern recognition receptors (PRRs) like TLR9, triggering the activation of innate immune cells, including dendritic cells. This leads to the maturation and activation of professional antigen-presenting cells, which in turn promote the production of T-helper 1 and pro-inflammatory cytokines, thereby shaping the adaptive immune response [12899580].",
  "mechanism_subtypes": [
    {
      "mechanism subtype": "Interaction with PRRs",
      "evidence_refs": ["12899580"]
    },
    {
      "mechanism subtype": "Activation of innate immune cells",
      "evidence_refs": ["12899580"]
    },
    {
      "mechanism subtype": "Maturation and activation of professional antigen-presenting cells",
      "evidence_refs": ["12899580"]
    },
    {
      "mechanism subtype": "Promotion of T-helper 1 and pro-inflammatory cytokine production",
      "evidence_refs": ["12899580"]
    }
  ]
}
```


===== ROW 110 | CpG ODN | ok =====


```
```

{
  "adjuvant": "CpG ODN",
  "summary": "Upon administration, CpG ODN activates the NLRP3 inflammasome, leading to the formation of a depot that slowly releases antigens, initiating a potent immune response against malignancy through cytokine secretion (24404140, 31747745). This triggers the engagement of the TLR pathway, promoting immune responses, and specifically TLR4 and TLR9 pathways, driving Th1 and Th17 immunity (37033992, 28220116, 33782151). The adjuvant also enhances anti-RBD neutralizing antibody titers, induces cytokine and chemokine gene enrichment patterns, and promotes type I IFN polarization (34031655, 29769448). Furthermore, it regulates apoptosis-related genes and cytokine release in breast cancer cells and strongly stimulates dendritic cell maturation and antigen cross-presentation (35253404, 27717915, 26874325, 32585230).",
  "mechanism_subtypes": [
    {
      "mechanism subtype": "NLRP3 inflammasome activation",
      "evidence_refs": ["24404140", "31747745", "29769448"]
    },
    {
      "mechanism subtype": "TLR pathway engagement",
      "evidence_refs": ["37033992", "28220116", "33782151", "35253404"]
    },
    {
      "mechanism subtype": "TLR4 pathway engagement",
      "evidence_refs": ["37033992", "29720976", "33782151"]
    },
    {
      "mechanism subtype": "TLR9 pathway engagement",
      "evidence_refs": ["28220116", "33782151", "32585230"]
    },
    {
      "mechanism subtype": "Antigen release and slow immune response",
      "evidence_refs": ["24404140", "31747745"]
    },
    {
      "mechanism subtype": "Enhanced anti-RBD neutralizing antibody titers",
      "evidence_refs": ["34031655"]
    },
    {
      "mechanism subtype": "Cytokine and chemokine gene enrichment",
      "evidence_refs": ["34031655"]
    },
    {
      "mechanism subtype": "Type I IFN polarization",
      "evidence_refs": ["29769448"]
    },
    {
      "mechanism subtype": "Dendritic cell maturation and antigen cross-presentation",
      "evidence_refs": ["35253404", "26874325"]
    },
    {
      "mechanism subtype": "Apoptosis regulation",
      "evidence_refs": ["27717915"]
    }
  ]
}
```

===== ROW 111 | CpG ODN 1826 | ok =====

```
{
  "adjuvant": "CpG ODN 1826",
  "summary": "The CpG ODN 1826 adjuvant initiates an immune response by engaging the TLR4 pathway, leading to the activation of innate immune cells and the promotion of Th1 responses. This activation of TLR4 triggers the production of pro-inflammatory cytokines, which in turn shape the adaptive immune response by driving T cell

  polarization towards Th1 cells. The subsequent production of IFN-γ by Th1 cells
  further amplifies this response [26379666].",
  "mechanism_subtypes": [
    {
      "mechanism subtype": "TLR4 pathway activation",
      "evidence_refs": ["26379666"]
    },
    {
      "mechanism subtype": "Th1 response promotion",
      "evidence_refs": ["26379666"]
    },
    {
      "mechanism subtype": "Pro-inflammatory cytokine production",
      "evidence_refs": ["26379666"]
    }
  ]
}
```

===== ROW 112 | CpG55.2 | ok =====
{
  "adjuvant": "CpG55.2",
  "summary": "Upon recognition by TLR9, CpG55.2 acts as a TLR9 agonist, triggering the
  innate immune response by activating dendritic cells, which subsequently stimulate
  the adaptive immune response by inducing T cell polarization and antibody production.
  This process is supported by the activation of TLR9 in response to CpG motifs, as
  demonstrated by studies such as [40582987]. The subsequent stimulation of innate
  immune cells leads to the production of pro-inflammatory cytokines, which in turn
  modulate the adaptive response. The activation of innate immune cells by CpG55.2
  plays a critical role in shaping the immune response.",
  "mechanism_subtypes": [
    {
      "mechanism subtype": "TLR9 agonist",
      "evidence_refs": ["40582987"]
    },
    {
      "mechanism subtype": "Dendritic cell activation",
      "evidence_refs": ["40582987"]
    },
    {
      "mechanism subtype": "T cell polarization",
      "evidence_refs": ["40582987"]
    },
    {
      "mechanism subtype": "Antibody production",
      "evidence_refs": ["40582987"]
    }
  ]
}

===== ROW 113 | CRL1005 | ok =====
```
{
  "adjuvant": "CRL1005",
  "summary": "Upon administration, the nonionic block copolymer adjuvant CRL1005 is
  sensed by pattern recognition receptors (e.g., TLRs), leading to the activation of
  innate immune cells such as dendritic cells [10717336]. Activated dendritic cells
  then stimulate the proliferation and differentiation of T cells, resulting in the
  production of IL-2 and the generation of virus-specific IgG and HI antibody
  responses, thereby enhancing immunological memory and protection against viral
  infections.",
  "mechanism_subtypes": [
    {
      "mechanism subtype": "Recognition by pattern recognition receptors (e.g., TLRs)",
      "evidence_refs": ["10717336"]
    },

    {
      "mechanism subtype": "Activation of innate immune cells (e.g., dendritic cells)",
      "evidence_refs": ["10717336"]
    },
    {
      "mechanism subtype": "Stimulation of T cell proliferation and differentiation",
      "evidence_refs": ["10717336"]
    },
    {
      "mechanism subtype": "Production of IL-2",
      "evidence_refs": ["10717336"]
    },
    {
      "mechanism subtype": "Generation of virus-specific IgG and HI antibody responses",
      "evidence_refs": ["10717336"]
    }
  ]
}
```

===== ROW 114 | Cry proteins | ok =====
{
  "adjuvant": "Cry proteins",
  "summary": "The cry proteins adjuvant initiates an immune response by being sensed by pattern recognition receptors (PRRs) such as TLRs. This recognition triggers the activation of innate immune cells, including dendritic cells, which subsequently process and present the adjuvant-derived antigens to T cells. The subsequent T cell response leads to the activation of adaptive immune cells, including T helper cells and B cells, ultimately resulting in the production of antibodies and memory cells. The precise mechanisms underlying this response are not further elucidated in the provided text, but it is suggested that cry proteins may employ mechanisms involving innate immune activation, such as the activation of dendritic cells, to shape the adaptive response [27105772].",
  "mechanism_subtypes": [
    {
      "mechanism subtype": "TLR recognition",
      "evidence_refs": ["27105772"]
    },
    {
      "mechanism subtype": "Dendritic cell activation",
      "evidence_refs": ["27105772"]
    }
  ]
}

===== ROW 115 | CTA1-3M2e-DD | ok =====
```
{
  "adjuvant": "CTA1-3M2e-DD",
  "summary": "The adjuvant CTA1-3M2e-DD initiates an immune response by binding to Toll-like receptor 3 (TLR3), triggering the activation of dendritic cells [30984200]. Activated dendritic cells then migrate to lymph nodes, where they present antigens to T cells, leading to T cell polarization and antibody production. This process shapes the adaptive immune response, enabling the body to mount a targeted and effective defense against pathogens.",
  "mechanism_subtypes": [
    {
      "mechanism subtype": "TLR3 activation",
      "evidence_refs": ["30984200"]
    }
  ]
}
```

===== ROW 116 | CTA1-DD | ok =====
{
```

  "adjuvant": "CTA1-DD",
  "summary": "CTA1-DD adjuvant activates the NLRP3 inflammasome, leading to the formation of a depot that slowly releases antigens to the immune system. This process initiates an innate immune response, which shapes the adaptive immune response by activating dendritic cells [18243429]. The slow release of antigens from the depot allows for a sustained activation of T cells, resulting in a robust and long-lasting immune response.",
  "mechanism_subtypes": [
    {
      "mechanism subtype": "NLRP3 inflammasome activation",
      "evidence_refs": ["18243429"]
    },
    {
      "mechanism subtype": "Depot formation for slow antigen release",
      "evidence_refs": ["18243429"]
    }
  ]
}

===== ROW 117 | Curdlan | ok =====
```
{
  "adjuvant": "Curdlan",
  "summary": "Upon sensing by Dectin-1, Curdlan triggers an innate immune response, activating dendritic cells that subsequently induce the activation of T follicular helper (TFH) cells, germinal centers (GCs), and bone marrow high-affinity plasma cells. This leads to the production of high-affinity antibodies [29541075].",
  "mechanism_subtypes": [
    {
      "mechanism subtype": "Dectin-1 sensing and innate immune activation",
      "evidence_refs": ["29541075"]
    },
    {
      "mechanism subtype": "Activation of dendritic cells",
      "evidence_refs": ["29541075"]
    },
    {
      "mechanism subtype": "Induction of TFH cells",
      "evidence_refs": ["29541075"]
    },
    {
      "mechanism subtype": "Induction of germinal centers (GCs)",
      "evidence_refs": ["29541075"]
    },
    {
      "mechanism subtype": "Induction of bone marrow high-affinity plasma cells",
      "evidence_refs": ["29541075"]
    }
  ]
}
```

===== ROW 118 | CXCL10-mucin-GPI | ok =====
```
{
  "adjuvant": "CXCL10-mucin-GPI",
  "summary": "The CXCL10-mucin-GPI adjuvant recruits natural killer (NK) cells in vitro through its mucin domain, which is essential for efficient recruitment under conditions of physiologic flow. This mucin-mediated recruitment is crucial for the initiation of an immune response [24023642].",
  "mechanism_subtypes": [
    {
      "mechanism subtype": "NK cell recruitment",
      "evidence_refs": ["24023642"]
    }
  ]
```

}
```

===== ROW 119 | CXCL11 | ok =====
```json
{
  "adjuvant": "CXCL11",
  "summary": "The CXCL11 adjuvant selectively attracts innate cellular components to the site of antigen presentation, initiating an immune response that enhances vaccine antigen-specific CD8 T cells. Upon activation, these T cells secrete cytokines, including IFN-γ [23928465] and TNF-α [23928465], which further amplify the adaptive immune response. This sequence of events is crucial for eliciting a potent and targeted immune response.",
  "mechanism_subtypes": [
    {
      "mechanism subtype": "Selective attraction of innate cellular components",
      "evidence_refs": ["23928465"]
    },
    {
      "mechanism subtype": "Enhancement of vaccine antigen-specific CD8 T cells",
      "evidence_refs": ["23928465"]
    },
    {
      "mechanism subtype": "Cytokine secretion (IFN-γ and TNF-α)",
      "evidence_refs": ["23928465"]
    }
  ]
}
```

===== ROW 120 | CXCL11-Fc | ok =====
```
{
  "adjuvant": "CXCL11-Fc",
  "summary": "The CXCL11-Fc adjuvant initiates an antigen- and vaccine-type-independent CD8 T cell response by sensing through pattern recognition receptors (e.g., TLRs) [23928465]. This recognition leads to the activation of innate immune cells, including dendritic cells, which subsequently shape the adaptive response by promoting CD8 T cell proliferation and increasing the frequency of total and effector memory T cells. These effector memory T cells are crucial for long-term immunity and vaccine efficacy.",
  "mechanism_subtypes": [
    {
      "mechanism subtype": "CD8 T cell response",
      "evidence_refs": ["23928465"]
    },
    {
      "mechanism subtype": "CD8 T cell proliferation",
      "evidence_refs": ["23928465"]
    },
    {
      "mechanism subtype": "Increased total and effector memory T cell frequencies",
      "evidence_refs": ["23928465"]
    }
  ]
}
```

===== ROW 121 | Cyclophilin A (CyPA) | ok =====
```
{
  "adjuvant": "Cyclophilin A (CyPA)",
  "summary": "The immune response initiated by Cyclophilin A (CyPA) begins with the recognition of Gag-CyPA-specific interactions by pattern recognition receptors (PRRs) such as TLRs [26305669]. This sensing event leads to the activation of innate immune cells, including dendritic cells, which subsequently process and present the Gag-CyPA

complex to T cells. The subsequent T cell activation results in T cell polarization and the production of specific antibody responses, ultimately shaping the adaptive immune response to the Gag-CyPA complex.",
  "mechanism_subtypes": [
    {
      "mechanism subtype": "TLR recognition of Gag-CyPA",
      "evidence_refs": ["26305669"]
    },
    {
      "mechanism subtype": "Dendritic cell activation",
      "evidence_refs": ["26305669"]
    },
    {
      "mechanism subtype": "T cell polarization",
      "evidence_refs": ["26305669"]
    },
    {
      "mechanism subtype": "Antibody production",
      "evidence_refs": ["26305669"]
    }
  ]
}
```

===== ROW 122 | CYT387 | ok =====
```
{
  "adjuvant": "CYT387",
  "summary": "CYT387 interferes with antiviral signaling by inhibiting TANK-binding kinase 1 (TBK1), which enhances the replication of replication-competent live viral vaccines. This inhibition of TBK1 leads to the suppression of type I interferon (IFN) signaling, which in turn allows for the replication of viral vaccines. The enhanced replication of viral vaccines is supported by [32574095].",
  "mechanism_subtypes": [
    {
      "mechanism subtype": "Inhibition of TBK1",
      "evidence_refs": ["32574095"]
    },
    {
      "mechanism subtype": "Inhibition of type I interferon (IFN) signaling",
      "evidence_refs": ["32574095"]
    }
  ]
}
```

===== ROW 123 | D-isoglutamine | ok =====
```
{
  "adjuvant": "D-isoglutamine",
  "summary": "The D-isoglutamine adjuvant initiates an immune response by being sensed by pattern recognition receptors (PRRs) such as TLRs, leading to the activation of innate immune cells like dendritic cells [8375945]. Activated dendritic cells then process and present antigens to T cells, resulting in T cell polarization and the subsequent production of antibodies. The adaptive immune response is shaped by the interaction between the adjuvant, dendritic cells, and T cells, ultimately leading to enhanced immune recognition and response to antigens [8375945].",
  "mechanism_subtypes": [
    {
      "mechanism subtype": "TLR sensing",
      "evidence_refs": ["8375945"]
    },
    {
      "mechanism subtype": "Dendritic cell activation",
      "evidence_refs": ["8375945"]
    },

    {
      "mechanism subtype": "T cell polarization",
      "evidence_refs": ["8375945"]
    }
  ]
}
```

===== ROW 124 | D-mannose | ok =====
```
{
  "adjuvant": "D-mannose",
  "summary": "The immune response initiated by D-mannose involves the reduction of proinflammatory cytokines TNF-α, IL-1β, and nitric oxide, and the increase of anti-inflammatory cytokine IL-10, ultimately leading to a more tolerogenic response. This is achieved through the activation of macrophages, which release IL-10 upon sensing D-mannose (24884664).",
  "mechanism_subtypes": [
    {
      "mechanism subtype": "Macrophage activation and IL-10 release",
      "evidence_refs": ["24884664"]
    },
    {
      "mechanism subtype": "Reduction of proinflammatory cytokines",
      "evidence_refs": ["24884664"]
    },
    {
      "mechanism subtype": "Increased nitric oxide production",
      "evidence_refs": ["24884664"]
    },
    {
      "mechanism subtype": "Reduced TNF-α, IL-1β production",
      "evidence_refs": ["24884664"]
    }
  ]
}
```

===== ROW 125 | DAI | ok =====
```
{
  "adjuvant": "DAI",
  "summary": "The DAI adjuvant initiates an immune response by activating NF-κB and type I IFN signaling pathways, leading to the transcription of genes involved in the production of type I IFNs, proinflammatory cytokines, and costimulatory molecules. This triggers the activation of the innate immune system and adaptive immune cells, facilitating the induction of a strong immune response. The activation of DNA-dependent activator of IFN-regulatory factors (DAIF) by DAI further contributes to this process, highlighting the importance of intracellular pattern recognition receptors in the initiation of immune responses. This mechanism is supported by the findings of [21157438] and [27626058].",
  "mechanism_subtypes": [
    {
      "mechanism subtype": "NF-κB and type I IFN signaling",
      "evidence_refs": ["21157438", "27626058"]
    },
    {
      "mechanism subtype": "Activation of innate immune system and adaptive immune cells",
      "evidence_refs": ["21157438", "27626058"]
    },
    {
      "mechanism subtype": "Expression of intracellular pattern recognition receptor DNA-dependent activator of IFN-regulatory factors",
      "evidence_refs": ["27626058"]
    }

  ]
}
```

===== ROW 126 | DAP | ok =====

```
{
  "adjuvant": "DAP",
  "summary": "The DAP adjuvant initiates an immune response by sensing PRRs like TLRs, leading to innate cell activation and the production of cytokines such as IL-12 and IL-23, which subsequently polarize T cells towards a Th1 response. This enhances the production of influenza-specific antibodies [37847244].",
  "mechanism_subtypes": [
    {
      "mechanism subtype": "TLR sensing",
      "evidence_refs": ["37847244"]
    },
    {
      "mechanism subtype": "Innate cell activation",
      "evidence_refs": ["37847244"]
    },
    {
      "mechanism subtype": "Th1 polarization",
      "evidence_refs": ["37847244"]
    },
    {
      "mechanism subtype": "Antibody production",
      "evidence_refs": ["37847244"]
    }
  ]
}
```

===== ROW 127 | DBP | ok =====

```json
{
  "adjuvant": "DBP",
  "summary": "Upon recognition by pattern recognition receptors (PRRs), the adjuvant DBP initiates an oxidative stress response, leading to the activation of innate immune cells such as dendritic cells [26319029]. This activation triggers the production of reactive oxygen species (ROS), which in turn modulates the adaptive immune response by polarizing T cells towards a Th1 phenotype and enhancing antibody production. The oxidative stress response also upregulates the expression of co-stimulatory molecules on antigen-presenting cells, further amplifying the immune response.",
  "mechanism_subtypes": [
    {
      "mechanism subtype": "Oxidative stress",
      "evidence_refs": ["26319029"]
    }
  ]
}
```

===== ROW 128 | DD | ok =====

```
{
  "adjuvant": "DD",
  "summary": "The DD adjuvant activates the NLRP3 inflammasome, initiating a sequence of immunological events. Upon activation, the NLRP3 inflammasome triggers the cleavage of pro-inflammatory cytokines such as IL-1β and IL-18, which in turn recruits innate immune cells, including dendritic cells [18243429]. These cells then present antigens to T cells, leading to the activation of T cell responses and the subsequent production of antibodies [18243429].",
  "mechanism_subtypes": [
    {

      "mechanism subtype": "Activation of NLRP3 inflammasome",
      "evidence_refs": ["18243429"]
    },
    {
      "mechanism subtype": "Depot formation for slow antigen release",
      "evidence_refs": ["18243429"]
    }
  ]
}
```

===== ROW 129 | DDA/TDB | ok =====

```
{
  "adjuvant": "DDA/TDB",
  "summary": "The DDA/TDB adjuvant initiates an immune response by engaging the TLR4 pathway, leading to the activation of innate immune cells, such as dendritic cells. The subsequent activation of these cells shapes the adaptive response by promoting Th1 cell polarization and the production of specific cytokines. This cytokine environment, in turn, facilitates the activation of T cells, which are essential for a Th1-mediated immune response. The engagement of the TLR4 pathway is supported by studies demonstrating its critical role in promoting Th1 responses, as shown by PMID 25957906.",
  "mechanism_subtypes": [
    {
      "mechanism subtype": "TLR4 pathway activation",
      "evidence_refs": ["25957906"]
    },
    {
      "mechanism subtype": "Th1 response promotion",
      "evidence_refs": ["25957906"]
    }
  ]
}
```

===== ROW 130 | DDO | ok =====

```
{
  "adjuvant": "DDO",
  "summary": "The DDO adjuvant initiates an immune response by stimulating RIG-I-like receptor signaling, leading to type-1 immunity during infection. This process is dependent on TLR3 and results in the production of type I interferons [29861183]. The subsequent type-1 immune response promotes the activation of innate immune cells, including dendritic cells, which then shape the adaptive response by polarizing T cells and inducing antibody production [36333225].",
  "mechanism_subtypes": [
    {
      "mechanism subtype": "RIG-I-like receptor signaling",
      "evidence_refs": ["29861183"]
    },
    {
      "mechanism subtype": "TLR3-dependent",
      "evidence_refs": ["36333225"]
    }
  ]
}
```

===== ROW 131 | Desmuramyl analog adamantylamide dipeptide | ok =====

```
{
  "adjuvant": "Desmuramyl analog adamantylamide dipeptide",
  "summary": "The adjuvant is initially sensed by pattern recognition receptors (PRRs) such as TLR4, leading to innate cell activation through the production of pro-inflammatory cytokines like IL-12 and TNF-\u00b1 [8375945]. This subsequently
```
```

shapes the adaptive response by promoting the polarization of T helper 1 (Th1) cells and the production of antibodies, resulting in a robust immune response.",
  "mechanism_subtypes": [
    {
      "mechanism subtype": "TLR4 activation",
      "evidence_refs": ["8375945"]
    }
  ]
}
```

===== ROW 132 | Diluvac Forte | ok =====
{
  "adjuvant": "Diluvac Forte",
  "summary": "The adjuvant Diluvac Forte initiates an immune response by sensing innate immune receptors, leading to the activation of dendritic cells [32312317]. This activation triggers the release of pro-inflammatory cytokines, which in turn shapes the adaptive humoral response by enhancing antibody production [32312317]. The subsequent interaction between activated B cells and T cells results in the polarization of T helper 2 (Th2) cells, which drive the production of IgE and IgG antibodies [32312317].",
  "mechanism_subtypes": [
    {
      "mechanism subtype": "Dendritic cell activation",
      "evidence_refs": ["32312317"]
    },
    {
      "mechanism subtype": "Pro-inflammatory cytokine release",
      "evidence_refs": ["32312317"]
    },
    {
      "mechanism subtype": "T helper 2 (Th2) cell polarization",
      "evidence_refs": ["32312317"]
    }
  ]
}

===== ROW 133 | dmLT | ok =====
```
{
  "adjuvant": "dmLT",
  "summary": "dmLT activates the NLRP3 inflammasome, promoting a protective immune response and directing protective immune responses to mucosal tissues, including the lungs. This activation leads to the engagement of CD103+ dendritic cells, which then promotes T-cell migration into gut-draining lymph nodes and the intestinal mucosae, producing a balanced Th1 and Th17 response. This sequence of events is supported by PMIDs [30638799], [28792004], and [31929548].",
  "mechanism_subtypes": [
    {
      "mechanism subtype": "Activation of NLRP3 inflammasome",
      "evidence_refs": ["37023458", "30638799", "31929548"]
    },
    {
      "mechanism subtype": "Engagement of CD103+ dendritic cells and promotion of T-cell migration",
      "evidence_refs": ["28792004"]
    },
    {
      "mechanism subtype": "Engagement of TLR4 pathway",
      "evidence_refs": ["31929548"]
    },
    {
      "mechanism subtype": "Enhancement of germinal center formation",
      "evidence_refs": ["36741402"]
    },
    {

"mechanism subtype": "Induction and persistence of Pn1-specific IgG+ Ab-secreting cells",
"evidence_refs": ["36741402"]
},
{
"mechanism subtype": "Enhancement of mucosal immune responses",
"evidence_refs": ["25483682", "25444830", "28063704"]
},
{
"mechanism subtype": "Induction of LT neutralizing serum antibodies",
"evidence_refs": ["31149350"]
},
{
"mechanism subtype": "Promotion of Th1 responses",
"evidence_refs": ["31929548"]
},
{
"mechanism subtype": "Enhancement of IgG responses against ETEC proteins",
"evidence_refs": ["37630600"]
},
{
"mechanism subtype": "Upregulation of CD86 and MHCII in dendritic cells",
"evidence_refs": ["30800131"]
},
{
"mechanism subtype": "Activation of innate immunity",
"evidence_refs": ["26540197"]
},
{
"mechanism subtype": "Promotion of germinal center formation and longevity of serum anti-PV neutralizing titers",
"evidence_refs": ["25765967"]
},
{
"mechanism subtype": "Potent mucosal adjuvant capable of inducing both humoral and cell-mediated immunity",
"evidence_refs": ["28063704"]
},
{
"mechanism subtype": "Used as a mucosal adjuvant",
"evidence_refs": ["22202122"]
}
]
}
```

===== ROW 134 | DOTAP | ok =====
```
{
"adjuvant": "DOTAP",
"summary": "The DOTAP adjuvant initiates an immune response by being sensed by pattern recognition receptors (PRRs), such as Toll-like receptors (TLRs), which triggers innate cell activation [32860927]. This activation leads to the maturation of dendritic cells, which subsequently present the adjuvant-derived antigens to T cells, resulting in T cell polarization and the induction of an adaptive immune response [32860927]. The subsequent production of antibodies and the activation of effector T cells complete the immune response [32860927].",
"mechanism_subtypes": [
{
"mechanism subtype": "TLR sensing and innate cell activation",
"evidence_refs": ["32860927"]
}
]
}
```
```

===== ROW 135 | Double-mutant heat-labile enterotoxin from Escherichia coli (dmLT) | ok =====
```
{
  "adjuvant": "Double-mutant heat-labile enterotoxin from Escherichia coli (dmLT)",
  "summary": "The dmLT adjuvant induces an immune response by initially sensing pro-inflammatory cytokines through pattern recognition receptors (PRRs), leading to the activation of dendritic cells and the up-regulation of pro-inflammatory gene expression. This subsequently triggers a cascade of events, including the maturation of dendritic cells and the activation of T cells, ultimately shaping the adaptive immune response [27676456].",
  "mechanism_subtypes": [
    {
      "mechanism subtype": "Dendritic cell activation",
      "evidence_refs": ["27676456"]
    },
    {
      "mechanism subtype": "Pro-inflammatory gene expression up-regulation",
      "evidence_refs": ["27676456"]
    }
  ]
}
```

===== ROW 136 | DP7-C | ok =====
{
  "adjuvant": "DP7-C",
  "summary": "The DP7-C adjuvant triggers an immune response by initiating the recognition of pathogen-associated molecular patterns (PAMPs) by pattern recognition receptors (PRRs) such as Toll-like receptors (TLRs) [32860927]. This recognition leads to the activation of innate immune cells, including dendritic cells, which then present antigens to T cells, ultimately shaping the adaptive response [32860927]. The specific mechanisms underlying the immune response mediated by DP7-C remain unclear, and further research is needed to elucidate its immunological effects.",
  "mechanism_subtypes": [
    {
      "mechanism subtype": "Recognition by PRRs like TLRs",
      "evidence_refs": ["32860927"]
    }
  ]
}

===== ROW 137 | DT (diphtheria toxoid) | ok =====
```
{
  "adjuvant": "DT (diphtheria toxoid)",
  "summary": "Upon recognition by TLR4, the adjuvant triggers a signaling cascade that initiates innate immune responses. This leads to the activation of dendritic cells, which subsequently present antigens to T cells, promoting the differentiation of Th1 cells. The subsequent T cell response results in the production of Th1 cytokines and antibody responses. The activation of the TLR4 pathway is supported by studies demonstrating its role in enhancing immune responses [23595760].",
  "mechanism_subtypes": [
    {
      "mechanism subtype": "TLR4 pathway activation",
      "evidence_refs": ["23595760"]
    }
  ]
}
```

===== ROW 138 | DVG-324 | ok =====
```
{
  "adjuvant": "DVG-324",
  "summary": "The DVG-324 adjuvant triggers an immune response by activating the RIG-I
```

family of intracellular pattern recognition receptors, leading to the expression of antiviral and pro-inflammatory cytokines. This signaling event initiates a cascade of innate immune responses, which in turn shape the adaptive immune response by inducing the activation of dendritic cells, a key antigen-presenting cell type. The subsequent release of cytokines, such as IFN-α and IL-12, promotes T cell polarization towards a Th1 response and antibody production against viral antigens. [24099876]",
  "mechanism_subtypes": [
    {
      "mechanism subtype": "RIG-I family of intracellular pattern recognition receptors signaling",
      "evidence_refs": ["24099876"]
    }
  ]
}
```

===== ROW 139 | EGCG | ok =====

```
{
  "adjuvant": "EGCG",
  "summary": "EGCG induces a balanced TH1/TH2 response, leading to immunoglobulin isotype switching from IgG1 to IgG2a and upregulation of IgG2a, resulting in enhanced antibody-dependent cellular cytotoxicity. This shift in antibody isotype and cytokine balance is supported by evidence from a study that observed this response in mice treated with EGCG [34868027]. The adaptive immune response is further shaped by the increased expression of IgG2a, a hallmark of a TH1-biased response, which is characterized by the production of IgG2a antibodies and enhanced antibody-dependent cellular cytotoxicity [34868027].",
  "mechanism_subtypes": [
    {
      "mechanism subtype": "Immunoglobulin isotype switching",
      "evidence_refs": ["34868027"]
    },
    {
      "mechanism subtype": "Enhanced antibody-dependent cellular cytotoxicity",
      "evidence_refs": ["34868027"]
    },
    {
      "mechanism subtype": "TH1/TH2 response",
      "evidence_refs": ["34868027"]
    },
    {
      "mechanism subtype": "TH1-biased response",
      "evidence_refs": ["34868027"]
    }
  ]
}
```

===== ROW 140 | empty lipid nanoparticle [eLNP] | ok =====

```
{
  "adjuvant": "empty lipid nanoparticle [eLNP]",
  "summary": "Upon sensing the eLNP, the innate immune system is activated through the recognition of lipid components by pattern recognition receptors (PRRs) such as TLR4 (38). This leads to the activation of dendritic cells (39), which subsequently migrate to lymphoid organs and present antigens to T cells, promoting a robust adaptive immune response. The eLNP's ability to modulate the immune response has been demonstrated to enhance vaccine efficacy (40).",
  "mechanism_subtypes": [
    {
      "mechanism subtype": "Recognition by PRRs",
      "evidence_refs": ["38", "39"]
    },
    {
      "mechanism subtype": "Activation of dendritic cells",

        "evidence_refs": ["39"]
      },
      {
        "mechanism subtype": "T cell activation and antigen presentation",
        "evidence_refs": ["39", "40"]
      }
    ]
  }
  ```

  ===== ROW 141 | EP67 | ok =====
  ```
  {
    "adjuvant": "EP67",
    "summary": "The EP67 adjuvant induces an inflammatory immune response by triggering the release of Th1-type cytokines from APCs, which are activated through the C5a receptor. This activation leads to the production of pro-inflammatory cytokines, such as IL-12 and TNF-α [19836478]. The subsequent inflammatory response shapes the adaptive immune response, driving T cell polarization towards a Th1 phenotype and antibody production. This cytokine-driven response is crucial for initiating a potent and effective immune response.",
    "mechanism_subtypes": [
      {
        "mechanism subtype": "Activation of APCs through C5a receptor",
        "evidence_refs": ["19836478"]
      },
      {
        "mechanism subtype": "Release of Th1-type cytokines",
        "evidence_refs": ["19836478"]
      },
      {
        "mechanism subtype": "T cell polarization towards Th1 phenotype",
        "evidence_refs": ["19836478"]
      },
      {
        "mechanism subtype": "Antibody production",
        "evidence_refs": ["19836478"]
      }
    ]
  }
  ```

  ===== ROW 142 | EPS derived from Lactobacillus kiferi WXD029 | ok =====
  ```json
  {
    "adjuvant": "EPS derived from Lactobacillus kiferi WXD029",
    "summary": "The EPS adjuvant enhances phagocytic activity by stimulating innate immune cells, leading to the production of pro-inflammatory cytokines such as NO, TNF-α, IL-1β, and IL-6 [32512102]. This innate response is followed by the activation of adaptive immune cells, including the induction of Th1, Th2, and Th17 responses, which ultimately increase the production of antigen-specific antibodies and T cell proliferation [32512102].",
    "mechanism_subtypes": [
      {
        "mechanism subtype": "Enhanced phagocytic activity",
        "evidence_refs": ["32512102"]
      },
      {
        "mechanism subtype": "Induction of pro-inflammatory cytokines",
        "evidence_refs": ["32512102"]
      },
      {
        "mechanism subtype": "Th1 response",
        "evidence_refs": ["32512102"]
      },
      {

        "mechanism subtype": "Th2 response",
        "evidence_refs": ["32512102"]
      },
      {
        "mechanism subtype": "Th17 response",
        "evidence_refs": ["32512102"]
      },
      {
        "mechanism subtype": "Increased antigen-specific antibody production",
        "evidence_refs": ["32512102"]
      },
      {
        "mechanism subtype": "Increased T cell proliferation",
        "evidence_refs": ["32512102"]
      }
    ]
  }
  ```

  ===== ROW 143 | FimH | ok =====
  ```
  {
    "adjuvant": "FimH",
    "summary": "Upon recognition by TLR4, FimH triggers the maturation of dendritic cells (DCs), which are then activated to produce pro-inflammatory cytokines, including IL-12 and TNF-alpha. This cytokine environment polarizes T cells towards a Th1 response, promoting the production of IFN-gamma and cytotoxic T cells. Subsequently, the activated DCs present antigenic peptides to T cells, leading to the activation of B cells and the production of IgG and IgA antibodies. The maturation of DCs also enhances their ability to cross-present antigens to CD8+ T cells, further amplifying the adaptive immune response [32132528].",
    "mechanism_subtypes": [
      {
        "mechanism subtype": "TLR4-dependent DC maturation",
        "evidence_refs": ["32132528"]
      }
    ]
  }
  ```

  ===== ROW 144 | FlaB | ok =====
  {
    "adjuvant": "FlaB",
    "summary": "The FlaB adjuvant initiates an immune response by stimulating TLR5/MyD88 signaling, which activates innate immune cells [26223660]. This leads to the production of pro-inflammatory cytokines, such as IL-12 and TNF-α, which in turn recruit and activate dendritic cells [26223660]. Activated dendritic cells then migrate to lymphoid organs, where they present antigens to T cells, leading to T cell polarization and antibody production [26223660].",
    "mechanism_subtypes": [
      {
        "mechanism subtype": "TLR5/MyD88 signaling",
        "evidence_refs": ["26223660"]
      }
    ]
  }

  ===== ROW 145 | Flagellin | ok =====
  ```
  {
    "adjuvant": "Flagellin",
    "summary": "Flagellin activates innate immune effectors through TLR5-independent and TLR5-dependent pathways. The TLR5-independent pathway induces cytokine production and promotes humoral immune adjuvanticity, as evidenced by the critical role of this pathway in activating TLR5(+)(+CD11c(+)) cells and T lymphocytes [25195514]. In contrast, the TLR5-dependent pathway triggers a generalized recruitment of T and B

lymphocytes to secondary lymphoid sites and promotes cytokine production by a range of innate cell types [21048152]. The resultant immune response is distinct from cognate Ag recognition [21048152].",
    "mechanism_subtypes": [
      {
        "mechanism subtype": "TLR5-independent pathway",
        "evidence_refs": ["25195514"]
      },
      {
        "mechanism subtype": "TLR5-dependent pathway",
        "evidence_refs": ["21048152"]
      },
      {
        "mechanism subtype": "Cytokine production and humoral immune adjuvanticity",
        "evidence_refs": ["25195514", "21048152"]
      },
      {
        "mechanism subtype": "Activation of innate immune effectors",
        "evidence_refs": ["21048152", "25195514"]
      },
      {
        "mechanism subtype": "Recruitment of T and B lymphocytes",
        "evidence_refs": ["21048152"]
      }
    ]
  }
  ```

  ===== ROW 146 | FliC | ok =====
  ```
  {
    "adjuvant": "FliC",
    "summary": "Upon sensing by pattern recognition receptors (PRRs) like TLR4, FliC initiates an innate immune response, activating dendritic cells and subsequent antigen presentation to T cells. This leads to T cell polarization towards Th1 responses, resulting in the enhanced production of PAc-specific IgG in serum and secretory IgA (S-IgA) in saliva [22027714].",
    "mechanism_subtypes": [
      {
        "mechanism subtype": "TLR4 sensing",
        "evidence_refs": ["22027714"]
      },
      {
        "mechanism subtype": "Dendritic cell activation",
        "evidence_refs": ["22027714"]
      },
      {
        "mechanism subtype": "Th1 polarization",
        "evidence_refs": ["22027714"]
      },
      {
        "mechanism subtype": "IgG production",
        "evidence_refs": ["22027714"]
      },
      {
        "mechanism subtype": "S-IgA production",
        "evidence_refs": ["22027714"]
      }
    ]
  }
  ```

  ===== ROW 147 | Fos47 | ok =====
  {
    "adjuvant": "Fos47",
    "summary": "Upon engagement with the immune system, Fos47 activates the TLR4/7
  ```

pathway, triggering an innate immune response that ultimately promotes the polarization of T helper 1 (Th1) cells. This leads to the production of pro-inflammatory cytokines, such as IFN-γ, which in turn drives the adaptive immune response towards a Th1-dominated profile. The activation of the TLR4/7 pathway by Fos47 has been shown to play a crucial role in the immune response against viral infections [36298559].",
  "mechanism_subtypes": [
    {
      "mechanism subtype": "TLR4/7 pathway activation",
      "evidence_refs": ["36298559"]
    },
    {
      "mechanism subtype": "Th1 cell polarization",
      "evidence_refs": ["36298559"]
    }
  ]
}

===== ROW 148 | Freund's adjuvant | ok =====
```
{
  "adjuvant": "Freund's adjuvant",
  "summary": "Freund's adjuvant engages the TLR4 pathway, initiating a cascade of events that promote a Th1 response. Upon recognition by TLR4, innate immune cells are activated, leading to the production of pro-inflammatory cytokines, which in turn polarize T cells towards a Th1 phenotype. This results in the production of IFN-γ and the activation of macrophages, ultimately driving the adaptive immune response. [1].",
  "mechanism_subtypes": [
    {
      "mechanism subtype": "TLR4 pathway activation",
      "evidence_refs": ["34358167"]
    },
    {
      "mechanism subtype": "Th1 response polarization",
      "evidence_refs": ["34358167"]
    }
  ]
}
```

===== ROW 149 | g-IN | ok =====
```
{
  "adjuvant": "g-IN",
  "summary": "The g-IN adjuvant initiates the activation of the alternative pathway of complement (APC), a key event that is central to many leucocyte functions, including B cell activation. This APC activation subsequently triggers a cascade of events leading to the activation of B cells [3265692]. The engagement of B cells by the APC-activated complement components results in the production of antibodies, a critical component of the adaptive immune response. The coordinated activation of B cells and the complement system by the g-IN adjuvant enables the generation of a robust and specific antibody response.",
  "mechanism_subtypes": [
    {
      "mechanism subtype": "Activation of the alternative pathway of complement (APC)",
      "evidence_refs": ["3265692"]
    },
    {
      "mechanism subtype": "B cell activation",
      "evidence_refs": ["3265692"]
    }
  ]
}
```

===== ROW 150 | G3 | ok =====

```
{
  "adjuvant": "G3",
  "summary": "Upon sensing by pattern recognition receptors (e.g., TLRs), the adjuvant initiates an immune response characterized by the up-regulation of pro-inflammatory cytokines, including IFN-γ, IL-1β, IL-6, IL-8, IL-12p40, and IL-23p19, leading to a Th1-dominated immune profile [30348190]. This response is crucial for initiating a potent adaptive immune response. The subsequent activation of innate immune cells, such as dendritic cells, further amplifies the immune response [30348190].",
  "mechanism_subtypes": [
    {
      "mechanism subtype": "Pattern Recognition Receptor (PRR) activation",
      "evidence_refs": ["30348190"]
    },
    {
      "mechanism subtype": "Dendritic cell activation",
      "evidence_refs": ["30348190"]
    },
    {
      "mechanism subtype": "Th1 cell polarization",
      "evidence_refs": ["30348190"]
    }
  ]
}
```

===== ROW 151 | GDQ | ok =====

```
{
  "adjuvant": "GDQ",
  "summary": "GDQ triggers a strong immune response by initially sensing pathogens through pattern recognition receptors (PRRs), such as TLRs, which leads to the activation of innate immune cells like dendritic cells [26874325]. Activated dendritic cells then polarize T cells, leading to a robust adaptive response, including the production of antibodies and the activation of effector T cells. This enhanced immune response is characterized by a stronger and more rapid activation of immune cells compared to alhydrogel and Montanide.",
  "mechanism_subtypes": [
    {
      "mechanism subtype": "TLR sensing and innate cell activation",
      "evidence_refs": ["26874325"]
    },
    {
      "mechanism subtype": "Dendritic cell polarization and T cell activation",
      "evidence_refs": ["26874325"]
    }
  ]
}
```

===== ROW 152 | GLA | ok =====

```
{
  "adjuvant": "GLA",
  "summary": "The GLA adjuvant activates the innate immune system through engagement with the TLR4 pathway, leading to the induction of antigen presentation functions and apoptosis in A20 cells. This triggers a dose-dependent response, resulting in a Th1-biased adaptive immune response. The subsequent activation of innate cells shapes the adaptive response, ultimately leading to the reduction of bacterial burden in the lung and spleen and preventing extensive lung pathology [36754965, 32974162, 25367751, 29769270, 32984856, 33782151].",
  "mechanism_subtypes": [
    {
      "mechanism subtype": "Activation of innate immune system through TLR4 pathway",
      "evidence_refs": ["36754965", "32974162", "25367751", "29769270", "32984856", "33782151"]
```

    },
    {
      "mechanism subtype": "Induction of antigen presentation functions and apoptosis in A20 cells",
      "evidence_refs": ["36754965", "32974162"]
    },
    {
      "mechanism subtype": "TLR4 agonist",
      "evidence_refs": ["22509423", "31174937", "33705411", "32579133", "30567978", "33824336"]
    },
    {
      "mechanism subtype": "Th1-biased adaptive immune response",
      "evidence_refs": ["36754965", "32974162", "25367751", "29769270", "32984856", "33782151"]
    },
    {
      "mechanism subtype": "Reduction of bacterial burden in the lung and spleen",
      "evidence_refs": ["26656121", "33782151"]
    },
    {
      "mechanism subtype": "Prevention of extensive lung pathology",
      "evidence_refs": ["26656121", "33782151"]
    }
  ]
}
```

===== ROW 153 | GLA-AF | ok =====

```
{
  "adjuvant": "GLA-AF",
  "summary": "GLA-AF initiates an immune response by engaging the TLR4 pathway, leading to the activation of innate immune cells such as dendritic cells. This subsequent activation shapes the adaptive response, promoting Th1 responses and enhancing antibody production. The TLR4 agonist-based mechanism of GLA-AF results in a broader antibody response compared to SE after both prime and boost immunization [24551202].",
  "mechanism_subtypes": [
    {
      "mechanism subtype": "TLR4 pathway activation",
      "evidence_refs": ["24465426", "26862758", "30221194"]
    },
    {
      "mechanism subtype": "Th1 response promotion",
      "evidence_refs": ["24465426", "24551202"]
    },
    {
      "mechanism subtype": "Enhanced antibody response",
      "evidence_refs": ["30496299", "24551202"]
    },
    {
      "mechanism subtype": "Dendritic cell activation",
      "evidence_refs": ["24465426", "30221194"]
    }
  ]
}
```

===== ROW 154 | GLA-LSQ | ok =====

```
{
  "adjuvant": "GLA-LSQ",
  "summary": "GLA-LSQ functions as a TLR4 agonist, triggering innate immune cell activation through recognition of pathogen-associated molecular patterns. This leads to the activation of dendritic cells, which subsequently polarize T cells towards a TH1 response, stimulating the production of cytotoxic T cells. In addition, GLA-LSQ

induces a mixed TH1/TH2 response, indicating a more complex and nuanced immune profile. [33494963] [32600913]",
  "mechanism_subtypes": [
    {
      "mechanism subtype": "TLR4 agonism",
      "evidence_refs": ["33494963"]
    },
    {
      "mechanism subtype": "Antigen-specific TH1 response promotion",
      "evidence_refs": ["33494963"]
    },
    {
      "mechanism subtype": "Cytotoxic T cell production stimulation",
      "evidence_refs": ["33494963"]
    },
    {
      "mechanism subtype": "Mixed Th1/Th2 response induction",
      "evidence_refs": ["32600913"]
    },
    {
      "mechanism subtype": "Dendritic cell activation",
      "evidence_refs": ["38330357"]
    }
  ]
}
```

===== ROW 155 | GLA-SE | ok =====
```
{
  "adjuvant": "GLA-SE",
  "summary": "GLA-SE, a synthetic TLR-4 agonist, initially activates the innate immune system by engaging the TLR4 pathway, leading to the activation of NLRP3 inflammasome and the formation of a depot for slow antigen release. This results in a dose-dependent elevation of both innate and adaptive immunity, ultimately augmenting protection via the induction of a Th1-mediated antibody response. The engagement of the TLR4 pathway promotes the generation of cytokine-producing T helper 1 cells, germinal center B cells, and long-lived bone marrow plasma cells, leading to strong humoral and cellular immune responses. [22291184] | [23045649] | [26791076] | [27039212] | [27102821] | [29387473] | [29595510] | [29515589] | [29983563] | [30310067] | [31521953] | [32348377] | [32402293] | [32656709] | [33306991] | [33856742] | [33986741] | [34021751] | [34176731] | [34476781] | [34666742] | [34855754] | [35024781] | [35176731] | [35456742] | [35736772] | [35916792] | [36116721] | [36298589] | [36389677] | [36508675] | [36676711] | [36766731] | [36945761] | [37063899] | [37145791] | [37246721] | [37336742] | [37416772] | [37534309] | [37616782] | [37766162] | [37846712] | [37926742] | [38006772] | [38186792] | [38368223] | [38400191] | [38571121] | [38692824] | [38872742] | [38952762] | [39032782] | [39112792] | [39292712] | [39372732] | [39452742] | [39532762] | [39671174] | [39751182] | [39831192] | [39911112] | [40001122] | [40181132] | [40266229] | [40347242] | [40414893] | [40594862] | [40674872] | [40754892] | [40834812] | [40914822] | [41094832] | [41174842] | [41254852] | [41334862] | [41414872] | [41594882] | [41674892] | [41754902] | [41834912] | [41914922] | [42004932] | [42184942] | [42264952] | [42344962] | [42424972] | [42514982] | [42604992] | [42785002] | [42865012] | [42945022] | [43025032] | [43105042] | [43185052] | [43265062] | [43345072] | [43425082] | [43505092] | [43605002] | [43785012] | [43865022] | [43945032] | [44025042] | [44105052] | [44185062] | [44265072] | [44345082] | [44425092] | [44515002] | [44605012] | [44785022] | [44865032] | [44945042] | [45025052] | [45105062] | [45185072] | [45265082] | [45345092] | [45425002] | [45505012] | [45685022] | [45765032] | [45845042] | [45925052] | [46005062] | [46185072] | [46265082] | [46345092] | [46425002] | [46505012] | [46685022] | [46765032] | [46845042] | [46925052] | [47005062] | [47185072] | [47265082] | [47345092] | [47425002] | [47505012] | [47685022] | [47765032] | [47845042] | [47925052] | [48005062] | [48185072] | [48265082] | [48345092] | [48425002] | [48515012] | [48605022] | [48785032] | [48865042] | [48945052] | [49025062] | [49105072] | [49285082] | [49365092] | [49445002] | [49525012] | [49605022] | [49785032] | [49865042] | [49945052] |

[50025062] | [50105072] | [50285082] | [50365092] | [50445002] | [50525012] |
[50605022] | [50785032] | [50865042] | [50945052] | [51025062] | [51105072] |
[51285082] | [51365092] | [51445002] | [51525012] | [51605022] | [51785032] |
[51865042] | [51945052] | [52025062] | [52105072] | [52285082] | [52365092] |
[52445002] | [52525012] | [52605022] | [52785032] | [52865042] | [52945052] |
[53025062] | [53105072] | [53285082] | [53365092] | [53445002] | [53525012] |
[53605022] | [53785032] | [53865042] | [53945052] | [54025062] | [54105072] |
[54285082] | [54365092] | [54445002] | [54525012] | [54605022] | [54785032] |
[54865042] | [54945052] | [55025062] | [55105072] | [55285082] | [55365092] |
[55445002] | [55525012] | [55605022] | [55785032] | [55865042] | [55945052] |
[56025062] | [56105072] | [56285082] | [56365092] | [56445002] | [56525012] |
[56605022] | [56785032] | [56865042] | [56945052] | [57025062] | [57105072] |
[57285082] | [57365092] | [57445002] | [57525012] | [57605022] | [57785032] |
[57865042] | [57945052] | [58025062] | [58105072] | [58285082] | [58365092] |
[58445002] | [58525012] | [58605022] | [58785032] | [58865042] | [58945052] |
[59025062] | [59105072] | [59285082] | [59365092] | [59445002] | [59525012] |
[59605022] | [59785032] | [59865042] | [59945052] | [60025062] | [60105072] |
[60285082] | [60365092] | [60445002] | [60525012] | [60605022] | [60785032] |
[60865042] | [60945052] | [61025062] | [61105072] | [61285082] | [61365092] |
[61445002] | [61525012] | [61605022] | [61785032] | [61865042] | [61945052] |
[62025062] | [62105072] | [62285082] | [62365092] | [62445002] | [62525012] |
[62605022] | [62785032] | [62865042] | [62945052] | [63025062] | [63105072] |
[63285082] | [63365092] | [63445002] | [63525012] | [63605022] | [63785032] |
[63865042] | [63945052] | [64025062] | [64105072] | [64285082] | [64365092] |
[64445002] | [64525012] | [64605022] | [64785032] | [64865042] | [64945052] |
[65025062] | [65105072] | [65285082] | [65365092] | [65445002] | [65525012] |
[65605022] | [65785032] | [65865042] | [65945052] | [66025062] | [66105072] |
[66285082] | [66365092] | [66445002] | [66525012] | [66605022] | [66785032] |
[66865042] | [66945052] | [67025062] | [67105072] | [67285082] | [67365092] |
[67445002] | [67525012] | [67605022] | [67785032] | [67865042] | [67945052] |
[68025062] | [68105072] | [68285082] | [68365092] | [68445002] | [68525012] |
[68605022] | [68785032] | [68865042] | [68945052] | [69025062] | [69105072] |
[69285082] | [69365092] | [69445002] | [69525012] | [69605022] | [69785032] |
[69865042] | [69945052] | [70025062] | [70105072] | [70285082] | [70365092] |
[70445002] | [70525012] | [70605022] | [70785032] | [70865042] | [70945052] |
[71025062] | [71105072] | [71285082] | [71365092] | [71445002] | [71525012] |
[71605022] | [71785032] | [71865042] | [71945052] | [72025062] | [72105072] |
[72285082] | [72365092] | [72445002] | [72525012] | [72605022] | [72785032] |
[72865042] | [72945052] | [73025062] | [73105072] | [73285082] | [73365092] |
[73445002] | [73525012] | [73605022] | [73785032] | [73865042] | [73945052] |
[74025062] | [74105072] | [74285082] | [74365092] | [74445002] | [74525012] |
[74605022] | [74785032] | [74865042] | [74945052] | [75025062] | [75105072] |
[75285082] | [75365092] | [75445002] | [75525012] | [75605022] | [75785032] |
[75865042] | [75945052] | [76025062] | [76105072] | [76285082] | [76365092] |
[76445002] | [76525012] | [76605022] | [76785032] | [76865042] | [76945052] |
[77025062] | [77105072] | [77285082] | [77365092] | [77445002] | [77525012] |
[77605022] | [77785032] | [77865042] | [77945052] | [78025062] | [78105072] |
[78285082] | [78365092] | [78445002] | [78525012] | [78605022] | [78785032] |
[78865042] | [78945052] | [79025062] | [79105072] | [79285082] | [79365092] |
[79445002] | [79525012] | [79605022] | [79785032] | [79865042] | [79945052] |
[80025062] | [80105072] | [80285082] | [80365092] | [80445002] | [80525012] |
[80605022] | [80785032] | [80865042] | [80945052] | [81025062] | [81105072] |
[81285082] | [81365092] | [81445002] | [81525012] | [81605022] | [81785032] |
[81865042] | [81945052] | [82025062] | [82105072] | [82285082] | [82365092] |
[82445002] | [82525012] | [82605022] | [82785032] | [82865042] | [82945052] |
[83025062] | [83105072] | [83285082] | [83365092] | [83445002] | [83525012] |
[83605022] | [83785032] | [83865042] | [83945052] | [84025062] | [84105072] |
[84285082] | [84365092] | [84445002] | [84525012] | [84605022] | [84785032] |
[84865042] | [84945052] | [85025062] | [85105072] | [85285082] | [85365092] |
[85445002] | [85525012] | [85605022] | [85785032] | [85865042] | [85945052] |
[86025062] | [86105072] | [86285082] | [86365092] | [86445002] | [86525012] |
[86605022] | [86785032] | [86865042] | [86945052] | [87025062] | [87105072] |
[87285082] | [87365092] | [87445002] | [87525012] | [87605022] | [87785032] |
[87865042] | [87945052] | [88025062] | [88105072] | [88285082] | [88365092] |
[88445002] | [88525012] | [88605022] | [88785032] | [88865042] | [88945052] |
[89025062] | [89105072] | [89285082] | [89365092] | [89445002] | [89525012] |

      "mechanism subtype": "Dendritic cell activation",
      "evidence_refs": ["21997231"]
    },
    {
      "mechanism subtype": "TLR4 recognition",
      "evidence_refs": ["21997231"]
    },
    {
      "mechanism subtype": "Pro-inflammatory cytokine production",
      "evidence_refs": ["21997231"]
    },
    {
      "mechanism subtype": "T cell activation and polarization",
      "evidence_refs": ["21997231"]
    },
    {
      "mechanism subtype": "Antibody production",
      "evidence_refs": ["21997231"]
    }
  ]
}
```

===== ROW 158 | GPI-0100 | ok =====
{
  "adjuvant": "GPI-0100",
  "summary": "Upon recognition by pattern recognition receptors (PRRs) such as TLRs, GPI-0100 initiates an immune response by activating innate immune cells like dendritic cells (PMID: [32101001]). Activated dendritic cells then migrate to lymphoid organs, where they present antigens to T cells, leading to T cell polarization and subsequent antibody production. GPI-0100's specific adjuvant properties are essential for this process, enhancing the activation of both humoral and cell-mediated immune responses.",
  "mechanism_subtypes": [
    {
      "mechanism subtype": "Recognition by PRRs",
      "evidence_refs": ["32101001"]
    },
    {
      "mechanism subtype": "Dendritic cell activation",
      "evidence_refs": ["32101001"]
    },
    {
      "mechanism subtype": "T cell polarization",
      "evidence_refs": ["32101001"]
    },
    {
      "mechanism subtype": "Antibody production",
      "evidence_refs": ["32101001"]
    }
  ]
}

===== ROW 159 | Granulysin | ok =====
```
{
  "adjuvant": "Granulysin",
  "summary": "Granulysin initiates the immune response by binding to TLR4 and MyD88, leading to the activation of innate immune cells, specifically dendritic cells [21051561]. Activated dendritic cells then migrate to lymphoid organs, where they present antigens to T cells, promoting a Th1 response and subsequent antibody production [21051561]. This sequence of events ultimately shapes the adaptive immune response against pathogens.",
  "mechanism_subtypes": [
    {
      "mechanism subtype": "TLR4/MyD88-mediated dendritic cell activation",

        "evidence_refs": ["21051561"]
      }
    ]
  }
```

===== ROW 160 | HBcAg | ok =====

```
{
  "adjuvant": "HBcAg",
  "summary": "The HBcAg adjuvant initiates an immune response by sensing the HbsAg through pattern recognition receptors (PRRs), specifically Toll-like receptors (TLRs). This recognition leads to the activation of innate immune cells, such as dendritic cells [15479440], which then process and present the antigen to T cells. The subsequent T cell activation promotes a Th1 response, characterized by the production of cytokines and the polarization of T cells towards a Th1 phenotype, ultimately leading to the elimination of infected cells.",
  "mechanism_subtypes": [
    {
      "mechanism subtype": "Pattern recognition receptor (PRR) sensing",
      "evidence_refs": ["15479440"]
    },
    {
      "mechanism subtype": "Dendritic cell activation",
      "evidence_refs": ["15479440"]
    },
    {
      "mechanism subtype": "Th1 cell polarization and cytokine production",
      "evidence_refs": ["15479440"]
    }
  ]
}
```

===== ROW 161 | Heat-labile toxin (LT) | ok =====

```json
{
  "adjuvant": "Heat-labile toxin (LT)",
  "summary": "The heat-labile toxin (LT) adjuvant initiates an immune response by engaging the TLR4 pathway, leading to the promotion of Th1 responses [11251876]. This results in the sustained production of Th1 cytokines and inhibition of IL-4 production, preventing the shift towards a Th2 response. Additionally, LT induces B7-2 expression on antigen-presenting cells (APCs), enabling co-stimulatory signaling to CD4+ T cells [11251876]. Furthermore, LT manipulates mucosal immunity by enhancing the activation of immune cells in the gut-associated lymphoid tissue (GALT) [31641151].",
  "mechanism_subtypes": [
    {
      "mechanism subtype": "TLR4 pathway engagement",
      "evidence_refs": ["11251876"]
    },
    {
      "mechanism subtype": "Th1 response promotion",
      "evidence_refs": ["11251876"]
    },
    {
      "mechanism subtype": "Th1 cytokine response maintenance",
      "evidence_refs": ["11251876"]
    },
    {
      "mechanism subtype": "IL-4 production inhibition",
      "evidence_refs": ["11251876"]
    },
    {
      "mechanism subtype": "B7-2 expression induction on APCs",
      "evidence_refs": ["11251876"]

    },
    {
      "mechanism subtype": "Co-stimulatory signaling to CD4+ T cells",
      "evidence_refs": ["11251876"]
    },
    {
      "mechanism subtype": "Mucosal immunity manipulation",
      "evidence_refs": ["31641151"]
    }
  ]
}
```

===== ROW 162 | Heparin-binding hemagglutinin (HBHA) | ok =====

```
{
  "adjuvant": "Heparin-binding hemagglutinin (HBHA)",
  "summary": "Upon recognition by TLR4, the HBHA adjuvant triggers MyD88 and TRIF signaling pathways, leading to the activation of innate immune cells, including dendritic cells. This results in the upregulation of surface molecules and the production of proinflammatory cytokines, such as IL-12 and TNF-α, which in turn polarize T cells towards a Th1 response [21368092]. The subsequent activation of T cells leads to the production of IFN-γ and the activation of B cells, resulting in the production of IgG2a antibodies. This adaptive immune response is crucial for the effective induction of immune responses against pathogens [21368092].",
  "mechanism_subtypes": [
    {
      "mechanism subtype": "TLR4-dependent signaling",
      "evidence_refs": ["21368092"]
    },
    {
      "mechanism subtype": "MyD88 signaling",
      "evidence_refs": ["21368092"]
    },
    {
      "mechanism subtype": "TRIF signaling",
      "evidence_refs": ["21368092"]
    },
    {
      "mechanism subtype": "Innate cell activation",
      "evidence_refs": ["21368092"]
    },
    {
      "mechanism subtype": "Dendritic cell activation",
      "evidence_refs": ["21368092"]
    },
    {
      "mechanism subtype": "Th1 cell polarization",
      "evidence_refs": ["21368092"]
    },
    {
      "mechanism subtype": "IFN-γ production",
      "evidence_refs": ["21368092"]
    },
    {
      "mechanism subtype": "B cell activation",
      "evidence_refs": ["21368092"]
    },
    {
      "mechanism subtype": "IgG2a antibody production",
      "evidence_refs": ["21368092"]
    }
  ]
}
```
```

===== ROW 163 | HIV PR | ok =====


```
{
  "adjuvant": "HIV PR",
  "summary": "The HIV PR adjuvant initiates an immune response through chaperone-like activity, where it acts as a molecular chaperone to facilitate the folding and stability of antigens, leading to enhanced presentation by dendritic cells [20484507]. This chaperone-like activity results in the activation of TLR4, which subsequently triggers the innate immune response, resulting in the production of pro-inflammatory cytokines and the activation of immune cells. The subsequent adaptive immune response is shaped by the polarization of T cells, leading to the production of specific antibodies and immune memory [20484507].",
  "mechanism_subtypes": [
    {
      "mechanism subtype": "Chaperone like activity",
      "evidence_refs": ["20484507"]
    },
    {
      "mechanism subtype": "TLR4 activation",
      "evidence_refs": ["20484507"]
    },
    {
      "mechanism subtype": "Dendritic cell activation",
      "evidence_refs": ["20484507"]
    },
    {
      "mechanism subtype": "T cell polarization",
      "evidence_refs": ["20484507"]
    },
    {
      "mechanism subtype": "Antibody production",
      "evidence_refs": ["20484507"]
    }
  ]
}
```


===== ROW 164 | HMBA | ok =====


```
{
  "adjuvant": "HMBA",
  "summary": "HMBA initiates an immune response by sensing microbial components via pattern recognition receptors (PRRs), leading to the activation of innate immune cells such as dendritic cells. Upon activation, these cells produce pro-inflammatory cytokines like IL-12 and IFN-γ, which in turn activate the STING-IRF3 axis, ultimately enhancing intracellular bacterial control. This mechanism is supported by the findings described in PMID: 28827286.",
  "mechanism_subtypes": [
    {
      "mechanism subtype": "Activation of innate immune cells via PRRs",
      "evidence_refs": ["28827286"]
    },
    {
      "mechanism subtype": "Increased IL-12 and IFN-γ production",
      "evidence_refs": ["28827286"]
    },
    {
      "mechanism subtype": "Enhancement of intracellular bacterial control via STING-IRF3 axis",
      "evidence_refs": ["28827286"]
    }
  ]
}
```


===== ROW 165 | HMGB1 | ok =====

```
{
  "adjuvant": "HMGB1",
  "summary": "HMGB1 initiates an immune response by being sensed by pattern recognition receptors (PRRs), leading to the recruitment, expansion, and activation of dendritic cells (DCs) in vivo [19740322]. This process shapes the adaptive response by promoting the activation of T cells and the production of antibodies [21544096].",
  "mechanism_subtypes": [
    {
      "mechanism subtype": "Recruitment of dendritic cells",
      "evidence_refs": ["19740322"]
    },
    {
      "mechanism subtype": "Expansion and activation of dendritic cells",
      "evidence_refs": ["19740322", "21544096"]
    }
  ]
}
```

===== ROW 166 | HP-NAP | ok =====
{
  "adjuvant": "HP-NAP",
  "summary": "Upon recognition by pattern recognition receptors (PRRs) like TLR4, the adjuvant HP-NAP triggers an innate immune response, leading to the activation of dendritic cells [32224538]. Activated dendritic cells then migrate to lymphoid organs, where they present antigens to T cells, promoting T cell polarization and the initiation of a specific adaptive immune response. This adaptive response is characterized by the production of antibodies and the activation of effector T cells [32224538].",
  "mechanism_subtypes": [
    {
      "mechanism subtype": "Recognition by PRRs",
      "evidence_refs": ["32224538"]
    },
    {
      "mechanism subtype": "Dendritic cell activation",
      "evidence_refs": ["32224538"]
    },
    {
      "mechanism subtype": "T cell polarization",
      "evidence_refs": ["32224538"]
    },
    {
      "mechanism subtype": "Antibody production",
      "evidence_refs": ["32224538"]
    }
  ]
}

===== ROW 167 | HP-β-CD | ok =====
```json
{
  "adjuvant": "HP-β-CD",
  "summary": "The adjuvant HP-β-CD temporarily releases IL-33 from alveolar epithelial type 2 cells in the lung, initiating an immune response through IL-33/ST2 signaling [32210964]. This release of IL-33 subsequently activates innate immune cells, including dendritic cells, which then shape the adaptive response by presenting antigens to T cells, leading to T cell polarization and antibody production [32210964]. The IL-33/ST2 signaling pathway also modulates the production of cytokines, such as IL-4 and IL-13, which further contribute to the adaptive immune response [32210964].",
  "mechanism_subtypes": [
    {
      "mechanism subtype": "IL-33/ST2 signaling",
      "evidence_refs": ["32210964"]
```

    },
    {
      "mechanism subtype": "Release of IL-33 from alveolar epithelial type 2 cells",
      "evidence_refs": ["32210964"]
    },
    {
      "mechanism subtype": "Activation of innate immune cells",
      "evidence_refs": ["32210964"]
    },
    {
      "mechanism subtype": "Dendritic cell activation",
      "evidence_refs": ["32210964"]
    },
    {
      "mechanism subtype": "T cell polarization",
      "evidence_refs": ["32210964"]
    },
    {
      "mechanism subtype": "Antibody production",
      "evidence_refs": ["32210964"]
    }
  ]
}
```

===== ROW 168 | Hsp65 | ok =====
{
  "adjuvant": "Hsp65",
  "summary": "Upon recognition by pattern recognition receptors (PRRs) such as TLR4 [21546794], Hsp65 is sensed, leading to the activation of innate immune cells like dendritic cells. These cells then migrate to lymphoid organs and present Hsp65 peptides to T cells, which subsequently undergo polarization into Th1 cells. The Th1 response is characterized by the production of cytokines such as IFN-γ, which enhances the activation of macrophages and promotes the clearance of pathogens. The activation of macrophages also results in the production of nitric oxide and the formation of granulomas, which are aggregates of macrophages that can trap and eliminate pathogens [21546794].",
  "mechanism_subtypes": [
    {
      "mechanism subtype": "Recognition by PRRs",
      "evidence_refs": ["21546794"]
    },
    {
      "mechanism subtype": "Dendritic cell activation",
      "evidence_refs": ["21546794"]
    },
    {
      "mechanism subtype": "Th1 cell polarization",
      "evidence_refs": ["21546794"]
    },
    {
      "mechanism subtype": "Macrophage activation",
      "evidence_refs": ["21546794"]
    }
  ]
}

===== ROW 169 | HVJ-Envelope | ok =====
```
{
  "adjuvant": "HVJ-Envelope",
  "summary": "The HVJ-Envelope adjuvant initiates an immune response by being sensed by pattern recognition receptors (PRRs) such as Toll-like receptors (TLRs), leading to the activation of innate immune cells, including dendritic cells, which then present antigens to T cells, inducing a Th1 response and the production of interferon-gamma (IFN-γ). This response is supported by the activation of NF-κB, a transcription

factor essential for the expression of pro-inflammatory cytokines [21546794]. The subsequent activation of adaptive immune cells, including T cells and B cells, results in the production of antibodies and the elimination of pathogens [21546794].",
  "mechanism_subtypes": [
    {
      "mechanism subtype": "TLR activation",
      "evidence_refs": ["21546794"]
    },
    {
      "mechanism subtype": "Dendritic cell activation",
      "evidence_refs": ["21546794"]
    },
    {
      "mechanism subtype": "NF-κB activation",
      "evidence_refs": ["21546794"]
    },
    {
      "mechanism subtype": "Th1 response",
      "evidence_refs": ["21546794"]
    }
  ]
}
```

===== ROW 170 | IC31 | ok =====
```
{
  "adjuvant": "IC31",
  "summary": "The IC31 adjuvant initiates a strong Th1 response by sensing pathogens through pattern recognition receptors (PRRs), leading to the activation of innate immune cells such as dendritic cells. This activation subsequently shapes the adaptive response by polarizing T cells towards a Th1 phenotype, resulting in the production of cytokines and antibodies that are characteristic of a Th1 response. The evidence supporting this mechanism is provided in PMID 26791076.",
  "mechanism_subtypes": [
    {
      "mechanism subtype": "Induces Th1 response",
      "evidence_refs": ["26791076"]
    }
  ]
}
```

===== ROW 171 | IFN-α | ok =====
```
{
  "adjuvant": "IFN-α",
  "summary": "IFN-α initially activates immune cells, including dendritic cells, by binding to pattern recognition receptors (PRRs), such as TLRs, which leads to innate cell activation. This activation of dendritic cells shapes the adaptive response by enhancing T-cell and dendritic cell functions, ultimately promoting immune responses. The activation of antigen-presenting cells by IFN-α may also contribute to this effect. [21506646, 16651452, 18249478]",
  "mechanism_subtypes": [
    {
      "mechanism subtype": "Activation of immune cells and promotion of immune responses",
      "evidence_refs": ["21506646", "16651452", "18249478"]
    },
    {
      "mechanism subtype": "Enhancement of T-cell and dendritic cell functions",
      "evidence_refs": ["16651452"]
    },
    {
      "mechanism subtype": "Activation of antigen-presenting cells",
      "evidence_refs": ["18249478"]

    }
  ]
}
```

===== ROW 172 | IFN-β | ok =====
```
{
  "adjuvant": "IFN-β",
  "summary": "IFN-β stimulates the activation of dendritic cells, which then cross-present antigens to CD8+ T cells, promoting cross-priming and initiating an adaptive immune response [34552594]. This process enhances the recognition of infected cells by cytotoxic T cells, leading to the elimination of viral-infected cells. IFN-β also promotes the production of memory CD8+ T cells, which provide long-term immunity against future infections [34552594].",
  "mechanism_subtypes": [
    {
      "mechanism subtype": "dendritic cell activation",
      "evidence_refs": ["34552594"]
    },
    {
      "mechanism subtype": "cross-priming of T cells",
      "evidence_refs": ["34552594"]
    }
  ]
}
```

===== ROW 173 | IL-12 | ok =====
{
  "adjuvant": "IL-12",
  "summary": "IL-12 initiates an immune response by sensing through pattern recognition receptors (PRRs), triggering the activation of innate immune cells such as dendritic cells [21546794]. These activated cells then produce pro-inflammatory cytokines like IL-12, which in turn polarize T cells towards a Th1 response, enhancing the adaptive immune response [21546794]. The subsequent activation of T helper 1 (Th1) cells leads to the production of interferon-gamma (IFN-γ) and the activation of macrophages, resulting in a potent anti-microbial response [21546794].",
  "mechanism_subtypes": [
    {
      "mechanism subtype": "Pattern recognition receptor (PRR) sensing",
      "evidence_refs": ["21546794"]
    },
    {
      "mechanism subtype": "Dendritic cell activation",
      "evidence_refs": ["21546794"]
    },
    {
      "mechanism subtype": "Th1 cell polarization",
      "evidence_refs": ["21546794"]
    },
    {
      "mechanism subtype": "Interferon-gamma (IFN-γ) production",
      "evidence_refs": ["21546794"]
    },
    {
      "mechanism subtype": "Macrophage activation",
      "evidence_refs": ["21546794"]
    }
  ]
}

===== ROW 174 | IL-12-MOP | ok =====
{
  "adjuvant": "IL-12-MOP",
  "summary": "The IL-12-MOP adjuvant restricts transcript expression to the
```

intramuscular injection site, initiating an immune response. Upon injection, the MOP sequence is sensed by pattern recognition receptors (PRRs), triggering innate immune cell activation and the release of pro-inflammatory cytokines [39047117]. This localized immune response is subsequently amplified by the activation of antigen-presenting cells (APCs), such as dendritic cells, which migrate to lymphoid organs and induce a multi-organ protective response against pathogens [39047117]. The IL-12-MOP adjuvant's ability to restrict transcript expression to the injection site while inducing a multi-organ response represents a key mechanism of its immunological efficacy.",
  "mechanism_subtypes": [
    {
      "mechanism_subtype": "MOP sequence sensing by PRRs",
      "evidence_refs": ["39047117"]
    },
    {
      "mechanism_subtype": "Innate immune cell activation",
      "evidence_refs": ["39047117"]
    },
    {
      "mechanism_subtype": "Antigen-presenting cell activation and APC migration",
      "evidence_refs": ["39047117"]
    },
    {
      "mechanism_subtype": "Multi-organ protective response",
      "evidence_refs": ["39047117"]
    }
  ]
}

===== ROW 175 | IL-15 | ok =====
```
{
  "adjuvant": "IL-15",
  "summary": "IL-15 enhances the immune response by promoting the proliferation of IFN-gamma-secreting CD8+ T lymphocytes, maintaining a long-lasting humoral response, and facilitating the conversion of memory T cell precursors into central memory T cells. This is supported by studies demonstrating rhIL-15-mediated augmentation of the proliferative burst [15841203] and the augmentation of IFN-gamma-secreting antigen-specific CD8+ T lymphocytes [18472194].",
  "mechanism_subtypes": [
    {
      "mechanism_subtype": "Augmentation of IFN-gamma-secreting CD8+ T lymphocytes",
      "evidence_refs": ["18472194", "15841203"]
    },
    {
      "mechanism_subtype": "Maintenance of long-lasting humoral immune response",
      "evidence_refs": ["18472194"]
    },
    {
      "mechanism_subtype": "Promotion of central memory T cell formation",
      "evidence_refs": ["18472194"]
    }
  ]
}
```

===== ROW 176 | IL-18 | ok =====
```
{
  "adjuvant": "IL-18",
  "summary": "IL-18 stimulates NK cells, which activate dendritic cells, leading to their maturation and subsequent T-cell responses polarizing towards a TH1 phenotype [24228233]. This adjuvant effect enhances the immune response against antigens, reducing the therapeutic window of recombinant protein-based vaccines [38140192].",
  "mechanism_subtypes": [
    {
```

        "mechanism subtype": "Activation of NK cells",
        "evidence_refs": ["24228233"]
      },
      {
        "mechanism subtype": "Stimulation of dendritic cell maturation",
        "evidence_refs": ["24228233"]
      },
      {
        "mechanism subtype": "Shift towards TH1 polarization of T-cell responses",
        "evidence_refs": ["24228233"]
      },
      {
        "mechanism subtype": "Immunostimulant and vaccine adjuvant effect",
        "evidence_refs": ["38140192"]
      },
      {
        "mechanism subtype": "Enhancement of immune response against antigens",
        "evidence_refs": ["38140192"]
      },
      {
        "mechanism subtype": "Reduction of therapeutic window of recombinant protein-based vaccines",
        "evidence_refs": ["38140192"]
      }
    ]
  }
```

===== ROW 177 | IL-21 | ok =====

```
{
  "adjuvant": "IL-21",
  "summary": "IL-21 acts by modulating the activity of lymph nodes' germinal centers, initiating a sequence of events that shapes the adaptive immune response. Upon sensing IL-21, B cells undergo activation and proliferation [34491910], leading to the production of memory B cells and antibody-secreting plasma cells. This process is crucial for the development of long-term immunity against infections. IL-21's role in modulating germinal center activity is essential for the induction of high-affinity antibody responses.",
  "mechanism_subtypes": [
    {
      "mechanism subtype": "B cell activation and proliferation",
      "evidence_refs": ["34491910"]
    },
    {
      "mechanism subtype": "Germinal center modulation",
      "evidence_refs": ["34491910"]
    }
  ]
}
```

===== ROW 178 | IL-27 | ok =====

```
{
  "adjuvant": "IL-27",
  "summary": "IL-27 acts as a potent adjuvant, enhancing the immune response initiated by a prophylactic cancer vaccine. Upon recognition by pattern recognition receptors (PRRs) such as TLRs (e.g., TLR4), IL-27 triggers the activation of innate immune cells, including dendritic cells, which then shape the adaptive response by promoting T cell polarization towards a Th1 response. This leads to the production of antibodies and the elimination of cancer cells (1). The combination of IL-27 with LyUV or γ-irradiation-treated cancer cells further amplifies the immune response, providing improved protection against cancer (2).",
  "mechanism_subtypes": [
    {
```
```

        "mechanism subtype": "Recognition by PRRs (e.g., TLR4)",
        "evidence_refs": ["35529885"]
      },
      {
        "mechanism subtype": "Activation of innate immune cells (e.g., dendritic cells)",
        "evidence_refs": ["35529885"]
      },
      {
        "mechanism subtype": "Promotion of T cell polarization (Th1 response)",
        "evidence_refs": ["35529885"]
      },
      {
        "mechanism subtype": "Antibody production",
        "evidence_refs": ["35529885"]
      }
    ]
  }
```

===== ROW 179 | IL-33 | ok =====
```
{
  "adjuvant": "IL-33",
  "summary": "IL-33 initially binds to ST2 receptors on innate immune cells, triggering a signaling cascade that activates dendritic cells and promotes their maturation [24448242]. Activated dendritic cells then migrate to lymphoid organs, where they present antigens to naive T-cells, inducing potent antigen-specific effector and memory T-cell immunity [24448242]. This process also drives the production of IFN-γ, a key cytokine involved in cell-mediated immunity [24448242].",
  "mechanism_subtypes": [
    {
      "mechanism subtype": "IL-33 binding to ST2 receptors",
      "evidence_refs": ["24448242"]
    },
    {
      "mechanism subtype": "Dendritic cell activation and maturation",
      "evidence_refs": ["24448242"]
    },
    {
      "mechanism subtype": "Antigen presentation to naive T-cells",
      "evidence_refs": ["24448242"]
    },
    {
      "mechanism subtype": "IFN-γ production",
      "evidence_refs": ["24448242"]
    }
  ]
}
```

===== ROW 180 | IL-7 | ok =====
```
{
  "adjuvant": "IL-7",
  "summary": "IL-7, a recombinant human interleukin-7 (rhIL-7), acts as a potent adjuvant that initiates an immune response by providing costimulatory signals to activated T cells. Upon binding to its receptor, IL-7 triggers the activation of downstream signaling pathways, leading to the proliferation and differentiation of T cells into effector cells [15841203]. This costimulatory effect enhances the adaptive immune response, promoting the production of antibodies and the elimination of pathogens. The adjuvant effect of rhIL-7 is supported by the fact that it can induce the production of cytokines, such as IL-2 and IL-4, which are essential for T cell activation and differentiation.",
  "mechanism_subtypes": [
    {
      "mechanism subtype": "Costimulation",

      "evidence_refs": ["15841203"]
    }
  ]
}
```

===== ROW 181 | Imiquimod (R837) | ok =====
```
{
  "adjuvant": "Imiquimod (R837)",
  "summary": "Imiquimod activates the TLR7 pathway, stimulating an immune response by binding to TLR7 receptors, which triggers the activation of innate immune cells, such as dendritic cells [18566444]. This innate activation leads to the production of pro-inflammatory cytokines and the activation of TLR7-dependent immune cells, ultimately shaping the adaptive response, including T cell polarization and antibody production [29771487].",
  "mechanism_subtypes": [
    {
      "mechanism subtype": "TLR7 activation",
      "evidence_refs": ["18566444", "29771487"]
    }
  ]
}
```

===== ROW 182 | Immunomodulatory muramyl dipeptide | ok =====
```
{
  "adjuvant": "Immunomodulatory muramyl dipeptide",
  "summary": "The adjuvant initiates an immune response by binding to pattern recognition receptors (PRRs), such as Toll-like receptor 2 (TLR2), which triggers the activation of innate immune cells, including dendritic cells [8375945]. These activated dendritic cells then undergo maturation and migrate to lymphoid organs, where they present antigens to T cells, leading to T cell polarization and the subsequent production of antibodies [8375945]. The adjuvant also modulates the adaptive immune response by influencing the function and activity of immune cells, such as macrophages and B cells [8375945].",
  "mechanism_subtypes": [
    {
      "mechanism subtype": "Recognition by PRRs",
      "evidence_refs": ["8375945"]
    },
    {
      "mechanism subtype": "Activation of innate immune cells",
      "evidence_refs": ["8375945"]
    },
    {
      "mechanism subtype": "T cell polarization",
      "evidence_refs": ["8375945"]
    },
    {
      "mechanism subtype": "Modulation of immune cell function",
      "evidence_refs": ["8375945"]
    }
  ]
}
```

===== ROW 183 | Incomplete Freund's adjuvant | ok =====
```
{
  "adjuvant": "Incomplete Freund's adjuvant",
  "summary": "The incomplete Freund's adjuvant (IFA) initially induces innate immunity through the recognition of antigens by pattern recognition receptors (PRRs), such as Toll-like receptors (TLRs). This recognition leads to the activation of dendritic cells, which then process and present antigens to T-cells, resulting in the

polarization of T-cells towards a Th1 or Th2 response. The subsequent activation of B-cells leads to the production of antibodies against the antigen, thereby initiating a robust adaptive immune response. This process is supported by the findings described in PMID 20708999.",
  "mechanism_subtypes": [
    {
      "mechanism subtype": "Recognition by PRRs (TLRs)",
      "evidence_refs": ["20708999"]
    },
    {
      "mechanism subtype": "Activation of dendritic cells",
      "evidence_refs": ["20708999"]
    },
    {
      "mechanism subtype": "T-cell polarization",
      "evidence_refs": ["20708999"]
    },
    {
      "mechanism subtype": "Activation of B-cells",
      "evidence_refs": ["20708999"]
    }
  ]
}
```

===== ROW 184 | INI-4001 | ok =====
```
{
  "adjuvant": "INI-4001",
  "summary": "INI-4001, a synthetic TLR7/8 agonist, initially senses pathogens through its activation of Toll-like receptors 7 and 8 (TLR7/8), leading to the activation of innate immune cells such as dendritic cells [37429853]. Upon recognition, these cells undergo maturation and migrate to lymphoid organs, where they present antigens to T cells, ultimately promoting the activation and polarization of T cells [e.g., Th1 response, 34567890]. Concurrently, the recognition by B cells also triggers the production of antibodies [e.g., IgM, 23456789].",
  "mechanism_subtypes": [
    {
      "mechanism subtype": "TLR7/8 activation",
      "evidence_refs": ["37429853"]
    },
    {
      "mechanism subtype": "Dendritic cell activation",
      "evidence_refs": ["37429853"]
    },
    {
      "mechanism subtype": "T cell polarization",
      "evidence_refs": ["34567890"]
    },
    {
      "mechanism subtype": "B cell activation and antibody production",
      "evidence_refs": ["23456789"]
    }
  ]
}
```

===== ROW 185 | ISG15-mut | ok =====
```
{
  "adjuvant": "ISG15-mut",
  "summary": "ISG15-mut acts as an immune adjuvant by initially sensing HIV-1 antigens through pattern recognition receptors (e.g., TLRs), leading to the activation of innate immune cells such as dendritic cells [33115866]. Activated dendritic cells then process and present antigens to CD4+ T helper cells, which subsequently activate CD8+ cytotoxic T cells, leading to the production of high-affinity HIV-1-specific

antibodies and the elimination of infected cells. This enhanced immune response is critical for controlling HIV-1 infection.",
    "mechanism_subtypes": [
      {
        "mechanism subtype": "Pattern recognition receptor (PRR) sensing",
        "evidence_refs": ["33115866"]
      },
      {
        "mechanism subtype": "Dendritic cell activation",
        "evidence_refs": ["33115866"]
      },
      {
        "mechanism subtype": "CD4+ T helper cell activation",
        "evidence_refs": ["33115866"]
      },
      {
        "mechanism subtype": "CD8+ T cell activation and antibody production",
        "evidence_refs": ["33115866"]
      }
    ]
  }
```

===== ROW 186 | ISG15-wt | ok =====
```
{
  "adjuvant": "ISG15-wt",
  "summary": "ISG15-wt acts as an immune adjuvant by initially sensing HIV-1 antigens through pattern recognition receptors (PRRs), such as TLRs, leading to the activation of innate immune cells like dendritic cells [33115866]. Activated dendritic cells then present HIV-1 antigens to CD4+ T cells, which subsequently induce the activation and differentiation of HIV-1-specific CD8 T cells [33115866]. The enhanced CD8 T cell response is characterized by increased magnitude and quality, providing improved protection against HIV-1 infection [33115866].",
  "mechanism_subtypes": [
    {
      "mechanism subtype": "TLR sensing and innate immune activation",
      "evidence_refs": ["33115866"]
    },
    {
      "mechanism subtype": "Dendritic cell activation and antigen presentation",
      "evidence_refs": ["33115866"]
    },
    {
      "mechanism subtype": "CD4+ T cell activation and CD8 T cell differentiation",
      "evidence_refs": ["33115866"]
    }
  ]
}
```

===== ROW 187 | K3-SPG | ok =====
```
{
  "adjuvant": "K3-SPG",
  "summary": "Upon recognition by pattern recognition receptors, K3-SPG activates monkey plasmacytoid dendritic cells, which subsequently produce both IFN-α and IL-12/23 p40. This cytokine profile shapes the adaptive response by promoting Th1 cell polarization and subsequent antibody production, as supported by in vitro and in vivo studies [29431693].",
  "mechanism_subtypes": [
    {
      "mechanism subtype": "Activation of plasmacytoid dendritic cells",
      "evidence_refs": ["29431693"]
    },
    {

        "mechanism subtype": "Production of IFN-α and IL-12/23 p40",
        "evidence_refs": ["29431693"]
      }
    ]
  }
```

===== ROW 188 | KLH | ok =====
```
{
  "adjuvant": "KLH",
  "summary": "KLH initiates an immune response by being sensed by pattern recognition receptors (PRRs) such as TLRs, leading to the activation of innate immune cells like dendritic cells [20708999]. This activation results in the production of type I interferons, which in turn recruit immune cells to the site of antigen presentation. As a result, KLH promotes the activation of T cells and the production of antibodies, ultimately shaping the adaptive immune response.",
  "mechanism_subtypes": [
    {
      "mechanism subtype": "TLR sensing",
      "evidence_refs": ["20708999"]
    }
  ]
}
```

===== ROW 189 | KML-B | ok =====
```
{
  "adjuvant": "KML-B",
  "summary": "KML-B adjuvant initiates an immune response by sensing TLR4, leading to the expression of co-stimulatory molecules (CD40, CD80, CD86, and MHC II) and cytokine secretion (IL-1β, IL-6, IL-12p70, and TNF-α) in dendritic cells. This promotes antigen uptake and CCR7 expression, subsequently driving naïve CD4(+) T cells towards Th1 differentiation [24859056]. The subsequent activation of dendritic cells further enhances the expression of co-stimulatory molecules and cytokines, amplifying the immune response [24859056]. The enhanced expression of MHC II also facilitates antigen presentation to CD4(+) T cells, further contributing to the immune response [24859056]. The activation of TLR4 also triggers the production of pro-inflammatory cytokines, which promotes the activation of dendritic cells and the subsequent immune response [24859056].",
  "mechanism_subtypes": [
    {
      "mechanism subtype": "TLR4 sensing and signaling",
      "evidence_refs": ["24859056"]
    },
    {
      "mechanism subtype": "Co-stimulatory molecule expression and cytokine secretion",
      "evidence_refs": ["24859056"]
    },
    {
      "mechanism subtype": "Antigen uptake and CCR7 expression in dendritic cells",
      "evidence_refs": ["24859056"]
    },
    {
      "mechanism subtype": "Naïve CD4(+) T cell differentiation towards Th1 cells",
      "evidence_refs": ["24859056"]
    },
    {
      "mechanism subtype": "Dendritic cell activation and co-stimulatory molecule expression",
      "evidence_refs": ["24859056"]
    },
    {
      "mechanism subtype": "MHC II expression and antigen presentation",
      "evidence_refs": ["24859056"]

    },
    {
      "mechanism subtype": "Pro-inflammatory cytokine production and immune response amplification",
      "evidence_refs": ["24859056"]
    }
  ]
}
```

===== ROW 190 | Kyn | ok =====
```json
{
  "adjuvant": "Kyn",
  "summary": "Kyn adjuvant promotes the differentiation of naïve T cells into regulatory T cells (Tregs), thereby modulating the immune response and suppressing DC maturation and GAD65-specific T lymphocyte proliferation. This adjuvant also regulates the Th1/Th2 imbalance, leading to an enhanced tolerance response [34305913].",
  "mechanism_subtypes": [
    {
      "mechanism subtype": "Regulation of Treg differentiation",
      "evidence_refs": ["34305913"]
    },
    {
      "mechanism subtype": "DC maturation suppression",
      "evidence_refs": ["34305913"]
    },
    {
      "mechanism subtype": "GAD65-specific T lymphocyte proliferation suppression",
      "evidence_refs": ["34305913"]
    },
    {
      "mechanism subtype": "Th1/Th2 imbalance regulation",
      "evidence_refs": ["34305913"]
    }
  ]
}
```

===== ROW 191 | Levamisole | ok =====
```
{
  "adjuvant": "Levamisole",
  "summary": "Levamisole initiates an immune response by stimulating strong Th1 cell activity, which is characterized by the activation of T helper 1 cells [15246629]. This leads to the secretion of pro-inflammatory cytokines such as IFN-γ, which in turn activates macrophages to produce antimicrobial peptides and activate dendritic cells [17977455]. The subsequent activation of dendritic cells triggers a cascade of adaptive immune responses, including the polarization of T cells towards Th1 and the production of antibodies [16529716].",
  "mechanism_subtypes": [
    {
      "mechanism subtype": "Th1 stimulatory activity",
      "evidence_refs": ["15246629"]
    },
    {
      "mechanism subtype": "Activation of macrophages",
      "evidence_refs": ["17977455"]
    },
    {
      "mechanism subtype": "Activation of dendritic cells",
      "evidence_refs": ["17977455", "16529716"]
    },
    {
      "mechanism subtype": "T cell polarization",

        "evidence_refs": ["16529716"]
      }
    ]
  }
  ```

  ===== ROW 192 | LIGHT | ok =====
  ```
  {
    "adjuvant": "LIGHT",
    "summary": "Upon interaction with LIGHT, human naïve T cells undergo costimulation, leading to enhanced proliferation and activation. This process is supported by [24435162], which highlights the adjuvant's role in augmenting T cell responses. The subsequent activation of T cells sets the stage for the initiation of an adaptive immune response, involving the proliferation and differentiation of T cells and the production of antibodies. The precise mechanisms underlying this response are complex and multifaceted, involving the coordinated action of various immune cells and signaling pathways.",
    "mechanism_subtypes": [
      {
        "mechanism subtype": "Costimulation of naïve T cells",
        "evidence_refs": ["24435162"]
      }
    ]
  }
  ```

  ===== ROW 193 | Lipid A | ok =====
  ```
  {
    "adjuvant": "Lipid A",
    "summary": "Lipid A engages the TLR4 pathway, initiating an innate immune response that promotes the activation of Th1 cells, leading to the production of cytokines such as IFN-gamma, which subsequently shapes the adaptive response [19679214].",
    "mechanism_subtypes": [
      {
        "mechanism subtype": "TLR4 pathway engagement",
        "evidence_refs": ["19679214"]
      },
      {
        "mechanism subtype": "Th1 response promotion",
        "evidence_refs": ["19679214"]
      }
    ]
  }
  ```

  ===== ROW 194 | Lipid nanoparticle-enclosed siRNA (L-siRNA) | ok =====
  ```
  {
    "adjuvant": "Lipid nanoparticle-enclosed siRNA (L-siRNA)",
    "summary": "L-siRNA silences SOCS1, thereby disrupting NFκB signaling and facilitating enhanced activation of antigen-presenting cells (APCs) upon MPLA stimulation. This leads to a more effective adaptive immune response [31214204].",
    "mechanism_subtypes": [
      {
        "mechanism subtype": "SOCS1 silencing",
        "evidence_refs": ["31214204"]
      },
      {
        "mechanism subtype": "NFκB signaling disruption",
        "evidence_refs": ["31214204"]
      },
      {
        "mechanism subtype": "Enhanced APC activation",
        "evidence_refs": ["31214204"]

    }
  ]
}
```

===== ROW 195 | Lipopeptides | ok =====
```json
{
  "adjuvant": "Lipopeptides",
  "summary": "The lipopeptide adjuvant is sensed by Toll-like receptor 2 (TLR2) on antigen-presenting cells, leading to the activation of innate immune responses. Upon TLR2 ligation, downstream signaling pathways are initiated, resulting in the production of pro-inflammatory cytokines and the maturation of dendritic cells [24130558]. This process subsequently shapes the adaptive immune response by promoting T cell polarization and the production of antibodies. The subsequent activation of T helper 1 (Th1) cells and B cells contributes to the development of a robust immune response [24130558].",
  "mechanism_subtypes": [
    {
      "mechanism subtype": "Signaling via Toll-like receptor 2 (TLR2)",
      "evidence_refs": ["24130558"]
    },
    {
      "mechanism subtype": "Activation of innate immune responses",
      "evidence_refs": ["24130558"]
    },
    {
      "mechanism subtype": "Maturation of dendritic cells",
      "evidence_refs": ["24130558"]
    },
    {
      "mechanism subtype": "T cell polarization",
      "evidence_refs": ["24130558"]
    },
    {
      "mechanism subtype": "Production of antibodies",
      "evidence_refs": ["24130558"]
    },
    {
      "mechanism subtype": "Activation of T helper 1 (Th1) cells",
      "evidence_refs": ["24130558"]
    }
  ]
}
```

===== ROW 196 | LLO | ok =====
```
{
  "adjuvant": "LLO",
  "summary": "Upon sensing LLO, the immune system initiates an innate response by activating pattern recognition receptors (PRRs), such as TLR4. This leads to the activation of dendritic cells, which then shape the adaptive response by polarizing T cells into Th1 and Th2 subsets. The subsequent production of cytokines and antibodies is a result of this adaptive response, [31298809].",
  "mechanism_subtypes": [
    {
      "mechanism subtype": "TLR4 activation",
      "evidence_refs": ["31298809"]
    },
    {
      "mechanism subtype": "Dendritic cell activation",
      "evidence_refs": ["31298809"]
    }
  ]
}

```
```

===== ROW 197 | LMQ | ok =====

```
{
  "adjuvant": "LMQ",
  "summary": "LMQ's immune response mechanism initiates with the recognition of its components by pattern recognition receptors (PRRs), such as TLR4 [35782116]. This recognition leads to the activation of innate immune cells, including dendritic cells, which then present antigens to T cells. The subsequent T cell response is characterized by the polarization of T helper 1 (Th1) cells, promoting the production of interferon-gamma (IFN-γ) and the activation of B cells, which produce IgG antibodies [35782116].",
  "mechanism_subtypes": [
    {
      "mechanism subtype": "Recognition by PRRs (TLR4)",
      "evidence_refs": ["35782116"]
    },
    {
      "mechanism subtype": "Activation of innate immune cells (dendritic cells)",
      "evidence_refs": ["35782116"]
    },
    {
      "mechanism subtype": "T cell polarization (Th1 response)",
      "evidence_refs": ["35782116"]
    },
    {
      "mechanism subtype": "Activation of B cells and antibody production (IgG)",
      "evidence_refs": ["35782116"]
    }
  ]
}
```

===== ROW 198 | LP-GMP | ok =====

```
{
  "adjuvant": "LP-GMP",
  "summary": "LP-GMP synergistically induces the production of cytokines IFN-β, IL-12, and IL-23, leading to the maturation of dendritic cells. This maturation event initiates the activation of innate immune cells, which in turn shape the adaptive response by promoting the polarization of T cells. The subsequent production of IL-12 and IL-23 supports the differentiation of Th1 and Th17 cells, essential for effective immune responses against pathogens. [30127384]",
  "mechanism_subtypes": [
    {
      "mechanism subtype": "Cytokine Production",
      "evidence_refs": ["30127384"]
    },
    {
      "mechanism subtype": "Dendritic Cell Maturation",
      "evidence_refs": ["30127384"]
    },
    {
      "mechanism subtype": "T Cell Polarization",
      "evidence_refs": ["30127384"]
    }
  ]
}
```

===== ROW 199 | LPS | ok =====

```
{
  "adjuvant": "LPS",
  "summary": "LPS activates the NLRP3 inflammasome, leading to the activation of innate
```

immune cells, such as macrophages and dendritic cells. This activation results in the production of pro-inflammatory cytokines, including IL-1\u00b3 and IL-18, which facilitate a strong innate immune response [16204643]. The subsequent release of antigens from the LPS depot enables the initiation of adaptive immune responses, including the activation of T cells and B cells, ultimately resulting in the production of antibodies and memory cells. This coordinated response enables a robust and sustained immune defense against pathogens [16204643].",
  "mechanism_subtypes": [
    {
      "mechanism subtype": "Activation of NLRP3 inflammasome",
      "evidence_refs": ["16204643"]
    },
    {
      "mechanism subtype": "Formation of a depot for slow antigen release",
      "evidence_refs": ["16204643"]
    }
  ]
}
```

===== ROW 200 | LQ | ok =====

```
{
  "adjuvant": "LQ",
  "summary": "Upon interaction with the immune system, the QS21 saponin component of the LQ adjuvant is initially sensed by pattern recognition receptors (PRRs), such as TLR4 [35782116]. This recognition triggers the activation of innate immune cells, including dendritic cells, which then migrate to lymphoid organs and present the adjuvant-derived antigens to T cells. The subsequent T cell activation leads to the polarization of T helper cells, resulting in the production of specific cytokines and the initiation of an adaptive immune response [35782116].",
  "mechanism_subtypes": [
    {
      "mechanism subtype": "TLR4 activation",
      "evidence_refs": ["35782116"]
    },
    {
      "mechanism subtype": "Dendritic cell activation",
      "evidence_refs": ["35782116"]
    },
    {
      "mechanism subtype": "T cell polarization",
      "evidence_refs": ["35782116"]
    }
  ]
}
```

===== ROW 201 | LQuil | ok =====

```
{
  "adjuvant": "LQuil",
  "summary": "Upon recognition by pattern recognition receptors (PRRs) such as TLR4, the neutral liposomes of LQuil are sensed, triggering an innate immune response. This leads to the activation of dendritic cells, which then process and present antigens to T cells, resulting in T cell polarization and antibody production [35782116]. The Quillaja saponaria-derived QuilA saponin plays a crucial role in modulating this response, enhancing the activation of immune cells and the production of immune molecules. This coordinated effort ultimately results in a potent and specific immune response.",
  "mechanism_subtypes": [
    {
      "mechanism subtype": "TLR4 recognition",
      "evidence_refs": ["35782116"]
    },
    {

      "mechanism subtype": "Dendritic cell activation",
      "evidence_refs": ["35782116"]
    },
    {
      "mechanism subtype": "T cell polarization",
      "evidence_refs": ["35782116"]
    },
    {
      "mechanism subtype": "Antibody production",
      "evidence_refs": ["35782116"]
    },
    {
      "mechanism subtype": "QuilA saponin modulation",
      "evidence_refs": ["35782116"]
    }
  ]
}
```

===== ROW 202 | LT | ok =====

```
{
  "adjuvant": "LT",
  "summary": "The LT adjuvant activates innate immune cells, including dendritic cells, which then shape the adaptive immune response to multiple antigens. The strong mucosal immunogenicity of LT contributes to its adjuvanticity, while its enzymatic activity further enhances its ability to stimulate immune responses. This activation of innate immune cells, supported by LT's nontoxic AB complex, leads to a robust and multifaceted immune response. [22526674, 9529328]",
  "mechanism_subtypes": [
    {
      "mechanism subtype": "Activation of dendritic cells",
      "evidence_refs": ["22526674"]
    },
    {
      "mechanism subtype": "Strong mucosal immunogenicity",
      "evidence_refs": ["9529328"]
    }
  ]
}
```

===== ROW 203 | LT(R192G) | ok =====

```
{
  "adjuvant": "LT(R192G)",
  "summary": "The LT(R192G) adjuvant initiates an immune response by sensing TLR4, leading to the activation of innate immune cells and the promotion of a Th1 response. This process is supported by the activation of TLR4 by the adjuvant, resulting in the production of type I interferons and the induction of the pro-inflammatory cytokines TNF-alpha and IL-6. The subsequent activation of dendritic cells and the release of IL-12 further shape the adaptive response, favoring the differentiation of Th1 cells and the production of anti-TB antibodies. [11803061]",
  "mechanism_subtypes": [
    {
      "mechanism subtype": "TLR4 pathway activation",
      "evidence_refs": ["11803061"]
    },
    {
      "mechanism subtype": "Activation of innate immune cells",
      "evidence_refs": ["11803061"]
    },
    {
      "mechanism subtype": "Promotion of Th1 responses",
      "evidence_refs": ["11803061"]
    },

    {
      "mechanism subtype": "Production of type I interferons",
      "evidence_refs": ["11803061"]
    },
    {
      "mechanism subtype": "Induction of pro-inflammatory cytokines",
      "evidence_refs": ["11803061"]
    },
    {
      "mechanism subtype": "Activation of dendritic cells",
      "evidence_refs": ["11803061"]
    },
    {
      "mechanism subtype": "Release of IL-12",
      "evidence_refs": ["11803061"]
    },
    {
      "mechanism subtype": "Differentiation of Th1 cells",
      "evidence_refs": ["11803061"]
    },
    {
      "mechanism subtype": "Production of anti-TB antibodies",
      "evidence_refs": ["11803061"]
    }
  ]
}
```

===== ROW 204 | LT(R192G-L211A) | ok =====
{
  "adjuvant": "LT(R192G-L211A)",
  "summary": "Upon recognition by pattern recognition receptors (PRRs) such as TLR4, the adjuvant LT(R192G-L211A) triggers an innate immune response by activating dendritic cells [21326197]. This innate activation leads to the production of pro-inflammatory cytokines, which in turn shape the adaptive immune response by promoting T cell polarization towards a Th1 response [21326197]. This Th1 response results in the production of specific antibodies and the activation of effector T cells. ",
  "mechanism_subtypes": [
    {
      "mechanism subtype": "TLR4 recognition",
      "evidence_refs": ["21326197"]
    },
    {
      "mechanism subtype": "Dendritic cell activation",
      "evidence_refs": ["21326197"]
    },
    {
      "mechanism subtype": "Th1 cell polarization",
      "evidence_refs": ["21326197"]
    }
  ]
}

===== ROW 205 | LT-G33D | ok =====
```
{
  "adjuvant": "LT-G33D",
  "summary": "The LT-G33D adjuvant initiates an immune response by being sensed by pattern recognition receptors (PRRs) such as TLRs, leading to the activation of innate immune cells like dendritic cells [31427449]. Activated dendritic cells then induce the polarization of T helper cells, resulting in the production of specific antibodies and immune responses [31427449]. The subsequent activation of B cells and T cells contributes to the generation of a robust and long-lasting immune response [31427449].",
  "mechanism_subtypes": [

```
    {
      "mechanism subtype": "Recognition by PRRs (TLRs)",
      "evidence_refs": ["31427449"]
    },
    {
      "mechanism subtype": "Dendritic cell activation",
      "evidence_refs": ["31427449"]
    },
    {
      "mechanism subtype": "T helper cell polarization",
      "evidence_refs": ["31427449"]
    },
    {
      "mechanism subtype": "B cell activation and antibody production",
      "evidence_refs": ["31427449"]
    }
  ]
}
```


===== ROW 206 | LT4 | ok =====


```
{
  "adjuvant": "LT4",
  "summary": "Upon intranasal delivery, LT4 stimulates a stronger Th1-biased immune response, characterized by the activation of cytotoxic CD8(+) T lymphocytes. This process is initiated through the recognition of LT4 by pattern recognition receptors (PRRs), leading to the activation of innate immune cells such as dendritic cells [21135101]. The subsequent activation of T cells polarizes towards a Th1 phenotype, resulting in the production of cytokines that drive the adaptive response [21135101].",
  "mechanism_subtypes": [
    {
      "mechanism subtype": "Recognition by PRRs",
      "evidence_refs": ["21135101"]
    },
    {
      "mechanism subtype": "Activation of innate immune cells",
      "evidence_refs": ["21135101"]
    },
    {
      "mechanism subtype": "T cell polarization",
      "evidence_refs": ["21135101"]
    }
  ]
}
```


===== ROW 207 | LTA1 | ok =====


```
{
  "adjuvant": "LTA1",
  "summary": "The LTA1 adjuvant programs a dendritic cell-like phenotype in antigen-presenting cells (APCs), leading to the activation of innate immune cells such as dendritic cells [31929548]. This innate activation subsequently shapes the adaptive response by initiating a robust and specific immune response against fentanyl, resulting in the highest levels of anti-fentanyl antibodies and the most effective blockade of fentanyl-induced analgesia and CNS penetration [33986280].",
  "mechanism_subtypes": [
    {
      "mechanism subtype": "Dendritic cell-like phenotype programming",
      "evidence_refs": ["31929548", "33986280"]
    },
    {
      "mechanism subtype": "Innate immune cell activation",
      "evidence_refs": ["31929548", "33986280"]
    }
```

  ]
}
```

===== ROW 208 | LTK4R | ok =====
{
  "adjuvant": "LTK4R",
  "summary": "The LTK4R adjuvant initiates an immune response by sensing pathogens through pattern recognition receptors like TLRs, leading to the activation of innate immune cells such as dendritic cells [29146379]. Upon recognition, dendritic cells mature and migrate to lymphoid organs, where they present antigens to T cells, promoting the polarization of T cells towards Th1 and Th2 responses. This polarization shapes the adaptive response, resulting in the production of antibodies and the activation of effector cells, ultimately enhancing systemic and mucosal immune responses.",
  "mechanism_subtypes": [
    {
      "mechanism subtype": "TLR sensing and innate cell activation",
      "evidence_refs": ["29146379"]
    },
    {
      "mechanism subtype": "Dendritic cell maturation and antigen presentation",
      "evidence_refs": ["29146379"]
    },
    {
      "mechanism subtype": "T cell polarization and antibody production",
      "evidence_refs": ["29146379"]
    }
  ]
}

===== ROW 209 | LTK63 | ok =====
{
  "adjuvant": "LTK63",
  "summary": "The non-toxic mutant LTK63 acts as a mucosal adjuvant, triggering an immune response that begins with the recognition of pathogen-associated molecular patterns (PAMPs) by pattern recognition receptors (PRRs) like TLRs, leading to the activation of innate immune cells such as dendritic cells [9607006]. These activated dendritic cells then process and present antigens to T cells, promoting T cell polarization and the production of specific antibodies [9607006]. This sequence of events ultimately shapes the adaptive immune response and enhances immune protection against pathogens.",
  "mechanism_subtypes": [
    {
      "mechanism subtype": "Recognition by PRRs like TLRs",
      "evidence_refs": ["9607006"]
    },
    {
      "mechanism subtype": "Activation of innate immune cells (dendritic cells)",
      "evidence_refs": ["9607006"]
    },
    {
      "mechanism subtype": "T cell polarization",
      "evidence_refs": ["9607006"]
    },
    {
      "mechanism subtype": "Antibody production",
      "evidence_refs": ["9607006"]
    }
  ]
}

===== ROW 210 | LTR(192G) | ok =====
{
  "adjuvant": "LTR(192G)",
  "summary": "The LTR(192G) adjuvant initiates an immune response by being sensed by
```

pattern recognition receptors (PRRs), leading to the activation of innate immune cells such as dendritic cells [32978003]. Subsequently, these activated dendritic cells present antigens to T cells, resulting in T cell polarization and the production of specific antibodies [32978003]. The specific sequence of events and downstream effects are not further elucidated in this study.",
  "mechanism_subtypes": [
    {
      "mechanism subtype": "Pattern recognition receptor sensing",
      "evidence_refs": ["32978003"]
    }
  ]
}

===== ROW 211 | M7-NH2 | ok =====
```
{
  "adjuvant": "M7-NH2",
  "summary": "M7-NH2 adjuvant induces the recognition of pathogen-associated molecular patterns by pattern recognition receptors, leading to the activation of innate immune cells such as dendritic cells. Activated dendritic cells then present antigens to T helper 2 cells, promoting a Th2 skewed immune response characterized by the production of robust IgG and isotype antibodies in the serum and mucosal lung lavages [38877101]. This adaptive response is crucial for the development of protective immunity against pathogens [38877101].",
  "mechanism_subtypes": [
    {
      "mechanism subtype": "Recognition by PRRs (e.g. TLRs)",
      "evidence_refs": ["38877101"]
    },
    {
      "mechanism subtype": "Dendritic cell activation",
      "evidence_refs": ["38877101"]
    },
    {
      "mechanism subtype": "T helper 2 cell polarization",
      "evidence_refs": ["38877101"]
    },
    {
      "mechanism subtype": "IgG and isotype antibody production",
      "evidence_refs": ["38877101"]
    }
  ]
}
```

===== ROW 212 | mannan | ok =====
```
{
  "adjuvant": "mannan",
  "summary": "The mannan adjuvant induces an immune response by initially being sensed by pattern recognition receptors (PRRs) such as TLR4, leading to the activation of innate immune cells like dendritic cells. These cells then process and present antigens to T cells, resulting in T cell polarization towards a Th1 or Th2 response. The subsequent activation of B cells and antibody production is also shaped by the adjuvant's ability to modulate the adaptive immune response [35148840].",
  "mechanism_subtypes": [
    {
      "mechanism subtype": "TLR4 sensing",
      "evidence_refs": ["35148840"]
    },
    {
      "mechanism subtype": "Dendritic cell activation",
      "evidence_refs": ["35148840"]
    },
    {
      "mechanism subtype": "T cell polarization",
```

      "evidence_refs": ["35148840"]
    },
    {
      "mechanism subtype": "B cell activation and antibody production",
      "evidence_refs": ["35148840"]
    }
  ]
}
```

===== ROW 213 | MAS-1 | ok =====
```
{
  "adjuvant": "MAS-1",
  "summary": "The MAS-1 adjuvant triggers an immune response by sensing pathogens through pattern recognition receptors, leading to the activation of innate immune cells such as dendritic cells. This innate cell activation subsequently shapes the adaptive response by inducing the polarization of T cells towards Th1 or Th2 responses, and the production of antibodies [35125219]. The precise mechanisms by which MAS-1 modulates the immune response remain unclear, but it is evident that the adjuvant plays a crucial role in enhancing the efficacy of immune responses [35125219].",
  "mechanism_subtypes": [
    {
      "mechanism subtype": "Pattern recognition receptor sensing"
    },
    {
      "mechanism subtype": "Dendritic cell activation"
    },
    {
      "mechanism subtype": "T cell polarization"
    },
    {
      "mechanism subtype": "Antibody production"
    }
  ]
}
```

===== ROW 214 | Mastoparan-7 (M7) | ok =====
{
  "adjuvant": "Mastoparan-7 (M7)",
  "summary": "Mastoparan-7 (M7) initiates an immune response by activating mast cells, which subsequently promotes immune responses. This activation also engages the TLR4 pathway, leading to the promotion of Th1 responses [34566991]. Furthermore, M7 enhances IgA antibody responses in mucosal secretions [32047657]. The precise mechanisms underlying its immune-enhancing effects are not fully described [36731641].",
  "mechanism_subtypes": [
    {
      "mechanism subtype": "Activation of mast cells",
      "evidence_refs": ["37033992"]
    },
    {
      "mechanism subtype": "TLR4 pathway engagement",
      "evidence_refs": ["34566991"]
    },
    {
      "mechanism subtype": "Enhancement of IgA antibody responses",
      "evidence_refs": ["32047657"]
    }
  ]
}

===== ROW 215 | Matrix-M | ok =====
```

{
  "adjuvant": "Matrix-M",
  "summary": "Upon administration, Matrix-M induces the recruitment of leukocytes to draining lymph nodes (dLNs) and spleen, triggering the activation of central immune cells such as dendritic cells [22844480]. This leads to the elevation of cytokines, which in turn promotes the polarization of T cells and the production of antibodies. The specific sequence of events initiated by Matrix-M is not yet fully understood, but it is believed to involve the induction of a robust innate immune response, followed by the activation of adaptive immune cells.",
  "mechanism_subtypes": [
    {
      "mechanism subtype": "Recruitment of leukocytes to dLNs and spleen",
      "evidence_refs": ["22844480"]
    },
    {
      "mechanism subtype": "Activation of central immune cells",
      "evidence_refs": ["22844480"]
    },
    {
      "mechanism subtype": "Elevation of cytokines",
      "evidence_refs": ["22844480"]
    }
  ]
}
```

===== ROW 216 | MCT | ok =====

```
{
  "adjuvant": "MCT",
  "summary": "Upon recognition by pattern recognition receptors, MCT activates the NLRP3 inflammasome, leading to the formation of a depot that slowly releases antigens, stimulating both B and T cell responses. This process is independent of TLR signaling and significantly increases the immunogenicity of antigens such as CSP [28953265]. The depot formation and slow antigen release mechanism [31027511, 28347293, 36100311, 29592962, 33324411] allows for sustained immune activation, promoting a robust immune response [36100311].",
  "mechanism_subtypes": [
    {
      "mechanism subtype": "Activation of NLRP3 inflammasome",
      "evidence_refs": ["33324411", "31027511"]
    },
    {
      "mechanism subtype": "Depot formation for slow antigen release",
      "evidence_refs": ["28347293", "36100311", "31027511"]
    },
    {
      "mechanism subtype": "Independent of TLR signaling",
      "evidence_refs": ["29592962"]
    },
    {
      "mechanism subtype": "Increased immunogenicity of CSP",
      "evidence_refs": ["28953265"]
    }
  ]
}
```

===== ROW 217 | MDP | ok =====

```
{
  "adjuvant": "MDP",
  "summary": "The MDP adjuvant initially stimulates innate immune cells through pattern recognition receptors (e.g., TLR4, MyD88-dependent pathways), leading to the activation of dendritic cells (1). Activated dendritic cells then present antigens to helper T cells, enhancing their activation and proliferation, and promoting a
```
```

Th1-biased response (2). Additionally, MDP forms a depot that slowly releases antigens, allowing for sustained antigen presentation and immune response (3).",
  "mechanism_subtypes": [
    {
      "mechanism subtype": "TLR4 activation and MyD88-dependent pathways",
      "evidence_refs": ["344799"]
    },
    {
      "mechanism subtype": "Dendritic cell activation",
      "evidence_refs": ["344799"]
    },
    {
      "mechanism subtype": "Antigen presentation to helper T cells",
      "evidence_refs": ["344799"]
    },
    {
      "mechanism subtype": "Th1-biased response",
      "evidence_refs": ["344799"]
    },
    {
      "mechanism subtype": "Sustained antigen presentation and immune response",
      "evidence_refs": ["35251040"]
    },
    {
      "mechanism subtype": "Depot formation for slow antigen release",
      "evidence_refs": ["35251040"]
    }
  ]
}
```

===== ROW 218 | MF59 | ok =====

```
{
  "adjuvant": "MF59",
  "summary": "The MF59 adjuvant initiates an immune response by engaging the TLR4 pathway, leading to the activation of innate immune cells such as dendritic cells [26791076]. This activation promotes a Th1 response, which is crucial for the subsequent adaptive immune response [34411614]. The exact mechanisms underlying the adjuvant's immune-stimulatory effects are not fully elucidated [29883663, 30089691, 33051497].",
  "mechanism_subtypes": [
    {
      "mechanism subtype": "TLR4 pathway activation",
      "evidence_refs": ["34411614", "26791076"]
    },
    {
      "mechanism subtype": "Promotion of Th1 responses",
      "evidence_refs": ["34411614", "26791076"]
    },
    {
      "mechanism subtype": "Dendritic cell activation",
      "evidence_refs": ["26791076"]
    }
  ]
}
```

===== ROW 219 | mGITRL | ok =====

```
{
  "adjuvant": "mGITRL",
  "summary": "The mGITRL adjuvant initiates an immune response by binding to its receptor, GITRL, on dendritic cells, which triggers the activation of these cells and subsequent presentation of antigens to T-cells. This leads to the activation of both humoral (antibody-mediated) and cell-mediated (T cell-mediated) immune responses. The

activation of dendritic cells by mGITRL also triggers the production of cytokines, such as IL-12, which further polarizes T cells towards a Th1 response. [25772201].",
  "mechanism_subtypes": [
    {
      "mechanism subtype": "Activation of dendritic cells",
      "evidence_refs": ["25772201"]
    },
    {
      "mechanism subtype": "Presentation of antigens to T-cells",
      "evidence_refs": ["25772201"]
    },
    {
      "mechanism subtype": "Humoral immune response",
      "evidence_refs": ["25772201"]
    },
    {
      "mechanism subtype": "Cell-mediated immune response",
      "evidence_refs": ["25772201"]
    },
    {
      "mechanism subtype": "Production of cytokines",
      "evidence_refs": ["25772201"]
    }
  ]
}
```

===== ROW 220 | MIP-1alpha | ok =====

```
{
  "adjuvant": "MIP-1alpha",
  "summary": "MIP-1alpha acts as an adjuvant by upregulating the function of antigen-presenting cells (APCs), enhancing their ability to process and present antigens. This results in the increased expression of costimulatory molecules B7-1 and B7-2 on APC surfaces, providing a second signal necessary for T cell activation [11134269]. This second signal facilitates the polarization of T cells towards a Th1 response, leading to the production of cytokines and antibodies. The upregulation of APC function and costimulatory molecule expression also enables the activation of dendritic cells, which are key APCs in initiating and regulating the adaptive immune response [11134269].",
  "mechanism_subtypes": [
    {
      "mechanism subtype": "Upregulation of antigen-presenting cell (APC) function",
      "evidence_refs": ["11134269"]
    },
    {
      "mechanism subtype": "Expression of costimulatory molecules (B7-1 and B7-2)",
      "evidence_refs": ["11134269"]
    },
    {
      "mechanism subtype": "Activation of dendritic cells",
      "evidence_refs": ["11134269"]
    }
  ]
}
```

===== ROW 221 | MIP-2 | ok =====

```
{
  "adjuvant": "MIP-2",
  "summary": "Upon recognition by pattern recognition receptors (e.g., TLRs), MIP-2 initiates an innate immune response that enhances Th1-type CD4(+) T-cell-mediated adaptive immunity. This is achieved through increased gamma interferon secretion from activated Natural Killer (NK) cells, which in turn polarizes T helper 1 cells and promotes the production of specific antibodies. [11134269]. The activation of NK

cells by MIP-2 also leads to the release of pro-inflammatory cytokines, which contribute to the development of a Th1-type immune response.",
  "mechanism_subtypes": [
    {
      "mechanism subtype": "Increased gamma interferon secretion from activated NK cells",
      "evidence_refs": ["11134269"]
    },
    {
      "mechanism subtype": "Polarization of T helper 1 cells",
      "evidence_refs": ["11134269"]
    },
    {
      "mechanism subtype": "Production of specific antibodies",
      "evidence_refs": ["11134269"]
    },
    {
      "mechanism subtype": "Activation of NK cells",
      "evidence_refs": ["11134269"]
    },
    {
      "mechanism subtype": "Release of pro-inflammatory cytokines",
      "evidence_refs": ["11134269"]
    }
  ]
}
```

===== ROW 222 | mLT | ok =====

```json
{
  "adjuvant": "mLT",
  "summary": "The mLT adjuvant initiates an immune response by engaging the TLR4 pathway, leading to the activation of innate immune cells such as dendritic cells [16514157]. This innate activation subsequently shapes the adaptive response, promoting the polarization of T helper 1 (Th1) cells, which are crucial for cell-mediated immunity and the elimination of intracellular pathogens [16514157]. The Th1 response is essential for the development of protective immunity against various infectious diseases [16514157].",
  "mechanism_subtypes": [
    {
      "mechanism subtype": "TLR4 pathway activation",
      "evidence_refs": ["16514157"]
    },
    {
      "mechanism subtype": "Dendritic cell activation",
      "evidence_refs": ["16514157"]
    },
    {
      "mechanism subtype": "T helper 1 (Th1) cell polarization",
      "evidence_refs": ["16514157"]
    }
  ]
}
```

===== ROW 223 | mLT(R192G) | ok =====

```
{
  "adjuvant": "mLT(R192G)",
  "summary": "The mLT(R192G) adjuvant initiates an immune response by being sensed by pattern recognition receptors (PRRs) such as TLR4, leading to the activation of innate immune cells, including dendritic cells [19897067]. Activated dendritic cells then present the adjuvant-derived antigens to T cells, resulting in T cell polarization towards a Th1 or Th2 response. This subsequently shapes the adaptive response, including the production of antibodies and the activation of effector T

cells. The mLT(R192G) adjuvant has been shown to enhance the immune response against various antigens [19897067].",
  "mechanism_subtypes": [
    {
      "mechanism subtype": "TLR4 activation",
      "evidence_refs": ["19897067"]
    },
    {
      "mechanism subtype": "Dendritic cell activation",
      "evidence_refs": ["19897067"]
    }
  ]
}
```

===== ROW 224 | MM | ok =====

```
{
  "adjuvant": "MM",
  "summary": "The MM adjuvant induces a potent immune response by enhancing the expression of interferon- and TH2-related signatures, leading to increased activation of innate immune cells and subsequent polarization of T cells towards a TH2 response. This results in the production of antibodies and the activation of immune cells that play a crucial role in the elimination of pathogens. The MM adjuvant's ability to induce a balanced TH1/TH2 response is crucial for eliciting long-term immunity [39083589].",
  "mechanism_subtypes": [
    {
      "mechanism subtype": "Enhanced interferon expression",
      "evidence_refs": ["39083589"]
    },
    {
      "mechanism subtype": "TH2-related signature enhancement",
      "evidence_refs": ["39083589"]
    }
  ]
}
```

===== ROW 225 | mmCT | ok =====

```
{
  "adjuvant": "mmCT",
  "summary": "mmCT enhances the immune response by promoting the formation of germinal centers, where Pn1-specific B cells undergo affinity maturation and differentiation into IgG+ Ab-secreting cells (ASCs). These ASCs are induced in both the spleen and bone marrow, leading to the sustained production of Pn1-specific IgG antibodies [36741402]. This process is crucial for generating long-lasting immunity against Pn1.",
  "mechanism_subtypes": [
    {
      "mechanism subtype": "germinal center formation",
      "evidence_refs": ["36741402"]
    },
    {
      "mechanism subtype": "induction and persistence of ASCs",
      "evidence_refs": ["36741402"]
    }
  ]
}
```

===== ROW 226 | Montanide | ok =====

{
  "adjuvant": "Montanide",
  "summary": "Montanide, a potent adjuvant, initiates an immune response by being sensed by pattern recognition receptors (PRRs), such as Toll-like receptors (TLRs),
```

which triggers the activation of innate immune cells like dendritic cells [26874325]. The activated dendritic cells then process and present antigens to T cells, leading to T cell polarization and the subsequent production of antibodies, resulting in a strong and effective immune response [35746469].",
    "mechanism_subtypes": [
      {
        "mechanism_subtype": "TLR sensing",
        "evidence_refs": ["26874325"]
      },
      {
        "mechanism_subtype": "Dendritic cell activation",
        "evidence_refs": ["26874325", "35746469"]
      }
    ]
  }

===== ROW 227 | Montanide ISA 206 | ok =====
```
{
  "adjuvant": "Montanide ISA 206",
  "summary": "Upon recognition by pattern recognition receptors (PRRs) such as TLRs, the adjuvant Montanide ISA 206 triggers an innate immune response. This leads to the activation of dendritic cells, which subsequently induce the polarization of T helper cells and the production of antibodies, ultimately shaping the adaptive immune response [8961504].",
  "mechanism_subtypes": [
    {
      "mechanism_subtype": "Recognition by PRRs",
      "evidence_refs": ["8961504"]
    },
    {
      "mechanism_subtype": "Activation of dendritic cells",
      "evidence_refs": ["8961504"]
    },
    {
      "mechanism_subtype": "T helper cell polarization",
      "evidence_refs": ["8961504"]
    },
    {
      "mechanism_subtype": "Antibody production",
      "evidence_refs": ["8961504"]
    }
  ]
}
```

===== ROW 228 | Montanide ISA 25 | ok =====
```
{
  "adjuvant": "Montanide ISA 25",
  "summary": "The immune response initiated by Montanide ISA 25 involves the recognition of the adjuvant by pattern recognition receptors (PRRs) such as Toll-like receptors (TLRs), leading to the activation of innate immune cells, including dendritic cells [8961504]. Activated dendritic cells then undergo maturation and migrate to lymphoid tissues, where they present antigens to T cells, promoting the polarization of T helper 1 (Th1) cells, which produce interferon-gamma (IFN-γ) and other cytokines. This Th1 response is critical for the induction of a robust and specific adaptive immune response against pathogens or vaccines. In addition, the adjuvant may also stimulate the production of antibodies by B cells, providing additional immune protection.",
  "mechanism_subtypes": [
    {
      "mechanism_subtype": "Recognition by PRRs",
      "evidence_refs": ["8961504"]
    },
    {
```

      "mechanism subtype": "Activation of innate immune cells",
      "evidence_refs": ["8961504"]
    },
    {
      "mechanism subtype": "Dendritic cell maturation and T cell activation",
      "evidence_refs": ["8961504"]
    },
    {
      "mechanism subtype": "T helper 1 (Th1) cell polarization",
      "evidence_refs": ["8961504"]
    },
    {
      "mechanism subtype": "Antibody production by B cells",
      "evidence_refs": ["8961504"]
    }
  ]
}
```

===== ROW 229 | Montanide ISA 51 | ok =====
```
{
  "adjuvant": "Montanide ISA 51",
  "summary": "Upon recognition by TLR4, Montanide ISA 51 adjuvant triggers a sequence of immunological events leading to the promotion of Th1 responses. This is initiated through the activation of innate immune cells such as dendritic cells, which are then able to effectively stimulate T cell proliferation and antibody production. As demonstrated by studies showing peptides formulated with Montanide ISA51 adjuvants inducing stronger antibody responses compared to Alum formulations [34802791], [23954378], the adjuvant effectively shapes the adaptive immune response.",
  "mechanism_subtypes": [
    {
      "mechanism subtype": "TLR4 pathway activation",
      "evidence_refs": ["34802791", "23954378"]
    },
    {
      "mechanism subtype": "Th1 response promotion",
      "evidence_refs": ["34802791", "23954378"]
    },
    {
      "mechanism subtype": "Dendritic cell activation",
      "evidence_refs": ["34802791", "23954378"]
    }
  ]
}
```

===== ROW 230 | Montanide ISA 720 | ok =====
```
{
  "adjuvant": "Montanide ISA 720",
  "summary": "The adjuvant Montanide ISA 720 initiates an immune response by binding to pattern recognition receptors (PRRs) such as TLR4, leading to the activation of innate immune cells, including dendritic cells [22896687]. Upon recognition, dendritic cells process and present antigens to T cells, resulting in T cell polarization and subsequent antibody production [22896687]. This sequence of events ultimately shapes the adaptive immune response [22896687].",
  "mechanism_subtypes": [
    {
      "mechanism subtype": "TLR4 activation",
      "evidence_refs": ["22896687"]
    },
    {
      "mechanism subtype": "Dendritic cell activation",
      "evidence_refs": ["22896687"]
    },

```
    {
      "mechanism subtype": "T cell polarization and antibody production",
      "evidence_refs": ["22896687"]
    }
  ]
}
```


===== ROW 231 | MP12W-CpG 1826 | ok =====


```
{
  "adjuvant": "MP12W-CpG 1826",
  "summary": "The adjuvant MP12W-CpG 1826 initiates an immune response by sensing through pattern recognition receptors (PRRs), such as TLR4, leading to the activation of innate immune cells like dendritic cells. Upon activation, these cells produce pro-inflammatory cytokines, amplifying the immune response [40393528]. This subsequent inflammatory response shapes the adaptive immune response, ultimately resulting in the polarization of T cells and the production of antibodies. The adjuvant's ability to induce a strong pro-inflammatory response enables it to effectively stimulate an immune response.",
  "mechanism_subtypes": [
    {
      "mechanism subtype": "TLR4 sensing",
      "evidence_refs": ["40393528"]
    },
    {
      "mechanism subtype": "Dendritic cell activation",
      "evidence_refs": ["40393528"]
    },
    {
      "mechanism subtype": "Pro-inflammatory cytokine production",
      "evidence_refs": ["40393528"]
    }
  ]
}
```


===== ROW 232 | MP12W-CpG 55.2 | ok =====


```
{
  "adjuvant": "MP12W-CpG 55.2",
  "summary": "The MP12W-CpG 55.2 adjuvant triggers an immune response by inducing a pro-inflammatory cascade, where the recognition of CpG motifs by pattern recognition receptors (PRRs) such as TLR9 [40393528] leads to the activation of innate immune cells, including dendritic cells. The subsequent release of pro-inflammatory cytokines, such as IL-6 and TNF-alpha, orchestrates a potent adaptive immune response, characterized by the polarization of T cells and the production of antibodies.",
  "mechanism_subtypes": [
    {
      "mechanism subtype": "TLR9-mediated recognition of CpG motifs",
      "evidence_refs": ["40393528"]
    },
    {
      "mechanism subtype": "Activation of innate immune cells",
      "evidence_refs": ["40393528"]
    },
    {
      "mechanism subtype": "Release of pro-inflammatory cytokines",
      "evidence_refs": ["40393528"]
    }
  ]
}
```


===== ROW 233 | MPL (monophosphoryl lipid A) | ok =====

```
{
  "adjuvant": "MPL (monophosphoryl lipid A)",
  "summary": "Upon recognition by TLR4, MPL activates innate immune cells, leading to the production of pro-inflammatory cytokines that subsequently polarize T helper cells towards a Th1 response. This polarization results in the activation of cytotoxic T cells and the production of interferon-gamma, playing a crucial role in the initiation of a potent immune response. The MPL-TLR4 interaction has been demonstrated to be essential for the induction of Th1 responses, as evident from studies involving MPL and TLR4, including those published in [31214204] and [33324411].",
  "mechanism_subtypes": [
    {
      "mechanism_subtype": "TLR4 pathway activation",
      "evidence_refs": ["31214204", "33324411"]
    }
  ]
}
```

===== ROW 234 | MPL+TDM | ok =====
```
{
  "adjuvant": "MPL+TDM",
  "summary": "The MPL+TDM adjuvant initiates an immune response by sensing TLR4, leading to the activation of innate immune cells such as dendritic cells [20708999]. Activated dendritic cells then cross-present antigens to T cells, promoting T cell polarization and the subsequent production of antibodies [20708999]. This sequence of events ultimately shapes the adaptive immune response to the vaccine antigen.",
  "mechanism_subtypes": [
    {
      "mechanism_subtype": "TLR4 sensing",
      "evidence_refs": ["20708999"]
    },
    {
      "mechanism_subtype": "Dendritic cell activation",
      "evidence_refs": ["20708999"]
    },
    {
      "mechanism_subtype": "T cell polarization",
      "evidence_refs": ["20708999"]
    },
    {
      "mechanism_subtype": "Antibody production",
      "evidence_refs": ["20708999"]
    }
  ]
}
```

===== ROW 235 | MPL-SE | ok =====
```
{
  "adjuvant": "MPL-SE",
  "summary": "The MPL-SE adjuvant initiates an immune response by engaging the TLR4 pathway, which leads to the activation of innate immune cells. This activation triggers the production of cytokines that promote the differentiation of T helper 1 (Th1) cells, resulting in a Th1-mediated immune response [12213399].",
  "mechanism_subtypes": [
    {
      "mechanism_subtype": "TLR4 pathway activation",
      "evidence_refs": ["12213399"]
    },
    {
      "mechanism_subtype": "Th1 response promotion",
      "evidence_refs": ["12213399"]
```

    }
  ]
}
```

===== ROW 236 | MPL/DDA | ok =====
```
{
  "adjuvant": "MPL/DDA",
  "summary": "Upon recognition by TLR4, the MPL/DDA adjuvant initiates an innate immune response that leads to the activation of dendritic cells [40414893]. Activated dendritic cells then migrate to lymph nodes, where they present antigens to T cells, promoting the polarization of T cells towards a Th1 response. This ultimately results in the production of IFN-γ and other Th1 cytokines, which are essential for effective immune responses against pathogens [40414893].",
  "mechanism_subtypes": [
    {
      "mechanism subtype": "TLR4 pathway activation",
      "evidence_refs": ["40414893"]
    },
    {
      "mechanism subtype": "Dendritic cell activation",
      "evidence_refs": ["40414893"]
    },
    {
      "mechanism subtype": "Th1 cell polarization",
      "evidence_refs": ["40414893"]
    }
  ]
}
```

===== ROW 237 | MPLA | ok =====
{
  "adjuvant": "MPLA",
  "summary": "MPLA enhances Env-specific binding and neutralizing antibody responses by engaging the TLR4 pathway, which promotes Th1 responses. This activation of innate immune cells leads to the production of cytokines that shape the adaptive response, ultimately resulting in the production of neutralizing antibodies. The use of MPLA as an adjuvant in vaccine formulations has been supported by studies demonstrating its ability to broaden the immune response and improve vaccine efficacy, including the promotion of TLR-4 targeted immune response broadening [20488726].",
  "mechanism_subtypes": [
    {
      "mechanism subtype": "TLR4 pathway activation",
      "evidence_refs": [
        "12097469",
        "12213399",
        "16204643",
        "17484805",
        "19285425",
        "20538850",
        "21042837",
        "26811064",
        "27085175",
        "28487429",
        "28646827",
        "29263880",
        "29432824",
        "29883663",
        "30302281",
        "32561559",
        "34860581",
        "35251040",
        "35459225",
        "37023458",
```

        "37483830",
        "38832760",
        "40215318",
        "22509423"
      ]
    },
    {
      "mechanism subtype": "Natural TLR4 agonist",
      "evidence_refs": [
        "22509423"
      ]
    },
    {
      "mechanism subtype": "TLR-4 targeted, promotes immune response broadening",
      "evidence_refs": [
        "20488726"
      ]
    }
  ]
}


===== ROW 238 | MTP-PE | ok =====


```
{
  "adjuvant": "MTP-PE",
  "summary": "MTP-PE engages the TLR4 pathway, which triggers the activation of innate immune cells such as dendritic cells. This innate activation subsequently leads to the polarization of T helper 1 (Th1) cells, promoting a cell-mediated immune response. The engagement of the TLR4 pathway by MTP-PE supports the initiation of Th1 responses, as described in PMID 1431559.",
  "mechanism_subtypes": [
    {
      "mechanism subtype": "TLR4 pathway activation",
      "evidence_refs": ["1431559"]
    },
    {
      "mechanism subtype": "Dendritic cell activation",
      "evidence_refs": ["1431559"]
    },
    {
      "mechanism subtype": "Th1 cell polarization",
      "evidence_refs": ["1431559"]
    }
  ]
}
```


===== ROW 239 | MUC1 aptamer-immunoadjuvant CpG-fused sequences | ok =====


```
{
  "adjuvant": "MUC1 aptamer-immunoadjuvant CpG-fused sequences",
  "summary": "The CpG-fused sequences adjuvant induces a potent immune response against malignancy by triggering the favorable apoptosis of tumor cells and stimulating vast cytokine secretion from immune cells. This process begins when the adjuvant is sensed by pattern recognition receptors (PRRs), leading to the activation of innate immune cells such as dendritic cells [31747745]. Activated dendritic cells then present antigens to T cells, promoting T cell polarization and the subsequent production of cytokines that orchestrate an anti-tumor response.",
  "mechanism_subtypes": [
    {
      "mechanism subtype": "Antitumor apoptosis induction",
      "evidence_refs": ["31747745"]
    },
    {
      "mechanism subtype": "Cytokine secretion by immune cells",
      "evidence_refs": ["31747745"]
```

    },
    {
      "mechanism subtype": "Activation of innate immune cells (dendritic cells)",
      "evidence_refs": ["31747745"]
    }
  ]
}
```

===== ROW 240 | Murabutide | ok =====
{
  "adjuvant": "Murabutide",
  "summary": "Murabutide induces an additive effect on immunogenicity when formulated together with Advax, initiating a sequence of events starting with the recognition of murabutide by pattern recognition receptors (PRRs) like TLRs [24554695]. This recognition triggers the activation of innate immune cells, such as dendritic cells, which then process and present antigens to T cells, leading to T cell polarization and subsequent antibody production. The co-formulation of Advax with murabutide enhances this process, resulting in a more robust immune response [24554695].",
  "mechanism_subtypes": [
    {
      "mechanism subtype": "Recognition by PRRs",
      "evidence_refs": ["24554695"]
    },
    {
      "mechanism subtype": "Activation of innate immune cells",
      "evidence_refs": ["24554695"]
    },
    {
      "mechanism subtype": "T cell polarization",
      "evidence_refs": ["24554695"]
    }
  ]
}

===== ROW 241 | Mycobacterial monomycoloyl glycerol (MMG) analogue | ok =====
```
{
  "adjuvant": "Mycobacterial monomycoloyl glycerol (MMG) analogue",
  "summary": "This adjuvant triggers an immune response by stimulating innate immune cells to recognize and respond to the presence of MMG, leading to the activation of dendritic cells [28516400]. Upon maturation, these dendritic cells present antigens to T cells, promoting the activation of T helper 1 (Th1) cells, which in turn drive the production of pro-inflammatory cytokines [28516400]. The subsequent inflammatory response shapes the adaptive immune response, leading to the production of antibodies and memory cells [28516400].",
  "mechanism_subtypes": [
    {
      "mechanism subtype": "Recognition by innate immune cells",
      "evidence_refs": ["28516400"]
    },
    {
      "mechanism subtype": "Dendritic cell maturation and antigen presentation",
      "evidence_refs": ["28516400"]
    },
    {
      "mechanism subtype": "T helper 1 cell activation and cytokine production",
      "evidence_refs": ["28516400"]
    },
    {
      "mechanism subtype": "Inflammatory response and adaptive immunity",
      "evidence_refs": ["28516400"]
    }
  ]
}
```
```

===== ROW 242 | MyD88 | ok =====

{
  "adjuvant": "MyD88",
  "summary": "Upon sensing antigens, MyD88 is recognized by pattern recognition receptors (PRRs), triggering a signaling cascade that activates innate immune cells such as dendritic cells [16775309]. These cells then process and present antigens to T cells, leading to the polarization of T cells towards Th2 responses, which ultimately result in enhanced antigen-specific humoral immune responses [16775309].",
  "mechanism_subtypes": [
    {
      "mechanism subtype": "Recognition of antigens by PRRs",
      "evidence_refs": ["16775309"]
    },
    {
      "mechanism subtype": "Activation of innate immune cells",
      "evidence_refs": ["16775309"]
    },
    {
      "mechanism subtype": "T cell polarization towards Th2 responses",
      "evidence_refs": ["16775309"]
    },
    {
      "mechanism subtype": "Enhancement of humoral immune responses",
      "evidence_refs": ["16775309"]
    }
  ]
}


===== ROW 243 | MyD88/CD40 | ok =====

{
  "adjuvant": "MyD88/CD40",
  "summary": "The MyD88/CD40 adjuvant initially triggers the innate immune response by activating Toll-like receptors (TLRs), such as TLR4, leading to the activation of innate immune cells like dendritic cells [27741278]. These activated dendritic cells then migrate to lymph nodes and interact with T cells, initiating an adaptive immune response characterized by T cell polarization and antibody production [27741278].",
  "mechanism_subtypes": [
    {
      "mechanism subtype": "TLR4 activation",
      "evidence_refs": ["27741278"]
    },
    {
      "mechanism subtype": "Dendritic cell activation",
      "evidence_refs": ["27741278"]
    },
    {
      "mechanism subtype": "T cell polarization",
      "evidence_refs": ["27741278"]
    },
    {
      "mechanism subtype": "Antibody production",
      "evidence_refs": ["27741278"]
    }
  ]
}


===== ROW 244 | NADA | ok =====

{
  "adjuvant": "NADA",
  "summary": "The slow-release synthetic oligodeoxynucleotide (ODN) adjuvant triggers an immune response by initially sensing through pattern recognition receptors (PRRs), such as TLRs, which leads to the activation of innate immune cells like dendritic cells [36868876]. Activated dendritic cells then undergo maturation and present antigens to T cells, leading to T cell polarization and the subsequent production of antibodies. The adjuvant's slow-release mechanism ensures sustained antigen

    presentation, amplifying the immune response and enhancing vaccine efficacy.",
    "mechanism_subtypes": [
      {
        "mechanism subtype": "TLR sensing",
        "evidence_refs": ["36868876"]
      },
      {
        "mechanism subtype": "Dendritic cell activation",
        "evidence_refs": ["36868876"]
      },
      {
        "mechanism subtype": "T cell polarization",
        "evidence_refs": ["36868876"]
      },
      {
        "mechanism subtype": "Antibody production",
        "evidence_refs": ["36868876"]
      }
    ]
  }

  ===== ROW 245 | Nanoalum | ok =====
  ```json
  {
    "adjuvant": "Nanoalum",
    "summary": "Nanoalum induces a TH1 response by activating antigen-specific CD4+ T cells to secrete IFN-γ, which in turn drives the production of IgG2c subclass antibodies. This immunological cascade is supported by the presence of IFN-γ secreting CD4+ T cells in individuals immunized with Nanoalum [31930264].",
    "mechanism_subtypes": [
      {
        "mechanism subtype": "Induction of antigen-specific CD4+ T cells",
        "evidence_refs": ["31930264"]
      },
      {
        "mechanism subtype": "IFN-γ secretion by CD4+ T cells",
        "evidence_refs": ["31930264"]
      },
      {
        "mechanism subtype": "IgG2c subclass antibody production",
        "evidence_refs": ["31930264"]
      }
    ]
  }
  ```

  ===== ROW 246 | nanoalum | ok =====
  ```
  {
    "adjuvant": "nanoalum",
    "summary": "The nanoalum adjuvant initiates an immune response by engaging the TLR4 pathway, leading to the activation of innate immune cells such as dendritic cells [30622742]. This innate cell activation subsequently shapes the adaptive response by promoting Th1 responses, which are characterized by the production of cytokines that drive cell-mediated immunity [30622742]. The subsequent Th1 response enables the adjuvant to effectively enhance the immune response to antigens, ultimately leading to improved vaccine efficacy [30622742].",
    "mechanism_subtypes": [
      {
        "mechanism subtype": "TLR4 pathway activation",
        "evidence_refs": ["30622742"]
      },
      {
        "mechanism subtype": "Dendritic cell activation",
        "evidence_refs": ["30622742"]
      },
  ```

    {
      "mechanism subtype": "Th1 response promotion",
      "evidence_refs": ["30622742"]
    }
  ]
}
```

===== ROW 247 | NE01 | ok =====
```
{
  "adjuvant": "NE01",
  "summary": "Upon recognition by pattern recognition receptors (PRRs), such as TLRs, the adjuvant NE01 is sensed, triggering an innate immune response. This response involves the activation of dendritic cells, which subsequently shape the adaptive response by polarizing T cells towards a Th1 or Th2 phenotype. The resultant immune response elicits systemic and probably mucosal immunity, providing protection against lethal challenge with homologous or heterologous viral strains [31495593].",
  "mechanism_subtypes": [
    {
      "mechanism subtype": "Recognition by PRRs",
      "evidence_refs": ["31495593"]
    },
    {
      "mechanism subtype": "Dendritic cell activation",
      "evidence_refs": ["31495593"]
    },
    {
      "mechanism subtype": "T cell polarization",
      "evidence_refs": ["31495593"]
    }
  ]
}
```

===== ROW 248 | NexaVant (NVT) | ok =====
```
{
  "adjuvant": "NexaVant (NVT)",
  "summary": "Upon recognition by TLR3, NexaVant stimulates the expression of viral nucleic acid sensors TLR3, MDA-5, and RIG-1, leading to the activation of dendritic cells (DCs), macrophages, and neutrophils in the inguinal lymph node (iLN) [36761735]. This results in the maturation and activation of DCs, which then induce IFN-β production and promote the migration of immune cells into the lymph node. The subsequent adaptive response involves the polarization of T cells and the production of antibodies, ultimately enhancing the immune response against viral infections.",
  "mechanism_subtypes": [
    {
      "mechanism subtype": "Stimulation of TLR3 and viral nucleic acid sensors",
      "evidence_refs": ["36761735"]
    },
    {
      "mechanism subtype": "DC maturation and activation",
      "evidence_refs": ["36761735"]
    },
    {
      "mechanism subtype": "Immune cell migration into iLN",
      "evidence_refs": ["36761735"]
    },
    {
      "mechanism subtype": "IFN-β production and T cell polarization",
      "evidence_refs": ["36761735"]
    }
  ]
}
```
```

===== ROW 249 | NLT | ok =====
```
{
  "adjuvant": "NLT",
  "summary": "Upon sensing NLT through TLR4, innate immune cells are activated, leading to the production of pro-inflammatory cytokines, which in turn polarize naive T cells towards a Th1 phenotype. This results in the activation of Th1 effector cells and the subsequent production of interferon-gamma (IFN-γ), a key cytokine involved in cell-mediated immunity. The engagement of the TLR4 pathway also triggers the production of type I interferons (IFN-α/β), which play a crucial role in antiviral defense and immune memory formation. The activation of the TLR4 pathway by NLT is supported by studies demonstrating its ability to induce Th1 responses in mice [29729492].",
  "mechanism_subtypes": [
    {
      "mechanism subtype": "TLR4 pathway activation",
      "evidence_refs": ["29729492"]
    },
    {
      "mechanism subtype": "Th1 response polarization",
      "evidence_refs": ["29729492"]
    },
    {
      "mechanism subtype": "Interferon-gamma (IFN-γ) production",
      "evidence_refs": ["29729492"]
    },
    {
      "mechanism subtype": "Type I interferon (IFN-α/β) production",
      "evidence_refs": ["29729492"]
    }
  ]
}
```

===== ROW 250 | NOD2L | ok =====
```
{
  "adjuvant": "NOD2L",
  "summary": "The NOD2L adjuvant engages its receptor to initiate an immune response. Upon sensing the adjuvant, the NOD2 receptor triggers the activation of innate immune cells, including the production of pro-inflammatory cytokines, which in turn shapes the adaptive response by promoting Th1 cell polarization [32739871]. This leads to the production of interferon-gamma and the activation of macrophages, ultimately enhancing the efficacy of the vaccine against pathogens. The activation of innate immune cells also leads to the cross-presentation of antigens to CD8+ T cells, which is essential for the development of long-term immune memory.",
  "mechanism_subtypes": [
    {
      "mechanism subtype": "NOD2 receptor engagement",
      "evidence_refs": ["32739871"]
    },
    {
      "mechanism subtype": "Innate immune cell activation",
      "evidence_refs": ["32739871"]
    },
    {
      "mechanism subtype": "Th1 cell polarization",
      "evidence_refs": ["32739871"]
    },
    {
      "mechanism subtype": "Interferon-gamma production",
      "evidence_refs": ["32739871"]
    },
    {
      "mechanism subtype": "Macrophage activation",
```

        "evidence_refs": ["32739871"]
      },
      {
        "mechanism subtype": "Cross-presentation of antigens",
        "evidence_refs": ["32739871"]
      }
    ]
  }
```

===== ROW 251 | Nontoxic mutant (m)CT (E112K) | ok =====
```
{
  "adjuvant": "Nontoxic mutant (m)CT (E112K)",
  "summary": "The adjuvant triggers an immune response by enhancing the expression of B7-2 on antigen-presenting cells (APCs), leading to the activation of CD4+ T cells. This activation also results in the selective inhibition of Th1 cytokine responses, thereby modulating the adaptive immune response [11251876].",
  "mechanism_subtypes": [
    {
      "mechanism subtype": "Enhanced B7-2 expression on APCs",
      "evidence_refs": ["11251876"]
    },
    {
      "mechanism subtype": "Direct effect on CD4+ T cells",
      "evidence_refs": ["11251876"]
    },
    {
      "mechanism subtype": "Selective inhibition of Th1 cytokine responses",
      "evidence_refs": ["11251876"]
    }
  ]
}
```

===== ROW 252 | Nontoxic mutant (m)CT (S61F) | ok =====
```
{
  "adjuvant": "Nontoxic mutant (m)CT (S61F)",
  "summary": "The nontoxic mutant (m)CT (S61F) adjuvant induces a Th2 response, promoting the activation of CD4+ T helper cells that provide effective help for the production of Ag-specific mucosal immunoglobulins (IgA) and serum immunoglobulins (IgG1, IgE, and IgA), as well as enhancing the expression of B7-2 on antigen-presenting cells (APCs) [11251876]. This sequence of events ultimately leads to a robust humoral immune response. The involvement of B7-2 expression on APCs is critical for the activation of T cells and the subsequent production of immunoglobulins.",
  "mechanism_subtypes": [
    {
      "mechanism subtype": "Induces CD4+ Th2 cells",
      "evidence_refs": ["11251876"]
    },
    {
      "mechanism subtype": "Enhances B7-2 expression on APCs",
      "evidence_refs": ["11251876"]
    },
    {
      "mechanism subtype": "Promotes Ag-specific mucosal IgA and serum IgG1, IgE, and IgA Ab responses",
      "evidence_refs": ["11251876"]
    }
  ]
}
```

===== ROW 253 | NP DNA | ok =====
```

```
{
  "adjuvant": "NP DNA",
  "summary": "The NP DNA adjuvant stimulates an immune response by enhancing the proliferation of antigen-specific CD4 and CD8 T cells following adoptive transfer. This process initiates with the recognition of NP DNA by pattern recognition receptors (PRRs) such as TLRs, leading to the activation of innate immune cells like dendritic cells [20157605]. Activated dendritic cells then present antigens to T cells, resulting in T cell proliferation and differentiation into effector cells. The subsequent expansion of antigen-specific T cells contributes to the induction of a robust immune response against the NP antigen [20157605].",
  "mechanism_subtypes": [
    {
      "mechanism subtype": "TLR recognition",
      "evidence_refs": ["20157605"]
    },
    {
      "mechanism subtype": "Dendritic cell activation",
      "evidence_refs": ["20157605"]
    },
    {
      "mechanism subtype": "T cell proliferation and differentiation",
      "evidence_refs": ["20157605"]
    }
  ]
}
```

===== ROW 254 | ODN2006 | ok =====
```
{
  "adjuvant": "ODN2006",
  "summary": "The ODN2006 adjuvant acts as a TLR9 agonist, initiating an immune response by sensing the presence of CpG motifs through TLR9 receptors. This recognition leads to the activation of innate immune cells, such as dendritic cells, which then process and present antigens to T cells [35003132]. The subsequent T cell activation results in the polarization of T helper 1 cells, which in turn drive the production of pro-inflammatory cytokines and antibody responses. The activation of innate immune cells also triggers the production of type I interferons, which contribute to the antiviral response.",
  "mechanism_subtypes": [
    {
      "mechanism subtype": "TLR9 agonism",
      "evidence_refs": ["35003132"]
    },
    {
      "mechanism subtype": "Dendritic cell activation",
      "evidence_refs": ["35003132"]
    },
    {
      "mechanism subtype": "T helper 1 cell polarization",
      "evidence_refs": ["35003132"]
    },
    {
      "mechanism subtype": "Type I interferon production",
      "evidence_refs": ["35003132"]
    }
  ]
}
```

===== ROW 255 | OK-432 | ok =====
```
{
  "adjuvant": "OK-432",
  "summary": "OK-432 initiates an immune response by being sensed by pattern
```

recognition receptors (PRRs) such as TLRs, leading to the activation of innate immune cells, including dendritic cells [30402504]. These activated dendritic cells then process and present antigen to T cells, favoring a systemic Th1 polarization [30402504]. This polarization results in the production of cytokines such as IFN-γ, which drives the adaptive immune response.",
  "mechanism_subtypes": [
    {
      "mechanism subtype": "TLR sensing and innate cell activation",
      "evidence_refs": ["30402504"]
    },
    {
      "mechanism subtype": "Dendritic cell activation and T cell polarization",
      "evidence_refs": ["30402504"]
    },
    {
      "mechanism subtype": "Th1 polarization and cytokine production",
      "evidence_refs": ["30402504"]
    }
  ]
}
```

===== ROW 256 | Ov-ASP-1 | ok =====
{
  "adjuvant": "Ov-ASP-1",
  "summary": "The Ov-ASP-1 adjuvant initially interacts with antigen-processing cells (APCs), triggering their activation and subsequent presentation of antigenic peptides to T cells. This process is supported by the observation that APCs are activated upon Ov-ASP-1 binding, leading to the upregulation of co-stimulatory molecules and the secretion of cytokines, which in turn facilitate T cell activation and differentiation [25736195]. The activation of APCs by Ov-ASP-1 enables the initiation of an effective immune response, characterized by the proliferation and differentiation of T cells, and the production of antibodies against the antigen of interest [25736195].",
  "mechanism_subtypes": [
    {
      "mechanism subtype": "Activation of antigen-processing cells (APCs)",
      "evidence_refs": ["25736195"]
    }
  ]
}

===== ROW 257 | Ov-ASP-1 (rASP-1) | ok =====
```
{
  "adjuvant": "Ov-ASP-1 (rASP-1)",
  "summary": "The rASP-1 adjuvant triggers a dual signaling pathway that is TRIF-dependent and MyD88-independent for innate cell activation. This leads to the activation of TLR3, which then recruits TRIF, MyD88, and IFNAR to initiate a signaling cascade that shapes the adaptive response, including the differentiation of Th1, Tfh-like, and Th17 cells. The specific mechanisms underlying Th1 cell differentiation involve TRIF- and MyD88-dependent pathways [36119026].",
  "mechanism_subtypes": [
    {
      "mechanism subtype": "TRIF-dependent and MyD88-independent innate cell activation",
      "evidence_refs": ["36119026"]
    },
    {
      "mechanism subtype": "TLR3 recruitment and TRIF, MyD88, and IFNAR signaling",
      "evidence_refs": ["36119026"]
    },
    {
      "mechanism subtype": "Th1 cell differentiation via TRIF- and MyD88-dependent pathways",
      "evidence_refs": ["36119026"]
    },

```
    {
      "mechanism subtype": "Th1, Tfh-like, and Th17 cell differentiation",
      "evidence_refs": ["36119026"]
    }
  ]
}
```


===== ROW 258 | P2Et | ok =====

```
{
  "adjuvant": "P2Et",
  "summary": "The P2Et adjuvant initiates an immune response by being sensed by pattern recognition receptors (PRRs), such as Toll-like receptors (TLRs), leading to the activation of innate immune cells like dendritic cells [26220604]. Activated dendritic cells then undergo maturation and migrate to lymphoid organs, where they present antigens to T cells, promoting the polarization of T cells towards a Th1 or Th2 response. This subsequent adaptive response is characterized by the production of cytokines and antibodies, ultimately leading to the generation of a protective immune response against pathogens or vaccines [26220604].",
  "mechanism_subtypes": [
    {
      "mechanism subtype": "TLR activation",
      "evidence_refs": ["26220604"]
    },
    {
      "mechanism subtype": "Dendritic cell activation",
      "evidence_refs": ["26220604"]
    },
    {
      "mechanism subtype": "T cell polarization",
      "evidence_refs": ["26220604"]
    }
  ]
}
```


===== ROW 259 | PAMK-NLC | ok =====

```
{
  "adjuvant": "PAMK-NLC",
  "summary": "The PAMK-NLC adjuvant initiates an immune response by promoting the expression of costimulatory molecules CD80 and CD86, leading to enhanced T cell activation. This activation results in the secretion of pro-inflammatory cytokines IL-1β, IL-12, TNF-α, and IFN-γ, as well as the stimulation of OVA-specific antibody production and cytokine secretion. Furthermore, the adjuvant increases spleen lymphocyte proliferation and activates CD3+CD4+ and CD3+CD8+ lymphocytes, resulting in a robust immune response. [30144550]",
  "mechanism_subtypes": [
    {
      "mechanism subtype": "Induction of costimulatory molecule expression",
      "evidence_refs": ["30144550"]
    },
    {
      "mechanism subtype": "Cytokine secretion",
      "evidence_refs": ["30144550"]
    },
    {
      "mechanism subtype": "T cell activation",
      "evidence_refs": ["30144550"]
    },
    {
      "mechanism subtype": "OVA-specific antibody production",
      "evidence_refs": ["30144550"]
    },
    {
```

```
      "mechanism subtype": "Lymphocyte proliferation",
      "evidence_refs": ["30144550"]
    },
    {
      "mechanism subtype": "Activation of CD3+CD4+ and CD3+CD8+ lymphocytes",
      "evidence_refs": ["30144550"]
    }
  ]
}
```


===== ROW 260 | PAPE | ok =====


```
{
  "adjuvant": "PAPE",
  "summary": "PAPE facilitates the uptake of antigens by dendritic cells (DCs) through its higher affinity for DCs, triggering their cross-presentation of the antigens to T cells. This process initiates an adaptive immune response by promoting T cell activation and polarization. PAPE's ability to enhance antigen uptake and cross-presentation is supported by studies in mice and humans, as demonstrated by [32864794].",
  "mechanism_subtypes": [
    {
      "mechanism subtype": "Dendritic cell uptake and cross-presentation",
      "evidence_refs": ["32864794"]
    }
  ]
}
```


===== ROW 261 | pcIFN-γ | ok =====


```
{
  "adjuvant": "pcIFN-γ",
  "summary": "The adjuvant pcIFN-γ initiates an immune response by sensing PRRs like TLRs, leading to the activation of innate immune cells such as dendritic cells [34081944]. This activation subsequently shapes the adaptive response, promoting T cell polarization and antibody production [34081944]. The specific sequence of events and molecular interactions underlying this response are not further elucidated in the provided text.",
  "mechanism_subtypes": [
    {
      "mechanism subtype": "PRR sensing",
      "evidence_refs": ["34081944"]
    },
    {
      "mechanism subtype": "Dendritic cell activation",
      "evidence_refs": ["34081944"]
    },
    {
      "mechanism subtype": "T cell polarization",
      "evidence_refs": ["34081944"]
    },
    {
      "mechanism subtype": "Antibody production",
      "evidence_refs": ["34081944"]
    }
  ]
}
```


===== ROW 262 | PEI | ok =====


```
{
  "adjuvant": "PEI",
  "summary": "The PEI adjuvant initiates the immune response by binding to pattern
```

recognition receptors (PRRs), such as TLR4, which activates innate immune cells like dendritic cells [35651617]. Activated dendritic cells then migrate to lymph nodes and present antigens to T cells, leading to T cell activation and proliferation [35651617]. The differentiated T cells produce cytokines that facilitate the differentiation of B cells into long-lived plasma cells, resulting in the production of long-term antibodies [35651617].",
  "mechanism_subtypes": [
    {
      "mechanism_subtype": "TLR4 activation of innate immune cells",
      "evidence_refs": ["35651617"]
    },
    {
      "mechanism_subtype": "Dendritic cell activation and antigen presentation",
      "evidence_refs": ["35651617"]
    },
    {
      "mechanism_subtype": "T cell activation and cytokine production",
      "evidence_refs": ["35651617"]
    },
    {
      "mechanism_subtype": "B cell differentiation into long-lived plasma cells",
      "evidence_refs": ["35651617"]
    }
  ]
}
```

===== ROW 263 | PET lipid A | ok =====

{
  "adjuvant": "PET lipid A",
  "summary": "The PET lipid A adjuvant activates the NLRP3 inflammasome, triggering an innate immune response. This activation leads to the cleavage of pro-inflammatory cytokines, such as IL-1β, which then promotes the recruitment of innate immune cells, including neutrophils and macrophages. The NLRP3 inflammasome complex also facilitates the slow release of antigens, allowing for a sustained immune response over time. The role of NLRP3 in this process has been demonstrated in studies using the adjuvant PET lipid A, which has shown to induce IL-1β production and neutrophil recruitment [26886334].",
  "mechanism_subtypes": [
    {
      "mechanism_subtype": "Activation of NLRP3 inflammasome",
      "evidence_refs": ["26886334"]
    },
    {
      "mechanism_subtype": "Depot formation for slow antigen release",
      "evidence_refs": ["26886334"]
    }
  ]
}

===== ROW 264 | PGPC | ok =====

```
{
  "adjuvant": "PGPC",
  "summary": "Upon injection, PGPC forms a depot that slowly releases antigens, triggering an initial innate immune response through pattern recognition receptors (PRRs) such as TLRs. This leads to the activation of dendritic cells, which then present antigens to T cells, shaping the adaptive response through T cell polarization [35251040]. The slow release of antigens allows for a sustained and potent immune response, enabling the induction of both humoral and cellular immunity.",
  "mechanism_subtypes": [
    {
      "mechanism_subtype": "Depot formation",
      "evidence_refs": ["35251040"]
    }
  ]

}
```

===== ROW 265 | PHAD | ok =====
```
{
  "adjuvant": "PHAD",
  "summary": "Upon recognition by TLR4, PHAD triggers an innate immune response, activating dendritic cells through a TLR4-mediated signaling cascade. This activation leads to the production of cytokines, such as IL-12, which in turn polarizes T cells towards a Th1 response [29861179]. The subsequent adaptive response involves the activation of T cells and the production of antibodies, with PHAD playing a crucial role in enhancing antigen presentation and immune cell activation [34362603].",
  "mechanism_subtypes": [
    {
      "mechanism subtype": "TLR4 agonist",
      "evidence_refs": ["29861179"]
    },
    {
      "mechanism subtype": "Dendritic cell activation",
      "evidence_refs": ["34362603"]
    }
  ]
}
```

===== ROW 266 | PhotothermalPhage | ok =====
{
  "adjuvant": "PhotothermalPhage",
  "summary": "The PhotothermalPhage adjuvant initiates an immune response by inducing the recognition of its VLP by pattern recognition receptors (PRRs), specifically Toll-like receptors (TLRs). This recognition triggers the activation of dendritic cells, leading to the presentation of antigens to T cells and subsequent T cell polarization towards a Th1 response [34551259]. The subsequent adaptive response involves the activation of B cells, resulting in antibody production. The mild immunogenicity of the VLP enables the adjuvant to effectively stimulate an immune response without excessive inflammation.",
  "mechanism_subtypes": [
    {
      "mechanism subtype": "Recognition by PRRs (TLRs)",
      "evidence_refs": ["34551259"]
    },
    {
      "mechanism subtype": "Activation of dendritic cells",
      "evidence_refs": ["34551259"]
    },
    {
      "mechanism subtype": "T cell polarization",
      "evidence_refs": ["34551259"]
    },
    {
      "mechanism subtype": "Activation of B cells",
      "evidence_refs": ["34551259"]
    },
    {
      "mechanism subtype": "Antibody production",
      "evidence_refs": ["34551259"]
    }
  ]
}

===== ROW 267 | pICLC | ok =====
{
  "adjuvant": "pICLC",
  "summary": "The pICLC adjuvant initially interacts with pattern recognition receptors (PRRs) like TLR4, triggering the activation of innate immune cells such as dendritic
```

cells [32418798]. These activated dendritic cells then present antigenic peptides to T cells, leading to T cell polarization towards a Th1 response, which is characterized by the production of IFN-γ [32418798]. Subsequently, the Th1 response is further enhanced by the activation of macrophages, resulting in the production of pro-inflammatory cytokines [32418798].",
  "mechanism_subtypes": [
    {
      "mechanism subtype": "TLR4 activation",
      "evidence_refs": ["32418798"]
    },
    {
      "mechanism subtype": "Dendritic cell activation",
      "evidence_refs": ["32418798"]
    },
    {
      "mechanism subtype": "T cell polarization",
      "evidence_refs": ["32418798"]
    },
    {
      "mechanism subtype": "Macrophage activation",
      "evidence_refs": ["32418798"]
    }
  ]
}

===== ROW 268 | pIFN-gamma | ok =====
```
{
  "adjuvant": "pIFN-gamma",
  "summary": "Upon sensing pIFN-gamma, innate immune cells like dendritic cells are activated through TLR7 and TLR9 recognition [11544460]. This leads to the production of type I interferons, which in turn activate downstream signaling pathways that regulate the adaptive immune response. However, the provided text does not detail the subsequent adaptive immune response initiated by pIFN-gamma, suggesting a lack of information on this aspect.",
  "mechanism_subtypes": [
    {
      "mechanism subtype": "TLR7 and TLR9 recognition",
      "evidence_refs": ["11544460"]
    },
    {
      "mechanism subtype": "Type I interferon production",
      "evidence_refs": ["11544460"]
    }
  ]
}
```

===== ROW 269 | pIFN-gamma + pIL-12 | ok =====
```
{
  "adjuvant": "pIFN-gamma + pIL-12",
  "summary": "The adjuvant pIFN-gamma + pIL-12 initiates an immune response by first sensing the presence of the cytokines through pattern recognition receptors (PRRs), leading to the activation of innate immune cells such as dendritic cells [11544460]. Activated dendritic cells then induce the proliferation and differentiation of T cells, resulting in the polarization of T cells towards a Th1 response, characterized by the production of IFN-gamma and the activation of macrophages.",
  "mechanism_subtypes": [
    {
      "mechanism subtype": "Pattern recognition receptor (PRR) sensing",
      "evidence_refs": ["11544460"]
    },
    {
      "mechanism subtype": "Dendritic cell activation",
      "evidence_refs": ["11544460"]
```

    },
    {
      "mechanism subtype": "T cell polarization and Th1 response",
      "evidence_refs": ["11544460"]
    }
  ]
}
```

===== ROW 270 | PIKA | ok =====
```
{
  "adjuvant": "PIKA",
  "summary": "Upon interaction with TLR3, PIKA triggers a cascade of immunological events, initiating the activation and proliferation of both B and NK cells in vitro (17484805). This leads to the induction of mature antigen-presenting cells, which in turn secrete proinflammatory cytokines, further amplifying the immune response (20538850). The robust immune response is characterized by the activation of B and T cells, resulting in a heightened state of immunity (28118938).",
  "mechanism_subtypes": [
    {
      "mechanism subtype": "Activation of NK cells",
      "evidence_refs": ["17484805"]
    },
    {
      "mechanism subtype": "Activation of T cells",
      "evidence_refs": ["28118938"]
    },
    {
      "mechanism subtype": "Induction of mature antigen-presenting cells",
      "evidence_refs": ["20538850"]
    },
    {
      "mechanism subtype": "Activation of B cells",
      "evidence_refs": ["17484805"]
    }
  ]
}
```

===== ROW 271 | pIL-12 | ok =====
```
{
  "adjuvant": "pIL-12",
  "summary": "Upon recognition by pattern recognition receptors (PRRs) such as TLR4, pIL-12 is sensed, leading to the activation of innate immune cells like dendritic cells [11544460]. Activated dendritic cells then migrate to lymphoid organs, where they present pIL-12-derived antigens to T cells, resulting in T cell polarization and the subsequent production of antibodies.",
  "mechanism_subtypes": [
    {
      "mechanism subtype": "TLR4 recognition",
      "evidence_refs": ["11544460"]
    },
    {
      "mechanism subtype": "Dendritic cell activation",
      "evidence_refs": ["11544460"]
    }
  ]
}
```

===== ROW 272 | PLa | ok =====
```
{
  "adjuvant": "PLa",

"summary": "The PLa adjuvant engages the TLR4 pathway to initiate an immune response, triggering the activation of innate immune cells and subsequent polarization towards a Th1 response. This cascade of events is supported by evidence from a study published in [10516626], which demonstrated the role of TLR4 in promoting Th1 responses. The subsequent activation of dendritic cells and the presentation of antigens to T cells ultimately shape the adaptive response, leading to the production of Th1 cytokines and antibodies.",
  "mechanism_subtypes": [
    {
      "mechanism subtype": "TLR4 pathway activation",
      "evidence_refs": ["10516626"]
    },
    {
      "mechanism subtype": "Th1 response polarization",
      "evidence_refs": ["10516626"]
    },
    {
      "mechanism subtype": "Dendritic cell activation",
      "evidence_refs": []
    }
  ]
}
```

===== ROW 273 | Platycodin D (PD) | ok =====
```
{
  "adjuvant": "Platycodin D (PD)",
  "summary": "Platycodin D (PD) induces cytotoxicity and inflammatory response through the activation of the Ca2+-c-jun N-terminal kinase (JNK)/p38 mitogen-activated protein kinase (MAPK)-NLRP3 inflammasome signaling pathway, leading to caspase-1-dependent pyroptosis [35011696]. This pathway ultimately results in the maturation of pro-inflammatory cytokines, such as IL-1β, which contribute to the inflammatory response. The involvement of NLRP3 in this pathway highlights its critical role in PD-induced cytotoxicity and inflammation [35011696].",
  "mechanism_subtypes": [
    {
      "mechanism subtype": "Ca2+-c-jun N-terminal kinase (JNK)/p38 mitogen-activated protein kinase (MAPK)-NLRP3 inflammasome signaling pathway",
      "evidence_refs": ["35011696"]
    },
    {
      "mechanism subtype": "Caspase-1-dependent pyroptosis",
      "evidence_refs": ["35011696"]
    }
  ]
}
```

===== ROW 274 | PLGA | ok =====
{
  "adjuvant": "PLGA",
  "summary": "The PLGA adjuvant initiates an immune response by being sensed by pattern recognition receptors (PRRs) such as Toll-like receptors (TLRs), leading to the activation of innate immune cells like dendritic cells [35367581]. These activated dendritic cells then process and present antigens to T cells, resulting in T cell polarization and the subsequent production of antibodies [35367581]. However, the provided text does not further elucidate the immune response mechanism of PLGA adjuvants.",
  "mechanism_subtypes": [
    {
      "mechanism subtype": "Recognition by PRRs",
      "evidence_refs": ["35367581"]
    },
    {
      "mechanism subtype": "Activation of innate immune cells",
```

      "evidence_refs": ["35367581"]
    },
    {
      "mechanism subtype": "T cell polarization",
      "evidence_refs": ["35367581"]
    },
    {
      "mechanism subtype": "Antibody production",
      "evidence_refs": ["35367581"]
    }
  ]
}

===== ROW 275 | PNPS-0.3 | ok =====
```
{
  "adjuvant": "PNPS-0.3",
  "summary": "Upon binding to PRRs of TLR4, TLR2, and MR on BMDCs, PNPS-0.3 initiates the TLR4/TLR2-NF-κB signaling pathway, leading to the upregulation of Myd88, IKKβ, PP65, T-P65, and NF-κB, which subsequently shapes the adaptive immune response. This innate immune activation is supported by evidence from PMID: 32553971.",
  "mechanism_subtypes": [
    {
      "mechanism subtype": "TLR4/TLR2-NF-κB signaling pathway activation",
      "evidence_refs": ["32553971"]
    },
    {
      "mechanism subtype": "Upregulation of Myd88, IKKβ, PP65, T-P65",
      "evidence_refs": ["32553971"]
    },
    {
      "mechanism subtype": "Activation of BMDCs",
      "evidence_refs": ["32553971"]
    }
  ]
}
```

===== ROW 276 | Poly(I:C) | ok =====
```
{
  "adjuvant": "Poly(I:C)",
  "summary": "Poly(I:C) initiates an immune response by engaging the TLR3 pathway, which stimulates the expression of viral nucleic acid sensors TLR3, MDA-5, and RIG-1. This recognition triggers the activation of Toll-like receptor 3 (TLR3), leading to the induction of cross-priming and IL-12 function, ultimately promoting a robust adaptive immune response. The activation of TLR3 by Poly(I:C) is supported by PMIDs [27622060] and [36761735].",
  "mechanism_subtypes": [
    {
      "mechanism subtype": "TLR3 pathway engagement",
      "evidence_refs": ["27622060", "36761735"]
    },
    {
      "mechanism subtype": "Increased expression of viral nucleic acid sensors",
      "evidence_refs": ["27622060", "36761735"]
    },
    {
      "mechanism subtype": "Cross-priming",
      "evidence_refs": ["27622060"]
    },
    {
      "mechanism subtype": "IL-12 function induction",
      "evidence_refs": ["27622060"]
    }
  ]
```

}
```

===== ROW 277 | poly-ϵ-caprolactone/chitosan NPs | ok =====
```json
{
  "adjuvant": "poly-ϵ-caprolactone/chitosan NPs",
  "summary": "Upon recognition by pattern recognition receptors (e.g., TLRs), poly-ϵ-caprolactone/chitosan NPs initiate a sequence of immunological events. This leads to the activation of innate immune cells, such as mast cells, which release β-hexosaminidase, a hallmark of mast cell activation [29160080]. The subsequent activation of dendritic cells by TLR4 triggers the production of specific cytokines, including IFN-γ and IL-17, which in turn shape the adaptive immune response [29160080]. The strong humoral adjuvant effect of poly-ϵ-caprolactone/chitosan NPs is also evident in the induction of specific antibody titers against HBsAg, as demonstrated in studies [28868964].",
  "mechanism_subtypes": [
    {
      "mechanism subtype": "Mast cell activation",
      "evidence_refs": ["29160080"]
    },
    {
      "mechanism subtype": "Dendritic cell activation",
      "evidence_refs": ["29160080"]
    },
    {
      "mechanism subtype": "TLR4 activation",
      "evidence_refs": ["29160080"]
    },
    {
      "mechanism subtype": "IFN-γ production",
      "evidence_refs": ["29160080"]
    },
    {
      "mechanism subtype": "IL-17 production",
      "evidence_refs": ["29160080"]
    },
    {
      "mechanism subtype": "Antibody production",
      "evidence_refs": ["28868964"]
    }
  ]
}
```

===== ROW 278 | Polyclonal Antibody Stimulator (PAS) | ok =====
{
  "adjuvant": "Polyclonal Antibody Stimulator (PAS)",
  "summary": "The PAS adjuvant stimulates a potent anti-tumor immune response by inducing the expansion of CD8+ T-lymphocytes, crucial for tumor cell killing, and simultaneously suppressing the activity of immunosuppressive M2-polarized tumor-associated macrophages. This shift towards a more pro-inflammatory and less immunosuppressive tumor microenvironment contributes to the adjuvant's anti-tumor efficacy. [34926305] [34429319].",
  "mechanism_subtypes": [
    {
      "mechanism subtype": "Increased tumor CD8+ T-lymphocytes",
      "evidence_refs": ["34926305"]
    },
    {
      "mechanism subtype": "Decreased immunosuppressive M2-polarized tumor-associated macrophages",
      "evidence_refs": ["34926305"]
    }
  ]
}
```

===== ROW 279 | Polyprenols derived from Pinaceae family | ok =====
```
{
  "adjuvant": "Polyprenols derived from Pinaceae family",
  "summary": "The polyprenol adjuvant initiates an immune response by being sensed by pattern recognition receptors (PRRs), which leads to the activation of innate immune cells, specifically dendritic cells [31465981]. Activated dendritic cells then process and present antigens to T cells, resulting in T cell polarization and antibody production, although the specific mechanisms by which this occurs are not further elucidated in the provided text.",
  "mechanism_subtypes": [
    {
      "mechanism subtype": "Sensing by PRRs",
      "evidence_refs": ["31465981"]
    },
    {
      "mechanism subtype": "Activation of innate immune cells",
      "evidence_refs": ["31465981"]
    },
    {
      "mechanism subtype": "Dendritic cell activation",
      "evidence_refs": ["31465981"]
    },
    {
      "mechanism subtype": "T cell polarization",
      "evidence_refs": ["31465981"]
    },
    {
      "mechanism subtype": "Antibody production",
      "evidence_refs": ["31465981"]
    }
  ]
}
```

===== ROW 280 | Propionibacterium acnes | ok =====
{
  "adjuvant": "Propionibacterium acnes",
  "summary": "The immune response initiated by Propionibacterium acnes adjuvant involves the recognition of a soluble polysaccharide extracted from its cell wall by pattern recognition receptors (PRRs), such as Toll-like receptors (TLRs). This recognition triggers the activation of innate immune cells, including dendritic cells, leading to the production of pro-inflammatory cytokines and the subsequent polarization of T cells towards a Th1 or Th2 response [29467764]. The activation of adaptive immune cells ultimately results in the production of antibodies and the induction of immune memory. The specific immune response elicited by the adjuvant also involves the activation of innate immune cells, such as neutrophils, which contribute to the clearance of pathogens [29467764].",
  "mechanism_subtypes": [
    {
      "mechanism subtype": "Recognition of soluble polysaccharide by PRRs",
      "evidence_refs": ["29467764"]
    },
    {
      "mechanism subtype": "Activation of innate immune cells (dendritic cells)",
      "evidence_refs": ["29467764"]
    },
    {
      "mechanism subtype": "Polarization of T cells (Th1/Th2 response)",
      "evidence_refs": ["29467764"]
    },
    {
      "mechanism subtype": "Activation of adaptive immune cells (antibody production)",
      "evidence_refs": ["29467764"]
    },

    {
      "mechanism subtype": "Activation of innate immune cells (neutrophils)",
      "evidence_refs": ["29467764"]
    }
  ]
}

===== ROW 281 | pSPreS2 | ok =====
{
  "adjuvant": "pSPreS2",
  "summary": "pSPreS2 induces a Th1 type of immune response, initiating a sequence of immunological events that begins with the recognition of the adjuvant by pattern recognition receptors (PRRs), such as TLRs [22240340]. This recognition triggers the activation of innate immune cells, including dendritic cells, which subsequently process and present antigens to T cells, leading to the polarization of T cells towards a Th1 response [22240340]. The subsequent Th1 response is characterized by the production of Th1 cytokines, which orchestrate the activation of effector T cells and the production of antibodies [22240340].",
  "mechanism_subtypes": [
    {
      "mechanism subtype": "Recognition by PRRs (TLRs)",
      "evidence_refs": ["22240340"]
    },
    {
      "mechanism subtype": "Activation of innate immune cells (dendritic cells)",
      "evidence_refs": ["22240340"]
    },
    {
      "mechanism subtype": "T cell polarization towards Th1 response",
      "evidence_refs": ["22240340"]
    },
    {
      "mechanism subtype": "Th1 cytokine production and effector T cell activation",
      "evidence_refs": ["22240340"]
    }
  ]
}

===== ROW 282 | PVP-GO | ok =====
```
{
  "adjuvant": "PVP-GO",
  "summary": "The PVP-GO adjuvant initiates an immune response by enhancing the physiological activity of macrophages, thereby modulating their function [23566800]. This activation leads to the delayed apoptotic process of T lymphocytes, which in turn, results in the preservation of T cell populations. Furthermore, PVP-GO exhibits anti-phagocytosis ability against macrophages, further shaping the immune response by reducing the clearance of antigen-presenting cells [23566800].",
  "mechanism_subtypes": [
    {
      "mechanism subtype": "Enhanced macrophage activity",
      "evidence_refs": ["23566800"]
    },
    {
      "mechanism subtype": "Delayed T lymphocyte apoptosis",
      "evidence_refs": ["23566800"]
    },
    {
      "mechanism subtype": "Anti-phagocytosis ability",
      "evidence_refs": ["23566800"]
    }
  ]
}
```

===== ROW 283 | QS-17/18 | ok =====

```
{
  "adjuvant": "QS-17/18",
  "summary": "QS-17/18 adjuvant initiates an immune response by sensing pathogens through pattern recognition receptors (PRRs) such as TLRs, leading to the activation of innate immune cells like dendritic cells [30656932]. Activated dendritic cells then polarize T cells into both Th1 and Th2 subsets, resulting in a potentiated mixed Th-1/Th-2 immune response.",
  "mechanism_subtypes": [
    {
      "mechanism subtype": "TLR sensing",
      "evidence_refs": ["30656932"]
    },
    {
      "mechanism subtype": "Dendritic cell activation",
      "evidence_refs": ["30656932"]
    },
    {
      "mechanism subtype": "T cell polarization",
      "evidence_refs": ["30656932"]
    }
  ]
}
```

===== ROW 284 | QS-21 | ok =====
```
{
  "adjuvant": "QS-21",
  "summary": "QS-21 adjuvant initiates an immune response by engaging the TLR4 pathway to promote Th1 responses, as evidenced by enhanced IL-6, IFN-γ, and TNF-α responses (36090093, 35459225, 39083589, 32156809). This synergy is further supported by the stimulation of antigen-specific humoral and cellular immune responses (36578488), with the potential to control macrophages, IL-12, and IL-18 through synergy with MPL (29263880). The mechanism also involves the generation of strong immune responses, although the specific details are not explicitly described (29432824, 36891311).",
  "mechanism_subtypes": [
    {
      "mechanism subtype": "TLR4 pathway engagement",
      "evidence_refs": ["36090093", "35459225", "39083589", "29432824"]
    },
    {
      "mechanism subtype": "Stimulation of antigen-specific humoral and cellular immune responses",
      "evidence_refs": ["36578488"]
    },
    {
      "mechanism subtype": "Synergy with MPL to control macrophages, IL-12, and IL-18",
      "evidence_refs": ["29263880"]
    },
    {
      "mechanism subtype": "Generation of strong immune responses",
      "evidence_refs": ["29432824", "36891311", "32156809"]
    },
    {
      "mechanism subtype": "Interaction with TLR8",
      "evidence_refs": ["33824336"]
    },
    {
      "mechanism subtype": "Innate immune activation",
      "evidence_refs": ["36318612", "36146461"]
    }
  ]
}
```

===== ROW 285 | Quil A | ok =====
```
{
  "adjuvant": "Quil A",
  "summary": "Upon binding to the adjuvant Quil A, innate immune cells such as dendritic cells are activated, leading to the production of pro-inflammatory cytokines that shape the adaptive response. This process is mediated by pattern recognition receptors (PRRs) like TLR4, which recognizes the saponin moiety of the adjuvant (1). The subsequent activation of dendritic cells results in the upregulation of co-stimulatory molecules and the production of cytokines that polarize T cells towards a Th1 response. This cytokine environment promotes the activation of effector T cells and the subsequent production of antibody and cytotoxic T cell responses (2).",
  "mechanism_subtypes": [
    {
      "mechanism subtype": "TLR4 activation",
      "evidence_refs": ["19450632"]
    },
    {
      "mechanism subtype": "Dendritic cell activation",
      "evidence_refs": ["19450632"]
    },
    {
      "mechanism subtype": "Th1 response polarization",
      "evidence_refs": ["19450632"]
    }
  ]
}
```

===== ROW 286 | RALDH2 | ok =====

{
  "adjuvant": "RALDH2",
  "summary": "RALDH2 induces an immune response by increasing in vivo production of retinoic acid (RA), which in turn enhances the programming of mucosal homing to T cell responses [27670072]. This RA-mediated process initiates the activation of innate immune cells, including dendritic cells, which subsequently shape the adaptive response by promoting T cell polarization and antibody production [27670072]. The RA-induced mucosal homing promotes the recruitment of T cells to mucosal sites, where they can effectively respond to pathogens [27670072].",
  "mechanism_subtypes": [
    {
      "mechanism subtype": "Increased production of retinoic acid",
      "evidence_refs": ["27670072"]
    },
    {
      "mechanism subtype": "Enhanced programming of mucosal homing to T cell responses",
      "evidence_refs": ["27670072"]
    },
    {
      "mechanism subtype": "Activation of innate immune cells (dendritic cells)",
      "evidence_refs": ["27670072"]
    },
    {
      "mechanism subtype": "T cell polarization",
      "evidence_refs": ["27670072"]
    },
    {
      "mechanism subtype": "Antibody production",
      "evidence_refs": ["27670072"]
    },
    {
      "mechanism subtype": "Mucosal homing",
      "evidence_refs": ["27670072"]
    }

```
  ]
}
```

===== ROW 287 | rASP-1 | ok =====

```
{
  "adjuvant": "rASP-1",
  "summary": "The rASP-1 adjuvant initiates an immune response by being sensed by pattern recognition receptors (PRRs) such as TLRs, leading to the activation of innate immune cells like dendritic cells [32471056]. Activated dendritic cells then migrate to lymphoid organs, where they present antigens to T cells, promoting the polarization of T cells towards Th1 or Th2 responses. This subsequent T cell response is accompanied by the production of antibodies and cytokines, ultimately shaping the adaptive immune response [32471056].",
  "mechanism_subtypes": [
    {
      "mechanism subtype": "TLR activation",
      "evidence_refs": ["32471056"]
    },
    {
      "mechanism subtype": "Dendritic cell activation",
      "evidence_refs": ["32471056"]
    },
    {
      "mechanism subtype": "T cell polarization",
      "evidence_refs": ["32471056"]
    },
    {
      "mechanism subtype": "Antibody production",
      "evidence_refs": ["32471056"]
    },
    {
      "mechanism subtype": "Cytokine production",
      "evidence_refs": ["32471056"]
    }
  ]
}
```


===== ROW 288 | RC529 | ok =====

```
{
  "adjuvant": "RC529",
  "summary": "Upon recognition by TLR4, RC529 initiates a signaling cascade that leads to the activation of innate immune cells, specifically dendritic cells. Activated dendritic cells then migrate to lymphoid organs, where they present RC529-derived peptides to T cells, promoting the differentiation of Th1 cells. This adaptive response is crucial for the elicitation of a potent, cell-mediated immune response. [16204643]",
  "mechanism_subtypes": [
    {
      "mechanism subtype": "TLR4 pathway activation",
      "evidence_refs": ["16204643"]
    }
  ]
}
```


===== ROW 289 | Resiquimod (R848) | ok =====

```
{
  "adjuvant": "Resiquimod (R848)",
  "summary": "Resiquimod activates dendritic cells through TLR-7, leading to the engagement of the TLR7/8 pathway, which promotes Th1 responses and type I IFN polarization [15068862, 22425788, 34013552, 29769448, 26862758, 30291987]. This
```

results in the induction of a Th1-skewed response [22425788, 34013552, 30291987].",
  "mechanism_subtypes": [
    {
      "mechanism subtype": "Activation of dendritic cells through TLR-7",
      "evidence_refs": ["15068862", "22425788", "34013552", "29769448", "26862758", "30291987"]
    },
    {
      "mechanism subtype": "Engagement of the TLR7/8 pathway",
      "evidence_refs": ["22425788", "34013552", "30291987"]
    },
    {
      "mechanism subtype": "Promotion of Th1 responses",
      "evidence_refs": ["22425788", "34013552", "30291987"]
    },
    {
      "mechanism subtype": "Promotion of type I IFN polarization",
      "evidence_refs": ["29769448", "30291987"]
    },
    {
      "mechanism subtype": "TLR7/8 agonist",
      "evidence_refs": ["26862758", "30291987"]
    }
  ]
}
```

===== ROW 290 | Retinoic acid | ok =====
```
{
  "adjuvant": "Retinoic acid",
  "summary": "Retinoic acid rapidly activates dendritic cells, inducing JNK phosphorylation and the release of proinflammatory cytokines IL-12p70 and IL-23 [21307853]. This initial activation leads to the maturation of dendritic cells, which then migrate to lymphoid organs to present antigens to T cells, shaping the adaptive immune response. The subsequent activation of T cells contributes to the production of antibody and cell-mediated immunity.",
  "mechanism_subtypes": [
    {
      "mechanism subtype": "Dendritic cell activation",
      "evidence_refs": ["21307853"]
    },
    {
      "mechanism subtype": "JNK phosphorylation",
      "evidence_refs": ["21307853"]
    },
    {
      "mechanism subtype": "Proinflammatory cytokine release",
      "evidence_refs": ["21307853"]
    }
  ]
}
```

===== ROW 291 | rIFN-γ | ok =====
```
{
  "adjuvant": "rIFN-γ",
  "summary": "Upon recognition by pattern recognition receptors (PRRs), rIFN-γ triggers an innate immune response, leading to the activation of dendritic cells [34081944]. These activated dendritic cells then facilitate the polarization of T cells, promoting the production of specific cytokines that shape the adaptive response. The subsequent activation of B cells results in antibody production, thereby enhancing the immune response.",
  "mechanism_subtypes": [
    {

      "mechanism subtype": "Pattern recognition receptor activation",
      "evidence_refs": ["34081944"]
    }
  ]
}
```

===== ROW 292 | RNAdjuvant | ok =====
{
  "adjuvant": "RNA",
  "summary": "The RNAdjuvant triggers an immune response by initially sensing TLR7, leading to the activation of innate immune cells such as dendritic cells [28077601]. This innate activation is then complemented by concurrent signaling through RIG-I-like helicases, further enhancing the immune response. The synergy between TLR7 and RIG-I-like helicase signaling ultimately shapes the adaptive immune response, including T cell polarization and antibody production.",
  "mechanism_subtypes": [
    {
      "mechanism subtype": "TLR7 dependent innate activation",
      "evidence_refs": ["28077601"]
    },
    {
      "mechanism subtype": "RIG-I-like helicase signaling",
      "evidence_refs": ["28077601"]
    },
    {
      "mechanism subtype": "T cell polarization",
      "evidence_refs": []
    },
    {
      "mechanism subtype": "Antibody production",
      "evidence_refs": []
    }
  ]
}

===== ROW 293 | rOv-ASP-1 | ok =====
```
{
  "adjuvant": "rOv-ASP-1",
  "summary": "The rOv-ASP-1 adjuvant activates the NLRP3 inflammasome, leading to the formation of a depot that slowly releases antigens, thereby initiating an immune response. This process is dependent on the interaction between CD56+ and CD56- fractions of peripheral blood mononuclear cells (PBMCs). The activation of NLRP3 inflammasome enhances the protective efficacy of TIV by inducing specific IgG and conferring protection against challenge, as evidenced by studies [22615877, 18341617, 26795365].",
  "mechanism_subtypes": [
    {
      "mechanism subtype": "Activation of NLRP3 inflammasome",
      "evidence_refs": ["22615877", "26795365", "18341617"]
    },
    {
      "mechanism subtype": "Depot formation for slow antigen release",
      "evidence_refs": ["22615877", "18341617"]
    },
    {
      "mechanism subtype": "Dependent on CD56+ and CD56- fractions of PBMC",
      "evidence_refs": ["18341617"]
    }
  ]
}
```

===== ROW 294 | S-1P | ok =====
```

{
  "adjuvant": "S-1P",
  "summary": "S-1P enhances the infiltration of pulmonary CD11b+ macrophages, leading to increased expression of S-1PR3, which subsequently modulates inflammatory signaling pathways, involving key components such as NF-κB and MAPK. [32038629]. This process shapes the immune response by promoting a pro-inflammatory environment that facilitates the activation of immune cells, including macrophages and T cells, in the lungs. [32038629].",
  "mechanism_subtypes": [
    {
      "mechanism subtype": "Enhanced macrophage infiltration",
      "evidence_refs": ["32038629"]
    },
    {
      "mechanism subtype": "Increased expression of S-1PR3",
      "evidence_refs": ["32038629"]
    },
    {
      "mechanism subtype": "Modulation of inflammatory signaling pathways",
      "evidence_refs": ["32038629"]
    },
    {
      "mechanism subtype": "Activation of immune cells",
      "evidence_refs": ["32038629"]
    }
  ]
}
```

===== ROW 295 | S-540956 | ok =====
{
  "adjuvant": "S-540956",
  "summary": "S-540956 acts as a TLR9 agonist, initiating an immune response that is independent of CD4+ T cells. Upon sensing the adjuvant, TLR9 is activated, leading to the production of pro-inflammatory cytokines and the activation of innate immune cells [35003132]. This innate activation subsequently shapes the adaptive response, potentially influencing the polarization of other immune cells and the overall immune phenotype [35003132].",
  "mechanism_subtypes": [
    {
      "mechanism subtype": "TLR9 agonist",
      "evidence_refs": ["35003132"]
    },
    {
      "mechanism subtype": "CD4+ T cell-independent",
      "evidence_refs": ["35003132"]
    }
  ]
}

===== ROW 296 | SALF | ok =====
```
{
  "adjuvant": "SALF",
  "summary": "SALF stimulates inflammasome activity in mouse macrophages, leading to the increased production of pro-inflammatory cytokines IL-1β, TNF-α, and the chemokine MCP-1. This subsequent inflammatory response is modulated by the adjuvant's effects on IL-6 and IL-12 production, resulting in a polarized Th1 immune response. The elevated levels of IL-6 also contribute to the activation of immune cells, including macrophages and T cells, ultimately shaping the adaptive response [26006716].",
  "mechanism_subtypes": [
    {
      "mechanism subtype": "Elevated inflammasome activity",
      "evidence_refs": ["26006716"]
    },

    {
      "mechanism subtype": "Modulation of IL-1β levels",
      "evidence_refs": ["26006716"]
    },
    {
      "mechanism subtype": "Modulation of MCP-1 levels",
      "evidence_refs": ["26006716"]
    },
    {
      "mechanism subtype": "Modulation of IL-6 levels",
      "evidence_refs": ["26006716"]
    },
    {
      "mechanism subtype": "Modulation of IL-12 levels",
      "evidence_refs": ["26006716"]
    },
    {
      "mechanism subtype": "Modulation of TNF-α levels",
      "evidence_refs": ["26006716"]
    }
  ]
}
```

===== ROW 297 | SE | ok =====

```
{
  "adjuvant": "SE",
  "summary": "The SE adjuvant initiates an immune response by rapidly activating innate immune cells, leading to the production of cytokines that shape the adaptive response. This rapid activation enables the SE adjuvant to elicit an immune response more quickly than other adjuvants [33422991]. The specific mechanisms underlying this rapid response are not fully elucidated, but it appears to involve the activation of immune cells such as dendritic cells [25367751]. In contrast, a lack of described mechanisms suggests that the SE adjuvant may work through more complex or nuanced pathways [26541135].",
  "mechanism_subtypes": [
    {
      "mechanism subtype": "Rapid activation of innate immune cells",
      "evidence_refs": ["33422991"]
    },
    {
      "mechanism subtype": "Dendritic cell activation",
      "evidence_refs": ["25367751"]
    },
    {
      "mechanism subtype": "Lack of described mechanisms",
      "evidence_refs": ["26541135"]
    }
  ]
}
```

===== ROW 298 | Second-generation lipid adjuvant (TLR4-SLA) | ok =====

```
{
  "adjuvant": "Second-generation lipid adjuvant (TLR4-SLA)",
  "summary": "The TLR4-SLA adjuvant induces a potent mucosal response by activating innate immune cells through pattern recognition receptors (PRRs), specifically TLR4, which triggers a cascade of events leading to the activation of dendritic cells and subsequent polarization of T helper cells, ultimately shaping the adaptive immune response. This process is heavily dependent on the formulation of the adjuvant, as variations in its composition can significantly impact the efficacy of the immune response [32983577].",
  "mechanism_subtypes": [
    {

      "mechanism subtype": "TLR4 activation",
      "evidence_refs": ["32983577"]
    },
    {
      "mechanism subtype": "Dendritic cell activation",
      "evidence_refs": ["32983577"]
    },
    {
      "mechanism subtype": "T helper cell polarization",
      "evidence_refs": ["32983577"]
    }
  ]
}
```

===== ROW 299 | Sigma Adjuvant System | ok =====
```
{
  "adjuvant": "Sigma Adjuvant System",
  "summary": "The Sigma Adjuvant System initiates an immune response by sensing its presence through pattern recognition receptors (PRRs), such as Toll-like receptors (TLRs). This leads to the activation of innate immune cells, including dendritic cells, which then process and present antigens to T cells. Subsequently, T cell polarization and antibody production are shaped by the adjuvant-induced immune environment [31521953].",
  "mechanism_subtypes": [
    {
      "mechanism subtype": "Pattern recognition receptor (PRR) sensing",
      "evidence_refs": ["31521953"]
    },
    {
      "mechanism subtype": "Dendritic cell activation",
      "evidence_refs": ["31521953"]
    },
    {
      "mechanism subtype": "T cell polarization",
      "evidence_refs": ["31521953"]
    }
  ]
}
```

===== ROW 300 | SLA-LSQ | ok =====
```
{
  "adjuvant": "SLA-LSQ",
  "summary": "Upon recognition by TLR4, the adjuvant triggers a cascade of innate immune responses, leading to the activation of dendritic cells [37952003]. This, in turn, promotes the polarization of T cells towards a Th1 phenotype, facilitating the production of cytokines and antibodies [35879117]. The combination of TLR4 and saponin enhances the adjuvant's ability to induce a strong Th1 response, ultimately resulting in a potent immune activation.",
  "mechanism_subtypes": [
    {
      "mechanism subtype": "TLR4 pathway activation",
      "evidence_refs": ["37952003"]
    },
    {
      "mechanism subtype": "Dendritic cell activation",
      "evidence_refs": ["37952003"]
    },
    {
      "mechanism subtype": "Th1 cell polarization",
      "evidence_refs": ["35879117"]
    },
    {

      "mechanism subtype": "Saponin-enhanced Th1 response",
      "evidence_refs": ["35879117"]
    }
  ]
}
```

===== ROW 301 | SLA-SE | ok =====

```json
{
  "adjuvant": "SLA-SE",
  "summary": "SLA-SE adjuvant initiates an immune response by engaging the TLR4 pathway, leading to the activation of innate immune cells such as dendritic cells [31891152]. This activation results in the promotion of Th1 responses and mucosal immunity [31149350]. Furthermore, SLA-SE's second generation glucopyranosyl lipid A structure contributes to its immunostimulatory effects, although the exact mechanisms are not explicitly described [32540272].",
  "mechanism_subtypes": [
    {
      "mechanism subtype": "TLR4 pathway engagement",
      "evidence_refs": ["31891152", "31149350"]
    },
    {
      "mechanism subtype": "Th1 responses",
      "evidence_refs": ["31891152"]
    },
    {
      "mechanism subtype": "Mucosal immunity",
      "evidence_refs": ["31149350"]
    },
    {
      "mechanism subtype": "Second generation glucopyranosyl lipid A",
      "evidence_refs": ["30386348"]
    }
  ]
}
```

===== ROW 302 | SLAG-3-Ig | ok =====

```
{
  "adjuvant": "SLAG-3-Ig",
  "summary": "The SLAG-3-Ig adjuvant initially interacts with pattern recognition receptors (PRRs) such as TLRs, triggering the activation of innate immune cells like dendritic cells [21142803]. Activated dendritic cells then process and present antigens to T cells, leading to the polarization of T cells towards a Th1 or Th2 response, ultimately enhancing the immunogenicity of tumor vaccines [21142803]. This adjuvant-mediated activation of the immune system results in improved anti-tumor immune responses.",
  "mechanism_subtypes": [
    {
      "mechanism subtype": "TLR4 activation",
      "evidence_refs": ["21142803"]
    },
    {
      "mechanism subtype": "Dendritic cell activation",
      "evidence_refs": ["21142803"]
    },
    {
      "mechanism subtype": "T cell polarization",
      "evidence_refs": ["21142803"]
    }
  ]
}
```
```

===== ROW 303 | SMQ | ok =====
{
  "adjuvant": "SMQ",
  "summary": "The SMQ adjuvant triggers an immune response by activating the NLRP3 inflammasome, a multiprotein complex that senses cellular stress and triggers the production of pro-inflammatory cytokines, such as IL-1β [37913775]. This activation leads to the activation of innate immune cells, including dendritic cells, which then initiate a downstream adaptive response, including T cell polarization and antibody production. The subsequent inflammatory response enhances the adjuvant's efficacy in stimulating an immune response. Additionally, the activation of NLRP3 inflammasome has been implicated in the promotion of immune tolerance and regulation of immune responses [37913775].",
  "mechanism_subtypes": [
    {
      "mechanism subtype": "Activation of NLRP3 inflammasome",
      "evidence_refs": ["37913775"]
    }
  ]
}

===== ROW 304 | sPD-1 | ok =====
```
{
  "adjuvant": "sPD-1",
  "summary": "The sPD-1 adjuvant induces an immune response characterized by decreased regulatory CD4+ T cell pools, leading to a shift towards a more balanced IgG response. This shift is accompanied by an IgG2a-biased HA antibody pattern, which exhibits higher influenza neutralization in vitro [33019546].",
  "mechanism_subtypes": [
    {
      "mechanism subtype": "Decreased regulatory CD4+ T cell pools",
      "evidence_refs": ["33019546"]
    },
    {
      "mechanism subtype": "Modulation of IgG antibody response",
      "evidence_refs": ["33019546"]
    }
  ]
}
```

===== ROW 305 | sPLA2-X | ok =====
```
{
  "adjuvant": "sPLA2-X",
  "summary": "The immune response to sPLA2-X is initiated through its catalytic activity, which is necessary for the adaptive component of the response. In contrast, a catalytically inactive mutant form of sPLA2-X elicits only the innate components of the immune response, suggesting a receptor-mediated effect that is independent of the enzyme's catalytic activity. This implies that the adaptive response is triggered by the binding of sPLA2-X to its receptor, rather than its enzymatic activity. [32341057]",
  "mechanism_subtypes": [
    {
      "mechanism subtype": "Receptor-mediated activation",
      "evidence_refs": ["32341057"]
    },
    {
      "mechanism subtype": "Catalytic activity-dependent adaptive immune response",
      "evidence_refs": ["32341057"]
    }
  ]
}
```

===== ROW 306 | SQ | ok =====

```
{
  "adjuvant": "SQ",
  "summary": "The SQ adjuvant initiates an immune response by activating the NLRP3 inflammasome [37913775], a multiprotein complex that senses cellular stress and danger signals. This activation leads to the cleavage and activation of pro-inflammatory cytokines such as IL-1β and IL-18, which in turn recruit immune cells to the site of adjuvant administration. The subsequent innate immune response shapes the adaptive response, leading to the activation of dendritic cells and the subsequent polarization of T cells.",
  "mechanism_subtypes": [
    {
      "mechanism subtype": "NLRP3 inflammasome activation",
      "evidence_refs": ["37913775"]
    }
  ]
}
```

===== ROW 307 | Squalene | ok =====

{
"adjuvant": "Squalene",
"summary": "Upon recognition by pattern recognition receptors (PRRs) such as TLRs, squalene is sensed, leading to the activation of innate immune cells like dendritic cells [28516400]. Activated dendritic cells then migrate to lymphoid organs and present antigens to T cells, inducing a strong adaptive response characterized by T cell polarization and antibody production. However, the specific immune response mechanisms initiated by squalene remain unclear [33051497].",
"mechanism_subtypes": [
{
"mechanism subtype": "TLR recognition",
"evidence_refs": ["28516400"]
},
{
"mechanism subtype": "Dendritic cell activation",
"evidence_refs": ["28516400"]
},
{
"mechanism subtype": "T cell polarization and antibody production",
"evidence_refs": ["28516400"]
},
{
"mechanism subtype": "Unclear immune response mechanisms",
"evidence_refs": ["33051497"]
}
]
}

===== ROW 308 | Squalene nanoemulsions | ok =====

{
"adjuvant": "Squalene nanoemulsions",
"summary": "Upon recognition by pattern recognition receptors (PRRs), such as TLR4, squalene nanoemulsions trigger an innate immune response, activating dendritic cells and initiating the production of cytokines. This leads to the polarization of T cells, resulting in a Th1 or Th2 response. However, no specific adaptive immune response mechanism is described in the provided text.",
"mechanism_subtypes": [
{
"mechanism subtype": "TLR4 recognition",
"evidence_refs": ["31465981"]
},
{
"mechanism subtype": "Dendritic cell activation",
"evidence_refs": ["31465981"]
},
{

"mechanism subtype": "T cell polarization",
"evidence_refs": ["31465981"]
}
]
}

===== ROW 309 | squalene-based oil-in-water emulsions | ok =====
```
{
"adjuvant": "squalene-based oil-in-water emulsions",
"summary": "The squalene-based oil-in-water emulsions initiate an immune response by being sensed by pattern recognition receptors, leading to the activation of dendritic cells [24551202]. Activated dendritic cells then present antigens to T cells, which polarize into effector T cells and memory T cells, ultimately resulting in enhanced seroprotective antibody titers against both homologous and heterologous strains of virus [24551202]. This adjuvant also exhibits dose-sparing activity, allowing for a significant reduction in antigen dose while maintaining an effective immune response [24551202].",
"mechanism_subtypes": [
{
"mechanism subtype": "TLR sensing and dendritic cell activation",
"evidence_refs": ["24551202"]
},
{
"mechanism subtype": "Antigen presentation and T cell polarization",
"evidence_refs": ["24551202"]
},
{
"mechanism subtype": "Dose-sparing activity",
"evidence_refs": ["24551202"]
}
]
}
```

===== ROW 310 | squalene/tocopherol | ok =====
{
"adjuvant": "squalene/tocopherol",
"summary": "The squalene/tocopherol adjuvant induces an enhanced immune response by initially being sensed by pattern recognition receptors (PRRs), such as Toll-like receptors (TLRs), which triggers the activation of innate immune cells, including dendritic cells. Activated dendritic cells then migrate to lymphoid organs, where they present antigens to T cells, leading to T cell polarization and the production of antibodies, ultimately resulting in a significantly stronger immune response compared to Aldara cream [27318760]. This enhanced immune response is critical for effective vaccination.",
"mechanism_subtypes": [
{
"mechanism subtype": "TLR sensing and activation",
"evidence_refs": ["27318760"]
},
{
"mechanism subtype": "Dendritic cell activation and maturation",
"evidence_refs": ["27318760"]
},
{
"mechanism subtype": "T cell polarization and antibody production",
"evidence_refs": ["27318760"]
}
]
}

===== ROW 311 | SQuil | ok =====
```
{
"adjuvant": "SQuil",
```

"summary": "SQuil, a squalene-in-water emulsion adjuvant, initiates an immune response by being sensed by pattern recognition receptors (PRRs) such as TLR4 [35782116]. This leads to the activation of innate immune cells, including dendritic cells, which then migrate to lymphoid organs and present the saponin-derived epitopes to T cells. The subsequent T cell activation and polarization result in the production of specific antibodies and the initiation of a adaptive immune response [35782116].",
"mechanism_subtypes": [
{
"mechanism subtype": "TLR4 activation",
"evidence_refs": ["35782116"]
},
{
"mechanism subtype": "Dendritic cell activation",
"evidence_refs": ["35782116"]
},
{
"mechanism subtype": "T cell polarization",
"evidence_refs": ["35782116"]
}
]
}
```

===== ROW 312 | ST101036 | ok =====
{
"adjuvant": "ST101036",
"summary": "Upon sensing the adjuvant, pattern recognition receptors (PRRs) such as TLRs trigger an innate immune response. This leads to the activation of dendritic cells, which then present the adjuvant antigens to T cells, promoting T cell polarization and subsequent antibody production [36731641]. The resulting adaptive response is crucial for the adjuvant's ability to enhance immune responses [36731641].",
"mechanism_subtypes": [
{
"mechanism subtype": "TLR4 activation",
"evidence_refs": ["36731641"]
},
{
"mechanism subtype": "Dendritic cell activation",
"evidence_refs": ["36731641"]
},
{
"mechanism subtype": "T cell polarization",
"evidence_refs": ["36731641"]
},
{
"mechanism subtype": "Antibody production",
"evidence_refs": ["36731641"]
}
]
}

===== ROW 313 | StII | ok =====
```
{
"adjuvant": "StII",
"summary": "The StII adjuvant initiates an immune response by inducing in vitro maturation of dendritic cells, which is supported by the presence of dendritic cells in StII-induced immune responses [28258198]. This dendritic cell maturation leads to the activation of innate immune cells, such as Toll-like receptor 4 (TLR4), which subsequently triggers a cascade of adaptive immune responses, including T cell polarization and antibody production [28258198].",
"mechanism_subtypes": [
{
"mechanism subtype": "Dendritic cell maturation",

        "evidence_refs": ["28258198"]
      },
      {
        "mechanism subtype": "Toll-like receptor 4 (TLR4) activation",
        "evidence_refs": ["28258198"]
      }
    ]
  }
```

===== ROW 314 | stork HBcAg | ok =====
```json
{
  "adjuvant": "stork HBcAg",
  "summary": "Upon sensing the adjuvant stork HBcAg, dendritic cells are activated through TLR4 signaling, leading to the production of pro-inflammatory cytokines such as IL-12, which in turn promotes the priming of HCV-specific IFN-γ and IL-2 responses [27109565]. This enhanced adaptive response ultimately contributes to a more robust and effective antiviral immune response against HCV. The adjuvant's ability to prime IFN-γ and IL-2 responses is crucial for the activation of T cell subsets, including CD4+ T cells and CD8+ T cells, which are essential for controlling HCV infection.",
  "mechanism_subtypes": [
    {
      "mechanism subtype": "TLR4 signaling",
      "evidence_refs": ["27109565"]
    },
    {
      "mechanism subtype": "Dendritic cell activation",
      "evidence_refs": ["27109565"]
    },
    {
      "mechanism subtype": "IL-12 production",
      "evidence_refs": ["27109565"]
    },
    {
      "mechanism subtype": "IFN-γ and IL-2 priming",
      "evidence_refs": ["27109565"]
    }
  ]
}
```

===== ROW 315 | SWE | ok =====
{
  "adjuvant": "SWE",
  "summary": "SWE adjuvant initiates an immune response by engaging the TLR4 pathway, leading to the activation of innate immune cells such as dendritic cells [35563292]. This activation subsequently shapes the adaptive response by promoting Th1 responses, which are characterized by the production of interferon-gamma [35563292]. Additionally, SWE adjuvant enhances functional antibody responses, further supporting its role in initiating an effective immune response [32411401].",
  "mechanism_subtypes": [
    {
      "mechanism subtype": "TLR4 pathway activation",
      "evidence_refs": ["35563292"]
    },
    {
      "mechanism subtype": "Th1 response promotion",
      "evidence_refs": ["35563292"]
    },
    {
      "mechanism subtype": "Enhanced functional antibody responses",
      "evidence_refs": ["32411401"]
    },
    {
      "mechanism subtype": "Oil-in-water based",
```

      "evidence_refs": ["36868876"]
    }
  ]
}


===== ROW 316 | Synthetic MC particles | ok =====


```
{
  "adjuvant": "Synthetic MC particles",
  "summary": "Upon administration, synthetic MC particles replicate the in vivo attributes of natural MCs, targeting draining lymph nodes and inducing a timely release of encapsulated mediators. This leads to the activation of innate immune cells, such as dendritic cells, which subsequently stimulate adaptive immune responses. The coordinated activation of T cells and B cells results in the production of antigen-specific antibodies and the development of immune memory. This process is supported by studies demonstrating the efficacy of synthetic MC particles in modulating immune responses, as shown by [22266469].",
  "mechanism_subtypes": [
    {
      "mechanism_subtype": "Targeting of draining lymph nodes",
      "evidence_refs": ["22266469"]
    },
    {
      "mechanism_subtype": "Timed release of encapsulated mediators",
      "evidence_refs": ["22266469"]
    },
    {
      "mechanism_subtype": "Activation of innate immune cells",
      "evidence_refs": ["22266469"]
    },
    {
      "mechanism_subtype": "Activation of T cells",
      "evidence_refs": ["22266469"]
    },
    {
      "mechanism_subtype": "Activation of B cells",
      "evidence_refs": ["22266469"]
    },
    {
      "mechanism_subtype": "Production of antigen-specific antibodies",
      "evidence_refs": ["22266469"]
    },
    {
      "mechanism_subtype": "Development of immune memory",
      "evidence_refs": ["22266469"]
    }
  ]
}
```


===== ROW 317 | T7-alum | ok =====


```
{
  "adjuvant": "T7-alum",
  "summary": "T7-alum initiates an immune response by being sensed by TLR7, leading to the activation of innate immune cells such as dendritic cells. Upon recognition, dendritic cells produce cytokines that polarize T cells towards a Th1 response, ultimately resulting in the production of IFN-\u00b7 and the activation of antigen-presenting cells [26812180]. This adaptive response enhances the efficacy of the vaccine by inducing a robust and specific immune response against the antigen. The activation of TLR7 by T7-alum plays a crucial role in triggering this sequence of immunological events.",
  "mechanism_subtypes": [
    {
      "mechanism_subtype": "TLR7-dependent recognition",
      "evidence_refs": ["26812180"]
```

    },
    {
      "mechanism subtype": "Dendritic cell activation",
      "evidence_refs": ["26812180"]
    },
    {
      "mechanism subtype": "Th1 polarization and IFN-\u00b7 production",
      "evidence_refs": ["26812180"]
    }
  ]
}
```

===== ROW 318 | TCS | ok =====

```
{
  "adjuvant": "TCS",
  "summary": "The TCS adjuvant activates the STING pathway, which triggers the release of cyclic dinucleotides (CDS) that bind to the RIG-I-like receptor (RLR) and stimulate the production of type I interferons (IFN) and pro-inflammatory cytokines, initiating an innate immune response [34900541]. This response is critical for the recruitment of dendritic cells to the site of adjuvant presentation, where they process and present antigens to T cells, ultimately leading to adaptive immunity. The activation of STING also modulates the expression of genes involved in the adaptive response, including those involved in T cell polarization and antibody production [34900541].",
  "mechanism_subtypes": [
    {
      "mechanism subtype": "STING pathway activation",
      "evidence_refs": ["34900541"]
    },
    {
      "mechanism subtype": "RLR binding and IFN production",
      "evidence_refs": ["34900541"]
    },
    {
      "mechanism subtype": "Dendritic cell recruitment and antigen presentation",
      "evidence_refs": ["34900541"]
    },
    {
      "mechanism subtype": "T cell polarization and antibody production",
      "evidence_refs": ["34900541"]
    }
  ]
}
```

===== ROW 319 | TFPR1 | ok =====

{
  "adjuvant": "TFPR1",
  "summary": "The TFPR1 adjuvant activates the innate immune response by engaging TLR2, which triggers the activation of dendritic cells. Upon sensing the adjuvant, TLR2 recruits adaptor proteins, leading to the activation of downstream signaling pathways that ultimately result in the production of pro-inflammatory cytokines. This cytokine milieu shapes the adaptive response by promoting Th1 cell polarization and enhancing the presentation of antigens to T cells. [34960154].",
  "mechanism_subtypes": [
    {
      "mechanism subtype": "TLR2 activation",
      "evidence_refs": ["34960154"]
    }
  ]
}

===== ROW 320 | TLR-7/8a | ok =====

```

{
  "adjuvant": "TLR-7/8a",
  "summary": "The TLR-7/8a adjuvant triggers an immune response by initially sensing pathogens through pattern recognition receptors (PRRs), which leads to the activation of CCR2+ monocytes. This activation subsequently promotes the production of IL-12, a cytokine that shapes the adaptive response by polarizing T cells and influencing antibody production.",
  "mechanism_subtypes": [
    {
      "mechanism subtype": "Activation of CCR2+ monocytes",
      "evidence_refs": ["30608149"]
    },
    {
      "mechanism subtype": "Production of IL-12",
      "evidence_refs": ["30608149"]
    }
  ]
}
```

===== ROW 321 | TLR4 ligand | ok =====
```
{
  "adjuvant": "TLR4 ligand",
  "summary": "Upon binding to TLR4, the adjuvant initiates a sequence of events that culminates in the polarization of T helper 1 (Th1) cells. This is supported by the observation that TLR4 activation leads to the production of pro-inflammatory cytokines, such as IFN-γ, which are characteristic of Th1 responses. The activation of TLR4 also triggers the production of IL-12, a cytokine that promotes the differentiation of naive T cells into Th1 cells. This sequence of events is further supported by studies demonstrating the increased production of IFN-γ in response to TLR4 ligand administration, as evidenced by [28724768].",
  "mechanism_subtypes": [
    {
      "mechanism subtype": "TLR4 pathway activation",
      "evidence_refs": ["28724768"]
    },
    {
      "mechanism subtype": "Th1 cell polarization",
      "evidence_refs": ["28724768"]
    }
  ]
}
```

===== ROW 322 | TLR4 ligand adjuvant | ok =====
```
{
  "adjuvant": "TLR4 ligand adjuvant",
  "summary": "The TLR4 ligand adjuvant initiates an immune response by sensing TLR4, leading to the activation of innate immune cells such as dendritic cells [32210973]. This activation results in the production of cytokines that are essential for polarizing a Th1 and Th17 immune response, thereby enhancing the adaptive response [32210973].",
  "mechanism_subtypes": [
    {
      "mechanism subtype": "TLR4 signaling",
      "evidence_refs": ["32210973"]
    },
    {
      "mechanism subtype": "Dendritic cell activation",
      "evidence_refs": ["32210973"]
    },
    {
      "mechanism subtype": "Th1 and Th17 polarization",
      "evidence_refs": ["32210973"]

}
]
}
```

===== ROW 323 | TLR7 agonist | ok =====

{
"adjuvant": "TLR7 agonist",
"summary": "TLR7 agonist activation initiates an innate immune response by binding to its receptor and triggering the activation of downstream signaling pathways [32146328]. This leads to the production of pro-inflammatory cytokines, which in turn recruit immune cells, including dendritic cells, to the site of infection or vaccination [32146328]. The activated dendritic cells then migrate to lymphoid organs, where they present antigens to T cells, promoting T cell polarization and adaptive immune responses [32146328].",
"mechanism_subtypes": [
{
"mechanism subtype": "TLR7 activation",
"evidence_refs": ["32146328"]
},
{
"mechanism subtype": "Dendritic cell activation",
"evidence_refs": ["32146328"]
},
{
"mechanism subtype": "T cell polarization",
"evidence_refs": ["32146328"]
}
]
}

===== ROW 324 | TLR7 ligand | ok =====

```
{
"adjuvant": "TLR7 ligand",
"summary": "The TLR7 ligand initiates an immune response by engaging the TLR7 pathway, leading to the activation of innate immune cells and the subsequent promotion of Th1 responses [28724768]. This process involves the recognition of pathogen-associated molecular patterns (PAMPs) by TLR7, resulting in the production of type I interferons and the activation of dendritic cells. The activated dendritic cells then present antigens to CD4+ T cells, leading to Th1 cell polarization and the production of cytokines such as IFN-γ [28724768].",
"mechanism_subtypes": [
{
"mechanism subtype": "TLR7 pathway activation",
"evidence_refs": ["28724768"]
},
{
"mechanism subtype": "Dendritic cell activation",
"evidence_refs": ["28724768"]
},
{
"mechanism subtype": "Th1 cell polarization",
"evidence_refs": ["28724768"]
}
]
}
```

===== ROW 325 | TLR7/8 ligands | ok =====

```
{
"adjuvant": "TLR7/8 ligands",
"summary": "TLR7/8 ligands initiate an immune response by sensing viral nucleic acids, which leads to the activation of dendritic cells [32210973]. Activated dendritic cells then migrate to lymph nodes and present viral antigens to T cells,

promoting Th1-and Th17-polarized T cell responses [32210973]. These T cell responses are crucial for the development of humoral responses, including the production of antibodies against influenza viruses [32210973].",
  "mechanism_subtypes": [
    {
      "mechanism subtype": "TLR7/8 ligand sensing",
      "evidence_refs": ["32210973"]
    },
    {
      "mechanism subtype": "Dendritic cell activation",
      "evidence_refs": ["32210973"]
    },
    {
      "mechanism subtype": "Th1-and Th17-polarized T cell response",
      "evidence_refs": ["32210973"]
    },
    {
      "mechanism subtype": "Humoral response",
      "evidence_refs": ["32210973"]
    }
  ]
}
```

===== ROW 326 | TLR7/8 RNA | ok =====
```
{
  "adjuvant": "TLR7/8 RNA",
  "summary": "Upon sensing TLR7/8 RNA, peripheral blood mononuclear cells undergo strong co-stimulation of Vδ2 T cells, leading to enhanced activation and proliferation. This co-stimulation is crucial for the effective activation of Vδ2 T cells, which play a key role in innate immunity and the detection of viral infections (34315922).",
  "mechanism_subtypes": [
    {
      "mechanism subtype": "TLR7/8 sensing",
      "evidence_refs": ["34315922"]
    },
    {
      "mechanism subtype": "Vδ2 T cell co-stimulation",
      "evidence_refs": ["34315922"]
    }
  ]
}
```

===== ROW 327 | TLR7/NOD2L | ok =====
```
{
  "adjuvant": "TLR7/NOD2L",
  "summary": "Upon recognition by TLR7 and NOD2L, dendritic cells are efficiently matured and reprogrammed to produce pro-inflammatory and adaptive cytokines, leading to a potent immune response. This sequence of events is supported by studies demonstrating the adjuvant's ability to stimulate TLR7 and NOD2, with evidence cited in [32739871]. The subsequent cytokine secretion promotes the activation of immune cells and the initiation of adaptive immunity.",
  "mechanism_subtypes": [
    {
      "mechanism subtype": "Stimulation of TLR7 and NOD2",
      "evidence_refs": ["32739871"]
    },
    {
      "mechanism subtype": "Maturation and reprogramming of human dendritic cells",
      "evidence_refs": ["32739871"]
    },
    {

      "mechanism subtype": "Secretion of pro-inflammatory and adaptive cytokines",
      "evidence_refs": ["32739871"]
    }
  ]
}
```

===== ROW 328 | TLR7L | ok =====
```
{
  "adjuvant": "TLR7L",
  "summary": "The TLR7L adjuvant initially engages the TLR7 receptor, which triggers the activation of innate immune cells [32739871]. This leads to the production of cytokines, such as IFN-α, and the maturation of dendritic cells, which subsequently present antigens to T cells, promoting a Th1-mediated adaptive response.",
  "mechanism_subtypes": [
    {
      "mechanism subtype": "TLR7 receptor engagement",
      "evidence_refs": ["32739871"]
    },
    {
      "mechanism subtype": "Innate immune cell activation",
      "evidence_refs": ["32739871"]
    },
    {
      "mechanism subtype": "Dendritic cell maturation",
      "evidence_refs": ["32739871"]
    },
    {
      "mechanism subtype": "T cell polarization",
      "evidence_refs": ["32739871"]
    }
  ]
}
```

===== ROW 329 | TLR8 | ok =====
```
{
  "adjuvant": "TLR8",
  "summary": "The TLR8 adjuvant initiates an immune response by sensing the presence of monocytes, leading to the activation of dendritic cells and the production of pro-inflammatory cytokines such as IL-1β, IL-18, and IL-12p70, which in turn co-stimulate Vδ2 T cells, resulting in a direct response [34315922].",
  "mechanism_subtypes": [
    {
      "mechanism subtype": "Monocyte-dependent immune response",
      "evidence_refs": ["34315922"]
    },
    {
      "mechanism subtype": "Cytokine-driven co-stimulation of Vδ2 T cells",
      "evidence_refs": ["34315922"]
    }
  ]
}
```

===== ROW 330 | TLR9 agonists | ok =====
```json
{
  "adjuvant": "TLR9 agonists",
  "summary": "TLR9 agonists induce antitumor responses within the innate immunity arm, triggering the activation of innate immune cells such as dendritic cells (1). This activation primes the adaptive immune response, enhancing the efficacy of checkpoint blockade therapies during the effector phase of tumor-cell killing. The initiation of innate immune responses is mediated by the recognition of tumor antigens by TLR9,

leading to the production of type I interferons and the induction of tumor cell death. By modulating the innate immune response, TLR9 agonists can augment the antitumor effects of checkpoint blockade therapies.",
  "mechanism_subtypes": [
    {
      "mechanism subtype": "TLR9 recognition and activation",
      "evidence_refs": ["32547560"]
    },
    {
      "mechanism subtype": "Dendritic cell activation",
      "evidence_refs": ["32547560"]
    },
    {
      "mechanism subtype": "Type I interferon production",
      "evidence_refs": ["32547560"]
    },
    {
      "mechanism subtype": "Tumor cell death",
      "evidence_refs": ["32547560"]
    }
  ]
}
```

===== ROW 331 | Trehalose-6,6'-dimycolate (TDM) | ok =====
```
{
  "adjuvant": "Trehalose-6,6'-dimycolate (TDM)",
  "summary": "Upon binding to the MCL receptor, Trehalose-6,6'-dimycolate (TDM) initiates innate immune responses, ultimately leading to the induction of Mincle upon stimulation [23602766]. This process drives the activation of innate immune cells, including dendritic cells, which then shape the adaptive response by presenting antigens to T cells and promoting T cell polarization. The subsequent activation of T cells leads to the production of antibodies and the initiation of adaptive immune responses [23740922].",
  "mechanism_subtypes": [
    {
      "mechanism subtype": "MCL receptor activation",
      "evidence_refs": ["23602766"]
    },
    {
      "mechanism subtype": "Mincle induction",
      "evidence_refs": ["23602766", "23740922"]
    },
    {
      "mechanism subtype": "Dendritic cell activation",
      "evidence_refs": ["23740922"]
    }
  ]
}
```

===== ROW 332 | Trehalose-6,6-dibehenate (TDB) | ok =====
```json
{
  "adjuvant": "Trehalose-6,6-dibehenate (TDB)",
  "summary": "Trehalose-6,6-dibehenate (TDB) initiates an immune response by binding to Mincle, a pattern recognition receptor, leading to the production of IL-1 in an Mincle-dependent manner [23308247]. This IL-1 signaling event then activates MyD88-dependent signaling pathways, resulting in the polarization of Th1 and Th17 T cells, which contribute to the overall immune response.",
  "mechanism_subtypes": [
    {
      "mechanism subtype": "Mincle-dependent IL-1 production",
      "evidence_refs": ["23308247"]
    },

    {
      "mechanism_subtype": "MyD88-dependent Th1/Th17 responses",
      "evidence_refs": ["23308247"]
    }
  ]
}
```

===== ROW 333 | TRIF | ok =====
```
{
  "adjuvant": "TRIF",
  "summary": "TRIF, a component of the Toll-like receptor (TLR) signaling pathway, initiates an immune response by engaging the TLR4 pathway, which promotes the production of Th1 responses [21303708]. This activation enhances cellular immune responses, leading to the activation of innate immune cells, such as dendritic cells, and subsequently shapes the adaptive response [20466439]. The activation of TRIF also plays a crucial role in promoting cellular immune responses [16775309].",
  "mechanism_subtypes": [
    {
      "mechanism_subtype": "TLR signaling pathway activation",
      "evidence_refs": ["21303708", "20466439", "16775309"]
    }
  ]
}
```

===== ROW 334 | Tween 80 | ok =====
```
{
  "adjuvant": "Tween 80",
  "summary": "Tween 80 induces a suppressed Th1 response, characterized by decreased T cell activation and cytokine production, resulting in a reduced adaptive immune response [15246629]. This immunosuppressive effect is likely mediated by the adjuvant's ability to inhibit TLR4 signaling, leading to the activation of regulatory T cells and the suppression of Th1 cell polarization [14682492]. The resulting Th2-dominated response promotes the production of IgE antibodies and the activation of eosinophils, contributing to allergic inflammation [14587671].",
  "mechanism_subtypes": [
    {
      "mechanism_subtype": "TLR4 inhibition",
      "evidence_refs": ["14682492"]
    },
    {
      "mechanism_subtype": "Regulatory T cell activation",
      "evidence_refs": ["14682492"]
    },
    {
      "mechanism_subtype": "Th1 cell polarization suppression",
      "evidence_refs": ["15246629"]
    },
    {
      "mechanism_subtype": "Th2-dominated response",
      "evidence_refs": ["14587671"]
    }
  ]
}
```

===== ROW 335 | U-Omp19 | ok =====
```
{
  "adjuvant": "U-Omp19",
  "summary": "U-Omp19 stimulates an immune response by increasing the frequency of RBD-specific geminal center B cells and plasmablasts, which subsequently produce antibodies. This is followed by the induction of RBD-specific Th1 and CD8+ T-cell

responses in spleens and lungs, as supported by the increase in these cells in these organs. [35296091]. The immune response is characterized by the activation of innate immune cells, such as dendritic cells, which recognize the adjuvant and initiate an immune response. [35296091].",
  "mechanism_subtypes": [
    {
      "mechanism subtype": "Geminal center B cell activation and plasmablast differentiation",
      "evidence_refs": ["35296091"]
    },
    {
      "mechanism subtype": "Th1 T-cell induction",
      "evidence_refs": ["35296091"]
    },
    {
      "mechanism subtype": "CD8+ T-cell induction",
      "evidence_refs": ["35296091"]
    },
    {
      "mechanism subtype": "Innate immune cell activation (dendritic cells)",
      "evidence_refs": ["35296091"]
    }
  ]
}
```

===== ROW 336 | UM-1098 | ok =====
```
{
  "adjuvant": "UM-1098",
  "summary": "UM-1098 stimulates a robust Th17 response by inducing high levels of Th17 polarizing cytokines, including IL-6, IL-1β, IL-23, IL-12p70, IL-4, and TNF-α, thereby promoting the differentiation of Th17 cells and the production of antibodies. This results in enhanced immune protection against pathogens. [38844494]",
  "mechanism_subtypes": [
    {
      "mechanism subtype": "Th17 response",
      "evidence_refs": ["38844494"]
    }
  ]
}
```

===== ROW 337 | Vaxfectin | ok =====
```
{
  "adjuvant": "Vaxfectin",
  "summary": "Vaxfectin initiates an immune response by being sensed by pattern recognition receptors (PRRs), leading to the activation of innate immune cells, including dendritic cells. Upon activation, dendritic cells induce a T cell response characterized by the polarization of T cells towards a Th1 or Th2 phenotype, resulting in the production of specific antibodies. The precise mechanisms of Vaxfectin-induced immune responses are supported by [21118032].",
  "mechanism_subtypes": [
    {
      "mechanism subtype": "Pattern recognition receptor (PRR) sensing",
      "evidence_refs": ["21118032"]
    },
    {
      "mechanism subtype": "Dendritic cell activation",
      "evidence_refs": ["21118032"]
    },
    {
      "mechanism subtype": "T cell polarization",
      "evidence_refs": ["21118032"]
    },

    {
      "mechanism subtype": "Antibody production",
      "evidence_refs": ["21118032"]
    }
  ]
}
```

===== ROW 338 | VISA | ok =====
```json
{
  "adjuvant": "VISA",
  "summary": "The VISA adjuvant initiates an immune response by activating NF-κB and IRF3, leading to the production of type I interferons (IFNs) [21303708]. This activation triggers a downstream signaling cascade that ultimately results in the production of IFNs, which play a crucial role in the innate immune response. The subsequent production of IFNs helps to induce an antiviral state in nearby cells, thereby limiting the spread of viral infections. Additionally, the activation of NF-κB and IRF3 also leads to the production of pro-inflammatory cytokines, which contribute to the recruitment of immune cells to the site of infection [21303708].",
  "mechanism_subtypes": [
    {
      "mechanism subtype": "Activation of NF-κB and IRF3",
      "evidence_refs": ["21303708"]
    },
    {
      "mechanism subtype": "Production of type I interferons (IFNs)",
      "evidence_refs": ["21303708"]
    }
  ]
}
```

===== ROW 339 | VP6 | ok =====
```
{
  "adjuvant": "VP6",
  "summary": "The VP6 adjuvant is initially sensed by pattern recognition receptors (PRRs), such as TLRs, leading to the activation of innate immune cells like dendritic cells. These dendritic cells efficiently uptake and present VP6, which subsequently shapes the adaptive response by promoting T cell polarization and antibody production [31083495]. This process is critical for eliciting a robust and specific immune response against the antigen.",
  "mechanism_subtypes": [
    {
      "mechanism subtype": "TLR activation",
      "evidence_refs": ["31083495"]
    },
    {
      "mechanism subtype": "Dendritic cell activation and antigen presentation",
      "evidence_refs": ["31083495"]
    },
    {
      "mechanism subtype": "T cell polarization",
      "evidence_refs": ["31083495"]
    },
    {
      "mechanism subtype": "Antibody production",
      "evidence_refs": ["31083495"]
    }
  ]
}
```

===== ROW 340 | VSA-1 | ok =====
```

{
  "adjuvant": "VSA-1",
  "summary": "The VSA-1 adjuvant initiates a sequence of immunological events starting with the recognition of pathogen-associated molecular patterns (PAMPs) by pattern recognition receptors (PRRs), such as TLRs, leading to the activation of innate immune cells like dendritic cells [36146461]. Subsequently, dendritic cells process and present antigens to T cells, resulting in the polarization of T cells towards either a Th1 or Th2 phenotype, promoting a mixed and antigen-specific immune response [31657920]. This mixed immune response is further amplified by the adjuvant's ability to stimulate both humoral and cellular immune responses, including the production of antibodies and the activation of cytotoxic T cells [36578488].",
  "mechanism_subtypes": [
    {
      "mechanism subtype": "Recognition of PAMPs by PRRs",
      "evidence_refs": ["36146461"]
    },
    {
      "mechanism subtype": "Dendritic cell activation",
      "evidence_refs": ["36146461"]
    },
    {
      "mechanism subtype": "T cell polarization",
      "evidence_refs": ["31657920"]
    },
    {
      "mechanism subtype": "Stimulation of humoral immune responses",
      "evidence_refs": ["36578488"]
    },
    {
      "mechanism subtype": "Stimulation of cellular immune responses",
      "evidence_refs": ["36578488"]
    }
  ]
}
```

===== ROW 341 | VSA-2 | ok =====

```
{
  "adjuvant": "VSA-2",
  "summary": "The VSA-2 adjuvant initiates an immune response by being sensed by pattern recognition receptors (PRRs), such as TLRs, which triggers the activation of innate immune cells like dendritic cells [36318612]. Upon activation, dendritic cells process and present antigenic peptides to T cells, leading to T cell polarization and subsequent antibody production [36318612]. This complex interplay of innate and adaptive immune responses shapes the overall immune response to the VSA-2 adjuvant.",
  "mechanism_subtypes": [
    {
      "mechanism subtype": "TLR sensing and innate cell activation",
      "evidence_refs": ["36318612"]
    },
    {
      "mechanism subtype": "Dendritic cell activation and antigen presentation",
      "evidence_refs": ["36318612"]
    },
    {
      "mechanism subtype": "T cell polarization and antibody production",
      "evidence_refs": ["36318612"]
    }
  ]
}
```

===== ROW 342 | VSA-2 (5b) | ok =====

{
  "adjuvant": "VSA-2 (5b)",
```

"summary": "Upon recognition by pattern recognition receptors (PRRs), such as TLRs, the adjuvant VSA-2 triggers the activation of innate immune cells, including dendritic cells, which then migrate to lymphoid organs and present antigens to T cells. This leads to the polarization of T cells, with a shift towards Th1 or Th2 responses, ultimately resulting in the production of specific antibodies and cellular immune responses. The subsequent immune response is modulated by the cytokine environment, which is influenced by the adjuvant-induced activation of immune cells.",
  "mechanism_subtypes": [
    {
      "mechanism subtype": "Recognition by PRRs",
      "evidence_refs": ["32101001"]
    },
    {
      "mechanism subtype": "Activation of innate immune cells",
      "evidence_refs": ["32101001"]
    },
    {
      "mechanism subtype": "Dendritic cell activation and migration",
      "evidence_refs": ["32101001"]
    },
    {
      "mechanism subtype": "T cell polarization",
      "evidence_refs": ["32101001"]
    },
    {
      "mechanism subtype": "Cytokine modulation of the immune response",
      "evidence_refs": ["32101001"]
    }
  ]
}

===== ROW 343 | wtLT | ok =====
```
{
  "adjuvant": "wtLT",
  "summary": "The wtLT adjuvant initiates an immune response by sensing TLR4, leading to the activation of innate immune cells such as dendritic cells. This activation triggers the production of pro-inflammatory cytokines, which in turn polarize T cells towards a Th1 response. The resulting Th1 response is characterized by the production of IFN-gamma and the elimination of intracellular pathogens. [11803061].",
  "mechanism_subtypes": [
    {
      "mechanism subtype": "TLR4 pathway activation",
      "evidence_refs": ["11803061"]
    },
    {
      "mechanism subtype": "Dendritic cell activation",
      "evidence_refs": ["11803061"]
    },
    {
      "mechanism subtype": "Th1 response polarization",
      "evidence_refs": ["11803061"]
    }
  ]
}
```

===== ROW 344 | YSFKPMPLaR (EP54) | ok =====
```
{
  "adjuvant": "YSFKPMPLaR (EP54)",
  "summary": "Upon recognition by pattern recognition receptors (PRRs) like TLRs, the adjuvant YSFKPMPLaR (EP54) induces an innate immune response, activating dendritic cells and leading to the production of pro-inflammatory cytokines [19428909]. This subsequently shapes the adaptive response by polarizing T cells towards a Th1 or Th17 phenotype, promoting the production of specific antibodies and enhancing vaccine
```

efficacy. The conformational bias of the adjuvant ensures a response-selective immune response, minimizing off-target effects and improving vaccine potency.",
  "mechanism_subtypes": [
    {
      "mechanism subtype": "TLR recognition",
      "evidence_refs": ["19428909"]
    },
    {
      "mechanism subtype": "Dendritic cell activation",
      "evidence_refs": ["19428909"]
    },
    {
      "mechanism subtype": "T cell polarization (Th1/Th17)",
      "evidence_refs": ["19428909"]
    }
  ]
}
```

===== ROW 345 | zymosan | ok =====
```
{
  "adjuvant": "zymosan",
  "summary": "Zymosan initiates an immune response by activating the complement system, particularly through the alternative pathway, leading to the recruitment and activation of macrophages and dendritic cells, which subsequently present antigens to T cells, polarizing them towards a Th1 response and driving antibody production.",
  "mechanism_subtypes": [
    {
      "mechanism subtype": "Complement activation",
      "evidence_refs": ["11380697"]
    },
    {
      "mechanism subtype": "Recruitment and activation of antigen-presenting cells (APCs)",
      "evidence_refs": ["11380697"]
    }
  ]
}
```

===== ROW 346 | α-GalCer | ok =====
```json
{
  "adjuvant": "α-GalCer",
  "summary": "The α-GalCer adjuvant initiates an immune response by activating the NLRP3 inflammasome, leading to the release of pro-inflammatory cytokines and the formation of a depot for slow antigen release. This process broadens the immune response to include IgG2c antibody production, and stimulates the production of IL-18, which plays a stimulatory role in adjuvant effects.",
  "mechanism_subtypes": [
    {
      "mechanism subtype": "Activation of NLRP3 inflammasome",
      "evidence_refs": ["28483194"]
    },
    {
      "mechanism subtype": "Depot formation for slow antigen release",
      "evidence_refs": ["28483194"]
    },
    {
      "mechanism subtype": "Broadening of immune responses to include IgG2c",
      "evidence_refs": ["35095921"]
    },
    {
      "mechanism subtype": "Stimulatory role of IL-18",
      "evidence_refs": ["26811064"]

```
    }
  ]
}
```

===== ROW 347 | α-tocopherol | ok =====
```json
{
  "adjuvant": "α-tocopherol",
  "summary": "The α-tocopherol adjuvant, AS03, initiates an immune response by increasing antigen loading in monocytes and recruiting granulocytes in the dLNs, which enhances the expression of cytokines such as CCL2, CCL3, IL-6, CSF3, and CXCL1. [21256188] This cytokine modulation modulates the innate immune response, leading to a subsequent adaptive immune response characterized by increased antibody production. [21256188].",
  "mechanism_subtypes": [
    {
      "mechanism subtype": "Antigen loading and recruitment of granulocytes",
      "evidence_refs": ["21256188"]
    },
    {
      "mechanism subtype": "Cytokine modulation (CCL2, CCL3, IL-6, CSF3, and CXCL1)",
      "evidence_refs": ["21256188"]
    }
  ]
}
```
