# Supplementary figures and images for: Vaxjo 2.0: An ontology- and large language model-powered knowledge base of vaccine adjuvants and mechanisms
Source: Front Cell Infect Microbiol. 2026 Jun 5;16:1763384. doi: 10.3389/fcimb.2026.1763384 (PMC13279671; doi:10.3389/fcimb.2026.1763384)

Overlap of Immune Mechanism Families Across Vaccine Adjuvants

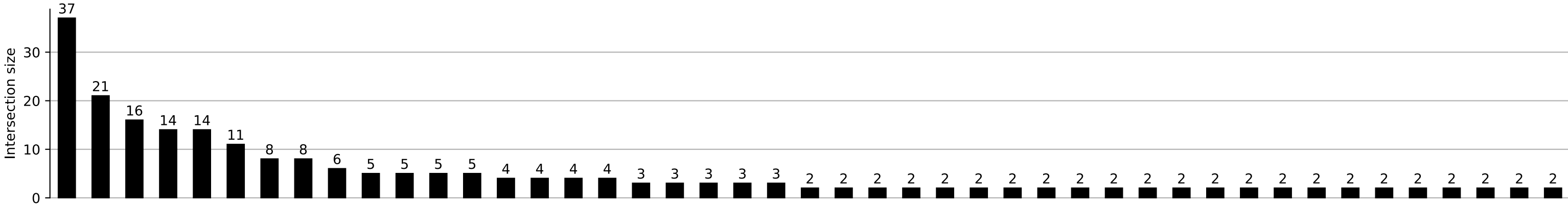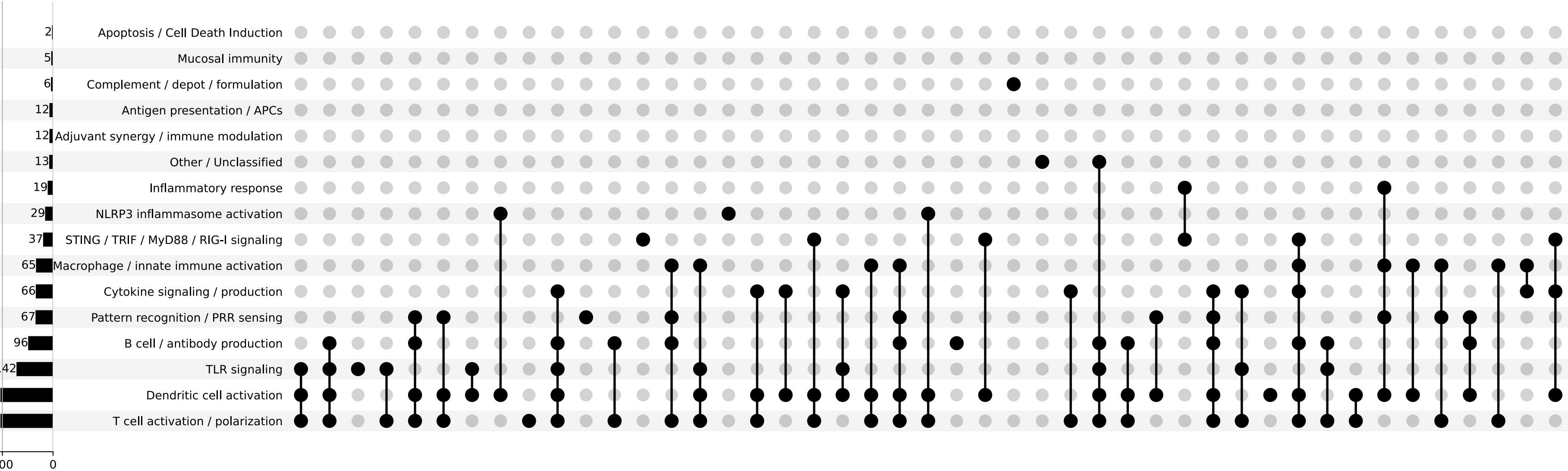

Supplement: Supplementary File 7 — UpSet plot of higher-order mechanism overlaps across adjuvants. An UpSet plot visualizing higher-order intersections among mechanism families across all adjuvants. This figure complements Figure 5 by systematically displaying complex multi-mechanism overlaps that are not easily captured in heatmap form. [file Supplementaryfile7.pdf]
